# Genome-Wide Analysis of the Late Embryogenesis Abundant (LEA) and Abscisic Acid-, Stress-, and Ripening-Induced (ASR) Gene Superfamily from *Canavalia rosea* and Their Roles in Salinity/Alkaline and Drought Tolerance

**DOI:** 10.3390/ijms22094554

**Published:** 2021-04-27

**Authors:** Ruoyi Lin, Tao Zou, Qiming Mei, Zhengfeng Wang, Mei Zhang, Shuguang Jian

**Affiliations:** 1 Key Laboratory of South China Agricultural Plant Molecular Analysis and Genetic Improvement & Guangdong Provincial Key Laboratory of Applied Botany, South China Botanical Garden, Chinese Academy of Sciences, Guangzhou 510650, China; linry@scbg.ac.cn (R.L.); zoutao@scbg.ac.cn (T.Z.); qmei4597@scbg.ac.cn (Q.M.); wzf@scbg.ac.cn (Z.W.); 2 University of the Chinese Academy of Sciences, Beijing 100039, China; 3 Center of Economic Botany, Core Botanical Gardens, Chinese Academy of Sciences, Guangzhou 510650, China; 4 CAS Engineering Laboratory for Vegetation Ecosystem Restoration on Islands and Coastal Zones, South China Botanical Garden, Chinese Academy of Sciences, Guangzhou 510650, China; 5 Key Laboratory of Vegetation Restoration and Management of Degraded Ecosystems, Center for Plant Ecology, Core Botanical Gardens, Chinese Academy of Sciences, Guangzhou 510650, China; 6 Southern Marine Science and Engineering Guangdong Laboratory (Guangzhou), Guangzhou 511458, China

**Keywords:** late embryogenesis abundant protein, abscisic acid-, stress-, and ripening-induced protein, salinity/alkaline, drought, *Canavalia rosea* (Sw.) DC

## Abstract

*Canavalia rosea* (bay bean), distributing in coastal areas or islands in tropical and subtropical regions, is an extremophile halophyte with good adaptability to seawater and drought. Late embryogenesis abundant (LEA) proteins typically accumulate in response to various abiotic stresses, including dehydration, salinity, high temperature, and cold, or during the late stage of seed development. Abscisic acid-, stress-, and ripening-induced (ASR) genes are stress and developmentally regulated plant-specific genes. In this study, we reported the first comprehensive survey of the LEA and ASR gene superfamily in *C. rosea*. A total of 84 *CrLEA*s and three *CrASR*s were identified in *C. rosea* and classified into nine groups. All CrLEAs and CrASRs harbored the conserved motif for their family proteins. Our results revealed that the *CrLEA* genes were widely distributed in different chromosomes, and all of the *CrLEA*/*CrASR* genes showed wide expression features in different tissues in *C. rosea* plants. Additionally, we introduced 10 genes from different groups into yeast to assess the functions of the *CrLEA*s/*CrASR*s. These results contribute to our understanding of *LEA/ASR* genes from halophytes and provide robust candidate genes for functional investigations in plant species adapted to extreme environments.

## 1. Introduction

Abiotic stresses, such as high temperature, high salinity/alkaline, extreme aridity, and cold or freezing, influence plant growth and often cause deficit in cellular water, thereby causing a series of changes, including biochemical alterations in gene expression, osmolytes, and the accumulation of specific proteins involved in the stress response. Late embryogenesis abundant proteins (LEAs) and abscisic acid-, stress-, and ripening-induced proteins (ASRs) are supposed to play crucial roles in the processes of drought resistance or other water-deficit stresses [1,2,3,4,5]. *LEA* genes have been characterized in plants ranging from algae to higher plants, as well as in invertebrates, fungi, and bacteria [6]. LEA proteins, which are intrinsically disordered proteins, often have the feature of high hydrophilicity and are identified as hydrophilins [7,8]. LEA proteins play protective roles during cell exposure to different abiotic stresses, and these roles are associated with antioxidant, metal ion binding, membrane and protein stabilization, hydration buffering, and DNA and RNA interaction properties [9,10]. Unlike LEA proteins, ASR proteins are plant specific and possess apparent features that are typically absent in Brassicaceae species [11]. Numerous reports have indicated that plant ASRs have important functions in response to water-deficit stress in many plant species, basically acting as transcription factors or chaperones [12,13]. Some previous reports also classified plant ASRs into the LEA superfamily [8,14].

LEAs are mainly composed of many hydrophilic amino acids with a repeated arrangement that forms a highly hydrophilic domain with high heat-stability characteristics [8]. The first plant LEA protein was initially isolated from cotton seeds during the late embryogenesis stage in 1983 [15]. Since then, numerous LEA members have been extensively characterized, and thousands of LEA sequences have been gathered in a dedicated database called LEAPdb [16]. In most plants, the LEA protein family consists of large and highly diverse polypeptides, and different reports have classified them into specific subfamilies based on various categorization features [17]. To date, most studies have widely accepted that based on the conserved motifs, amino acid composition, and phylogenetic relationships, plant LEA proteins can be classified into eight subgroups, including LEA_1, LEA_2, LEA_3, LEA_4, LEA_5, LEA_6, dehydrin (DHN), and seed maturation protein (SMP) [18,19]. 

LEA proteins are reported to be involved in response to various stress conditions and plant development. Many reports have indicated that the majority of plant *LEA* mRNAs are induced by various abiotic stresses, such as drought, high salinity, low temperature, oxidation, and osmotic stress [6,18,20]. Some reports have also indicated that in addition to transcriptional regulation, LEA proteins undergo translational or posttranslational modifications, reflecting the complexity of the regulation of these proteins at various levels [21,22]. Basically, LEA proteins are predicted to keep intrinsically disordered proteins in the fully hydrated state, which can then be folded in the water-deficit state and acquire α-helical structures. The ordered LEAs can act as molecular chaperones to bind enzymes, membranes, DNA/RNA, water or ions, and reactive oxygen species (ROS), therefore playing crucial roles in stabilizing proteins/membranes and maintaining cellular environmental homeostasis under abiotic stress [23]. Many studies have characterized several *LEA* gene families from a few sequenced genomes of plant species, particularly from plant species in specialized habitats, such as halophytes [24], xerophytes [25,26], heat- or freeze-tolerant plants [27,28], or cash crops with strong adversity resistance [24,25,26,27,28,29]. In general, the LEA families that have been identified and studied all contain dozens of members with similar structural and functional plasticity, with the typical evolutionary features and genomic diversification resulting from the adaptation of plants to water-limited environments [20]. 

The ASR proteins, by contrast, seem to be plant-specific with a small gene family [11,29]. ASRs also have a low molecular weight and exhibit heat-stable and highly hydrophilic characteristics in plants [30]. As observed for plant LEAs, many reports have found that ASRs are also functionally involved in abiotic stress responses, acting as molecular chaperons [31], osmotic adjustment proteins [32], metal-binding protein [33], and antioxidation or detoxification proteins [34,35]. In addition, some ASRs also possess unique functions as transcription factors [12,36]. Due to their highly similar biochemical properties and biological functions, some recent studies have also classified ASRs as LEA proteins in the LEA_7 group [11,37,38]. Numerous reports also indicate that plant *ASR* genes respond to ABA and abiotic stresses such as water deficit, salt, drought, cold, and osmotic stress [11,13].

*Canavalia rosea* (Fabaceae) is a wild legume occurring on coastal sand dunes and is mainly distributed in tropical and subtropical regions. In addition to the edible and medicinal characteristics of the mature bean [39], *C. rosea* also demonstrates excellent growth potential under saline-alkali and high-temperature arid coral reef environments, with great advantages in saline-alkali tolerance and drought resistance [40]. Therefore, as a typical symbiotic nitrogen-fixing legume, *C. rosea* constitutes a superior wild plant resource and plays important roles in the island greening, sand fixation, and revegetation on tropical and subtropical coral islands and coastal zones. There is no doubt that *C. rosea* has developed elaborate mechanisms to adapt to the high sanity/alkaline drought stress caused by saltwater intrusion or freshwater deficit in sand dunes or coral reefs, and thus the identification of stress-relevant genes in this species is quite necessary. In the present study, based on the genome sequence of *C. rosea*, we explored the number of LEA/ASR proteins and their families as well as their structural characterization, gene chromosomal location, and tissue- or habitat- specific expression patterns in *C. rosea*. The availability of a whole-genome sequence of *C. rosea* will facilitate genome-wide analysis for identifying the evolutionary relationships of *C. rosea* LEAs/ASRs with related leguminous species. Additionally, the genome provides a source for mining the genetic resources, including *LEA*/*ASR* genes, of *C. rosea* plants, which have evolved to survive under extreme stress.

## 2. Results

### 2.1. Identification of the C. rosea LEA Family and ASR Family

Based on the Hidden Markov model profile search and protein Blast research results, a total of 84 CrLEA and three CrASR members were identified and annotated from the *C. rosea* genome database. The set of *C. sativa* LEA proteins included three LEA_1 members, 60 LEA_2 members, six LEA_3 members, two LEA_4 members, two LEA_5 members, two LEA_6 members, five dehydrins, four SMP members, and three ASR members (Table 1 and Table S2). The predicted *CrLEA*s and *CrASR*s were named according to their subfamilies and the *C. rosea* gene nomenclature system (Table 1). The sequence information of all 84 *CrLEA* and three *CrASR* genes can be found in Table S2.

The physiochemical properties were assessed using a series of bioinformatics programs. In this large and varied family, the protein lengths of all CrLEAs and CrASRs ranged from 82 to 499 aa, with a molecular weight (MW) ranging from 8.58 (CrDHN1) to 57.65 (CrLEA2-35) kDa (Table 1). The theoretical isoelectric points (PI) of the CrLEAs and CrASRs ranged from 4.77 (CrSMP3) to 10.30 (CrLEA2-46), and most of them (67 members, 75%) were considered to be basic (pI > 7). The instability index ranged between 62.03 (CrLEA2-37) and −5.30 (CrDHN5), and averaged around 39, and a small number of members (38 members, 43%) had a high instability index (II) (>40), which indicated that most of these members might be stable. Most CrLEAs and CrASRs presented calculated grand average of hydropathy (GRAVY) values of less than 0, implying that these proteins are quite hydrophilic. Conversely, the aliphatic index (AI) assessment showed that most members had lower values, indicating that only a small number of these proteins appeared to be lipophilic or hydrophobic. We calculated the contents of disordered amino acids in all CrLEAs and CrASRs according to the prediction of PrDOS, and the results indicated that most of CrLEA_2s and all five CrSMPs had the lower values (<30%) than other CrLEAs or CrASRs (>40% in most members) (Table 1), which is coincided with LEA_2 subfamily has been considered atypical because of containing more hydrophobic amino acids and possessing more defined secondary structure in solution than the other LEA subfamilies [20]. We also summarized the 3D structures of all CrLEAs and CrASRs (Figure S1), and the results showed that all of CrLEA_2s comprised several β-sheets and presented defined secondary structure, while the other CrLEAs and CrASRs, except that two CrLEA_4s contained two α-helices, showed disordered structures, which was in complete accordance with the features of intrinsically disordered proteins (IDPs) [23]. The detailed disorder profile plots of all CrLEAs and CrASRs predicted by PrDOS program were also summarized in Supplementary Materials Figure S2.

The subcellular localization prediction revealed that almost all of the CrLEA and CrASR proteins were present in all subcellular compartments, including the nucleus, cytoplasm, chloroplast, and mitochondria, and even extracellular regions or secretory pathways. Exceptionally, the dehydrin subfamily was more likely to be located in the cell nucleus, and the SMP subfamily was more likely to exist in the cytoplasm. Regarding the CrASR members, they were predicted locating both in the nucleus and in the cytoplasm (Table 1), which is consistent with their possible functions being transcription factors or chaperones, as previous reports in other species [11,12,14].

### 2.2. Phylogenetic Analysis of CrLEA Proteins and CrASR Proteins

**Figure 1:**
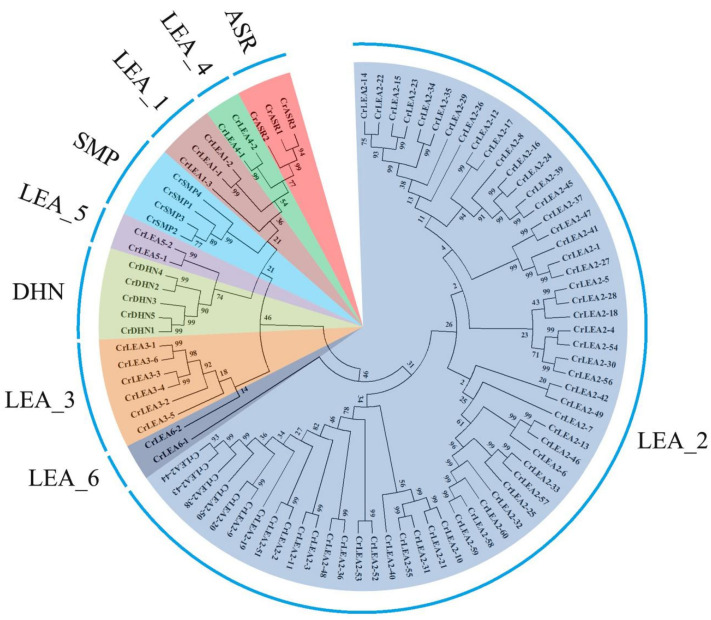
Phylogenetic analysis of the LEA and ASR proteins in *Canavalia rosea*. LEA and ASR subfamilies were distinguished by different colors. The unrooted tree was generated by ClustalW in MEGA6 with the sequences of the 87 *C. rosea* LEA and ASR proteins.

To systematically classify the *C. rosea LEA* and *ASR* genes and uncover the evolutionary relationships among this diverse gene family, an unrooted phylogenetic tree of 84 CrLEA and three CrASR members was constructed with MEGA6 using neighbor-joining analysis based on the sequences of the CrLEA and CrASR proteins (Figure 1). The members of this superfamily, including a total of 87 members, clustered together according to their Pfam domains and were classified into two main branches: The LEA_2 subfamily was the largest, with only one distinctive member, CrLEA6-1; the other LEA_1, LEA_3, LEA_4, LEA_5, SMP, DHN, ASR members, and CrLEA6-2 formed another branch. Interestingly, according to the evolutionary relationships, the CrASR subfamily showed a closer relationship with the LEA_4 and LEA_1 subfamilies. There were 29 sister gene pairs in the evolutionary tree, with a bootstrap support value >90% (Figure 1). Among them, nine sub-branches, including *CrLEA2-16/CrLEA2-24/CrLEA2-39/CrLEA2-45*, *CrLEA2-37/CrLEA2-47/CrLEA2-41/CrLEA2-1/CrLEA2-27*, *CrLEA2-13/CrLEA2-46/CrLEA2-6*, *CrLEA2-33/CrLEA2-57/CrLEA2-25/CrLEA2-32/CrLEA2-60/CrLEA2-58/CrLEA2-59*, *CrLEA2-10/CrLEA2-21/CrLEA2-31/CrLEA2-55*, *CrLEA2-50/CrLEA2-38/CrLEA2-43/CrLEA2-44*, *CrLEA3-4/CrLEA3-3/CrLEA3-6/CrLEA3-1*, *CrDHN1/CrDHN5/CrDHN3/CrDHN2/CrDHN4*, and *CrASR2/CrASR1/CrASR3*, all showed bootstrap support values over 90% on each branch (Figure 1). 

As plant-specific ASRs have been considered as a small gene family independent from the plant LEA family in some previous reports [11], here we also constructed a single CrASR phylogenetic tree using MEGA6 (Figure S3). The sequence information of all plant ASR proteins is presented in Table S2. Our result indicated that the ASR members did not show strict evolutionary relationships within the same species, or even within the same family. The sequences varied vastly in the same species ASRs, which indicated that this family was not particularly evolutionarily conserved (Figure S3).

### 2.3. Gene Structures and Conserved Motifs of CrLEAs and CrASRs in C. rosea

**Figure 2:**
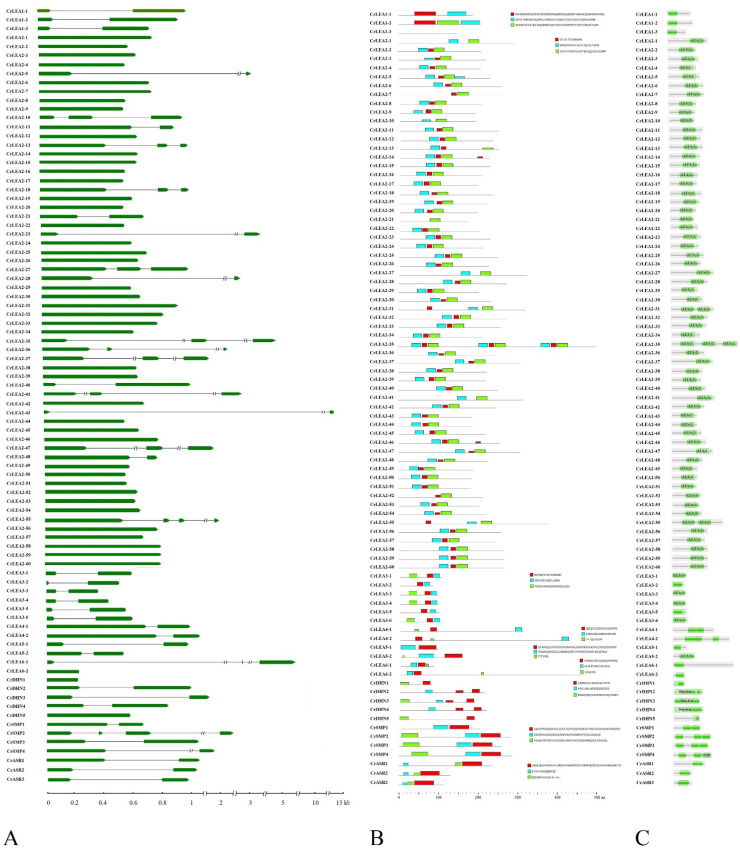
Exon–intron structures of *CrLEA/CrASR* genes and conserved motifs in CrLEA/CrASR proteins. (**A**) The lengths and positions of introns and exons were shown with black lines and thick green arrows for total of *CrLEA/CrASR* genes ordered by their names. (**B**) Conserved motifs of *C. rosea* CrLEA/CrASR proteins in the same subfamily, and these motifs were identified using Multiple EM for Motif Elicitation (MEME) and boxes with different colors represent different motifs. (**C**) Predications of CrLEA/CrASR proteins by the Pfam database (http://pfam.xfam.org/, accessed on 1 March 2021). The conserved pfam domains were marked with green boxes.

The structure of genes should be of important reference significance for estimating their evolutionary relationships and functional expression patterns, and the conserved motif analyses of proteins are greatly valuable for determining their biochemical functions. To investigate the structural characteristics of *CrLEA*s and *CrASR*s, the exon–intron structures (Figure 2A) and conserved motifs (Figure 2B) of 87 member genes or proteins were analyzed. Gene structure analysis showed that the majority of the *CrLEA*s and *CrASR*s contained 0 or one intron, and only 12 *CrLEA* genes (*CrLEA2-10*, *CrLEA2-13*, *CrLEA2-18*, *CrLEA2-27*, *CrLEA2-35*, *CrLEA2-36*, *CrLEA2-37*, *CrLEA2-41*, *CrLEA2-47*, *CrLEA2-55*, *CrLEA6-1*, and *CrSMP2*) possessed two or three introns (Figure 2A). The *CrLEA_2* subfamily had the most intronless members, which might indicate that these members probably evolved recently through retrotransposon processes under a certain degree of evolutionary pressure imposed by natural selection. Meanwhile, in each subfamily, some members possessed similar exon–intron structures and intron and exon lengths, which also indicated that these genes possessed closer evolutionary relationships than the other members.

The conserved motifs of the CrLEA and CrASR proteins were analyzed and compared (Figure 2B). Due to the smaller molecular lengths of this protein superfamily, we separately analyzed three independent conserved motifs in each CrLEA and CrASR subfamily. These conserved motifs are listed in Table S3. The results suggested that members of each subfamily possessed similar specific conserved motifs, implying functional specificities of different subfamily proteins. We also compared the conserved motifs with the Pfam domain searched by the Pfam database, and the preliminary results suggested that the conserved motif prediction was consistent with the Pfam domain localization in most CrLEA or CrASR members (Figure 2C).

### 2.4. Chromosomal Locations and Evolutionary Characterization of CrLEAs and CrASRs

**Figure 3:**
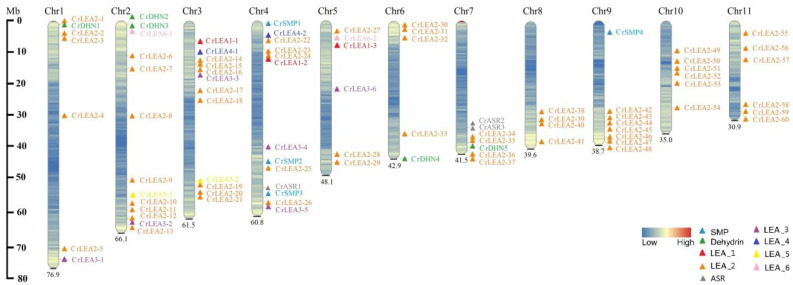
Chromosomal distribution of *CrLEA/CrASR* genes in *C. rosea*. Different colors showed different subfamilies, and the scale of the chromosome is in millions of bases (Mb).

To further investigate the evolutionary relationships and genomic position of the *CrLEA/CrASR* superfamily, we mapped their chromosomal locations according to their gene locus in Table 1. Most of the *CrLEAs/CrASRs* were extensively and evenly distributed on the 11 chromosomes. High-density *LEA_2* gene clusters were identified in certain chromosomal regions, including chromosomes 2, 3, 7, 8, 9, 10, and 11 (Figure 3), which indicated that these cluster-chromosomal locations of the *CrLEA_2* genes may be the result of gene duplication.

Gene family expansion occurs via three mechanisms: Tandem duplication, segmental duplication, and whole-genome duplication (WGD). Tandem duplication and segmental duplication are essential for the evolution of gene families in order to adapt to varying environmental conditions. The results showed that 19 pairs of segmentally duplicated genes and seven pairs of tandem duplicated genes were identified in 84 *C. rosea* LEA genes (Table 2), and one tandem duplication gene cluster was found on the end of the 11th chromosome (CrLEA2-58/CrLEA2-59/CrLEA2-60) (Figure 3).

**Figure 4:**
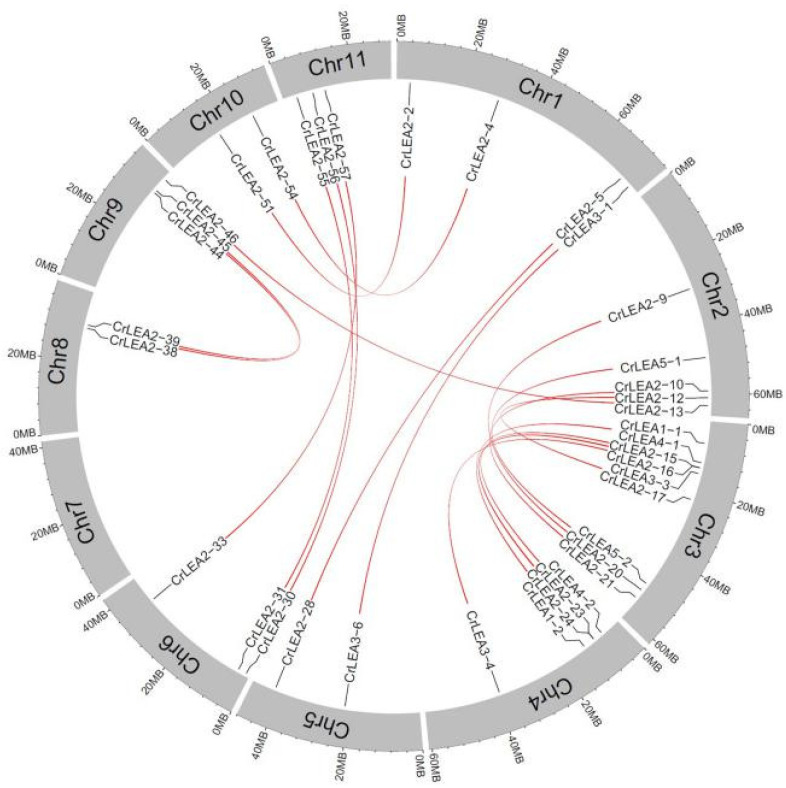
Segmental duplication of *CrLEA/CrASR* genes in *C. rosea* genome.

Synonymous (Ks) and nonsynonymous (Ka) values were calculated to explore the selective pressures on duplicated *CrLEA*s based on all of the nucleotide sequences of *CrLEA*s. In general, when the ratio is greater than 1, the replicated gene is under positive selection, whereas the replicated gene is neutrally selected when the ratio is equal to 1, and the replicated gene is under purifying selection when the ratio is less than 1. The results revealed that most of the *CrLEA*s possessed Ka/Ks ratios greater than 0.1. The Ka/Ks for a paralogous gene pair of *CrLEA*s was 0.101–0.750 with a mean value of ~0.307. These results indicated that they appeared to have undergone extensive purifying selection during evolution and might preferentially conserve function and structure under selective pressure (Table 2). The distribution of segmentally duplicated *CrLEA* genes in the *C. rosea* chromosomes is simply illustrated in Figure 4.

### 2.5. Cis-Regulatory Element Analyses of CrLEAs and CrASRs

**Figure 5:**
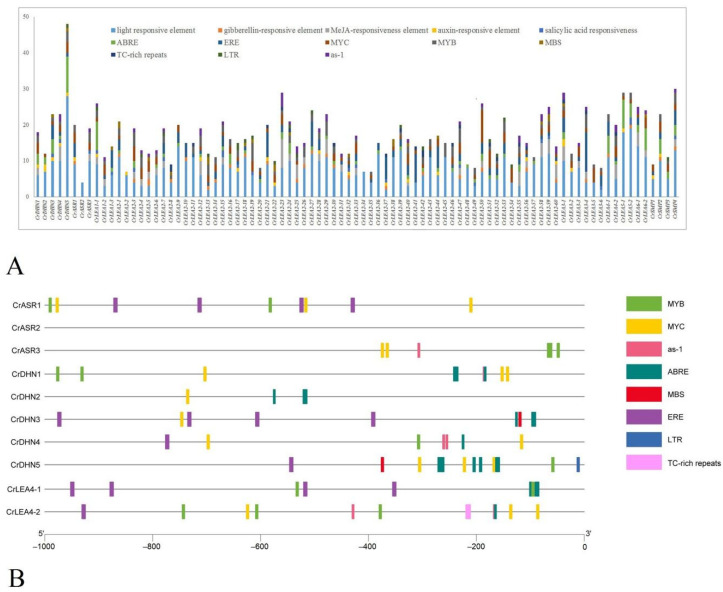
Prediction of *cis*-regulatory elements in the 1000-bp upstream regulatory regions of *CrLEA/CrASR* genes. (**A**) Summaries of the twelve *cis*-regulatory elements in the 87 *CrLEA/CrASR* promoter regions. (**B**) Distribution of the eight *cis*-regulatory elements (MYB, MYC, as-1, ABRE, MBS, ERE, LTR, and TC-rich repeat) in the 10 *CrLEA/CrASR* genes promoter regions. The scale bar represents 200 bp.

The *CrLEA* members showed a duplication-prone pattern (Table 2, Figure 4), and in some subfamily members, the *CrLEA/CrASR* members often possessed similar gene structures and conserved amino acid motifs (Figure 2), which suggested that these members might have functional redundancy or superposition. This is also a process of adaptive evolution in which plant species promote their adaptability and improve their survival under extreme environments or special habitats by altering their DNA. Compared with the numbers or structures of genes, the promoter regions of functional genes showed more variability, which also created a more elaborate and more efficient regulatory mechanism for exercising the biological functions of the genes. To further understand the potential functions and regulatory mechanisms of *CrLEAs* and *CrASRs*, especially for exploring the possible roles of this gene family for the adaptation of *C. rosea* to high salinity and drought, we analyzed the promoter regions of the upstream 1000 bp of 87 putative *CrLEAs* and *CrASRs*, which are believed to play important roles in regulating the spatial and temporal expression of genes. In general, a total of 13 *cis*-regulatory elements were summarized in this study, including light response elements, gibberellin-responsive elements, MeJA-responsive elements, auxin response elements, salicylic acid responsiveness elements, ABRE, ERE, MYC, MYB and MBS, TC-rich repeats, LTR, and as-1. The results are shown in Figure 5A, and the interpretation and localization of these *cis*-regulatory elements are provided in Table S4.

In addition, ASRs and dehydrins have been reported to be crucial in the tolerance to different abiotic stresses, including drought, high salinity/alkaline, or extreme temperatures [11,41]. The LEA_4 subfamily has been suggested to have a close association with the evolution of desiccation tolerance in plants [20]. In this study, we paid special attention to the promoter regions of *CrASR*s (three members), *CrDHN*s (five members), and *CrLEA_4*s (two members). We summarized the abiotic stress-related *cis*-regulatory elements (including MYB, MYC, as-1, ABRE, MBS, ERE, LTR, and TC-rich repeats) within these 10 *CrASR* and *CrLEA* promoter regions (Figure 5B). The categories and numbers of these elements suggested that the mechanisms regulating *CrASR* and *CrLEA* expression are involved in stress responses. As these genes had a high number of *cis*-regulatory elements related to ABA and drought stress responses, they were also selected for further functional analysis to obtain their detailed functions and regulatory mechanisms.

### 2.6. Expression Profiles of CrLEAs and CrASRs in Different Tissues and Plants Residing in Different Habitats

**Figure 6:**
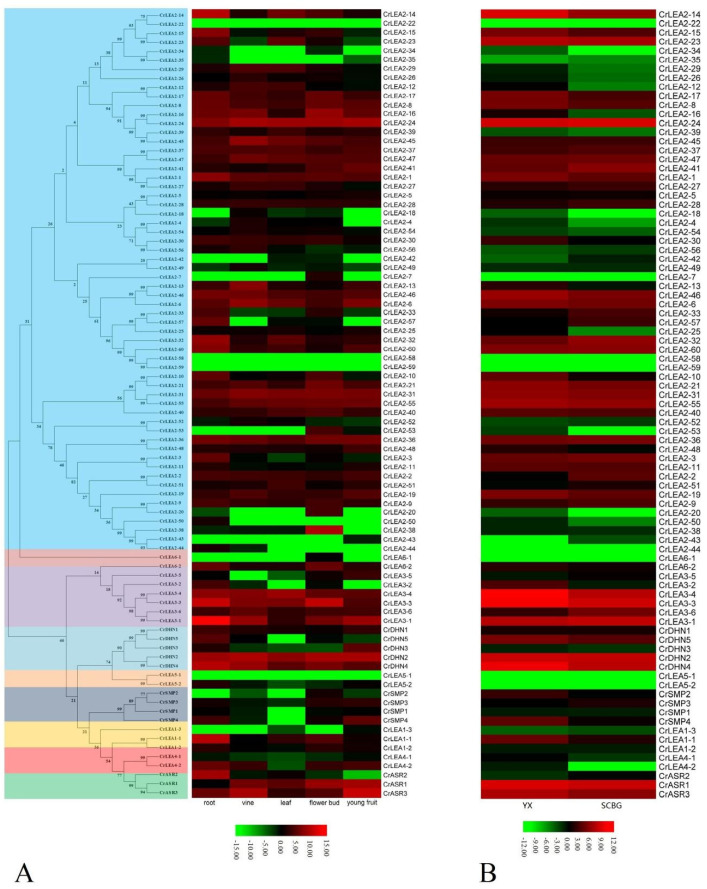
Heatmaps showing (**A**) the expression levels of the 87 *CrLEA/CrASR* genes in the root, vine, leaf, flower bud, and young fruit of *C. rosea* plant and (**B**) expression differences of the 87 *CrLEA/CrASR* genes in mature *C. rosea* leaves planting in South China Botanical Garden (SCBG) and in Yongxing Island (YX). On the far left is the phylogenetic tree showing the different subfamilies by different colored boxes.

To obtain the expression patterns of the *CrLEA* and *CrASR* family members in different tissues, we selected five tissue types, including the roots, vines, young leaves, flowering buds, and young fruits gathered from SCBG, for detailed RNA-Seq analysis. As shown in Figure 6A, there were only four gene members (*CrLEA2-22*, *CrLEA2-58*, *CrLEA2-59*, and *CrLEA5-1*) whose transcripts could not be detected in all five tissues, which indicated that these genes were probably not expressed or only showed very low transcriptional levels. Furthermore, in combination with the *CrLEA* gene duplication, we did not find obvious expressive similarity between the duplicated gene pairs (Figure 6A, Table 2), which indicated that these genes underwent genetic evolution mainly related to the regulation of gene expression patterns before their basic functions exhibited significant differentiation. Generally, the RNA-Seq analysis using different tissues showed that *CrLEA*s and *CrASR*s have tissue expression specificity, and some showed relatively higher expression levels in all tested tissues, while some were even undetectable (Figure 6A).

We further analyzed the transcriptional differences of the *CrLEA*s and *CrASR*s between the mature leaves collected from two habitats: SCBG and YX Island. As we can see from Figure 6B, except for the eight undetectable *CrLEA*s, including *CrLEA2-7*, *CrLEA2-22*, *CrLEA2-44*, *CrLEA2-58*, *CrLEA2-59*, *CrLEA5-1*, *CrLEA5-2*, and *CrLEA6-1*, a great amount of the other members (52 *CrLEA*s and *CrASR*s) showed higher expression in the YX sample than in the SCBG sample, except that 27 members were opposites, including *CrLEA1-3*, *CrLEA2-2*, *CrLEA2-3*, *CrLEA2-9*, *CrLEA2-24*, *CrLEA2-27*, *CrLEA2-28*, *CrLEA2-32*, *CrLEA2-33*, *CrLEA2-35*, *CrLEA2-36*, *CrLEA2-37*, *CrLEA2-41*, *CrLEA2-42*, *CrLEA2-43*, *CrLEA2-45*, *CrLEA2-47*, *CrLEA2-51*, *CrLEA2-52*, *CrLEA2-56*, *CrLEA2-57*, *CrLEA2-60*, *CrLEA3-1*, *CrLEA3-5*, *CrLEA3-6*, *CrSMP3*, and *CrASR2*. Given the extreme heat, drought, and salt/alkaline environment in YX Island, this suggested that their biological functions are closely related to the adaptation to coral reef habitats.

### 2.7. Expression Profiles of CrLEAs and CrASRs in Response to Different Stressors and the ABA Treatment

**Figure 7:**
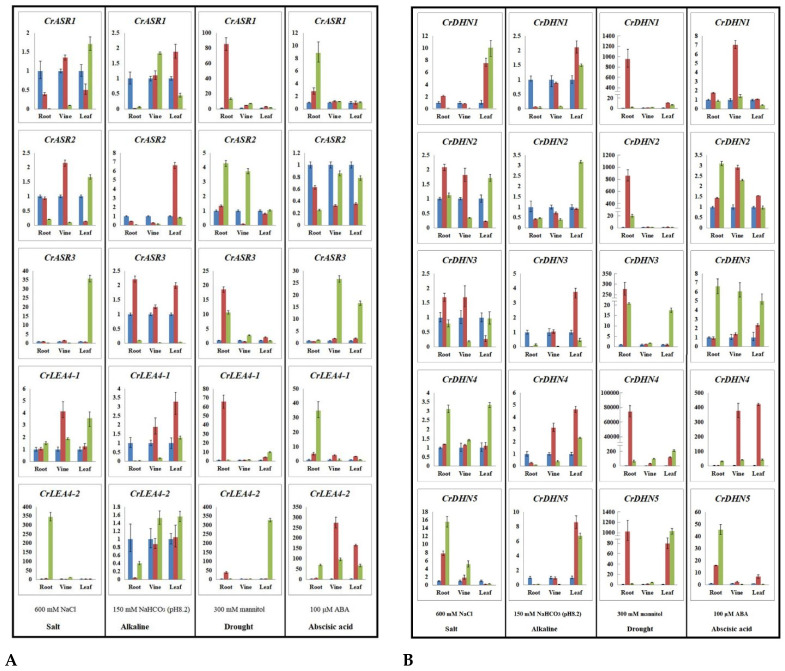
Quantitative RT-PCR detection of the expression levels of the 10 *CrLEA/CrASR* genes responding to different stresses (600 mM NaCl, 150 mM NaHCO_3_, 300 mM mannitol, or 100 mM ABA) in *C. rosea* seedling plants. (**A**) 3 *CrASR*s and 2 *CrLEA_4*s. (**B**) 5 *CrDHN*s. Relative expression values were calculated using the 2^−^^ΔCt^ method with housekeeping gene *CrEF-α* as reference gene.

Based on reports on the different *LEA* numbers involved in abiotic stresses [8,11,20,41,42,43,44,45], with reference to the RNA-Seq results and the promoter region analysis, we further identified 10 genes (including three *CrASR*s, five *CrDHN*s, and two *CrLEA_4*s) for expression analysis in different *C. rosea* tissues. Our aim was to examine the spatio-temporal patterns of these genes under various abiotic stress conditions and ABA treatment, from which we simulated the stressful conditions of the tropical coral reef in the laboratory, as far as possible. It has been proved that plant *ASR*s are closely related with water deficit stress and drought tolerance [11,41], and the *dehydrin*s play fundamental roles in plant response and adaptation to salinity and dehydration stresses [8,42]. *LEA_4* genes, also known as group 3 [8], have been proved being strongly associated with drought tolerance in basal and angiosperm resurrection plants via ABA signaling pathway [20]. The RNA-Seq analysis showed that nine of these 10 genes displayed higher expression levels in the YX leaf sample than in the SCBG leaf sample (except for *CrASR2*). The qRT-PCR analysis showed that all 10 genes were induced by different stresses or ABA in different organs to various degrees, while their expression levels were specific and variable (Figure 7). The mannitol stress (simulated drought) and ABA treatment caused significantly higher expression induction than the high salinity and alkaline treatments. Generally, alkaline stress showed a slighter effect than the other three treatments. *CrASR1* showed obviously elevated expression in the *C. rosea* roots under mannitol or ABA challenges, while *CrASR3* showed marked expression induction in the *C. rosea* leaves or vines in response to high salt or ABA treatments. *CrASR3* expression was also induced by mannitol in the roots (Figure 7A). Distinctively, *CrLEA4-1* and *CrLEA4-2* were significantly induced in all *C. rosea* tissues with the mannitol stress and ABA treatment, and the transcripts of *CrLEA4-2* showed a significant increase in the roots of *C. rosea* under high salt treatment (Figure 7A). Regarding the five dehydrin genes in *C. rosea*, all of the *CrDHN*s, without exception, had strong responses to mannitol and showed extremely significant expression induction in the root tissues of the *C. rosea* plants, while their expression patterns in response to the other three treatments (including high salinity, alkaline, and ABA) showed different degrees of induction or suppression (Figure 7B). Based on the above analysis, the results of the qRT-PCR are basically consistent with the results of the RNA-Seq analysis pertaining to the different habitats, and we propose that these genes may play important roles in the response of *C. rosea* to abiotic stress and adaptation to the extreme environment in the coral reef.

### 2.8. Abiotic Stress Tolerance of Yeast Heterologously Expressing CrLEAs and CrASRs

**Figure 8:**
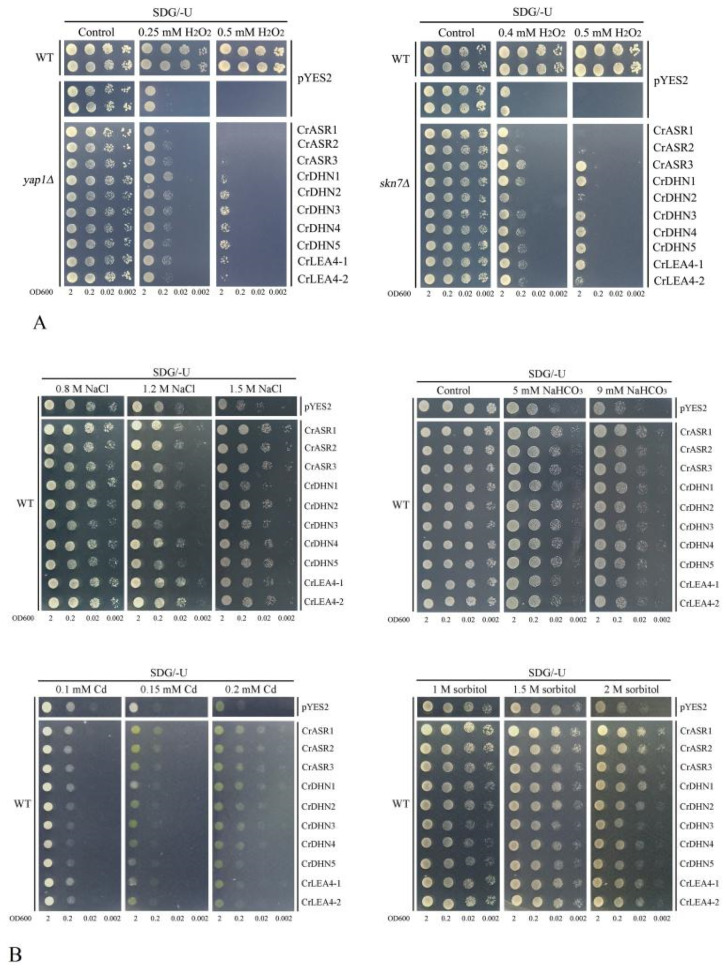
The tolerance confirmations of the 3 *CrASR*s, 5 *CrDHN*s, and 2 *CrLEA_4*s heteroexpression in yeast. (**A**) H_2_O_2_ oxidative stress tolerance confirmations in yeast. (**B**) Salt, alkaline, cadmium, and high osmotic tolerance confirmations in yeast. The stress factors with different concentrations were showed in figures.

Combined with our transcriptional analyses and promoter regions’ prediction, we could conclude preliminarily that these 10 *CrLEA*s and *CrASR*s (three *CrASR*s, five *CrDHN*s, and two *CrLEA_4*s) are closely related with the adaptation to abiotic stresses in *C. rosea*, especially to high salinity, alkaline, or water deficit, etc. To identify their potential roles in vivo, we performed a series of heterogeneous expression assays of the above 10 genes in a yeast system for functional stress tolerance investigation. For the antioxidation tolerance test, 10 *CrLEA*s and *CrASR*s were introduced into two H_2_O_2_-sensitive mutant strains *yap1Δ* and *skn7Δ*, with the corresponding wild-type *(*WT) yeast BY4741 and two mutant strains transformed with the empty vector pYES2 as controls. As we can see from Figure 8A, with the exception of CrASR1, the rest of the nine *C. rosea* genes all showed varying degrees of increased tolerance to H_2_O_2_. This indicated that these nine genes all possessed some antioxidation activities (Figure 8A). Due to the highly hydrophilic features and putative antioxidative abilities of CrLEAs and CrASRs, we also tested several other abiotic stress tolerances in the yeast WT strain (Figure 8B, upper). In Figure 8B with respect to salt stress, the majority of the above 10 genes showed elevated salinity tolerance in yeast than empty vector pYES2, while the effects varied. The expression of all 10 genes elevated the alkaline stress tolerance of the yeast. We assessed the cadmium (Cd) tolerance of WT yeast expressing *CrLEA*s and *CrASR*s, mainly based on their antioxidative abilities (Figure 8A) as well as the speculations of some reports that ASR or dehydrin proteins possess the ability to bind metals due to their possession of His-rich motifs [33,46,47]. Our results indicated that at least three *CrASR*s and four *CrDHN*s (including *CrDHN1*, *CrDHN2*, *CrDHN3*, and *CrDHN4*) all presented a certain degree of enhanced Cd tolerance in yeast (Figure 8B, lower). Furthermore, most of the 10 *CrLEA*s and *CrASR*s could increase the high osmotic stress tolerance of yeast when expressed in the WT strain (Figure 8B, lower). Although these yeast stress tolerance results are preliminary, and a few members even showed no significant effects against some stress challenges, there is still need for further functional identification in plants using transgenic systems.

### 2.9. Analysis of the Transcriptional Activation Activity of CrASRs

**Figure 9:**
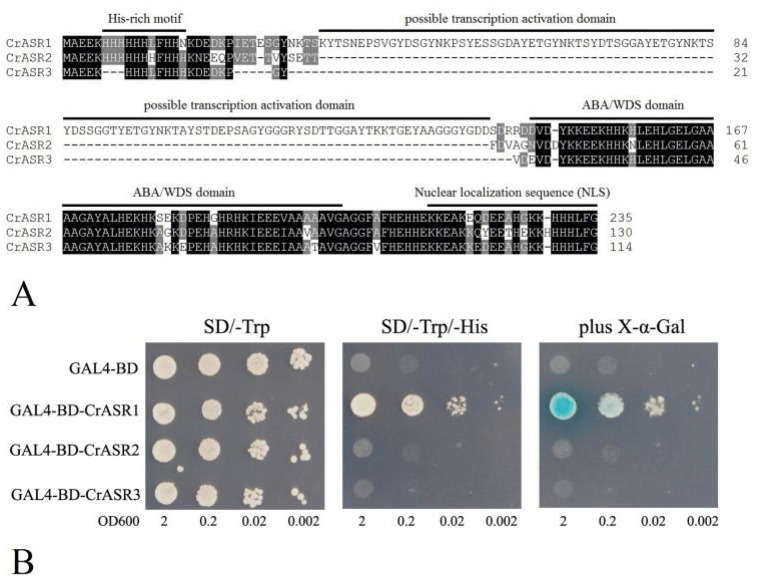
CrASRs sequence alignment and GAL4 DNA binding domain-CrASRs fusion analyses for transactivation activity in yeast. (**A**) Sequence alignment of CrASR1, CrASR2, and CrASR3. The N-terminal His-rich motif, possible transcription activation domain, ABA/WDS domain, and *C*-terminal nuclear localization sequence were marked with black bold lines. (**B**) The GAL4 DNA binding domain was fused with 3 CrASRs and transformed into the yeast strain AH109 containing the *His3* and *LacZ* reporter genes. Analysis of β-galactosidase activity of the relative yeast strains on plates. The yeast culture (OD600 to 2) was serially diluted to OD600 values of 0.2, 0.02 and 0.002, and then the 2-μL yeast liquid was spotted onto SD plates and cultured for 2 days at 30 °C.

Plant-specific ASRs are typically small gene families comprising several members, and some ASRs have been confirmed to act as transcription factors, with their N-terminals possessing transcription activation ability [48], although in some ASR members, this domain is obviously absent. We first aligned the sequences of three CrASRs, which indicated that only CrASR1 had the possible *N*-terminal transcription activation domain and could act as a transcription factor (Figure 9A). To further assess our speculation, the complete coding regions of all three CrASR cDNAs were ligated in-frame with the GAL4 DNA binding domain of the pGBKT7 vector and transformed in yeast in this study, with the empty vector GAL4-BD (pGBKT7-BD) as the negative control. The yeast growth on the SD medium lacking tryptophan (SD/-Trp) was normal and even, while the growth on the SD medium lacking tryptophan and histidine (SD/-Trp/-His) was inhibited. Only the yeast cells containing GAL4-BD-CrASR1 exhibited normal growth, and the LacZ staining assay of β-galactosidase activity was positive (Figure 9B), which indicated that CrASR1 showed transcription activation activity and might be a transcription factor. 

## 3. Discussion

Due to the significant roles of LEA and ASR proteins in water deficit stress responses and ROS scavenging abilities, they are believed to act in multiple developmental processes and in response to various stresses, as indicated in a number of previous reports [1,2,3,9]. As *C. rosea* is a halophyte with the typical features of high salt/alkaline and drought tolerance, it is necessary to systematically investigate the potential role of LEA/ASRs, especially given the lack of studies on *LEA*/*ASR* genes in legumes (Fabaceae). As *C. rosea* generally occurs in tropical and subtropical coastal regions, water shortages are one of the main impacts of its environmental surroundings. It is believed that *C. rosea* has developed a series of sophisticated mechanisms at multiple levels to cope with stress, including morphological, physiological, and genetic changes and adaptations, ultimately regulating the expression of stress-responsive genes through complex networks. The downstream responsive genes include some water stress-related genes, such as *LEA*s or *ASR*s, *aquaporin*s, or ROS-producing and scavenging genes.

*LEA* protein genes, which were first identified on account of their marked transcript accumulation in embryos for coping with rapid dehydration during seed maturation [15], were later found to be induced in vegetative plant tissues under environmental stress conditions. *ASR* genes, which encode hydrophilic proteins or transcription factors, were first identified from a tomato cDNA library and participated in response to water-stressed conditions both in stressed leaves and in ripe fruits [41]. LEA proteins constitute a large multigene family that is closely related to the response to abiotic stresses in multiple plant species and that protects cells against water deficit caused by drought and other stresses. While *LEA*s are not just confined to Plantae, ASRs are exclusive to the plant kingdom (but are absent in *Brassicaceae*). Both *LEA*s and *ASR*s are known to participate in multiple developmental processes and in response to various stresses, mainly water shortage challenges. In the present study, we conducted whole-genome scanning in *C. rosea*, and a total of 84 *CrLEAs* and three *CrASRs* were identified (Table 1). The number of *LEA* or *ASR* genes in *C. rosea* was compared with other plant species [11,20], and the number of different subfamilies of *LEA*/*ASR* genes from other legume species was summarized (Table S5). We found that the numbers of *LEA*/*ASR* genes in all of these typical diploid legume species were similar, while the soybean (*Glycine max*) genome contained obviously more (143), probably due to a whole-genome duplication event in the distant past. Among them, the LEA_2 subfamily (PF03168) possessed the largest member number and the greatest variability in all plant species (Figure 1, Table S5), which might imply a diversified functionality of this atypical LEA subfamily [20].

All of these CrLEA/CrASR proteins possess common characteristics, including small molecular weights, an abundance of hydrophilic amino acids, and coding genes that are intronless or contain few introns (Table 1, Figure 2). Most LEA proteins are termed hydrophilins mainly due to their unifying and outstanding feature of high hydrophilicity and a high content of Gly and small amino acids such as Ala and Ser [8]. Previous research indicated that some LEA proteins present a high degree of unordered structure in solution and are considered IDPs [23]. The high hydrophilicity and intrinsically disordered features of LEAs facilitate their protective functions by promoting associations with membrane surfaces or protein partners for protection, and by sequestering H_2_O, ROS, ions, or other small molecules for alleviating damage or toxicity [23]. Except for some LEA_2 members, most of CrLEA/CrASR proteins showed negative GRAVY scores (<0) (Table 1), indicating that these proteins have strong hydrophilicity and could offer pivotal protective roles under rapid and severe dehydration in plants. Subcellular localization prediction revealed that the CrLEA/CrASR proteins were present in all subcellular compartments, which is consistent with the functional prediction that LEA/ASR proteins in principal groups are ubiquitous within cells and are required in all cellular compartments responding to abiotic stress [49]. We also predicted the 3D structures and calculated the disordered amino acid contents of all CrLEA/CrASR proteins, and the results indicated that except CrLEA_2 and CrSMP subfamilies, the other proteins showed obviously disordered structures (Figures S1 and S2). Furthermore, previous studies have indicated that plant stress-responsive genes without or with few introns could reduce the time required from transcription to translation, therefore providing good adaptation abilities for plants responding to changes in environments and habitats [46]. Combined with the CrLEA/CrASR gene structure and protein motif, we can infer that CrLEA/CrASR proteins are evolutionarily conserved, and their functions have group specificity.

Gene duplication plays a crucial role in the expansion of gene families and is a major way in which genomes can be reshaped, therefore promoting organismal adaptive evolution to the environment [47]. Based on the evolutionary characterization of *CrLEA*s and *CrASR*s, we found that most segmental and tandem duplications occurred in the *CrLEA_2* subfamily, and similar characteristics have also been reported for *LEA* genes in other plant species [20], which suggests that the *LEA_2* subfamily contains the most diverse *LEA* members in plants. LEA_2 is considered as an atypical LEA protein because it possesses more hydrophobic amino acids and a more defined secondary structure compared with other LEA subfamily members [20]. Additionally, also we found that this subfamily contained the only tandem duplicate cluster in *C. rosea* (Table 2), indicating that tandem duplications have contributed significantly to the expansion and diversification of the large LEA_2 family in *C. rosea*. The other segmental duplications occurring in CrLEA_1, CrLEA_3, CrLEA_4, and CrLEA_5 indicated that these LEA genes might participate in the adaptive evolution of *C. rosea* to water shortage stresses by increasing their gene numbers.

As *LEA*s have been confirmed to endow plants with a variety of abiotic and biological stress tolerances, LEA families often have multiple copies of LEA genes in various plant species for that very reason. Correspondingly, even within the same LEA subfamily, our transcriptome data showed that the expression of different members was specific (Figure 6), which is probably derived from the influence of the promoter regions. Our statistical analysis results of the promotors of *CrLEA*s/*CrASR*s showed that most of the promoter regions contained *cis*-regulatory elements, such as ABRE, MYC, MYB, MBS, TC-rich repeats, and several hormone-related responsive elements, suggesting that these genes could be regulated or affected by different stresses (Figure 5A). We also analyzed 10 promoter regions (including three *CrASR*s, five *CrDHN*s, and two *CrLEA_4*s) in detail. We found that the promoter regions showed more variability than the gene coding region, which corresponds with their gene-specific expression pattern (Figure 5B and Figure 6). The contribution of single *CrLEA* or *CrASR* genes to the stress tolerance and environmental adaptation of *C. rosea* needs to be further explored. 

An investigation of the natural habitat of *C. rosea* indicates that this species possesses significant growth potential and is an adaptable pioneer species that can be used for island greening, sand fixation, and the ecological restoration of coral islands and coastal zones in tropical or subtropical regions [40]. We firstly examined the expression of the *CrLEA* and *CrASR* genes in the different tissues and developmental periods using RNA-Seq. The expression profiles revealed spatial variations in the expression of *CrLEA*s or *CrASR*s in different organs (Figure 6A). Our further habitat-specific RNA-Seq data indicated that most of the *CrLEA*/*CrASR* genes had greater expression levels in coastal *C. rosea* (YX) than in inland *C. rosea* (SCBG), while some were quite the opposite (Figure 6B). Our results suggested that the differential expression of *CrLEA*/*CrASR* genes might be an adaptive mechanism for dealing with intracellular and extracellular water-deficit signals in *C. rosea* plants, which is associated with different water loss strategies in different habitats and, in the longer term, may also function in the biological adaptability to changes in environmental factors and different habitats.

We also used qRT-PCR to investigate the expression patterns of 10 candidate genes in *C. rosea* plants under salt, alkaline, high osmotic treatment, and ABA stresses. Detailed expression profiles of these 10 *CrLEA*/*CrASR* genes revealed that most were significantly up-regulated after the high osmotic stress (NaCl or mannitol) or ABA treatment, while the alkaline stress showed the least impact on the expression changes of candidate genes (Figure 7). This result is consistent with several previous reports of *LEA* genes in other plant species, in which their expression was also greatly affected by abiotic stress challenges and hormone treatments [19,48,50]. The relevant roles of *ASR*s, *DHN*s, or the *LEA_4*s subfamily genes during plant stress tolerance have been reported in earlier studies using various techniques [11,20,42]. Here both the RNA-Seq and the qRT-PCR results indicated that these genes might be vital factors influencing the adaptability of *C. rosea* plants to salt/alkaline and drought stresses in tropical or subtropical coastal regions.

The accumulation of LEA proteins in cells is crucial for the response of organisms to different abiotic stresses. Numerous reports have demonstrated that overexpressing *LEA* genes in different organisms resulted in improved salinity and dehydration tolerance. For example, *Ipomoea pes-caprae* is a perennial herbaceous vine plant distributed mainly on sandy beaches or in sunny positions on the roadside in tropical and sub-tropical regions. It exhibits excellent salt tolerance and drought resistance, and the induction of *IpLEA*s in yeast obviously improved the salt and oxidative stress tolerance of yeast clones [51]. Prior studies revealed that some *LEA* genes from *Dendrobium officinale* were able to enhance the cellular tolerance to high temperature and salt stresses of *E. coli* cells [49]. For ASR genes, our previous research also demonstrated that an *I. pes-caprae* ASR gene, *IpASR*, could improve salinity and drought tolerance in transgenic *E. coli* and *Arabidopsis* [45]. In summary, *LEA* and *ASR* genes are pertinent research subjects in plant stress physiology and are mainly involved in drought or salinity tolerance in plants. Here, we identified and characterized 10 *CrLEA*/*CrASR* genes in *C. rosea* for the first time and performed functional verification in yeast to investigate bioactivities for abiotic stress resistance. Based on our results, nine of the candidate genes increased the yeast cell resistance to H_2_O_2_, showing individual differences (Figure 8A). The majority of the 10 tested genes elevated the high salinity, alkaline, and high osmotic stress tolerance of yeast compared with the empty vector control, while some even showed heavy metal Cd tolerance, but not significantly so (Figure 8B). Our results demonstrated that these *CrLEA*/*CrASR* genes may play protective roles in cells under water-deficit conditions caused by high salt/alkaline and high osmotic stresses, and they also could improve cell survival via their antioxidant capacities or metal chelating abilities.

Although CrASR1 did not present antioxidant properties in yeast, it is a highly hydrophilic protein with a relatively low GRAVY value score (Table 1). Unlike CrASR2 or CrASR3, CrASR1 has a remarkable property in that it contains a Gly-rich domain in its N-terminus (Figure 9A). According to our previous reports [35,45], it is uncertain whether this type of ASR protein could activate transcription and act as a transcription factor, while our result on the transcriptional activation assay in yeast indicated that CrASR1 did exhibit transcription factor activity, yet CrASR2 and CrASR3 had exactly the opposite effect (Figure 9B). This indicated that CrASR1 could act as a transcriptional regulator together with chaperone-like proteins or hydrophilins, like other LEA/ASR proteins [11,34]. Previous studies have proved that tomato ASR1 is a drought stress-responsive transcription factor, and its consensus ASR1-binding motif was enriched in some specific tomato genomic loci, possibly containing several aquaporin genes [12,52]. A recent study on CaASR1 in another Solanaceae species, *Capsicum annuum*, also provided evidence that CaASR1 could interact with the transcription factor CabZIP63 and is a positive regulator of the defense response of pepper to the pathogen *R. solanacearum*, probably by acting as a transcription activator [53]. Our results strongly suggest that CrASR1 showed quite different features from the other two CrASRs or CrLEAs (Figure 8 and Figure 9), and this protein might fulfill an important function in salt/drought tolerance and regulate the environmental adaptation of *C. rosea* to tropical coastal regions, although more research is needed for further clarification.

## 4. Materials and Methods 

### 4.1. Plant Materials and Stress Treatments

*C. rosea* plants growing in Yongxing Island (YX, 16°83′93′′ N, 112°34′00′′ E) and South China Botanical Garden (SCBG, 23°18′76′′ N, 113°37′02′′ E) were used in this study. To analyze the tissue-specific transcriptional patterns of the *CrLEA* and *CrASR* genes, the roots, stems, leaves, flowers, and fruits were sampled from *C. rosea* plants grown in SCBG. To investigate the involvement of the *CrLEA* and *CrASR* genes in the adaptation to a high-temperature island climate, coastal saline environment, and seasonal drought environment, adult plant leaves were also gathered from *C. rosea* growing on both YX Island and SCBG. 

The responses of the *CrLEA* and *CrASR* genes to different stresses and hormone treatments were investigated. Seedlings of *C. rosea* germinated from seeds in a soil/vermiculite mixture for 30 d were subjected to treatments. In brief, for high salinity stress, the *C. rosea* seedlings were removed from the pots and carefully washed with distilled water to remove soil from the roots, following which they were transferred into 600 mM NaCl solution; for salt-alkaline stress, the cleaned *C. rosea* seedlings were soaked in a 150 mM NaHCO_3_ (pH 8.2) solution; for the drought treatment, the seedlings were soaked in a 300 mM mannitol solution; and for abscisic acid (ABA) treatment, a freshly prepared working solution of 100 μM exogenous ABA was sprayed onto the leaves of the *C. rosea* seedlings. The second and/or third mature leaves from the shoot apexes were collected at 0, 2, and 24 h during the stress treatments, and the starting point (0 h) was used as the control. All samples were immediately frozen in liquid nitrogen and stored at −80 °C for subsequent gene expression analysis. Three independent biological replicates were conducted.

### 4.2. Identification of LEA/ASR Genes in the C. rosea Genome and Phylogenetic Analysis of CrLEA/CrASR Superfamily Proteins

The putative *CrLEA* and *CrASR* gene sequences were collected from the genome database of *C. rosea*. All of the *C. rosea* proteins were first identified using DIAMOND [54] and InterProscan (https://www.ebi.ac.uk/interpro/search/sequence/, accessed on 1 March 2021) to assess the conserved domains and motifs (e < 1 × 10^−5^), following which they were annotated using the Pfam database (http://pfam.xfam.org/, accessed on 1 March 2021). The Pfam ID PF03760 (LEA_1), PF03168 (LEA_2), PF03242 (LEA_3), PF02987 (LEA_4), PF00477 (LEA_5), PF10714 (LEA_6), PF00257 (dehydrin, DHN), PF04927 (SMP), and PF02496 (ASR) were used to search the *CrLEA*s and *CrASR*s, and the putative sequences of CrLEA and CrASR proteins were identified and submitted to SMART (http://smart.embl-heidelberg.de/, accessed on 1 March 2021) and the NCBI Conserved Domain Database (https://www.ncbi.nlm.nih.gov/Structure/cdd/wrpsb.cgi, accessed on 1 March 2021) to confirm the presence of the LEA or ASR domains. Finally, the selected *CrLEA*s and *CrASR*s were named based on their sequence homology with other known plant LEAs or ASRs and the *C. rosea* genome annotations. An unrooted neighbor-joining phylogenetic tree was created based on multiple protein sequence alignments of all identified CrLEAs and CrASRs from *C. rosea* using Clustal X 2.0 and MEGA 6 with 1000 bootstrap replicates. The obtained LEA and ASR nucleotide and protein sequences from *C. rosea* are listed in Table S1. The gene structure analysis for *CrLEA*s and *CrASR*s was displayed with GSDS (http://gsds.cbi.pku.edu.cn/, accessed on 1 March 2021). 

### 4.3. Analysis of Protein-Conserved Motifs and Biochemical Features of CrLEAs/ASRs

To investigate the characteristics of the CrLEA and CrASR proteins, the molecular weight (MW), theoretical isoelectric point (pI), and grand average of hydropathy (GRAVY) were predicted using the ProtParam tool (http://web.expasy.org/protparam/, accessed on 1 March 2021). Furthermore, the subcellular localization predictions for these CsLEA proteins were carried out using the WoLF_PSORT tool (http://www.genscript.com/wolf-psort.html, accessed on 1 March 2021). The contents of disordered amino acids (%) in each CrLEA/ASR were calculated according to the online program PrDOS (Protein DisOrder prediction System, http://prdos.hgc.jp/cgi-bin/top.cgi, accessed on 22 April 2021). The deduced amino acid sequences of the CrLEAs and CrASRs were analyzed by the Multiple Expectation Maximization for Motif elicitation (MEME) tool (http://meme-suite.org/index.html, accessed on 1 March 2021) to identify conserved domains and motifs of each subgroup of these proteins. The selection of the maximum number of motifs was set to 3, with a minimum width of 11 and a maximum width of 50 amino acids, and an e-value < 1 × 10^−^^8^. The Phyre2 server (http://www.sbg.bio.ic.ac.uk/phyre2/html/page.cgi?id=index, accessed on 1 March 2021) was used for homology modelling of the three-dimensional (3D) structure of all CrLEA and CrASR proteins.

### 4.4. Gene Duplication and Collinearity Analysis of CrLEAs

The *CrLEA* and *CrASR* genes were mapped onto *C. rosea* chromosomes according to the positional information of these genes in the *C. rosea* genome database and were displayed using MapInspect software (http://mapinspect.apponic.com/, accessed on 1 March 2021). Gene segmental duplications were assessed using MCScanX software (http://chibba.pgml.uga.edu/mcscan2/, accessed on 1 March 2021), and tandem duplications were identified manually. The number of synonymous substitutions per synonymous site (Ka), the number of non-synonymous substitutions per non-synonymous site (Ks), and the *p*-value from Fisher’s exact test of neutrality were calculated using the Nei-Gojobori model with 1,000 bootstrap replicates [55]. A Ka/Ks ratio < 1 indicates purifying selection, a Ka/Ks ratio = 1 indicates neutral selection, and a Ka/Ks ratio > 1 indicates positive selection. The gene segmental duplications of the *CrLEA*s were visualized using the online shinyCircos software (https://venyao.xyz/shinyCircos/, accessed on 1 March 2021).

### 4.5. Promoter Sequence Profiling of CrLEAs/ASRs

The promoter regions (1000 bp upstream from the translation start site) of all C*rLEA* and *CrASR* genes were retrieved from the genome database of *C. rosea* for further analysis of the *cis*-regulatory elements and motifs by querying them through the PlantCARE database (http://bioinformatics.psb.ugent.be/webtools/plantcare/html/, accessed on 1 March 2021). The stress- and hormone-related *cis*-regulatory elements included light response elements, gibberellin-responsive elements, methyl jasmonate (MeJA)-responsive elements, auxin response elements, salicylic acid responsiveness elements, ABA response elements (ABRE), ethylene response elements (ERE), MYC transcription factor binding elements (MYC), MYB transcription factor binding elements (MYB and MBS), TC-rich repeats, LTR, and as-1. These elements are believed to be involved in plant responses to dehydration, low temperature, salt stress, and other abiotic stresses (http://bioinformatics.psb.ugent.be/webtools/plantcare/html/, accessed on 1 March 2021).

### 4.6. RNA-Seq of Different C. rosea Tissues

The *C. rosea* RNA-Seq datasets were constructed using Illumina HiSeq X sequencing technology. In brief, seven different tissues from *C. rosea* plants (root, vine, young leaf, flower bud, and young silique samples collected from *C. rosea* plants growing in SCBG; mature leaf samples from *C. rosea* growing in SCBG and on YX Island) were examined using FastQC (http://www.bioinformatics.babraham.ac.uk/projects/fastqc/, accessed on 1 March 2021) based on the primary 40 Gb clean reads and were mapped to the *C. rosea* reference genome using Tophat v2.0.10 (http://tophat.cbcb.umd.edu/, accessed on 1 March 2021). The heatmaps showing the *CrLEA* and *CrASR* gene expression profiles were generated using TBtools [56], principally adopting the fragments per kilobase of transcript per million mapped reads (FPKM) values, and the expression levels (log2 of FPKM values) of these genes were visualized.

### 4.7. Expression Analysis by Quantitative Reverse Transcription (qRT) PCR of CrLEAs/ASRs under Different Stress Treatments

The transcript abundance of several *CrLEA* and *CrASR* genes was further investigated using qRT-PCR. Total RNA was extracted from the *C. rosea* seedling roots, vines, and leaves under the stress/ABA treatment and unstressed treatment using an RNA plant extraction kit (Magen, Biotech, Guangzhou, China) according to the manufacturer’s instructions, and approximately 1 μg of purified total RNA was reverse transcribed into cDNA in a 20 μL reaction volume using AMV reverse transcriptase (TransGen Biotech, Beijing, China) according to the supplier’s instructions. In brief, total RNA was extracted from *C. rosea* seedling tissues under the stress/ABA treatments and reverse transcribed into cDNA. Quantitative RT-PCR was conducted using the LightCycler480 system (Roche, Basel, Switzerland) and TransStart Tip Green qPCR SuperMix (TransGen). All of the gene expression data obtained via qRT-PCR were normalized to the expression of *CrEF-α* (Table S1). The primers used for qRT-PCR (CrEF-αRTF/CrEF-αRTR for the reference gene and other *CrLEA*- or *CrASR*-specific primer pairs) are listed in Supplementary Materials Table S1.

### 4.8. Cloning of CrLEA/ASR cDNAs and Heterologous Expression in Yeast

To clone the CrLEA/ASR cDNAs, total RNA isolation and cDNA synthesis was performed as described above. The full-length CrLEA/ASR cDNA was PCR-amplified from *C. rosea* leaf cDNA using the gene specific primers listed in Supplementary Materials Table S1. The construction of the yeast expression vector pYES2-CrLEAs/ASRs were constructed with in-fusion technique (BD In-Fusion PCR cloning Kit, Takara Bio USA, Mountain View, CA, USA) according to the manufacturer’s instruction. In short, the PCR fragments were inserted into the *Bam*HI and *Eco*RI sites of pYES2 and after sequencing confirmation, these recombinant expression vectors were transformed into different yeast strains to verify the stress tolerance functions. The wild-type (WT) yeast (*Saccharomyces cerevisiae*) strain BY4741 (Y00000, MATa; *ura3Δ0; leu2Δ0; his3Δ1; met15Δ0*) and the two H_2_O_2_-sensitive mutant strains *yap1Δ* (Y00569, BY4741; MATa; *ura3Δ0; leu2Δ0; his3Δ1; met15Δ0; YML007w::kanMX4*) and *skn7Δ* (Y02900, BY4741; *MATa; ura3Δ0; leu2Δ0; his3Δ1; met15Δ0; YHR206w::kanMX4*) were obtained from Euroscarf (Frankfurt, Germany). The various yeast strains were transformed using the LiOAc/PEG technique, and uracil complementation was used for selection. A synthetic defined (SD) yeast medium without uracil (SD-Ura) containing 2% (*w*/*v*) galactose (Gal) was used as the yeast induction medium (SDG-Ura) during the salt/alkaline tolerance and oxidative stress experiments on solid medium for the localization observations. The concentrations of the different stress factors are indicated in the figure legends.

### 4.9. Transcriptional Activity Analysis of CrASRs in Yeast

The open reading frames (ORFs) of three CrASRs were cloned into the vector pGBKT7 (Clontech, Mountain View, CA, USA) for transcription activation analysis with the yeast two-hybrid assay according to the manufacturer’s introduction (Clontech, CA, USA). In brief, the full CDSs of CrASRs were amplified from *C. rosea* leaves cDNA using the corresponding primers as listed in Supplementary Materials Table S1. Then all PCR products were inserted into GAL4-DBD vector pGBKT7 at *Eco*RI site and sequenced. These constructs along with the negative control pGBKT7 (containing the binding domain, BD) were transformed into yeast strain AH109 using the LiOAc/PEG method. The yeast clones were cultured in liquid SD-Trp medium to an OD600 value of 2, after which they were diluted using a gradient dilution (1:10, 1:100, and 1:1000). Two-microliter yeast cultures were spotted onto the corresponding synthetically defined (SD/-Trp and SD/-Trp/-Leu) medium plates for 2 days at 30 °C. Yeast transformation and determination of blue/white colonies were conducted according to the instructions of the manufacturer (Clontech), and X-α-Gal was used as a substrate for the reporter gene MEL1. The primers used in construction of pGBKT7 vectors for CrASRs transactivation activity assay in yeast are listed in Supplementary Materials Table S1.

### 4.10. Statistical Analysis

All the experiments in this study were repeated three times independently, with the results shown as the mean ± SD (*n* ≥ 3). Pairwise differences between means were analyzed using Student’s *t*-tests in Excel 2010 (Microsoft Corporation, Albuquerque, NM, USA). 

## 5. Conclusions

In summary, this study is the first to systematically summarize members of the *LEA* and *ASR* gene family in a leguminous halophyte, *C. rosea*. In total, 84 *CrLEA* genes and three *CrASR* genes were identified in the *C. rosea* genome and were classified into nine subgroups. Chromosomal mapping and synteny analysis revealed that these members are distributed in all 11 chromosomes of *C. rosea*, with several gene tandem and segmental duplication patterns for LEA gene expansion in the *C. rosea* genome. The CrLEA/CrASR members within the same subfamily are highly conserved in both gene structures and protein motifs. The *CrLEA/CrASR* superfamily was involved in the adaptation of *C. rosea* to different habitats, and 10 of the *CrLEA*/*CrASR* members showed obvious differences in gene expression in response to salt/alkaline stress, high osmotic stress, or ABA treatment. This systematic study provides new information on the LEA/ASR superfamily in *C. rosea* and further expands our understanding of the association of *CrLEA*/*CrASR* genes with natural ecological adaptability and abiotic stress responses in *C. rosea*. Our findings should also inform the genetic improvement of other legume plants or crops and provide candidate stress-resistance genes for future research.

## Figures and Tables

**Table 1:** Description of CrLEAs identified from *Canavalia rosea* genome.

Name	Gene Locus	Length (aa)	Mw (kD)	PI	Instability Index (II)	Aliphatic Index (AI)	GRAVY	Content of Disordered aa (%)	Subcellular Localization *
CrLEA1-1	03T007960	174	17.92	9.13	11.32	35.00	−1.060	83.91	mito: 10, nucl: 4
CrLEA1-2	04T011772	192	20.00	5.38	45.87	49.53	−0.725	83.33	nucl: 7, cyto: 3
CrLEA1-3	05T014731	136	14.98	9.03	54.57	60.44	−0.992	94.12	nucl: 9, mito: 3
CrLEA2-1	01T000091	294	32.86	10.00	57.75	79.18	−0.266	25.51	chlo: 3, cyto: 3, E.R.: 3
CrLEA2-2	01T000357	210	22.61	9.47	39.79	88.24	0.250	10.48	chlo: 5, mito: 5
CrLEA2-3	01T000402	221	24.59	9.21	37.80	103.57	0.078	18.55	chlo: 5, cyto: 5
CrLEA2-4	01T001888	207	22.93	9.32	34.25	95.12	0.038	12.08	plas: 8, cyto: 4
CrLEA2-5	01T003219	230	25.26	9.21	34.53	111.39	0.283	16.96	plas: 4.5, vacu: 4, nucl_plas: 3
CrLEA2-6	02T004727	259	28.57	9.88	35.46	82.39	−0.232	18.53	chlo: 10, mito: 3
CrLEA2-7	02T004857	262	29.07	9.80	50.89	84.35	−0.331	22.14	cyto: 7
CrLEA2-8	02T005159	207	23.10	9.78	33.03	100.24	−0.048	13.04	chlo: 4, plas: 4
CrLEA2-9	02T006010	192	20.74	9.28	13.81	106.98	0.272	6.25	vacu: 4, E.R.: 3
CrLEA2-10	02T006879	190	21.16	6.83	28.11	96.42	−0.112	6.32	nucl: 8, chlo: 3
CrLEA2-11	02T006881	248	27.67	10.05	33.42	94.68	−0.042	14.52	cyto: 8
CrLEA2-12	02T007016	233	26.24	9.41	48.70	97.38	−0.033	21.46	cyto: 8
CrLEA2-13	02T007186	250	27.17	9.34	36.97	90.88	−0.004	16.4	chlo: 8, mito: 4
CrLEA2-14	03T008517	227	25.78	9.40	36.41	89.30	−0.131	7.49	golg: 4.5, golg_plas: 4.5, plas: 3.5, E.R.: 3
CrLEA2-15	03T008518	228	26.55	9.48	37.63	81.18	−0.291	10.09	chlo: 10, pero: 3
CrLEA2-16	03T008519	209	23.77	9.71	36.98	97.32	0.018	7.18	cyto: 11
CrLEA2-17	03T009222	197	21.48	9.76	31.71	110.71	0.356	7.61	chlo: 6, E.R.: 4
CrLEA2-18	03T009270	235	26.49	8.98	33.86	103.19	0.329	6.81	cyto: 10
CrLEA2-19	03T010271	222	24.12	9.64	24.57	121.08	0.232	15.77	nucl: 6
CrLEA2-20	03T010272	195	21.68	6.95	23.09	100.41	0.074	6.67	E.R.: 4, mito: 3, extr: 3
CrLEA2-21	03T010452	176	19.07	5.86	26.80	113.47	0.181	6.25	plas: 6, vacu: 5
CrLEA2-22	04T011627	200	23.20	9.40	35.70	104.65	0.115	0.5	extr: 3, vacu: 3, E.R.: 3
CrLEA2-23	04T011636	228	26.46	9.57	34.97	81.67	−0.233	11.84	chlo: 8
CrLEA2-24	04T011638	210	23.82	9.63	47.73	101.52	0.099	6.19	cyto: 7
CrLEA2-25	04T012994	249	27.84	9.90	38.97	101.37	−0.087	20.48	cyto: 7, nucl: 3, E.R.: 3
CrLEA2-26	04T013772	225	25.61	9.42	41.04	76.62	−0.399	11.11	chlo: 14
CrLEA2-27	05T014592	322	35.41	10.11	58.09	71.71	−0.333	31.68	chlo: 3, vacu: 3
CrLEA2-28	05T016584	272	29.42	9.60	42.56	84.19	−0.214	32.35	nucl: 5, chlo: 4, plas: 3
CrLEA2-29	05T016770	202	23.15	9.75	44.59	107.13	0.049	11.39	chlo: 5, mito: 3
CrLEA2-30	06T017171	233	26.69	10.13	39.27	102.45	−0.009	19.31	cyto: 5, plas: 4
CrLEA2-31	06T017436	320	35.70	4.80	25.33	91.97	−0.437	16.25	cyto: 9
CrLEA2-32	06T017480	273	30.26	9.43	50.16	89.56	−0.200	19.41	cyto: 4, chlo: 3, plas: 3
CrLEA2-33	06T018775	257	28.95	10.17	49.92	99.69	−0.162	23.35	nucl: 6, cyto: 5
CrLEA2-34	07T020998	211	24.12	9.10	30.10	95.59	0.045	6.64	extr: 5, chlo: 3, E.R.: 3
CrLEA2-35	07T020999	499	57.65	9.51	31.38	89.80	−0.086	2.2	plas: 7.5, golg_plas: 6, golg: 3.5
CrLEA2-36	07T021162	247	27.83	9.15	54.28	97.41	−0.057	13.77	plas: 4, golg: 3
CrLEA2-37	07T021185	308	34.10	9.58	62.03	62.27	−0.514	25.32	chlo: 3, plas: 3, E.R.: 3
CrLEA2-38	08T022453	223	24.65	9.50	27.00	97.89	0.155	15.25	chlo: 5, cyto: 3
CrLEA2-39	08T022523	219	24.64	9.74	53.37	106.80	0.185	5.94	E.R.: 5
CrLEA2-40	08T022549	252	27.66	6.83	38.09	110.32	0.311	5.56	cyto: 7
CrLEA2-41	08T023188	317	35.77	9.67	57.68	82.93	−0.391	36.59	chlo: 4, nucl: 3, mito: 3
CrLEA2-42	09T024657	244	27.90	7.50	40.73	100.20	0.075	18.03	chlo: 6
CrLEA2-43	09T024732	184	19.82	9.68	21.51	121.25	0.497	4.89	vacu: 5, extr: 3
CrLEA2-44	09T024733	186	20.56	8.99	14.50	107.37	0.354	3.76	extr: 4, vacu: 4
CrLEA2-45	09T024798	220	24.57	8.97	38.64	104.95	0.119	6.82	chlo: 4
CrLEA2-46	09T025174	255	27.99	10.30	36.23	87.22	−0.138	18.04	chlo: 8, mito: 5
CrLEA2-47	09T025255	308	34.13	9.93	58.73	72.37	−0.347	29.55	chlo: 4, E.R.: 3
CrLEA2-48	09T025271	228	25.83	9.63	28.59	105.39	0.153	10.96	vacu: 3
CrLEA2-49	10T025889	190	21.52	9.52	31.74	88.79	−0.412	9.47	vacu: 5, extr: 3, E.R.: 3
CrLEA2-50	10T025950	185	20.54	8.80	27.57	114.27	0.268	4.86	extr: 3, vacu: 3, E.R.: 3
CrLEA2-51	10T026128	183	20.17	9.67	34.72	89.40	0.371	5.46	chlo: 7, vacu: 3
CrLEA2-52	10T026178	192	21.44	8.41	50.06	97.40	0.068	18.23	cyto: 7.5, cyto_nucl: 6, nucl: 3.5
CrLEA2-53	10T026179	204	22.68	10.14	39.68	94.61	−0.012	10.29	chlo: 6, E.R.: 4
CrLEA2-54	10T026917	221	24.71	9.14	41.43	97.47	−0.089	18.55	cyto: 7, cysk: 4
CrLEA2-55	11T027970	382	42.52	4.97	23.26	96.83	−0.283	17.02	cyto: 8, nucl: 3
CrLEA2-56	11T028314	258	29.48	10.11	52.91	92.91	−0.151	20.93	chlo: 11
CrLEA2-57	11T028577	244	27.78	10.10	47.89	104.18	−0.164	18.85	cyto: 5, nucl: 3
CrLEA2-58	11T029314	264	29.23	9.27	46.36	88.98	−0.062	18.56	cyto: 8, chlo: 4
CrLEA2-59	11T029316	264	29.19	9.11	47.73	90.45	−0.031	18.18	cyto: 8, chlo: 3
CrLEA2-60	11T029318	264	29.43	9.23	48.32	83.45	−0.146	18.18	cyto: 7.5, cyto_nucl: 4.5, chlo: 4
CrLEA3-1	01T003524	106	11.73	10.09	56.38	89.25	−0.325	46.23	chlo: 12
CrLEA3-2	02T007058	84	9.69	9.34	40.26	68.57	−0.664	39.29	chlo: 5, mito: 4, nucl: 3
CrLEA3-3	03T008662	97	10.57	9.91	43.60	76.39	−0.300	47.42	cyto: 7, chlo: 6
CrLEA3-4	04T012522	97	10.20	9.70	27.95	77.63	−0.252	46.39	chlo: 13
CrLEA3-5	04T013832	101	11.26	9.25	44.98	66.63	−0.655	48.51	mito: 8, chlo: 3
CrLEA3-6	05T015908	105	11.32	9.88	60.57	69.62	−0.456	54.29	cyto: 8, mito: 5
CrLEA4-1	03T008401	310	34.21	7.05	28.99	49.00	−1.093	65.16	chlo: 6, nucl: 6
CrLEA4-2	04T011481	427	46.51	6.04	36.13	43.89	−1.296	77.52	nucl: 12
CrLEA5-1	02T006409	95	10.26	5.87	48.75	41.16	−1.355	87.37	nucl: 6, cyto: 6
CrLEA5-2	03T010121	158	17.54	9.44	49.86	48.73	−1.237	72.78	chlo: 9, nucl: 5
CrLEA6-1	02T003983	82	9.20	9.20	47.60	54.76	−1.194	73.17	nucl: 6, mito: 5
CrLEA6-2	05T014654	457	50.38	9.10	56.56	52.32	−0.962	71.12	nucl: 13
CrDHN1	01T000101	80	8.58	6.49	24.98	51.12	−1.068	48.75	nucl: 5
CrDHN2	02T003923	215	24.36	5.25	57.49	51.63	−1.520	79.07	nucl: 12
CrDHN3	02T003929	190	19.95	8.81	30.05	39.11	−1.043	82.63	nucl: 13
CrDHN4	06T019348	220	25.01	5.45	43.12	47.41	−1.636	80.91	nucl: 11
CrDHN5	07T021123	194	19.47	6.28	−5.30	16.65	−1.215	72.16	nucl: 9
CrSMP1	04T011095	203	20.80	4.97	28.89	82.81	−0.185	17.24	cyto: 7, chlo: 6
CrSMP2	04T012849	278	28.83	5.20	34.95	78.35	−0.357	25.9	cyto: 6, chlo: 4
CrSMP3	04T013711	258	26.15	4.77	36.17	74.65	−0.286	17.05	cyto: 5, chlo: 4
CrSMP4	09T023499	284	29.44	4.90	28.98	79.82	−0.137	25	chlo: 8
CrASR1	04T013503	235	25.92	5.79	40.95	28.00	−1.449	90.64	nucl: 6, cyto: 3
CrASR2	07T020512	130	14.98	6.34	42.06	47.46	−1.321	80.77	mito: 5, nucl: 4, cyto: 3
CrASR3	07T020519	114	13.09	6.41	36.67	51.58	−1.341	79.82	mito: 5, nucl: 4, cyto: 3

The physicochemical parameters, including molecular weight (kDa) and pI, of each CrLEA proteins were calculated using the compute pI/Mw tool of ExPASy (http://www.expasy.org/tools/, accessed on 1 March 2021). GRAVY (grand average of hydropathy) values were calculated using the PROTPARAM tool (http://web.expasy.org/protparam/, accessed on 1 March 2021). The contents of disordered amino acids (%) in each CrLEA/ASR were calculated according to the online program PrDOS (Protein DisOrder prediction System, http://prdos.hgc.jp/cgi-bin/top.cgi, accessed on 1 March 2021), with the false positive rate (FP) = 5%, and also the correspondingly disorder profile plots for each protein were shown in Figure S2. Subcellular location prediction was conducted using the WoLF_PSORT (https://www.genscript.com/wolf-psort.html, accessed on 1 March 2021). nucl: Nucleus; plas: Plasma membrane; cyto: Cytoplasm; mito: Mitochondria; ER: Endoplasmic reticulum; pero: Peroxisomal; chlo: Chloroplast; golg: Golgi; vacu: Tonoplast membrane; cysk: Cytoskeleton; extr: Extracellular region; secr: Secretory. * The scores predicted by WoLF PSORT below 3 were ignored. The scores represent the probabilities of the subcellular localization.

**Table 2:** Ka/Ks analysis and duplicated date calculation for *CrLEA* genes.

Duplicated Pair	Duplicate Type	Ka	Ks	Ka/Ks	Positive Selection
*CrLEA1-1/CrLEA1-2*	Segmental	0.391	1.260	0.310	No
*CrLEA2-2/CrLEA2-51*	Segmental	0.125	0.992	0.126	No
*CrLEA2-4/CrLEA2-54*	Segmental	0.235	0.911	0.258	No
*CrLEA2-5/CrLEA2-28*	Segmental	0.267	1.221	0.219	No
*CrLEA2-9/CrLEA2-20*	Segmental	0.343	0.858	0.400	No
*CrLEA2-10/CrLEA2-21*	Segmental	0.268	0.981	0.273	No
*CrLEA2-12/CrLEA2-17*	Segmental	0.215	0.704	0.305	No
*CrLEA2-13/CrLEA2-46*	Segmental	0.232	0.967	0.240	No
*CrLEA2-15/CrLEA2-23*	Segmental	0.253	0.857	0.295	No
*CrLEA2-16/CrLEA2-24*	Segmental	0.105	1.042	0.101	No
*CrLEA2-30/CrLEA2-56*	Segmental	0.187	0.761	0.246	No
*CrLEA2-31/CrLEA2-55*	Segmental	0.102	0.449	0.227	No
*CrLEA2-33/CrLEA2-57*	Segmental	0.157	0.564	0.278	No
*CrLEA2-38/CrLEA2-44*	Segmental	0.320	0.591	0.541	No
*CrLEA2-39/CrLEA2-45*	Segmental	0.174	0.757	0.230	No
*CrLEA3-1/CrLEA3-6*	Segmental	0.278	0.692	0.402	No
*CrLEA3-3/CrLEA3-4*	Segmental	0.138	0.669	0.206	No
*CrLEA4-1/CrLEA4-2*	Segmental	0.419	1.700	0.246	No
*CrLEA5-1/CrLEA5-2*	Segmental	0.086	0.702	0.123	No
*CrLEA2-14/CrLEA2-15*	Tandem	0.488	1.489	0.328	No
*CrLEA2-19/CrLEA2-20*	Tandem	0.906	n/c ^a^	n/c	No
*CrLEA2-34/CrLEA2-35*	Tandem	0.141	0.384	0.367	No
*CrLEA2-43/CrLEA2-44*	Tandem	0.270	0.360	0.750	No
*CrLEA2-52/CrLEA2-53*	Tandem	0.560	0.937	0.598	No
*CrLEA2-58/CrLEA2-59*	Tandem	0.003	0.020	0.150	No
*CrLEA2-59/CrLEA2-60*	Tandem	0.056	0.126	0.444	No

^a^: Ks cannot be calculated due to saturation of substitutions.

## Data Availability

The datasets used and/or analyzed during the current study are available from the corresponding author on reasonable request. However, most of the data is shown in Supplementary files.

## Supplementary Materials

Supplementary materials can be found at https://www.mdpi.com/article/10.3390/ijms22094554/s1.

## Abbreviations

LEA	late embryogenesis abundant
ASR	abscisic acid-, stress-, and ripening-induced
LEAPdb	database for the late embryogenesis abundant proteins
DHN	dehydrin
SMP	seed maturation protein
ROS	reactive oxygen species
IDP	intrinsically disordered protein
WGD	whole-genome duplication
WT	wild type
YX	Yongxing Island
SCBG	South China Botanical Garden
