# Supplementary material for: Genome-Wide Analysis of the Late Embryogenesis Abundant (LEA) and Abscisic Acid-, Stress-, and Ripening-Induced (ASR) Gene Superfamily from Canavalia rosea and Their Roles in Salinity/Alkaline and Drought Tolerance
Source: Int J Mol Sci. 2021 Apr 27;22(9):4554. doi: 10.3390/ijms22094554 (PMC8123667; doi:10.3390/ijms22094554)
Supplement: Supplementary file 1 [file ijms-22-04554-s001.zip › ijms-1163896-supplementary.pdf]

Supplementary Materials Information

Supplementary Materials Table S1. The primer lists used in this study.
Supplementary Materials Table S2. The sequence information of all 84 *CrLEA* and 3 *CrASR* genes.
Supplementary Materials Table S3. The conserved motifs of the CrLEA proteins analyzed with MEME tool.
Supplementary Materials Table S4. The *cis*-regulatory elements identified in promoter regions of *CrLEA*s and *CrASR*s.
Supplementary Materials Table S5. The numbers of subfamilies of LEA/ASR genes in different legume species.
Supplementary Materials Figure S1. Predicted 3D models of CrLEA and CrASR proteins. Models were generated by using Phyre2 server at intensive mode. Models were visualized by rainbow color from N to C terminus and organized in order their subfamilies and names.
Supplementary Materials Figure S2. Predicted disorder profile plots of CrLEA and CrASR proteins by PrDOS (Protein DisOrder prediction System, http://prdos.hgc.jp/cgi-bin/top.cgi, accessed on 22 April 2021), with the false positive rate (FP) = 5%.
Supplementary Materials Figure S3. Phylogenetic tree of the plant ASR members. Three CrASRs were marked with symbol "▲". The amino acid sequences of plant ASRs used were as follows: SbASR-1 from *Salicornia brachiata* (NCBI accession no.: ACI15208.1); SulASR from *Suaeda liaotungensis* (NCBI accession no.: AGZ20206.1); CqASR from *Chenopodium quinoa* (NCBI accession no.: XP021737749.1); IpASR from *Ipomoea pes-caprae* (NCBI accession no.: AWW16497.1); tomato ASRs (NCBI accession no.: AAY97998.1 for SlASR1, AAP37982.1 for SlASR2, AAY98003.1 for SlASR3 , AAY98032.1 for SlASR4， and XP_004237807.1 for SlASR5); OsASRs from *Oryza sativa* (NCBI accession no.: BAG88534.1 for OsASR1, XP_015635329.1 for OsASR2, BAG89007.1 for OsASR3, XP_025880738.1 for OsASR4, BAG99580.1 for OsASR5, and BAG87564.1 for OsASR6); soybean ASRs (Phytozome locus name: Glyma.20G167500.1, Glyma.10G224300.1, and Glyma.16G166600.1), common bean ASRs (Phytozome locus name: Phvul.007G080400.1 and Phvul.004G094600.1); alfalfa ASRs (Phytozome locus name: Medtr6g037220.1, Medtr0583s0010.1, and Medtr1g098680.1), chickpea ASRs (Phytozome locus name: Ca_04381, Ca_25393, Ca_25396, and Ca_16211), and apple ASRs (Phytozome locus name: MDP0000253074, MDP0000136340, MDP0000435025, MDP0000564469, MDP0000128468, and MDP0000145407).

Table S1. The primer list used in this study.

Primer ID	Sequence (from 5′ to 3′)	Purpose
CrASR1F	TACCGAGCTCGGATCCATGGCTGAAGAGAAACACCAC	Primer pair for construction of CrASR1-pYES2 for functional verification in yeast
CrASR1R	GATATCTGCAGAATTCTCAGCCAAACAGGTGGTGGTG	
CrASR2F	TACCGAGCTCGGATCCATGGCTGAGGAGAAACACC	Primer pair for construction of CrASR2-pYES2 for functional verification in yeast
CrASR2R	GATATCTGCAGAATTCTCAACCAAAAAGATGGTGATG	
CrASR3F	TACCGAGCTCGGATCCATGGCTGAGGAGAAGCACCAC	Primer pair for construction of CrASR3-pYES2 for functional verification in yeast
CrASR3R	GATATCTGCAGAATTCTCAGCCAAACAGATGATGGTG	
CrDHN1F	TACCGAGCTCGGATCCATGGCAAATTATCAGAACCA	Primer pair for construction of CrDHN1-pYES2 for functional verification in yeast
CrDHN1R	GATATCTGCAGAATTCCTACTTGTTGTGATGATGTG	
CrDHN2F	TACCGAGCTCGGATCCATGGCAGAGGAAAATCAGAAC	Primer pair for construction of CrDHN2-pYES2 for functional verification in yeast
CrDHN2R	GATATCTGCAGAATTCTCAGTCACCGGCACTCTCC	
CrDHN3F	TACCGAGCTCGGATCCATGTCTGAAGCCCAACTACG	Primer pair for construction of CrDHN3-pYES2 for functional verification in yeast
CrDHN3R	GATATCTGCAGAATTCTCAGTTGGTGTGATGGCCAG	
CrDHN4F	TACCGAGCTCGGATCCATGGCAGAGGAGAAGCTGAAC	Primer pair for construction of CrDHN4-pYES2 for functional verification in yeast
CrDHN4R	GATATCTGCAGAATTCTCAATGAGTATCGGTCTCC	
CrDHN5F	TACCGAGCTCGGATCCATGGCAAGTTATCAGAGTC	Primer pair for construction of CrDHN5-pYES2 for functional verification in yeast
CrDHN5R	GATATCTGCAGAATTCCTACTTCTCGTTGTGTCCTC	
CrLEA4-1F	TACCGAGCTCGGATCCATGGCGTCGAGGCAAGAATTC	Primer pair for construction of CrLEA4-1-pYES2 for functional verification in yeast
CrLEA4-1R	GATATCTGCAGAATTCTCACATTTTCTCACGACCAC	
CrLEA4-2F	TACCGAGCTCGGATCCATGGCGTCAAGGCAACAACC	Primer pair for construction of CrLEA4-2-pYES2 for functional verification in yeast
CrLEA4-2R	GATATCTGCAGAATTCTTATTTGTTACGTCGCTCCAC	
CrASR1RTF	CCACAACAAGGACGAGGATAAG	Primer pair for qRT-PCR of *CrASR1* in *C. rosea*
CrASR1RTR	TAGCCAACGGAAGGTTCATTAC	
CrASR2RTF	ACCATCATCACCACCATCAC	Primer pair for qRT-PCR of *CrASR22* in *C. rosea*
CrASR2RTR	CATCCACATTTCCAGCAACATC	
CrASR3RTF	CTGCTGGTGCTTATGCTTTG	Primer pair for qRT-PCR of *CrASR3* in *C. rosea*
CrASR3RTR	GCCGCAATCTCCTCTTCTATC	
CrDHN1RTF	GTGGCAGTACTAAAGTGGTAGG	Primer pair for qRT-PCR of *CrDHN1* in *C. rosea*
CrDHN1RTR	CTTGTTGTGATGATGTGTTCCAG	
CrDHN2RTF	ACAGCAGCTCAAGCTCTTC	Primer pair for qRT-PCR of *CrDHN2* in *C. rosea*
CrDHN2RTR	CCTCGATCTTCTCCTTCAATCC	
CrDHN3RTF	GAGGAAGAAGAAAGGACTGAAGG	Primer pair for qRT-PCR of *CrDHN3* in *C. rosea*
CrDHN3RTR	GATGTAGTGGTGGTGGTTGTAG	
CrDHN4RTF	ACGATACCACTTCCACCAATAC	Primer pair for qRT-PCR of *CrDHN4* in *C. rosea*
CrDHN4RTR	TGAGGCTTCTCTTCTTCCTTTC	
CrDHN5RTF	TGGTTATGGTGCCACTGATTAT	Primer pair for qRT-PCR of *CrDHN5* in *C. rosea*
CrDHN5RTR	CGGTAGTTCCTGTTCCACTTC	
CrLEA4-1RTF	GGTGTGATAGGGTCCATGTTTAG	Primer pair for qRT-PCR of *CrLEA4-1* in *C. rosea*
CrLEA4-1RTR	GGTCTTCTGGGTGGTGTAATC	
CrLEA4-2RTF	GCTACTGATGCTGCTAAGAGAG	Primer pair for qRT-PCR of *CrLEA4-2* in *C. rosea*
CrLEA4-2RTR	GCAGTCTCCGACGTTCTATTT	

CrEF-αRTF	GACCTTCTTCGTTTCTCGCA	Primer pair for qRT-PCR of reference gene *CrEF-α* in *C. rosea*
CrEF-αRTR	CGAACCTCTCAATCACACGC	
CrASR1-BDF	CATGGAGGCCGAATTCATGGCTGAAGAGAAACACCAC	Primer pair for construction of CrASR1-pGBKT7 BD vector for transactivation activity assay
CrASR1-BDR	GGATCCCCGGGAATTCTCAGCCAAACAGGTGGTGGTG	
CrASR2-BDF	CATGGAGGCCGAATTCATGGCTGAGGAGAAACACC	Primer pair for construction of CrASR2-pGBKT7 BD vector for transactivation activity assay
CrASR2-BDR	GGATCCCCGGGAATTCTCAACCAAAAAGATGGTGATG	
CrASR3-BDF	CATGGAGGCCGAATTCATGGCTGAGGAGAAGCACCAC	Primer pair for construction of CrASR3-pGBKT7 BD vector for transactivation activity assay
CrASR3-BDR	GGATCCCCGGGAATTCTCAGCCAAACAGATGATGGTG	

Table S2. The sequence information of all 84 *CrLEA* and 3 *CrASR* genes.

**The sequence information of the CrLEA/CrASR superfamily**	
**Protein**	
CrLEA1-1	MQGAKKAGESAKETVANLGASAKSGMEKTKATVQEKTERMRARDPLQKDLATQKKEEKINQAELDKQAAREHN<br>AAAKQSATAGHMGQGHHTTGTGTGTATYSTTGQHGQPMGAHQTSAMPGHGTGQPTGHVTEGVVGSHPIGTNRD<br>PDGTATAHNTRAGGNPNDYGYGTGGTYT
CrLEA1-2	MQAAKQAVENIKETAANIGASAKSGLEKTKASIQEKTEKMSAHDEIQKEIATRKKEERINQAEIEKQQAREYN<br>AAAKQSAMAGHMSQAHYTNSGPGTETATDLTIGSRPETDATYSTTGPGPEIPTTYSTTGPGIETAIFPATGSG<br>PDTATHPIGEYAQPMGITPMGVVGSNPSGTNTGVDSTAPMSGHNFS
CrLEA1-3	MQSSKEKLQNVAAAAKEHVDIYKAKIDEKAERATARTEEEKAIVHERAKAKEAKAKMELHEAKARHAAEKLSN<br>KQSHYYGLHEHESPVVGAQQQPPLVGNQTQYQQGHHSLGAVPMSGTTYPSYPLGGNPPSSKHI
CrLEA2-1	MHAKTDSEVTSLDASSTTRSPRRAVYYVQSPSHDGEKTTTSLHSTPVLSPMGSPPHSHSSSSRFSGSRKMNHH<br>RNNKTWKDIDVIEEEGLLQSEERDRTLSRRFYFLAFVIAFFLLFSLFSLILWGASRPMKPKIVIKSIKFDHLR<br>VQAGSDSTGVATDMITMNSTLKFTYRNTGTFFGVHVTSTPLDLSYSEIVIATGNMNKFYQSRKSQRLVSVAVM<br>GNKIPLYGSGASLSSSTGMPTVPVPLKLSFVIRSRAYVLGRLVKPKYYKRVDCSITLDPKKITLPLSLKKSCT<br>YD
CrLEA2-2	MADGPPATTAATKPRRPRPPSGRTNLASCVVATIFLIFIIIVILIVYYTVFKPQDPKIAVSAVQLPSFSVVNG<br>TVNFTFSQYASVRNPNRAAFSHYDSSLQLLYSGSQVGFMFIPAGEIDAGRTQYMAATFSVQSFPLSAPARLGP<br>TLANGDGVGFNYGLRVEPTMEIESKLEMAGRVRVLHFFSHHVHAKAGCRVAIAVTDGSVLGFHC
CrLEA2-3	MAQSPEQVKPLAPFISSNYLSRQEDQNISERKIIRIRKFVRCCGCVTALVLIFVVIILVLGFTVYNVKEPEVR<br>MNRVILLNRTLANGAINDVTLLADISLKNSNAFTFRFGYTNTTVYYDGTEIGEGTSPPGKAKARRTIRFNVTM<br>EIMAKKLLAVPSSESDIRDDQALNISSYTMVDGKVKIYNLFMKKVVVELNCTIQYNITTGLTSSNGGNCLGLV<br>DL
CrLEA2-4	MATNMPKFKRKLGMGADGRTNPIVWLIAIICTIIAVAVVVAGIAVFIGYIVIHPRVPIISVTDAHLDFLRNDY<br>AGRLQTQIRIVVTAKNGNAKAHATFSKMTFNISYQGQDIAVLVADPFEVPKNSSKELHYVVQSSSIPLNPDQM<br>EEVTNAWKQNEIEFDFKGAARTQWRVGPLGSVKFLCHLDCDLKFRPLNGSYVPSHCTSKSK
CrLEA2-5	MMSGRENVVRVSEEGEATSRQQQLYYAATPPSSHSSTCKGCCCCLFLLFSFLGLLVLAALLLIVLVVKPRNPH<br>LDLSQVGFQYMAIRPNPNDPATASLYLIIRLVLSVVNPNRVRIRYGESRLSLMFRGVTLGRASVPAFFQRAHS<br>VTEVVATVAVDGVNLSEADAADFTRDALLNDRVELRVLGHVAAKIRLFNLYSPPLQVSVNCVIVISPRKQSLT<br>YKQCGLEGLNV
CrLEA2-6	MTEPSSKPNGNGAINGAATTTNGNPGPVKSQLYNPNRQLYRPQSHYHRRQRSHRNLCCCCCFWTILTLLAVAL<br>LAAIVGAALYVLYRPHRPEFSVTNLRIAKMNLTTSSDSPSHLTTLFNLTLISKNPNNHLVFFYDPFTVNVFSN<br>SVPVGNGSLTAFTSDKNNQTSIRTVLSGSQDLDTESLTSLRSGLKKKGFPVEIQMDTKVKMKMDWLKSKKVGI<br>RVTCDGIKGTVPAGKTPAVASVINSDCKVDLRIKIWKFSF
CrLEA2-7	MEERVSSQLSPPPPVVQSPPQDPSIQNHSSKALQLSPNLDPGTYVVRVPKDQVYRVPPPENAEIAELHRSTPQ<br>KNTKRTRCWCVTCFIAVIIVILIIIGGILGGLFSVVLKPKDPRFSIQHFNVLKGTPKPKYNITLQAHNPNSKV<br>GIWYKEGGHVSLSLRPKEIASGSYPSFHQTPHDSTPFGVTLKASKAGFPKEVEESITNHKHKVHVAFSLTIHV<br>PARMKMGLLRSGTMQFDVTCQVTLDSLAKTPRVLSQQCQTKRH
CrLEA2-8	MSQITIKSPKHCATKQGLKIERNYKKLFYVFSAFFTSILLLILLIWLILHPSKPQFSLKQVDIYQLNLSGPNL<br>NSSIQLTLLSKNPNQKVGIYYDEFQVYATYKGQQITGDTPVPPFYQGQEENNLLTASLVGNGLPVAPSLGYEV<br>GRDQTVGRLVLNVKVNGKLRWKVATWVSGRYRFNVNCVSIIAFGPSAPLTSKQGARCSTTI
CrLEA2-9	MKAGSGKGRKVCLTVTGVVIAIVMLMVILALTVFKAKHPVTTVDSVMLQDFHMSLDIAKLKVDLNVTLDVDVS<br>VKNPNKVGFKYSDSTAHLNYRGQLIGEVPISAGEISSGETKGFNLTLTIMADRLISNAQLYSDFTSGTLPLNT<br>FIRISGKVSILGFIKVHVVSSTSCDFAVNLSNRTVGNQECQYKTKL
CrLEA2-10	MILSTRLRPLISGNREVTCDTYHHVSAWDETCGIRVWSRNLLLNKAKDFVSEKINNVAKPEASVTDVDFKRVS<br>RDSIEYLAKVSVRNPYSTPIPICEIKYSFKSASREIASGRMPDPGSLKAKDTTMVEVPVKVPYSILMSLAKDI<br>GADWDIDYQLDLGLVIDLPAIGNFTIPLSHNGEVKLPTLSTMFA
CrLEA2-11	MAEREQVRPLAPSIDRPSSDEDDTTLHRTSPARTKFIKWWACPLAFLLLLAIIIIVLIFTVFHVKDPDIRMNG<br>VKITKLELVNTLTPKPGANMSLIADVSVKNPNVASFRYSNTTTSLFYHGIMVGEARGPPGRAKAKRTIRMNVS<br>IDVITDRIVSNPDFRTDFVSGLLTMTSFSRVPGQVKILNLFKKHIVVKMNCTTTFNISTQGIQEQSCKRKQKT<br>TCSTISPFGATNSLKVIISYGTKQDLRVI
CrLEA2-12	MQIHDQEEPTQQHSQNQPHHFRVKVPQQQHYNMDKGIPPRFKPNAPKREHCICITVFLLLLGIIILILWLAYH<br>PSKPRFTVASAAIYGVNTTSPPLMSITMQFNILIRNPNKRVSIYFDRLSAYVSYRNQPITPRVMLPPLFLEKH<br>SAVSLSPEIGGVPVPVSEEVSNGLAMDESYGVVAVKLMFLGRLRWKTGDINSAHYGLYVKCDVLMGLRKGFVG<br>QVPLLGAPVCEVNT
CrLEA2-13	MTDRVHPSANGGKTATFPATKSQLYGATRPSYCPQPYHQRRSSSRGWCCSLCLCLILILLFLLLLIGGAGTVA<br>YFLYHPQRPSFSVTSLKLSYLKLTSSSTLNSKFELKLSATNPNNKIVFSYDPTSVSILSNEDIDIAHGTIPSF<br>RHSQRNITILKVSIASSEESVESDAAMRLKGIMKSNSGLALKVKLETKVQANMGVLHTPSVPVTAFCDGVAVT<br>LPDGDKPATASIANTECNVDVRFKIWKWTVG

CrLEA2-14	MYEKQPQLNGAYYGPSIPPPKSYHRPGRGGGCDCCCGCIFSLIFKLILTVIIIVGIAVFLFWLIVRPNVVKVHVTEATLTEFNYTGNTLYYDLALNMTIRNPNKRLGIYYDRIEARAIFQDARLDSKFLEPFYMGHKTTKLLNPAFKGHQVVPLNTDQTAELKKENSTGIYEIDVKLYLRVRFKLGVFKTKTLKPKVTCDLRVPFTSPNGPSPALGAFQTTKCDWDR
CrLEA2-15	MADKQPQLNGAFYGPAIPPPAQQPRYHRHHRGRSCCCCLFGIFWKILVALIVLAGLAGLIFYLVVQPRSFKFYVTEANLNQFEYHSNNNTLNYDMVLNFTARNPNKKLSIYYDKVEALTFYEGARFSNYDVITHFNSFRQYKKSSSPMSGVFKGNRVVVLDNDQLSELNRDQNERVYDIYVRLYFRIRFRLGDVISRDFKPKVKCHLKVPLASTTTTLFQPTKCDVDF
CrLEA2-16	MSVKECDHHKGKKHKIFRRIFWGIVIFVFIVLVTILIIWAILKPTKPTFILQDVTVYAFNASIPNLLTSNFQVTLSSRNPNDKIGIYYDRLNTYVTYRNQQVTYRTSIPPSYQGHKEEDVWSPFVYGTNVPVAPYNFAGLSQDQSNGNVLVIVKIDGKVRWKVGAFISGHYNLYVRCPAFITFGPRSNGIAIGDNAVKYQLVQRCTVGV
CrLEA2-17	MEKDKISGSPKRAVCTCITIFLLLVAITLLVLWLVYRPHKPRFVVVGAAIYGLNTTTPPLMSATMQLTVLIRNPNRRVSVYYDRFSAFVSYRNQAITPQVALPPLHQDKHSTVSLSPVIGGTAVPVSVEVSNGLMVDEAYGVVGLKLTFQGRVRWKAGAIKTAHYGLYVNCNLLMGLKKGFVGPVPLLGAQPCDVDL
CrLEA2-18	MDVSASNSDCFWWKELHPGFSFKKCCFFLLIFLLITCMVVGLATFFVILIVRPHKPVFSVREVQINFYKIDDRSNLTLLVSSVISLTLNAENHNKFGIGFSPSRFLVYQEGLHIGTIRVPWFFQPPHTENVTVPSRVLLQCVNLSKIVANTSLQEMSKQNTAPIKITGDARVHVWVLHIKLFEIKIALDCGMNFNIREFAFTNEAFGAKISKSDLGSVLSDSKGISMKCASSVYI
CrLEA2-19	MSALAPKQEIMTEAQNNEDQEQVVVISQKKLKRRKVCVITTGVVLLLLVVLIIVGLVLVLNLLKPKDPRADLVSATLEGIAPRVTLPAIDIQINVTLDLKIQVENRNHVSFKHQGGKSVLLYGGKEVGETQLYPGVIPSKGSTILPCRLTLQAEKLASNFSGLVEDLMGGQISMGAVTRIPGRVTFLGFIKKHIIAKSNCQFTVSVSDFKIISQACKNKAKL
CrLEA2-20	MIEKYGHRKKSNMCLVVTCIAVIAILAVATILVSTMLKPRQPITKVEGIRLDDMNMNMNMFKMRMDINVTLKVDMSVENTNKFGFVYNDSLAQLNYGGELIGEAPIPNGEILSEETKGMNLTLTIMADRMFSNSQAFKDVTTGSLPLNTIVKLSGQVIILGLINFHVGSTLSCDFNLNVSNRTIDHKECHHDTKI
CrLEA2-21	MRVGLGVKVSLGVIGVSLILAAFKMSELLDKAKNYVSEKVANMPKPEAAVADVDFKRVSREGVEYLAKVSVSNPYPSPIPICEIKYSLKSAGREIASGTIPDPGSLKAKDTTMLDVPVKVPHSILLSLARDIGRDWDIDYQLDLVLIIDLPVVGNFSIPLSQQGEIKLPTISDMFT
CrLEA2-22	MCKTVGRLLALLLVIIPVTVTVTAFLFCLIVRPNVVKFHVTDATLAQFSYTNNTLYYDLTLIVEVRNPNTKIRIHYNYMEEVVLYQNVKFCSLILGTYIQPHKNTAMLTPVFKGQQVMSLNKDHILETYNNEKRSGIYHIDFKLYLKVKFDLGVYKTNNMKSTVTCNLQLPLQSYNGTSTAGRFQLTRCNFDYKHILFIF
CrLEA2-23	MADYKQPQLNGAYYGPSIPPAEPPRQRAHRGRRCCCCLFSFFWKFLLAIAVLIGLAILIFWLLVQPRAFKLHVTEASLTQFNYTSNTLQYNLVLNFTARNPNKKLNIYYDKVEGHVFYDSVRFASTDVITWQNSFRQYTKSTNRMSGVFSGQRVIVLDHNRASDLEEDKRSGIFHIDVRLYFTIRFRLGDFIWNSDIKPKAKCGLKIPFSSNGTTVNEFRPTKCDVDF
CrLEA2-24	MSVKECDLHKGKKRKIFLRIFWGIIVFLFIVLVTILIIWAILRPSKPSFILQDVTVYAFNATVPNLLTSNFQVTLSSRNPNDHIGVYYDRLDTYVTYQSQQITYRTAIPPSYQGHKEIDVWSPFVYGTNVPVAPFNYAGLSQDQSNGNILVTIKIDGRVRWKVGAFISGRYHLYVRCPAFINFGTRNNGISVGENGAIKYQLVQSCSVSV
CrLEA2-25	MADHQRQRIHPMEVGAPPPPTIPLVPPGSSRSEKGNPLHRPPQLHARPAIYPSPKKGNCFCKCICWTLCLVFLLLIILAATVGILYLVFKPKLPNYSVETLRISDLRLNFDLSLYAKFDVKITANNPNKKIGIYYEEGGKLSVWYRKTRLCEGSLPKFYQGHQNKTVLNVSLTGQVQSGSTLMTALQQQQTGRIPLDLKVHAPVAIKLGRFKLRKVRVLGECLLVVDSLSSNNLITIKASNCKFSMKL
CrLEA2-26	MRSNDHIPVHHVAGPNPKPMKRHRTARYYVHRVHESLTTRVSKIICATFLGLLAIVGLITFILWLSLRPHRPRFHIHEFNMPGLTQQSGFENARITFNVTARNSNQNIGVYYESMDGAVYYRDQKIGSTSLLNPFYQEPKNTTEVDGDLSGATLTVSSQHWAEFQSDRADGSVVFRLEVTSVIRFKISTWDSKRHTMHANCDVGVGPDGSLLSNYKDKRCPVYFS
CrLEA2-27	MKPNHNITMLAKTDSEVSSLSQSSPARSPRRAVYYVQSPSRDSSNDGEKTTNSFQSSPLQSPLGSPPHSHSNSSLGPHSRESASTRFSGSRKTNESNRKGPWRPWKDQFHAIEEEGLLDAHDAAQHGFPRRCYFLAFVVGFVVLFSVFSLILWGASRPQKPAITLKSITFDQFVIQAGADVSGVATSLVSMNSSVKLTFRNTATFFGVHVTSTPLNLNYYQLTLATGSMPKFYQSRKSQRSVRVMVKGSHIPLYGGGANLNSVNGAPVEAVPLKLSVMVRSRAYVLGKLVKPKFYKKIDCSVVMDPKKMGKPISLMTKCTYQ
CrLEA2-28	MTNRGGERERMTTTGNQPQPHPHPQPQPQPQPQPQPNGERRQRPAPASAPETPAQYSASSNGNGYRQYNPTTPSRSSSSASFKGCCCCLFLLFSLLALLVLAVVLVIILAVKPKKPQFDLQQVGVQYMGITPNPPSTASLSLTIRLLFTAVNPNKVGIKYGQSSFTVMYRGIPLGKASVPGFYQDSHSVRQVVATIAVDRVNLLQADAADLIRDASLNDRVDLRVLGDVGAKIRVMNFDSPGVQVSVDCAIVISPRKQSLTYKQCGFDGLSV
CrLEA2-29	MAHPPPQSQPQAGKPNRSILLRYIAMIILFLIIILVGIAVLIIWLVLKPKRLEYSVEDAAIHKFNLTDANHLYANFDFTIRAYNPNSRVSLYYDSVEVAVRYEDQTLATNAVKPFFQSHKNVTRLHVGLTAQTVALYDSVPKDLRLERSSGDIELDVLLRARIRFKVGVWKSKHRIMTIFCSPVFLTFSKAKSFQRTPCEVQL
CrLEA2-30	MPSLSPSPLEKLGIPKQPRNQHFRELEAPNSLSNTLVILGQPHLQRTKPIIWCAAVLCLIFSLVLIFFGIATLILYLAMKPRNPTFDIPNASLNVVYFDSPQYLNGEFTLLANFSNPNRRFHVRFESLKVELFFSDRLISTQSIKPFTQRPKETRLQPVNFISSLVFLPQDVGVKLQRQVQNNRVNYNARGTFKVRVNMGLIHLSYWLHSICQIEMTSPPTGILVARQCITNR
CrLEA2-31	MSTSDKPEIVERGSKDEKHKEGEKEEGKGGFIEKVKDFIHDIGEKIEGAIGFGKPTADVTAIHIPSINLHKADLVVDVLIKNPNPVPIPLIDIDYLIESDGRKLVSGLIPDAGTIHAHGEETVNIPVTLIYDDIKQTYNDIQPGSIIPYRVKVDLIFDVPILGRFTLPLEKTGEIPIPYKPDIDLEKIHFERFSFEETIATLHLKLENKNDFDLGLNALDYEVWFGDVSIGGAELTKSAKIEKSGISYIDIPITFRPKDFGSAVWDMIRGKGTGYTMKGNIDVDTPFGPMKLPISREGGTTRLKKKKEDRDYDDDDDDED
CrLEA2-32	MADRVHPQDSPPVSGESQTTSSQQPAIVQNPAPQPLSEKPVPPPGTYVIRIPKDQVYRVPPPENARRQDQFIRRKHRRSRCCCCFCWLIGFIFILIVLLGIAAGVLYLVFRPEAPKYTIDGIAIRGMNVTSPSSAAAVSPEFDVTVKADNPNDKIGIRYETDSSAEIFYNDVRLCNGALPAFYQPSNNVTVFKTVLRGNGIKLRSGDRSALLEAQTKRKVPLTVSLRAPVKIKVGSVQTWKITVKVDCDVTVNELTAQAKIVSKHCNYNVDLW
CrLEA2-33	MADPQRIHPVHDVEAPNHPTAPLVPRSVSKSDAGDPENVVHHPPPPTSLPSQPVMHSKPPKKRRSCCCRFLCWFISILLILIVAIGITIGILFLVFRPKLPKYSVDELRVTQFDLADNNSLSVTFNLTIIARNPNKKIGIDYRGGSHISAWYMDTKLCEGSLPKFYQGHRNITVLSIPLIGHTQDASGLQSTIQQQLQQTGNVPLNLKVKQPVRIKLGKLKLFKIKFRVRCRLVVDNLNANNSIRIRSSSCKFRFRL
CrLEA2-34	MNCRPRCSFCCICCIYTTIYVLMFILMLLLIIFWIVISPSSVKFHVTDATLTQFNLTTNNTLHYNLKVNITVR

	NPNNNVKVYYRAITAIAWYKDNDIGLVSLTPFDQGHKNTTFLQAVFIGQSVIKLQAKQLDEYKEETSVGIYND
	LAVDLDLTIRAKFGRFRSSHYNPPIVQCRRLKVPLISNAKTTPPFSVTKCSTGYFFKDRDADAEG
	MCCKPRSTFCCIYCTFYTLMIIFFLSVIIFWIIISPSNVKFHITEASLTQFNLTNNNTLYYNLKVNITVRNPN
	NNIIVYYRRIKAIAWYKDNDFGWVSLTPFDQGHKNTTFLQVEFKGQSVIKLKAQQLGEYKEETSVGIYNDLAV
	DLDLRIRAKYGRFKSSRFNPPIVQCRRLKVPLISNGKTPTPFSVTKCKSGSFFVDLISPSSVKFHVTDASLTQ
	FNLTNNNTLYYNLKVNITVRNPNNNIIVYYRGITAIAWYKDNDFGWVSLTPFDQGHKNTTFLQAVFKGQSVIK
	LKTKQLGEYKEETSVGIYNDLAVDLDLRIRSKYGRSGFSFCCFFCIYNTFYTFIFLFTLSLIIFWIIISPSSV
	KFHVTDASLTQFNLTNNNTLYYNLKVNITVRNPNKKSIVYYSCITAIAWYKDNDFGLVSLTPFDQGHKNTTFL
CrLEA2-35	QAVFKGQSVFKLKAKQLGEYTEETSARIYNDLAVDLDLRIRFKEGRFKSSRFNPPIVQCVA
	MTGKDEFLSYSPLPSNPNPYSNNPNPNPNPNPYHYPQNVVVLLPSYRSPSLRRRRCILYSAALFIFFLLVAA
	VFIFYPSDPEIRLVRIRLNHIGIRTSPRPILDLSFSITVKVRNRDFLSLSYDSLSVSVGYRGRQLGFVTSSAA
	GRIRARSSSYVDATLTVNGFEVIYDAFYLLQDIARGVIPFDTDTRVEGNLGLLFFNIPLKATVSCEVYVNINQ
CrLEA2-36	QTIVRQNCYPESLGDPLDQSTYIGVADT
	MHAKTDSDVTSCDPSSPRSPKRAVYYVQSPSRESHDGDKSSSMHATPACNSPMESPSHHSYGHHSRASSSSRV
	SGSYNSSWGRKGDRKRNDKEWPECKVIEEEGGNGDFYPDNKGFSRRTQIFIAMIGFVLIFGVFCLIIWGASRP
	YKPQLSVKSLTVHNFYFGEGTDMTGVPTKMLTVNCSMRMMVHNPATFFGIHVNSKAVSLMYSEITVATGELKN
	HYQQRKSRRTVFVNLQGSKVPLYGAGASLAGLVDKGKIPMTLVFEVKSQGNVVGKLVRSKHRQHVSCSVAIDS
CrLEA2-37	HNNKPIKLKNNACTYD
	MKQNTIGSKIFLCTPALVFPKKQDFKGNPNCSYYPEPETMAKRNLKICISVSAVFFIIVITVFLTLILTIFKP
	KNPDIFLHPVDLKNFQLLSSNSTNAPLGMVVTIVNPNYGSFRYINSTGYLEYRNTTVAKVPLETKSVPARTRT
	NVSTSAGIMTEKLIDDPKFWSEIEGGTFNLTAKATLPGKVSMFNIFKLKATVHISCDISINIIAIDSGSTCVA
CrLEA2-38	KLKL
	MSSKDCGHHEEERRQLLRRIVGAIVAFIVLILLIIFLIWIILRPTKPRFIIQDATLYTFNFSSTSPSVTSPTP
	NILTLTMQVTLAAHNPNHRIGIYYHNLYAYASYRNQQISLATSIPNTYQGHKDFIIWSPFIYGNAVPVSPFLL
CrLEA2-39	SALQLDQSTGTVLVNVKVNGRVKWKVGTWISGRYHIYVNCPAYIRFAGDRSNAIGGVTPAVKFQLLQSCSVDV
	MLSNILTWTGPMGDPEGLWPREVTSIALWRGASLRSAQISVLIFGLNVSHLFLNIPQLIHSFLILQVAMGKKV
	KWSWSSAFVGAASAVAATAILSAKPKDPTFHLISINFTSLKLNLPLLDAELLLTVHVTNPNIAPIHYSSTTMS
	IFYEGSFLGSAQVQAGSQPPRSCQLLRLPARLHALQLAHHATRFVHDVARREMVLDAAVDIAGTAKVLWWDHN
CrLEA2-40	FKVHVDSRVTVDPVFLDVIDQESTSELEVFTAA
	MGGRMHTKSDSEVTSNSIDHSSSSPPRSPPRRPLYYVQSPSNHDVEKMSYGSSPLGSPHHHIHYYLSSPIHHS
	RESSTSRFSASLKNPRPFSSSWKKLHPHPHHGVDDDEDDDDDDLHDSSRNLRLYLCFFLLFVLLFTLFSLILW
	ATSKTYKPQLAIKSIVFENLNVQSGNDGTGVPTDMLSLNSTVRILYRNPGTFFGVHVTSTPLQLTYYQLALAS
	GQMQKFYQSRKSQRKVVVVVLGHQIPLYGGVSGLLGNYKEHIDSVALPLNLTFAVRSRAFILGRLVKSKFYRR
CrLEA2-41	IRCSVTLHGNKLGKPLNLKDSCVYK
	MSSDPYAHQRYVSLQNQGYVSGPPPYGYGRKPPRYHHHSGSGCLCGCLKFFCCCFYSCCRCCICAFFIFILIL
	VIIIMVLYYLLKPDIPSYNIQGIDINTFDLKIDNKLFSNISIVVKADNPNEGIGLNYLDNEVRLLYSGSQLCS
	GAFPPFLQPERNVTTFNVTLKGESEFGPEMQQHLMQEQDKGTIHLLIAVRLPIRLVVDDLIHLRKFVVNINCS
CrLEA2-42	MIIDHLEQNKKPNILRKDFIYGIEF
	MVSRGGKICIGVSLILVIAIGTVIATLSLTIFKIRDPVISIHPVGLENLKFSLNFSSNVSLGMILTMGNPNYA
	SFEFKNSISHVNFHKNVVADVPIQGEVVPARGTINVTTSADLMVRKLILDPKFWSDILGGHLNLTSTATLPGV
CrLEA2-43	AHVLNFIKLKATAYSSCTISLEIRTKNISSNCISKIKL
	MATRSVKICLSVSLLFLIIVTIVIVTLFLTLFKPKDPNITVHPLGLENLQFSLLPNLTLNVSLGMLITIGNPN
	YGSFEYKNSTGYIKFHDTVVAQVPMEAELVPARSTINVNTTADFMVGKMINDPNFWTDVLTGTLNMTSTSTLP
CrLEA2-44	GEARMLKIIKFKATAYSSCDISFNITSKHVDTKCISKFKL
	MTKDCGHHDEERQQLLRRIFAAILGFIILILLVIFLIWIILRPTKPRFILEDATVYAFNLSSTGDIPSPTAPT
	PNTLTLTMQVTLTALNPNSRIGIYYHNLHVYASYRGQQVSLATELPPTYQGHRDTTVWSPFLYGNAVPVSPYV
	LEVLEQDKTSGGVLVNVKVNGRVKWKVGTWVSGRYHIYANCPAYIRLAGDRDTGIGVAAPAVKFQLFQNCVVD
CrLEA2-45	V
	MTDRVYPSAKPAANGNGAAANPSFPATKAQLYGATRPTYRPQPHHRRRSRRRCCCTFCFWLILILLILLFLLG
	VAGTVFYLLYRPHRPSFTVTSLKLSYLNLTTSSSSLSSRFDLNVSATNPNKKILFAYDITSISILSGDVDVGD
	GTIPAFQHGKKNTTLLKASISSSGQALTSDDASRLKANMKSKSGLPLEVKLETKVKAKMGNLKTPKVGIRVFC
CrLEA2-46	DGIRVTLPTGKKPATASTKNVKCDVNVRFKIWKWTV
	MLSAKSESDITSLAPSSPSRSPKRPVYYVQSPSRDSHDGDKSSSMQATPISNSPMESPSHPSFGRHSRNSSAS
	RFSGIFRSSSGRKGSRKRNDKGWPECDVILEEGSYHEFQDKAFTRRFQALIAVFTFVVVFTVFCLIIWGASRP
	YKTEVTVKSFTVHNLYVGEGLDFTGVPTKMLTVNGTLRMSIYNPATFFGIHVHSTPINLIFSDITVATGKLKK
	YYQPRKSHRIVSVNVEGNKVPLYGAGSTITVSQTGVEVPLTLNFEIRSRGNVVGKLVRSKHHKEITCPLVINS
CrLEA2-47	SGSKPIKFKRNACTYE
	MMCWIYVPSSTFINCNSKNTESVQRKAMKISKAAYDLLLRKPHQHQNDIVYSRRCVLTLTCTLGLVLLLTTIY
	AFWPSDPDLKIVGFRLKRVKLHPLPPVTVDISMLITLRVHNTDMYSLDFGAVNVIVAYRGVRLGHVTSSHGHV
	RARGSSYVDADVEFSGISVLPQMVWLLEDVVKGIVPFDTVSQVRGQLGLLFFHFPMKSRLSCEVVVSIQNQTI
CrLEA2-48	VRQHCHRGR
	MCESKSFYKWLMQFICLLGLLVLCLWLSLRPKSPSYSIVCISIEQASNSNQNGTISYQLQIENPNKESSIYHD
	DIILSFLYGQMEDEVGEDTIGSFHQETGNTRDKIGTVNAKPRPFKPLLNAISNATAELTVALKTKFRYKTWGI
CrLEA2-49	KSKSHGLHLKGILPIDSNGKLSRNKKKYPLSPNSQKIGRYKIKH
	MRCSKGLKICCAVSAILLIIILVVVLVILFFTVFKPKDPDIILQSVKLERIEIGFPSPEINVSLGLVVTVENPN
	HGSFTYQNSTAYLNYRGNIVAEAPIHEDTIPARGDHNISTSLDIFADIRKFKDLPSDFLRGVINFTSTTTLQG
CrLEA2-50	KVKILHLFKFKATSYSTCDLSLLVHDKSINSTCNTKIKL
	MTKPQSRTNLASCFVATIFLIFLVIVVFVVYFTVFKPQDPKISVSAVQLPSFSVTNGTVNFTFSQYASVRNPN
	RGTFSHYDSSLQLLYYGRQVGFMFIPAGKIDAGRTQYMAATFTVQAFPLGFGPTFINGPINVGPTMEIESRIS
CrLEA2-51	MAGRVRVLHLFSHHVEAKALCRVPIAVPDGTVLGFHC
	MSETVTESRSSPERGGIYTHTTSEKLIAMFPIRKEEEKSSKCVVYALVTIATLLGVVCFSSIFLVVRDPKIEL
	ISARLVDNINHTVISASSPFNLSMIGRVSLWNPNFGRFYYDNTTVSVMYGDFSIGASELGGAILEAREIKEFN
CrLEA2-52	SIVNMRFSEELVNLTNDISSCMLKLRSHSDLSGIGYVLKFIKKRKTIQMDCTMNLDLTSSSLRLLQC
	MEMATKHSSSRHHTIHQETAIYKPIHQERSSKWFVFLFSAFVILCAVLLVFASVLRVRSPEVKLRSATLKQIN
	YRASPAPSFNATLITFLRIRNPNYGAFSYENSTVTVLYAGENIGDKGIGSDRVSYRETKQMNVTVNVRSSKLP
CrLEA2-53	VTGNLSGAINSGMLNLTGYVKFSGTVHLLKIINSRKTIQIACTMNLNLTSHAIKGIQC

CrLEA2-54	MPSHHDHGHVAEHHHHHHRSPIPRRLNTPHNDSTHPLIWLAAILCTIIAIGVVIAGIVVFIGYMVIHPRIPIISITNAHLDLLRNDYAGLLQTQLRIIVVAQNGNAKAHATFSDIKFNVSYQGQGIAVMVADPFDVPKNNSKTLPYVVQSAPIPLTPDQMEEVDVSWKRDIIGFDIKGSARTRWRVGPLGSVRFWCTLECQLKFHPSNGSYIPKRCTSESK
CrLEA2-55	MSTSDKPEVVERESKDGKQKEDNDEKGGFLDSVKGFIQDIGEKIEETIGFGKPTADVTGIHIPSINLEKVELVVDVLIKNPNPVPIPLIDINYLVESDGRKLVSGLIPDSGTIHAHGEETVKIPLTLVYDDIKNTYNDIKPGSIIPYKVKVDLIVDVPVFGRLTLPLEKTGEIPVPYKPDVDLEKIQFERFSFEETVAILHLKLDNKNDFDLGLNSLDYEVWLGDVSIGGAELTKSAKIVKTGITDIDVPITFRPKDFGSALWDMIRGRGTGYSIKGHIDVDTPFGAMKLPITKEGGTTRLKKKKEDGEDDEEEPDWNSVYDSCLAEFQYRHSGDAQKQGCFSKLTLETVSFNLIRVVFLVYPKGLKKKWQPVIVSVLLFTA
CrLEA2-56	MLALPPPPPPPPSLPSPPSLAIRQTKQVSPGQIVKSKQPIRQNSQELEAPDYTTTKSIRPLPRLRQPPFQRTNPIIWFGAVLCLIFSLVLIFFGVATLIIFLAIKPRNPTFDIPNANLNGLYFDSPEYFNGDFTLLVNITNPNRKMDVRFESLDVDLFFSDRIISTQSIQPFTQRRKESRLESLHFISSLVFLPEDLGVNLKKQVQGNRVNYNVRGTFKMRITMGFFHLSYWLHSRCQIEMTSPPTGVLIARNCITKR
CrLEA2-57	MSDHQRIHPVHDVEAPHRPLVPSNIGKSDKDVQPLPQRTIPVMHSKPPKKRNCCCRVLCWTLSVLVILIIALAITIGILFLVFRPKLPKYSVDQLRISQFNVSDNNSLYATFNVTITARNPNKKIGIYYEGGSHISAWYMDTQLCEGSLPKFYQGHRNITVLNLFLTGQAQDATGLQSRIQEQLQQTNNIPLNLKVNLPVRVKLGKLKLFKVKFRVRCRLVVDNIGANNDIKISSSRCKFRLRL
CrLEA2-58	MTDPVHLKNSPPQSNDAQTSPPHEAAVTSNSSSLAATYVIRIPKDQVYRVPPPESALRYANYSRRKNRRSRCCCCLCCFISIVFSLVVLLAIASGVFYFVFRPKAPDYAIERVAVKGMNLTSPSFTAALSPEFDVTVRADNDNSKIGIYYEDGSSLEMFYNDARLCDGALPAFYQPSNNVTVFDTVLRGNGIALTTSDQTALVNDVAKQSVPLTLKLRAPVKIKVGSVKTWKITVKITCNVTVDQLTMKARIVNKDCRYRLALW
CrLEA2-59	MTDPVHLKNSPPQSNDAQTSPPHEAAVTSNSSSLAATYVIRIPKDQVYRVPPPESALRYANYSRRKNRRSRCCCCLCCFISIVFSLVVLLAIASGVFYFVFRPKAPDYAIERVAVKGMNLTSPSFTAALSPEFDVTVRADNDNSKIGIYYEDGSSLEMFYNDARLCDGALPAFYQPSNNVTVFDTVLRGNGIALTTSDQTALVNDVAKQSVPLTLKLRAPVKIKVGSVKTWKITVKITCDVTVDQLTMKARIVNKDCRYLLALW
CrLEA2-60	MADRVHPKNSPPQSNDAQTSPPHEAAVTTKPSSPAATYVIRIPKDQVYRVPPPENAHHYANYTRRKYRRSRCCCCLCWFISIVFSLVVLLAIAAGVFYFVFRPKAPDYTIERVAVKGMNLTSPSFTAALSPEFDVTVRADNGNSKIGIYYEDGSSVEMFYNDARLCDGSLPAFYQPSNNVTMFEMVLRGNDIALTASDQTALVNDVAKRSVPLTLKLRAPVKIKVGSVKTWKITVSIDCDVTVDQLTAKARVVNKDCRYRLALW
CrLEA3-1	MARSLSQPNRLGFIVAQSISLIPVYRRGYSAASDVLGRVGLGIIARRSGMVGRTEEKPVTRDGSEAYSAWAPDPVTGYYRPINHTPEIDAVDLRQMLLNHKLRSPH
CrLEA3-2	MANFLLRKRCYGVVAECLRLEMESTMVKAKAASPNGNSNKDEIFWMRDPKSGNWIPENHFGEVDAAELRNKFLPRHTKLPNQLK
CrLEA3-3	MARSFTNAKIFSAVVLDGFPSVFTRRGYAVATQSATRGGTASISGKVAPKAVEDKVASNNKVSWVPDPVTGYYKPENINEIDVAELRQTLLRKKFNN
CrLEA3-4	MARSFANAKVLSALVADGFSNTLTRRGYAAATQSATRGGAASISGKIAPKSGEDKVAATEKVSWVPDPVTGYYKPENTKEIDVAELRATLLGKKFNL
CrLEA3-5	MVRSGITKANMLLLRSCRGTERAKSGLSKLLPAATENSGGISKSSGKMENSELWVPHPRTGIYFPKGHEWVMDDVPEDAARLSQTHWFRNIDGVDNPKYQT
CrLEA3-6	MACSFAQAKRVLAFALHRRGYAVMSDASVRGGLDRIGSGSRSRSGSTVVGKVEEKSVSKSGSEGSSAWAPDPVTGYYRPINHTNEIDPVELREMLLNHRVRSSSP
CrLEA4-1	MASRQEFKEDRAEAAAKLAAKDIGHVNRTNEREESYTLHSDIHFKEARAEAAAKLAAKDLEDVNRARESAFREKQRYCDEQKPGVIGSMFRAAKEAVVGKPHEGVEVRDRGTSPGVYDYTTQKTTETKDATKQKAGEYADYTSQKAKEAKDKAGEYKNYAADKMKEAGDATMNKAKEAKDATMQKAGEYKDYTAEKTKEGTESAAGKLGELKDSAADAAKRAMGFFAGKKEETKEKLSGTEEEAMRKMEHVKVEDRKYGGGGGGKMEIKVEESRPGAMVDVLRAADKALNNVGRLDEEGVIHVERGREKM
CrLEA4-2	MASRQQPEDRAEAAAKVASKELEQVNKARDHNLAIEDQQQHDQEKRGVISSVLRAVQGTYENAKGAVVGKKDPADPYTTEVVHHVNVEDEDARTGEVRDISPNKAGSKVGEYADYASQKAKETKDATAQKAGEYADYASQKAKEAKDKTMEKGGEYKDYAAEKAKEAKDATMNKAGEYKDYTAEKTKEGKDTTMGKLGELKDSATDAAKRAVDYLSGKKEETKEKAAETAEATKNRTSETAETAKKKATEMKDAAKEKAAETTEATKNRTSETAETAKKKAAEMKDAAKDKAAETTEATKNKTSETAETAKKKATEMKDAAKDKAAESTEAAKQKTAEAKDKTKENVSGGGEEDTRRKMEELKVHGKGYGEEPERRGGDKVVLRVEESRPGAVAETLKATDQMTGQTFNDVGRMDDEGVINVERRNK
CrLEA5-1	MASKQQNRQELDERAKQGETVVPGGTGGKSLEAQQHLAEGRSRGGQTRKEQLGTEGYQEMGRKGGLSSMDKSGGERAQEEGIDIDESKFTTASRN
CrLEA5-2	MNLSSFTCVSNHKVFPLANTRNYHPHSVPLQRQKRVLRRKGLSVNNMASQQQNREELDEKARHGETVVPGGTGGKSLEAQENLAEGRSRGGQTRKQQLGSEGYHEIGTKGGQTRKEQMGTEGYKEMGRKGGLSTMDKSGGERAEEEGIDIDESKFRNH
CrLEA6-1	MANNGKQVEHKTGECPNRKEKGKIEMLPLEISPCVKYNDVQKEYNRNAYDTQRHLQPKPNQAGGTTHIAPTPSDAPLKLTTK
CrLEA6-2	MENQTEKKAESESGKEGLPMKDSPYLQYKDVEDYKRQGYGTHGHQEPNPNQGAGATEAPTLSGAAVSSESEFKATDAINRKGPDASKAMVESSIKPNQTRSFRYMATKNVKGKWNKAEEEEDEEEEEALSLCDLPINLEAQPRNQEHKHVNEATQEEFNFRSWSGFFSKEPEMCVADEVFFKGQILPMRLSFSTEAGLLTTGSQHYAKRFINESLDHASLGDFQSNNSSRSSSVRSQNSSSSTSSTTTTTTTTTTAAAPPRFKPSVRNQFHTHPSPKPQLRVSAPRQLVNQGRKSSSAWEIFRLGVVPAPEIGLQDLKVRSSNSANRNCVSRNSSSSSNNSASNSNAKSVKTSNDGNKSNHVLKQLIGKGGGLLSGCDCSFETVQSNMVMIKGGKSTNKAESTTHAVKEKVVELKKQKQRQKQGKKVMSRRRTFEWLKELHASHDPDDEKALLSKPS
CrDHN1	MANYQNQYGAVPTSKDLIQKEMDTTGDTTGVGGGSGSTKVVGDDYDRSSADTTGVSEQHHKKGIIEKIKEKLPGTHHHNK
CrDHN2	MAEENQNKFETTESSEGEIKDRGLFDFLGKKKEEEKPQEEVIVTEFEKVTVSEEKKEDEGEKKHSLLEKLHRSDSSSSSSSEEEGEEGEKKKKKKKEKKGLKEKIEEKIGDDHKHEDTSVPVEKVEVEKVEVEKVEVDPAHTEEKKGFLDKIKEKLPGHKKTEEVPPPPPPPAAASSEHGEGAHEKKGILEKIKEKLPGYHPKTEEEKQKESAGD
CrDHN3	MSEAQLRDQHGNPVQFTDQYGNPVTLTDEHGQPVQLKGVATTGGATTGTAGSGFGTYGTGAYGGGAATGTTTVADLIATERATEMPTAQHHQREPGELRRSSSSSSSSSEDDGHGGRRKKKGLKDKIKEKLPGVGGERKDHAHTPTTTTTTSFGTGATHPPPPTATHPHQHEKKGIMEKIKEKFPGHHTN
CrDHN4	MAEEKLNKSHEYDTTSTNTETEIKDRGVFDFLGKRKEEEKPQEEVLATNFDNKVKLSNEPETKLQEQEGDKKH

	TLLEKLHRSNSSSSSSSDEEEEGEGGEKKKKKKKEKKGLKEKIGGDDHKEQDTAVPVEKVEVDSEHPDTEEKK<br>GFLDKIKEKLPGQHKKAEEVSPTSSEYVAAHTAETHEGEAKDKEKKGLLDKIKEKLPGYHSKPNEDKEKETDT<br>H
CrDHN5	MASYQSQYGDQGRKTDQYGNPVTQTDQYGNPVRQTGETGKQYGTTGYGANTADTGSGHRSGTTGGYGTRTEGV<br>GAGYGTTGGTTTGYGATDYGNTGGTTTGYGGTDYGTTGGTGGTGYGSGTGTTGGTGYGSGTGYGINTGGAHTD<br>AGYGREHRHGHDQSHGGYDASGEQHHEKKGIMDKIKEKLPGTGGHNEK
CrSMP1	MQSAAAVNTDAGIVGRQDISDVAREQGVSVSQTKVGVNRVITESVGGQVVGQFVEPDVPMNSPGMALEPDAIT<br>IGEALEASAVAGAGDKPVDQSDAAAIQAAEMRATGKNETEPGGLGARAQSAATHNTRTPSYFHKTTLSHVLAD<br>AKEKLPTDKHVTREDAEGVIAAELRNKPDMKTTPAGVAASVAAAAAINQNAYLTIIL
CrSMP2	MSQEQPRREEAIKYGDVFNVERELGSKAVAPVDAAMMQKAETAMIGKTEKGGAAATMQAAALKNERDGVVGHN<br>DITEVVAQSGVSVTESDLPGRQVISESVGGQVVHTLEEKERNICKLVEVVEQFGQKVASNTTMPSSLAQDLGS<br>RVGGGITIGEALEATVLTAGKKAVEWSDAAAIQAAEVRATGRTNIVPGGVAAAAQSAATLNARTTKDEDKTKL<br>ADVLSDATSKLPSDRPATRRDAEGVTGAEMRNDPNLTTHPGGVSASVAAAARLNQTNNN
CrSMP3	MSQEQPRHPQPAQDPIKYGDVFDVSGDLAKKPVAPEDAAMMQSAETRVLGQTQAGGAASAMQSAATRNEQAGV<br>VGHRDVTDVTGDRGVTVSETQVPGRRIITESVGGQVVGQYVEPTPVQAGRAGAVRDSAITIGEALEATAQTAG<br>EKPVDKSDASAIQAAEVRATGSNVITPGGLASMAQSAAAFNAECKRDQEKVKLADVLTGATAKLPADKAATIE<br>DAEGVACAEVRNNPDATATPGGVAASVAAAARLNENVNV
CrSMP4	MFTTTIWKFLTISFRSETMSHGQPKRPQAEDSFHCEPIKYGDVFKVTGELASKPISPQEAAAMKAAEAGVLGQ<br>PQKGSPAVVMQAAAAINQACGAVDGNGVAIKKEDVSVTETTTAGNRIITESVGGQVLGKRVDTHAPVVPTDLG<br>SAVIDGDPITIGEALEAVAIAVGDKPVNQNDAAAISAAEIRATGEKNVRAGGVGASAQSAATLNSHVMRVQDM<br>TKLSDILTDATDKLAMDKAVTQEDAEAVYTAEMRFPWRGDATDVISEPGGVAASMATAAKLNEEK
CrASR1	MAEEKHHHHHHLFHHNKDEDKPIETESGYNKTSKYTSNEPSVGYDSGYNKPSYESSGDAYETGYNKTSYDTSG<br>GAYETGYNKTSYDSSGGTYETGYNKTAYSTDEPSAGYGGGRYSDTTGGAYTKKTGEYAAGGGYGDDSDRRDDV<br>DYKKEEKHHKHLEHLGELGAAAAGAYALHEKHKSEKDPEHGHRHKIEEEVAAAAAVGAGGFAFHEHHEKKEAK<br>EQDEEAHGKKHHHLFG
CrASR2	MAEEKHHHHHHHFHHHKNEEQPVETTVYSETTFDVAGNVDDYKKEEKHHKNLEHLGELGAAAAGAYALHEKHK<br>AGKDPEHAHRHKIEEEIAAVAAVGAGGFAFHEHHEKKEAKKQYEETHEKKHHHHLFG
CrASR3	MAEEKHHHLFHHHKDEDKPGYVDEVDYKKEEKHHKHLEHLGELGAAAAGAYALHEKHKAKKEPEHAHKHKIEE<br>EIAAATAVGAGGFVFHEHHEKKEAKKEDEEAHGKKHHHLFG
**CDS**	
CrLEA1-1	ATGCAGGGAGCAAAGAAAGCAGGAGAATCCGCTAAGGAAACTGTTGCCAACTTGGGTGCTTCTGCCAAGTC<br>TGGCATGGAGAAGACCAAGGCCACCGTCCAGGAGAAGACAGAGAGGATGAGGGCACGTGATCCTTTGCAAA<br>AGGATTTGGCAACTCAGAAGAAAGAGGAGAAGATCAACCAGGCGGAGCTGGACAAACAGGCCGCGCGTGAG<br>CACAACGCGGCGGCAAAGCAGTCAGCCACGGCCGGGCACATGGGACAGGGCCACCACACCACCGGAACTGG<br>GACCGGAACCGCCACGTACTCCACCACAGGACAACACGGGCAGCCCATGGGGGCCCATCAGACGTCAGCGA<br>TGCCTGGCCATGGAACTGGGCAGCCCACGGGCCATGTTACTGAAGGAGTCGTGGGCTCACACCCCATTGGA<br>ACCAATAGAGACCCGGATGGGACTGCTACGGCCCATAATACCCGGGCGGGGGGGAATCCAAATGACTATGG<br>GTATGGGACTGGGGGTACTTACACTTAA
CrLEA1-2	ATGCAAGCAGCGAAGCAAGCGGTTGAGAACATTAAGGAAACCGCAGCCAACATTGGCGCCTCTGCTAAGTC<br>TGGCTTGGAGAAGACCAAAGCCAGTATCCAAGAGAAGACTGAGAAGATGAGTGCCCATGATGAAATTCAAA<br>AGGAGATCGCAACCCGAAAGAAAGAAGAGAGGATCAACCAGGCTGAGATAGAGAAACAACAGGCACGTGAG<br>TACAACGCCGCCGCCAAACAATCTGCCATGGCTGGGCACATGAGTCAGGCCCACTATACTAACTCTGGGCC<br>AGGGACTGAAACTGCCACAGATCTCACAATTGGGTCCAGGCCTGAGACTGATGCTACTTACTCCACTACTG<br>GGCCCGGGCCTGAGATTCCTACGACATATTCTACCACTGGGCCTGGAATTGAGACCGCCATATTCCCTGCC<br>ACTGGGTCCGGGCCTGACACTGCAACACACCCCATTGGTGAGTATGCACAACCTATGGGGATAACACCTAT<br>GGGCGTGGTAGGATCAAATCCGTCTGGGACTAACACTGGCGTAGATAGTACTGCTCCTATGAGTGGACATA<br>ATTTCAGTTGA
CrLEA1-3	ATGCAATCTTCAAAGGAGAAGCTGCAAAACGTGGCCGCTGCTGCCAAGGAGCACGTCGACATTTATAAAGC<br>CAAAATAGACGAAAAGGCAGAGAGAGCAACGGCAAGAACCGAGGAGGAGAAAGCAATAGTCCATGAGCGTG<br>CAAAGGCAAAGGAAGCTAAAGCAAAGATGGAGCTGCATGAAGCTAAAGCCAGGCATGCCGCCGAAAAGCTA<br>AGCAACAAACAATCACATTATTATGGTCTCCACGAGCACGAGAGTCCCGTAGTTGGGGCTCAACAACAACC<br>ACCCTTGGTAGGAAATCAGACTCAGTACCAGCAAGGGCACCACTCACTTGGAGCAGTTCCCATGTCTGGAA<br>CCACTTATCCATCTTATCCACTGGGAGGAAACCCTCCCAGCAGTAAGCATATATGA
CrLEA2-1	ATGCATGCTAAGACTGACTCAGAGGTGACAAGCTTGGATGCGTCGTCCACCACACGGTCCCCTCGGAGAGC<br>GGTGTATTACGTTCAAAGTCCATCTCACGATGGGGAGAAAACGACGACGTCGTTGCACTCTACTCCTGTGC<br>TCAGCCCCATGGGATCCCCTCCTCACTCTCACTCTTCTTCAAGTCGATTTTCCGGTTCACGAAAGATGAAC<br>CATCATCGCAACAACAAGACTTGGAAAGACATTGACGTCATCGAAGAAGAAGGCCTTCTTCAATCCGAAGA<br>ACGTGACCGTACTCTCTCTAGAAGATTCTATTTCCTCGCTTTTGTTATTGCCTTCTTCCTTCTCTTCTCTC<br>TCTTTTCCCTTATACTATGGGGTGCTAGCAGGCCCATGAAGCCCAAAATAGTCATCAAGAGCATTAAATTT<br>GATCATCTCAGAGTTCAAGCTGGTTCTGATTCCACCGGAGTCGCCACCGACATGATCACCATGAATTCCAC<br>CCTCAAATTCACGTATCGCAACACCGGCACATTCTTTGGGGTTCATGTCACTTCAACACCATTGGATCTCT<br>CCTATTCAGAAATCGTAATTGCCACCGGCAATATGAACAAATTTTATCAGTCAAGGAAGAGTCAGAGATTG<br>GTGAGTGTTGCAGTGATGGGAAACAAGATCCCGTTGTATGGAAGTGGTGCTAGCTTGAGTAGTTCAACAGG<br>TATGCCAACAGTGCCTGTGCCTTTGAAATTGAGCTTTGTGATTCGATCCAGAGCTTACGTGCTTGGAAGAT<br>TGGTGAAGCCAAAGTACTATAAGAGGGTTGACTGTTCCATCACTTTGGATCCGAAGAAGATCACTCTTCCA<br>CTTTCACTCAAGAAGTCTTGCACATACGATTGA
CrLEA2-2	ATGGCGGATGGACCCCCGGCGACCACCGCCGCCACGAAGCCAAGGAGGCCACGGCCACCGTCTGGACGTAC<br>CAACCTGGCCTCCTGCGTGGTCGCCACCATCTTCCTAATCTTCATCATCATCGTCATCCTCATTGTCTACT<br>ACACCGTCTTCAAGCCACAGGACCCCAAGATCGCCGTCAGCGCCGTTCAGCTCCCATCATTCTCCGTCGTT<br>AACGGCACCGTCAACTTCACCTTCTCCCAGTATGCTTCCGTCAGAAACCCTAACCGCGCCGCTTTCTCTCA<br>CTATGACAGCTCCCTCCAGCTGCTCTACTCCGGCAGCCAGGTCGGGTTCATGTTCATCCCCGCCGGCGAGA<br>TCGACGCCGGTCGGACACAGTACATGGCTGCCACCTTCTCCGTTCAGTCCTTCCCCCTGTCGGCGCCAGCC<br>AGATTGGGGCCCACACTGGCCAATGGCGATGGTGTTGGGTTTAACTATGGGCTTAGGGTGGAGCCCACGAT<br>GGAGATCGAGTCCAAGTTGGAGATGGCGGGGCGCGTCAGGGTTTTGCACTTCTTCAGCCATCACGTTCACG<br>CCAAAGCCGGTTGCAGGGTTGCAATTGCCGTTACCGATGGATCTGTGTTAGGTTTTCATTGCTAA
CrLEA2-3	ATGGCTCAGAGTCCAGAGCAGGTTAAGCCCTTAGCTCCATTCATATCATCAAATTACCTCAGCAGACAAGA

	AGATCAAAACATCTCCGAAAGAAAAATCATTCGCATAAGAAAGTTCGTCCGTTGCTGCGGCTGTGTCACTG
	CCCTTGTTTTGATATTTGTAGTCATAATATTAGTCTTGGGCTTCACAGTTTACAATGTGAAAGAACCTGAA
	GTAAGGATGAACAGGGTTATCCTTCTCAATAGAACTTTAGCTAACGGTGCTATCAATGACGTTACACTTCT
	TGCTGATATCTCTTTGAAGAATTCAAACGCTTTCACCTTCAGGTTTGGATACACCAACACCACTGTTTACT
	ATGATGGCACGGAAATAGGTGAGGGAACATCTCCACCGGGTAAGGCCAAGGCAAGAAGGACAATAAGGTTC
	AACGTGACGATGGAAATTATGGCAAAGAAGCTTTTGGCTGTCCCAAGCTCGGAGAGTGACATCAGAGATGA
	TCAAGCTTTGAATATCAGCAGCTATACCATGGTAGATGGTAAGGTCAAAATATACAATCTGTTTATGAAGA
	AGGTTGTGGTTGAATTGAATTGTACAATTCAATACAACATCACCACTGGTTTGACCTCCTCAAACGGTGGC
	AATTGCCTCGGACTCGTTGATCTTTAG
	ATGGCCACCAATATGCCTAAATTCAAGCGAAAACTGGGAATGGGTGCAGATGGCCGCACCAACCCCATAGT
	CTGGCTAATAGCCATTATCTGCACCATCATAGCCGTAGCAGTGGTAGTTGCAGGCATTGCAGTGTTTATAG
	GTTACATAGTGATCCACCCCAGGGTTCCTATCATCAGTGTCACAGATGCTCATTTAGATTTTCTGAGAAAT
	GATTATGCTGGTCGACTCCAAACCCAAATCAGAATTGTTGTGACTGCAAAGAATGGGAATGCCAAGGCTCA
	TGCAACATTTTCTAAGATGACTTTCAACATTAGCTACCAAGGCCAAGATATAGCTGTGCTTGTTGCAGACC
	CATTTGAGGTGCCCAAGAACAGTTCCAAAGAGCTCCATTATGTTGTACAATCATCCTCCATACCATTAAAT
	CCTGATCAGATGGAGGAAGTGACTAATGCATGGAAACAGAATGAGATTGAGTTTGATTTCAAAGGAGCTGC
	AAGGACACAGTGGAGGGTAGGGCCTTTAGGCTCTGTTAAATTCTTGTGTCATCTGGATTGTGACCTCAAGT
CrLEA2-4	TTCGCCCCTTGAATGGGTCTTACGTACCCTCGCATTGCACCTCCAAATCAAAATGA
	ATGATGAGTGGAAGAGAGAACGTAGTTAGAGTTTCAGAGGAGGGTGAAGCAACAAGTAGGCAGCAGCAACT
	GTACTACGCGGCAACGCCACCATCGTCTCATTCCTCCACCTGTAAAGGGTGCTGCTGCTGCCTCTTCCTCC
	TCTTCTCTTTCCTCGGTCTCTTAGTCCTAGCAGCACTGCTACTCATCGTGCTGGTCGTTAAGCCCAGGAAC
	CCTCACTTGGACCTTTCCCAAGTGGGCTTCCAGTATATGGCCATCAGGCCCAACCCCAACGATCCAGCAAC
	AGCCAGTCTCTACCTTATCATCAGGTTGGTGTTGTCGGTGGTGAATCCCAACAGGGTTAGGATCAGGTACG
	GGGAGTCCAGGTTAAGCCTCATGTTCCGCGGTGTGACGTTGGGGCGGGCCTCGGTGCCAGCTTTCTTCCAG
	CGTGCTCACAGCGTGACGGAGGTGGTGGCCACCGTCGCCGTCGATGGGGTCAACTTATCTGAGGCTGATGC
	TGCTGACTTCACCAGAGACGCTTTGTTGAATGACCGGGTCGAGCTTCGGGTCTTGGGCCATGTTGCCGCCA
	AGATTCGCCTCTTCAACCTCTATTCTCCTCCTCTTCAGGTGTCGGTGAATTGTGTTATAGTGATAAGTCCA
CrLEA2-5	AGAAAGCAATCTCTTACTTATAAGCAGTGTGGATTGGAGGGATTGAATGTTTGA
	ATGACCGAACCATCATCCAAACCCAACGGAAACGGCGCAATTAACGGTGCTGCCACCACTACTAACGGCAA
	CCCTGGTCCCGTTAAGTCCCAACTCTACAACCCAAACCGTCAACTTTACCGACCACAATCCCACTACCACC
	GTCGTCAACGCTCTCACCGAAACCTCTGCTGCTGCTGCTGCTTCTGGACCATTCTCACCCTCCTCGCCGTC
	GCTCTCCTCGCCGCAATTGTCGGCGCCGCACTGTACGTACTGTACCGCCCTCACCGTCCCGAATTCTCCGT
	CACAAACCTCCGCATTGCGAAGATGAACCTCACAACCTCGAGCGACTCACCCTCCCACCTCACCACGCTCT
	TCAACCTCACACTCATCTCCAAGAATCCGAACAACCACCTCGTCTTCTTCTACGATCCCTTCACCGTCAAC
	GTCTTCTCTAACTCCGTCCCCGTCGGAAACGGTTCCCTAACGGCGTTTACTTCGGACAAGAACAACCAGAC
	TAGTATTCGCACGGTGCTGTCGGGCTCCCAAGATCTGGACACGGAGTCGTTGACCAGCCTGAGATCGGGTC
	TGAAGAAGAAGGGTTTCCCCGTGGAGATTCAGATGGACACTAAGGTGAAGATGAAGATGGATTGGCTCAAG
	AGCAAGAAGGTCGGGATTAGAGTCACCTGTGATGGAATCAAAGGCACCGTTCCCGCCGGTAAGACGCCGGC
CrLEA2-6	GGTTGCTTCCGTTATCAACTCTGACTGTAAGGTCGATCTTCGAATCAAGATCTGGAAGTTCTCTTTCTAG
	ATGGAGGAGCGGGTGTCGTCGCAGCTATCGCCGCCGCCACCAGTAGTACAATCTCCTCCTCAAGACCCATC
	TATACAGAACCATAGTAGCAAAGCACTCCAATTATCCCCGAACTTGGACCCCGGCACATACGTTGTCCGGG
	TTCCAAAGGACCAAGTCTACCGTGTTCCACCGCCGGAAAATGCCGAAATCGCGGAGCTCCATCGGAGCACT
	CCCCAAAAGAATACAAAGAGGACGCGTTGCTGGTGTGTCACGTGTTTCATTGCTGTTATAATTGTCATTTT
	GATCATAATTGGAGGCATTTTGGGTGGGCTTTTCTCTGTTGTACTTAAACCAAAAGACCCAAGGTTTTCGA
	TCCAACACTTTAATGTCCTAAAGGGCACTCCCAAACCCAAATACAACATCACTTTACAAGCCCACAATCCA
	AATTCTAAAGTAGGCATTTGGTACAAAGAAGGAGGCCACGTTTCATTGTCTTTAAGGCCGAAAGAGATTGC
	TTCAGGATCCTACCCAAGTTTTCATCAAACCCCTCATGATTCAACACCCTTTGGCGTTACTTTAAAAGCCT
	CAAAAGCTGGTTTCCCTAAGGAGGTGGAGGAAAGCATCACAAATCATAAACACAAAGTGCATGTCGCATTC
	TCATTAACCATTCACGTCCCAGCACGAATGAAAATGGGATTGTTGCGTAGTGGGACAATGCAATTCGACGT
	TACGTGTCAGGTGACGTTGGATTCCTTGGCCAAAACCCCTCGTGTACTTTCTCAACAATGTCAAACTAAAC
CrLEA2-7	GACACTAG
	ATGTCTCAAATCACAATAAAATCTCCAAAACACTGTGCTACCAAGCAAGGACTGAAAATTGAGAGGAACTA
	CAAGAAACTCTTCTATGTTTTTTCAGCATTTTTCACCTCAATCCTATTGTTAATACTTCTTATCTGGCTCA
	TCCTTCACCCTTCCAAGCCTCAATTCTCCCTCAAACAAGTTGACATCTACCAGCTGAACTTGTCAGGACCA
	AACCTCAACTCCTCCATCCAACTCACTCTTCTATCCAAGAATCCAAACCAGAAAGTTGGTATTTACTATGA
	TGAATTTCAAGTTTATGCAACCTATAAGGGTCAGCAGATAACTGGTGACACCCCTGTGCCTCCCTTTTACC
	AAGGACAAGAAGAGAATAATCTCTTAACAGCCTCTTTGGTAGGAAATGGGTTACCAGTGGCTCCTTCTCTT
	GGTTATGAAGTGGGTCGTGATCAAACTGTTGGAAGACTAGTTTTGAATGTCAAAGTTAATGGAAAGCTTCG
	TTGGAAGGTGGCAACTTGGGTCTCTGGACGTTACAGGTTCAATGTTAATTGTGTTTCAATCATTGCCTTTG
CrLEA2-8	GACCTTCAGCTCCTCTCACTTCAAAGCAGGGAGCTCGGTGTTCTACCACAATTTAA
	ATGAAAGCAGGATCTGGTAAAGGGAGAAAAGTTTGCCTGACAGTGACAGGTGTTGTGATTGCAATTGTAAT
	GCTAATGGTGATACTAGCATTGACGGTGTTCAAAGCCAAGCATCCTGTTACCACAGTGGACTCAGTGATGC
	TGCAGGACTTCCACATGAGCTTGGATATAGCAAAACTAAAGGTAGATTTGAATGTGACCCTGGACGTGGAT
	GTCTCTGTGAAGAACCCAAATAAGGTGGGATTCAAGTACTCAGACAGCACCGCCCACCTCAATTACAGAGG
	GCAGCTGATAGGTGAAGTCCCAATCTCAGCTGGAGAGATTTCTTCTGGTGAGACCAAAGGATTCAACCTCA
	CCCTCACCATTATGGCCGACCGTTTGATCTCCAATGCCCAGCTTTACTCTGATTTCACATCTGGTACATTG
	CCCCTAAACACTTTCATTAGGATTTCTGGCAAAGTCAGCATCTTAGGCTTTATCAAAGTCCATGTGGTTTC
	CTCCACATCTTGTGATTTTGCGGTTAATCTTTCCAATAGAACAGTTGGAAACCAAGAGTGCCAATACAAGA
CrLEA2-9	CGAAACTTTGA
	ATGATCTTATCGACACGTTTGAGACCGTTGATTAGTGGCAACAGAGAGGTCACGTGCGACACGTATCATCA
	CGTGTCGGCATGGGACGAGACGTGTGGTATACGTGTCTGGAGCAGGAACCTCTTGCTGAATAAAGCCAAGG
	ACTTCGTGTCCGAGAAGATCAACAACGTGGCCAAGCCTGAGGCGAGTGTCACCGACGTCGACTTCAAGCGC
	GTGAGCAGAGATAGCATCGAATACTTGGCCAAGGTCTCTGTTCGCAATCCTTATTCAACTCCAATACCAAT
	CTGTGAGATCAAGTACTCTTTCAAAAGCGCGTCAAGGGAGATAGCATCAGGCAGAATGCCAGACCCAGGAT
	CGCTGAAGGCAAAGGATACGACAATGGTGGAGGTACCAGTGAAGGTGCCATATAGCATATTAATGAGTTTA
CrLEA2-10	GCTAAGGATATTGGAGCTGATTGGGATATTGACTACCAACTGGATCTGGGTCTCGTTATTGACCTTCCTGC

	CATTGGCAATTTCACCATTCCTCTCTCTCACAACGGAGAGGTCAAGCTACCAACCCTCTCTACTATGTTCG<br>CTTAG
CrLEA2-11	ATGGCAGAGAGAGAGCAGGTTAGACCCTTAGCTCCATCCATAGATCGTCCAAGCAGCGACGAGGACGACAC<br>CACTCTACACCGCACATCACCTGCTCGTACAAAATTCATCAAATGGTGGGCTTGTCCATTAGCTTTCTTAC<br>TTCTTCTAGCAATTATAATCATAGTTTTAATATTCACCGTGTTTCATGTCAAGGACCCTGATATCAGAATG<br>AACGGTGTCAAAATCACAAAGCTAGAACTTGTCAACACCTTAACCCCTAAACCTGGGGCTAACATGTCCTT<br>AATTGCAGATGTATCGGTGAAAAACCCTAACGTTGCATCCTTTAGGTATAGTAACACCACCACAAGTTTGT<br>TCTATCATGGCATCATGGTGGGAGAGGCTAGAGGACCCCCAGGGAGGGCCAAGGCTAAACGAACAATTAGG<br>ATGAATGTTTCAATTGATGTCATCACGGATCGTATTGTGTCTAATCCTGATTTAGGACAGATTTCGTTTC<br>AGGGTTGTTGACTATGACCAGCTTCTCCAGAGTTCCAGGGCAGGTTAAGATCTTGAACTTGTTCAAGAAAC<br>ATATTGTGGTCAAAATGAATTGTACCACCACTTTCAATATTTCTACACAGGGAATTCAAGAACAGAGTTGT<br>AAGCGCAAGCAAAAGACAACTTGCTCTACAATCTCTCCATTTGGGGCTACCAATTCTTTGAAAGTTATTAT<br>TTCATATGGCACTAAGCAAGATTTGCGTGTAATCTGA
CrLEA2-12	ATGCAAATACACGATCAAGAAGAACCAACCCAACAACACTCACAAAACCAACCTCATCATTTCCGTGTCAA<br>AGTGCCTCAACAACAACACTACAACATGGACAAAGGTATCCCTCCTCGCTTCAAACCCAATGCCCCAAAAC<br>GTGAACACTGCATTTGCATCACAGTTTTTCTCCTTCTACTTGGCATCATTATCCTCATCCTCTGGCTCGCT<br>TACCACCCTTCCAAGCCACGCTTCACGGTGGCCAGCGCCGCCATCTATGGCGTCAACACCACATCACCGCC<br>ACTCATGTCCATCACAATGCAATTCAATATCCTCATTAGGAACCCAAACAAGCGTGTCTCAATCTACTTTG<br>ACAGACTCTCTGCTTACGTGTCCTACAGAAACCAACCAATCACGCCACGTGTCATGCTGCCACCACTCTTC<br>CTCGAGAAACACAGCGCCGTCTCGCTGTCGCCGGAGATCGGAGGCGTGCCGGTGCCGGTGTCGGAGGAGGT<br>CTCAAATGGATTGGCAATGGATGAGAGTTATGGGGTGGTGGCTGTGAAGCTTATGTTTCTTGGAAGGTTAA<br>GGTGGAAAACTGGTGACATAAACTCTGCACATTATGGATTGTATGTCAAATGTGACGTGTTAATGGGTTTG<br>AGGAAAGGTTTTGTGGGTCAGGTTCCTCTTCTCGGAGCTCCGGTTTGTGAAGTGAATACTTGA
CrLEA2-13	ATGACTGATAGAGTCCATCCCTCCGCCAACGGCGGAAAAACCGCCACATTTCCCGCCACAAAATCTCAACT<br>TTACGGCGCCACCCGTCCCTCCTACTGCCCCCAACCGTATCACCAACGCCGTAGCAGCAGTCGGGGATGGT<br>GTTGCAGCCTCTGCCTCTGCCTCATCCTCATCCTCCTCTTCCTCCTCCTCCTCATCGGCGGCGCCGGCACG<br>GTCGCGTACTTCCTCTACCACCCTCAACGACCTTCCTTCTCCGTCACCTCTCTGAAACTCTCGTACCTGAA<br>ACTCACCTCTTCCTCGACTCTTAATTCCAAGTTTGAGCTCAAACTCTCAGCCACAAACCCTAACAACAAAA<br>TTGTCTTCTCCTACGACCCCACATCCGTCTCCATCCTCTCCAACGAAGACATCGACATCGCACACGGCACC<br>ATTCCCTCCTTCCGCCACTCCCAGAGGAACATCACGATCCTCAAAGTTTCCATTGCGAGCAGCGAGGAAAG<br>CGTGGAGAGCGACGCCGCGATGCGGCTGAAGGGGATCATGAAGAGCAATAGCGGGTTGGCGCTGAAGGTGA<br>AATTGGAGACGAAGGTGCAGGCCAATATGGGTGTGCTTCACACCCCAAGTGTCCCCGTTACTGCTTTCTGT<br>GATGGCGTCGCCGTCACACTTCCCGACGGAGATAAACCGGCGACGGCGTCCATCGCGAACACGGAGTGCAA<br>CGTGGATGTGAGGTTCAAGATATGGAAATGGACCGTTGGATGA
CrLEA2-14	ATGTATGAGAAACAACCCCAATTGAACGGAGCCTACTATGGCCCTTCAATTCCGCCACCAAAATCTTACCA<br>TCGCCCAGGGCGCGGCGGCGGCTGCGACTGCTGCTGTGGCTGTATCTTCAGCCTGATCTTCAAACTGATCC<br>TGACGGTGATCATCATCGTTGGAATCGCCGTCTTCCTGTTCTGGCTCATCGTTCGACCCAACGTGGTGAAG<br>GTTCACGTGACGGAGGCGACCCTGACGGAATTCAACTACACGGGGAACACCCTATACTATGACTTGGCTCT<br>GAACATGACAATTCGCAACCCCAACAAGAGGCTGGGTATCTACTATGATCGCATCGAAGCCCGTGCAATTT<br>TCCAGGATGCAAGGTTGGACTCCAAATTCCTGGAGCCCTTCTATATGGGCCACAAGACCACCAAGCTTTTG<br>AATCCTGCGTTCAAGGGTCACCAGGTGGTTCCACTCAACACTGACCAAACTGCGGAATTGAAGAAGGAGAA<br>CAGCACCGGGATTTACGAGATTGATGTGAAGCTGTACCTCAGGGTGAGATTCAAGTTGGGAGTGTTCAAGA<br>CCAAGACGCTCAAGCCTAAGGTTACCTGTGACTTACGCGTGCCTTTCACTTCACCTAATGGACCCTCGCCA<br>GCGCTCGGTGCTTTCCAGACTACCAAGTGCGATTGGGATCGTTGA
CrLEA2-15	ATGGCCGATAAGCAACCTCAGCTGAACGGTGCGTTTTACGGTCCTGCCATTCCCCCACCAGCGCAGCAACC<br>ACGCTACCATCGCCACCACCGGGGCCGAAGCTGCTGTTGCTGCCTCTTCGGAATCTTCTGGAAGATTCTGG<br>TTGCGCTCATTGTCCTGGCAGGCCTGGCAGGCCTCATCTTCTACCTGGTGGTTCAACCCCGCTCCTTCAAG<br>TTCTACGTCACGGAAGCCAACCTTAACCAATTCGAATACCACAGCAACAACAACACCCTTAACTACGACAT<br>GGTACTCAACTTCACTGCCCGCAACCCCAACAAAAAGCTGAGCATCTACTACGACAAAGTGGAGGCTTTAA<br>CATTCTACGAAGGAGCCAGGTTCAGCAATTACGATGTCATCACGCACTTCAACTCATTCCGGCAGTATAAG<br>AAGAGCAGCAGCCCCATGAGCGGCGTTTTCAAAGGGAACAGAGTGGTGGTGCTGGACAATGACCAGCTTTC<br>TGAGTTAAACAGAGACCAGAATGAAAGGGTTTATGATATCTATGTGAGGCTGTACTTCAGGATTAGGTTCA<br>GACTCGGTGACGTCATATCCAGGGACTTCAAGCCCAAGGTCAAATGTCACCTCAAAGTTCCCTTAGCTTCT<br>ACCACCACAACTCTGTTTCAGCCCACCAAGTGCGACGTCGATTTCTAG
CrLEA2-16	ATGTCGGTGAAGGAGTGTGACCACCACAAGGGGAAGAAGCACAAAATCTTCCGGCGCATCTTCTGGGGCAT<br>AGTGATCTTCGTGTTTATCGTGCTGGTAACAATTCTGATAATATGGGCAATCCTAAAGCCTACGAAGCCAA<br>CCTTCATCCTCCAAGACGTCACCGTTTACGCCTTCAACGCAAGCATCCCCAACCTCCTCACCTCCAATTTC<br>CAGGTGACACTCTCCTCTCGTAATCCCAACGACAAAATCGGCATCTACTATGACCGTCTCAATACATATGT<br>CACTTACCGGAACCAGCAGGTCACCTACCGCACTTCCATTCCTCCCTCTTACCAAGGTCACAAGGAAGAGG<br>ACGTCTGGTCACCGTTTGTTTACGGTACTAATGTCCCCGTTGCTCCCTATAATTTCGCTGGCCTCAGTCAA<br>GACCAGTCTAACGGTAACGTCCTCGTTATCGTTAAAATTGACGGCAAGGTCCGTTGGAAGGTCGGTGCCTT<br>TATCTCCGGCCATTACAACCTCTATGTACGCTGCCCTGCTTTCATCACATTTGGCCCTCGGAGTAACGGAA<br>TTGCCATTGGCGATAACGCTGTTAAGTATCAATTGGTTCAGCGTTGCACCGTTGGCGTTTGA
CrLEA2-17	ATGGAAAAGGACAAAATTTCCGGTTCACCCAAACGTGCAGTGTGCACATGCATCACAATCTTCCTTCTGTT<br>AGTCGCCATCACTCTCCTCGTCCTCTGGCTCGTCTACCGTCCCCACAAGCCTCGCTTCGTGGTGGTCGGCG<br>CCGCCATCTACGGCCTCAACACAACCACCCCTCCCCTCATGTCTGCCACCATGCAGCTCACCGTCCTCATT<br>AGGAACCCAAACAGGCGCGTCTCCGTTTACTATGACAGATTCTCCGCCTTCGTTTCCTACAGGAACCAAGC<br>CATAACGCCTCAGGTTGCGCTGCCACCGCTGCACCAGGACAAGCACAGCACGGTGTCACTATCGCCGGTGA<br>TCGGAGGCACGGCGGTTCCGGTGTCGGTGGAGGTGTCAAATGGGTTGATGGTGGACGAGGCTTATGGGGTG<br>GTGGGTCTGAAGCTGACTTTTCAGGGAAGGGTGAGGTGGAAGGCTGGAGCCATCAAAACTGCACACTACGG<br>ACTGTATGTCAACTGCAACTTATTGATGGGTTTGAAGAAAGGTTTTGTTGGTCCAGTTCCTCTCCTCGGAG<br>CTCAACCTTGCGATGTCGATTTATGA
CrLEA2-18	ATGGATGTTTCCGCTTCTAATTCCGATTGCTTCTGGTGGAAAGAACTTCATCCCGGATTTTCTTTTAAAAA<br>ATGCTGCTTCTTTCTGCTTATCTTCCTTTTGATCACATGCATGGTGGTTGGATTAGCCACTTTTTTTGTGA<br>TCCTCATCGTTAGACCCCACAAACCGGTTTTCTCTGTTAGAGAGGTTCAAATAAATTTTTACAAGATTGAT<br>GATCGTTCTAATCTGACACTGTTGGTTTCATCTGTAATCTCCTTAACCCTCAATGCAGAAAATCATAATAA

	GTTTGGTATAGGTTTTAGTCCATCAAGGTTCCTTGTTTATCAAGAAGGCTTGCATATAGGAACTATTCGAG
	TTCCATGGTTTTTTCAACCTCCTCACACTGAAAATGTGACCGTACCATCTCGTGTCTTATTGCAATGTGTT
	AATCTCAGCAAAATTGTCGCCAACACATCCTTACAAGAAATGTCAAAGCAAAACACGGCGCCGATCAAGAT
	CACAGGGGATGCCAGAGTCCACGTGTGGGTACTTCATATAAAACTTTTTGAGATTAAGATTGCACTAGATT
	GTGGTATGAATTTTAATATAAGAGAGTTTGCTTTCACAAATGAAGCGTTTGGTGCCAAAATTAGCAAGAGT
	GACTTGGGTTCAGTTCTCAGTGATTCAAAAGGCATCTCTATGAAATGTGCTTCATCTGTCTACATATAA
	ATGTCTGCATTAGCACCAAAACAAGAAATCATGACAGAAGCACAAAATAATGAGGATCAAGAGCAAGTGGT
	GGTGATTTCACAAAAAAAGCTCAAGAGAAGAAAAGTGTGTGTAATTACCACAGGAGTAGTGTTGTTACTTC
	TTGTGGTGCTAATCATAGTGGGGCTTGTCCTTGTTTTGAACTTGCTCAAGCCTAAAGATCCAAGGGCAGAC
	CTTGTTTCTGCCACTTTGGAGGGTATAGCACCTCGTGTCACACTTCCTGCTATTGACATACAAATCAATGT
	TACTCTTGATCTCAAGATTCAAGTTGAGAACAGAAACCATGTTAGTTTCAAGCATCAAGGAGGGAAGAGTG
	TTTTGTTATATGGAGGAAAAGAGGTTGGAGAGACTCAGTTATACCCTGGTGTGATTCCTTCAAAGGGTTCT
	ACTATACTTCCTTGTAGACTCACCCTTCAGGCAGAGAAACTTGCTTCCAATTTTTCTGGTTTGGTTGAGGA
	CTTGATGGGAGGACAAATCTCCATGGGAGCTGTTACTAGGATTCCTGGGAGAGTTACCTTCCTTGGATTCA
	TAAAAAAACATATTATTGCTAAGTCCAATTGCCAATTCACTGTTAGTGTTTCTGACTTCAAGATCATTAGC
CrLEA2-19	CAAGCTTGCAAGAATAAGGCCAAACTATGA
	ATGATAGAAAAATATGGGCACCGCAAAAAGAGTAACATGTGCCTGGTGGTAACATGTATTGCAGTGATAGC
	AATTCTGGCGGTAGCAACGATCTTAGTATCAACGATGTTAAAACCAAGGCAACCTATAACCAAAGTGGAAG
	GCATAAGGCTTGATGACATGAATATGAACATGAATATGTTTAAAATGAGGATGGATATTAACGTGACATTG
	AAAGTGGATATGTCGGTGGAGAACACAAATAAGTTTGGATTTGTGTACAATGATAGCCTTGCTCAACTCAA
	CTATGGAGGGGAATTAATTGGGGAAGCCCCAATACCCAATGGAGAGATATTATCTGAGGAAACTAAAGGAA
	TGAACTTGACACTAACCATTATGGCTGATCGCATGTTCTCCAATTCTCAAGCTTTCAAAGATGTTACAACG
	GGTTCATTGCCCCTCAACACTATTGTTAAACTTTCTGGCCAAGTCATCATCTTGGGCCTCATCAATTTTCA
	TGTGGGTTCCACCTTATCTTGTGATTTCAACCTCAATGTTTCCAATAGAACCATTGACCACAAAGAGTGTC
CrLEA2-20	ACCATGACACAAAGATTTGA
	ATGAGAGTGGGTTTGGGTGTGAAGGTGTCGTTGGGTGTGATCGGTGTGAGTCTGATATTGGCAGCGTTTAA
	AATGTCGGAGTTGTTGGACAAAGCCAAAAACTATGTTTCAGAGAAAGTGGCCAACATGCCCAAACCTGAGG
	CCGCTGTCGCTGACGTGGATTTCAAGCGCGTGAGTCGAGAGGGCGTCGAGTACTTGGCCAAGGTCTCTGTT
	TCCAATCCTTATCCCTCTCCCATTCCAATCTGTGAGATCAAGTACTCCCTCAAAAGTGCTGGCAGGGAGAT
	AGCATCAGGGACAATACCAGATCCAGGGTCGTTGAAGGCAAAAGACACAACAATGCTAGATGTGCCAGTGA
	AGGTGCCTCATAGCATATTGTTAAGCTTGGCAAGGGACATTGGTAGAGATTGGGACATAGACTATCAATTG
	GATCTTGTCTTAATTATTGACCTTCCTGTGGTTGGCAACTTCAGTATTCCTCTTTCTCAGCAGGGAGAGAT
CrLEA2-21	CAAACTCCCCACCATCTCTGATATGTTTACCTAA
	ATGTGCAAAACCGTAGGCAGATTATTGGCCTTACTCTTGGTCATTATCCCCGTAACGGTGACCGTTACGGC
	CTTTCTTTTTTGTCTCATTGTGCGACCCAACGTCGTTAAATTTCACGTGACCGATGCTACCCTCGCCCAAT
	TCAGTTACACTAATAACACCCTTTACTACGATCTCACCCTCATCGTGGAAGTTCGCAACCCCAACACAAAG
	ATCAGAATCCACTATAACTACATGGAAGAAGTTGTGTTATACCAAAATGTGAAGTTCTGTTCGCTGATCTT
	AGGAACCTACATTCAGCCACACAAGAACACTGCCATGTTGACTCCTGTTTTTAAAGGTCAACAAGTCATGT
	CCCTCAACAAAGACCACATTTTGGAGACTTACAATAATGAGAAGCGTTCTGGGATTTACCACATCGATTTT
	AAACTTTACTTGAAGGTTAAGTTCGATTTGGGTGTGTACAAAACTAACAATATGAAGTCCACGGTGACTTG
	TAACCTACAACTCCCTCTTCAATCTTACAATGGAACCTCAACCGCTGGTAGGTTTCAACTTACCCGCTGCA
CrLEA2-22	ACTTCGATTACAAACATATTTTGTTTATTTTCTGA
	ATGGCTGATTATAAGCAACCCCAACTAAACGGTGCGTATTATGGCCCTTCCATTCCTCCAGCGGAGCCACC
	ACGCCAACGCGCTCACCGCGGGAGAAGATGCTGTTGCTGCCTCTTCAGCTTCTTCTGGAAGTTTCTCCTCG
	CAATCGCTGTCCTCATTGGCCTCGCCATCCTCATCTTCTGGCTCTTAGTTCAACCACGCGCCTTCAAGTTG
	CACGTGACGGAAGCGAGTCTCACTCAATTCAACTACACCTCCAACACTCTCCAGTACAATCTCGTCCTCAA
	CTTCACGGCCCGAAACCCTAACAAAAAACTCAACATCTACTATGACAAAGTGGAGGGTCACGTGTTCTACG
	ACAGCGTCAGGTTTGCTTCAACGGACGTTATCACGTGGCAAAACTCGTTCCGTCAATACACGAAGAGCACG
	AACCGCATGAGCGGCGTGTTCTCGGGGCAACGCGTGATTGTGCTTGATCATAATCGGGCCTCAGATTTGGA
	AGAAGATAAGAGAAGTGGGATTTTTCACATTGATGTGAGGCTCTACTTCACCATTAGGTTCAGGCTTGGTG
	ACTTCATATGGAATAGTGATATCAAGCCCAAAGCCAAGTGTGGGCTTAAGATTCCTTTCAGTTCGAATGGG
CrLEA2-23	ACTACGGTGAATGAGTTTCGGCCCACCAAGTGCGATGTTGATTTCTGA
	ATGTCGGTGAAGGAGTGCGATCTTCACAAGGGGAAGAAGAGGAAGATCTTCCTGCGAATATTCTGGGGAAT
	AATTGTCTTCCTTTTCATCGTGCTCGTTACAATCCTTATAATTTGGGCAATTCTAAGACCCTCAAAACCTT
	CCTTCATTCTCCAAGACGTCACCGTTTATGCCTTCAACGCCACCGTTCCCAATCTGTTAACCTCAAATTTC
	CAAGTCACACTGTCTTCACGGAACCCAAACGATCACATAGGCGTCTATTACGACCGTTTGGATACTTATGT
	CACTTACCAGAGCCAGCAAATTACTTACCGCACCGCAATTCCTCCCTCCTATCAAGGCCACAAAGAGATCG
	ACGTTTGGTCGCCGTTTGTTTACGGCACCAACGTCCCCGTCGCGCCTTTCAATTACGCCGGTCTTAGTCAG
	GATCAGTCCAACGGTAACATCCTCGTCACCATTAAAATTGACGGCAGAGTTCGTTGGAAGGTTGGCGCCTT
	CATCTCTGGCCGTTACCACCTCTACGTCCGGTGCCCTGCTTTCATCAATTTTGGGACCCGGAATAACGGAA
CrLEA2-24	TTTCCGTTGGTGAAAACGGCGCCATTAAGTACCAGCTTGTTCAGTCATGCTCCGTTAGCGTTTGA
	ATGGCAGATCATCAGAGACAAAGGATCCACCCTATGGAGGTAGGAGCACCACCACCACCAACCATCCCATT
	GGTACCTCCAGGTTCCTCAAGATCAGAAAAGGGTAACCCTTTACACCGTCCACCACAACTACATGCTAGGC
	CAGCAATATACCCTTCACCAAAAAAAGGAAACTGCTTTTGCAAGTGCATATGTTGGACTCTATGCTTGGTT
	TTCCTCCTGCTCATTATCTTGGCAGCAACTGTTGGAATCCTTTACCTAGTTTTCAAACCAAAACTTCCCAA
	TTACTCAGTTGAAACACTGAGGATAAGTGATCTTAGGCTCAACTTTGATTTGAGTCTGTATGCAAAGTTTG
	ATGTGAAGATCACAGCAAATAACCCAAATAAAAAGATTGGTATTTACTATGAGGAAGGTGGGAAATTGAGT
	GTGTGGTACAGAAAGACAAGACTTTGTGAAGGGTCATTACCAAAGTTCTACCAGGGTCATCAGAACAAAAC
	AGTGCTGAATGTGTCCTTGACTGGTCAAGTGCAATCTGGAAGCACCTTAATGACGGCACTGCAGCAACAAC
	AGCAAACAGGACGCATTCCATTGGATCTCAAAGTGCATGCACCAGTTGCCATCAAACTTGGGAGGTTCAAG
	CTGAGGAAGGTCAGAGTCTTGGGTGAATGCTTGTTGGTTGTGGATAGCTTATCATCTAATAATCTCATAAC
CrLEA2-25	CATCAAGGCTAGCAACTGCAAATTCTCAATGAAACTTTAA
	ATGCGCAGCAATGACCACATACCCGTTCATCATGTCGCGGGACCGAACCCGAAACCCATGAAACGACACCG
	TACCGCTCGGTACTACGTTCATCGGGTCCATGAAAGCCTCACAACCCGCGTGTCCAAGATAATCTGCGCCA
	CTTTCTTGGGCCTACTTGCCATTGTGGGCCTCATCACCTTCATCCTCTGGCTCAGCCTCCGGCCCCACCGG
CrLEA2-26	CCCAGGTTTCATATCCATGAATTCAACATGCCGGGCCTGACCCAACAATCCGGGTTCGAAAATGCCCGGAT

	AACCTTCAATGTAACTGCCCGAAACTCCAACCAGAACATCGGGGTTTACTACGAGTCCATGGATGGAGCGG
	TTTATTACCGGGACCAGAAAATCGGGTCGACATCGTTACTTAATCCGTTTTATCAAGAGCCCAAGAACACG
	ACGGAGGTGGACGGTGATCTCAGTGGTGCCACGTTGACCGTTAGTAGTCAACATTGGGCAGAATTCCAAAG
	CGATAGGGCTGATGGTAGCGTGGTGTTCCGCTTGGAAGTGACGTCTGTGATCAGATTCAAGATATCAACGT
	GGGACAGCAAGCGCCACACGATGCACGCTAACTGTGATGTGGGCGTGGGACCCGATGGTTCCCTCTTGTCC
	AATTACAAAGACAAGAGGTGCCCCGTTTATTTCTCTTGA
	ATGAAACCCAATCACAACATAACAATGCTCGCCAAGACCGATTCCGAGGTTAGTAGCCTCAGTCAGTCGTC
	CCCGGCGAGGTCTCCCCGACGGGCCGTCTACTATGTGCAAAGCCCATCACGTGACTCCTCCAACGACGGCG
	AGAAGACAACCAATTCATTCCAGTCCAGCCCGCTGCAAAGCCCATTGGGCTCCCCTCCCCACTCCCACTCC
	AATTCATCTTTAGGCCCGCATTCTCGCGAATCTGCCTCCACCCGCTTCTCGGGCTCCCGAAAGACCAACGA
	GTCCAACCGCAAGGGCCCATGGAGGCCCTGGAAGGATCAGTTCCACGCCATCGAAGAGGAAGGCCTCCTCG
	ATGCCCATGACGCTGCACAACATGGCTTCCCTCGTCGCTGCTATTTTCTCGCATTTGTTGTGGGCTTTGTA
	GTGCTCTTCTCTGTTTTCTCCCTCATTCTGTGGGGCGCAAGCCGCCCCCAGAAACCCGCCATTACGCTTAA
	GAGTATTACCTTTGATCAATTTGTGATTCAAGCGGGTGCGGATGTGTCAGGAGTGGCCACAAGCTTGGTAT
	CCATGAATTCCTCAGTGAAACTAACCTTCCGTAACACGGCTACATTTTTCGGAGTCCATGTGACATCAACT
	CCTCTAAATCTCAATTATTATCAGCTTACTCTGGCCACCGGAAGTATGCCAAAGTTTTATCAATCTAGGAA
	AAGCCAGAGATCTGTTAGGGTGATGGTGAAAGGGAGTCATATTCCGCTGTACGGAGGGGGAGCCAACCTGA
	ACAGCGTGAATGGTGCGCCAGTTGAAGCGGTACCATTGAAATTGAGCGTGATGGTAAGGTCAAGAGCTTAT
	GTTTTGGGGAAATTGGTGAAGCCCAAGTTCTACAAGAAGATAGATTGCTCCGTCGTTATGGATCCGAAGAA
CrLEA2-27	GATGGGCAAACCCATTTCGCTGATGACCAAGTGCACTTATCAGTAA
	ATGACCAATAGAGGGGGAGAGAGAGAGAGAATGACCACAACAGGTAACCAACCACAACCACACCCACATCC
	ACAGCCACAGCCACAGCCACAGCCACAGCCACAGCCAAACGGTGAGCGAAGACAAAGGCCAGCACCAGCAT
	CAGCACCAGAAACACCGGCACAATATTCGGCTTCTTCAAATGGAAATGGTTACAGGCAGTACAACCCAACG
	ACGCCGTCTCGTTCGTCGTCCTCGGCGTCGTTTAAAGGGTGTTGTTGCTGTCTGTTCCTGCTGTTCTCGTT
	GCTGGCACTGTTGGTGTTAGCGGTGGTGCTGGTAATAATCCTAGCGGTGAAGCCGAAGAAGCCACAGTTCG
	ATCTTCAGCAAGTTGGGGTGCAGTACATGGGCATAACACCCAATCCTCCCTCCACCGCCTCCCTCTCCCTC
	ACCATACGCCTCCTCTTCACCGCCGTTAACCCAAACAAGGTCGGCATCAAGTACGGCCAGTCTAGCTTCAC
	CGTCATGTACCGCGGCATCCCCCTCGGCAAAGCCTCCGTTCCCGGCTTCTACCAAGACTCTCACAGCGTCC
	GACAAGTCGTCGCCACCATCGCCGTCGATCGGGTCAATTTGCTTCAGGCCGATGCCGCCGATTTGATCCGA
	GACGCTTCCCTCAATGACCGCGTCGACCTTCGTGTCCTCGGCGATGTTGGTGCCAAGATCCGTGTCATGAA
	CTTCGATTCTCCTGGTGTTCAGGTCTCCGTGGATTGTGCTATAGTGATCAGTCCAAGAAAGCAATCTCTGA
CrLEA2-28	CTTACAAGCAGTGTGGATTCGATGGATTGAGTGTTTAA
	ATGGCTCATCCTCCACCACAATCACAGCCTCAAGCAGGGAAGCCAAACCGCTCAATCCTGCTCCGTTACAT
	TGCAATGATAATTCTTTTCTTAATTATCCTTGTGGGTATAGCTGTGCTCATAATTTGGCTGGTTTTGAAGC
	CGAAACGGTTGGAGTACAGTGTGGAAGATGCTGCAATCCACAAATTCAACTTAACGGATGCAAATCACTTG
	TATGCGAACTTTGATTTCACCATAAGAGCATACAATCCCAATTCAAGAGTTTCTCTGTACTATGACTCTGT
	GGAGGTGGCAGTGCGTTATGAGGATCAAACATTAGCCACCAATGCGGTTAAGCCATTCTTCCAGTCACATA
	AAAATGTGACTAGGCTGCATGTGGGACTCACTGCTCAAACCGTGGCTTTGTATGATTCTGTGCCTAAGGAC
	CTTAGACTTGAGAGGTCCTCAGGGGATATTGAGTTAGATGTGTTGTTGAGGGCAAGGATACGCTTCAAGGT
	TGGCGTCTGGAAGTCAAAGCATCGAATTATGACCATTTTTTGTTCTCCTGTGTTCCTCACATTTTCCAAAG
CrLEA2-29	CCAAGAGTTTCCAAAGGACACCTTGTGAAGTACAACTCTGA
	ATGCCATCCCTCTCACCCTCTCCTCTTGAAAAACTTGGCATACCAAAACAACCAAGGAATCAACATTTCCG
	GGAATTAGAAGCACCAAATTCCCTTAGTAACACTCTGGTAATACTTGGGCAACCTCACCTTCAAAGGACCA
	AACCAATTATATGGTGTGCTGCAGTGCTGTGTTTAATATTCAGCCTTGTACTTATTTTCTTTGGAATTGCA
	ACATTGATTTTGTACCTTGCTATGAAACCACGAAACCCAACATTTGACATTCCCAACGCAAGCCTCAATGT
	AGTGTATTTTGACTCACCACAGTACCTCAATGGTGAATTCACTCTCCTTGCAAATTTTTCTAATCCTAACA
	GAAGATTTCACGTGAGATTTGAGTCCTTAAAAGTCGAGCTTTTCTTCTCGGACAGGCTCATATCAACACAG
	TCAATCAAGCCTTTTACTCAGAGGCCAAAGGAAACAAGGTTGCAACCGGTGAACTTCATATCCAGCTTGGT
	GTTTCTGCCTCAAGATGTTGGGGTGAAGCTTCAAAGGCAGGTGCAGAACAACAGGGTCAATTACAATGCAA
	GGGGTACTTTTAAGGTGAGGGTCAACATGGGCCTAATCCATTTATCTTACTGGCTCCATAGCATATGCCAG
CrLEA2-30	ATAGAAATGACTAGTCCACCAACAGGAATTCTTGTAGCTAGACAATGCATAACAAACCGATGA
	ATGTCGACATCTGATAAACCAGAAATAGTGGAAAGGGGTTCTAAGGATGAAAAGCACAAGGAGGGTGAGAA
	AGAGGAAGGCAAGGGTGGATTTATTGAGAAGGTGAAGGATTTCATTCATGACATTGGTGAGAAGATCGAGG
	GAGCTATCGGGTTTGGGAAGCCAACCGCTGATGTTACTGCGATACACATTCCATCAATCAATCTTCACAAG
	GCAGATCTTGTTGTTGATGTGCTCATAAAAAACCCAAATCCAGTGCCAATCCCTCTGATTGACATAGATTA
	CTTGATTGAGAGTGATGGAAGGAAACTGGTTTCAGGATTGATACCTGATGCTGGTACTATCCATGCGCACG
	GAGAGGAGACTGTCAATATTCCTGTTACCTTGATTTATGATGACATCAAGCAAACATATAATGATATTCAA
	CCCGGAAGCATCATTCCTTATAGGGTGAAGGTTGATCTCATTTTTGATGTTCCTATCTTGGGAAGGTTTAC
	ACTACCTTTGGAGAAAACTGGAGAAATTCCCATACCATACAAGCCTGATATTGATCTGGAGAAGATTCATT
	TTGAAAGGTTCTCTTTTGAGGAGACAATTGCAACTCTTCATTTGAAATTGGAAAACAAGAATGATTTTGAC
	CTGGGCCTCAATGCGCTTGATTATGAGGTCTGGTTTGGTGATGTTAGCATTGGAGGTGCAGAACTCACCAA
	ATCTGCAAAAATTGAAAAAAGTGGAATTAGTTACATTGATATTCCTATTACCTTCAGGCCAAAGGATTTTG
	GTTCTGCTGTCTGGGACATGATTAGAGGAAAGGGAACCGGTTACACCATGAAAGGAAATATTGATGTTGAC
	ACTCCCTTTGGGCCTATGAAATTGCCCATCAGCAGAGAAGGTGGTACTACCCGTCTTAAGAAAAAGAAGGA
CrLEA2-31	AGATCGTGATTATGATGATGATGATGATGATGAGGATTAA
	ATGGCCGACCGAGTTCACCCTCAAGACTCGCCTCCTGTTTCCGGAGAGTCGCAAACGACGTCGTCTCAGCA
	GCCTGCCATCGTTCAGAATCCGGCGCCGCAACCACTGTCGGAGAAGCCTGTTCCTCCGCCGGGAACCTACG
	TCATCAGGATTCCCAAGGACCAAGTCTACCGCGTCCCTCCGCCGGAGAACGCCCGCCGTCAAGACCAATTC
	ATTCGCCGGAAACACCGTCGGAGCCGATGCTGCTGCTGCTTCTGCTGGCTCATCGGATTTATCTTCATTCT
	CATCGTGCTCCTCGGAATCGCCGCCGGAGTTCTCTACCTAGTTTTCCGTCCGGAGGCGCCGAAATACACCA
	TCGACGGCATCGCTATCAGAGGAATGAATGTCACGTCGCCATCTTCTGCGGCGGCAGTTTCGCCGGAGTTC
	GACGTCACCGTCAAGGCCGACAACCCCAACGACAAAATTGGAATTCGTTACGAGACTGATAGCTCCGCCGA
	AATCTTCTATAACGACGTGAGGCTGTGTAACGGCGCTCTTCCGGCGTTTTACCAGCCTTCAAATAACGTAA
	CGGTGTTTAAGACGGTGTTAAGGGGAAACGGAATCAAGCTGAGGAGCGGAGACCGAAGCGCGTTGCTGGAG
	GCCCAGACCAAACGGAAAGTGCCGTTGACCGTTAGTCTTAGGGCGCCGGTGAAAATAAAAGTGGGGTCCGT
CrLEA2-32	TCAGACGTGGAAGATTACCGTTAAGGTGGACTGTGATGTAACGGTGAATGAGCTAACGGCGCAGGCCAAGA

	TTGTTTCGAAGCATTGTAATTATAACGTGGATCTTTGGTGA
CrLEA2-33	ATGGCAGATCCTCAGAGAATTCACCCTGTCCATGATGTGGAGGCACCAAACCACCCTACAGCACCATTGGT<br>TCCTAGAAGCGTGTCTAAATCTGATGCAGGTGATCCAGAGAATGTGGTGCACCACCCTCCTCCTCCTACTT<br>CTCTTCCTTCACAACCTGTGATGCATTCAAAGCCACCAAAGAAAAGAAGAAGTTGTTGCTGCAGGTTTCTG<br>TGTTGGTTTATAAGCATATTGCTGATTCTGATTGTGGCTATTGGCATTACCATTGGGATACTGTTCCTTGT<br>GTTTAGACCAAAGCTTCCAAAATACTCAGTTGATGAACTTAGAGTCACACAGTTTGATCTTGCTGACAACA<br>ATAGCCTATCAGTTACTTTCAACTTGACAATAATTGCTAGAAACCCAAACAAGAAGATTGGAATAGACTAT<br>AGAGGTGGAAGTCACATAAGTGCTTGGTATATGGACACAAAACTATGTGAAGGGTCTTTGCCAAAATTCTA<br>TCAGGGTCACCGCAACATCACAGTGCTTAGTATCCCTCTTATAGGCCATACACAGGATGCTAGTGGCTTGC<br>AAAGTACCATTCAGCAGCAGCTGCAACAAACGGGTAATGTTCCCCTCAATTTGAAGGTCAAACAGCCTGTG<br>AGGATTAAGCTTGGCAAGTTGAAGCTGTTCAAAATCAAGTTCAGGGTCAGGTGCAGGCTTGTGGTGGACAA<br>CCTTAATGCTAATAATTCTATTAGAATTCGGAGCAGTAGTTGTAAATTCAGGTTTAGGCTGTGA
CrLEA2-34	ATGAATTGTAGACCTCGTTGCAGCTTCTGCTGCATCTGTTGCATCTACACCACAATCTACGTCCTTATGTT<br>CATCTTAATGCTTCTTCTCATCATCTTCTGGATAGTAATCTCACCCTCAAGTGTCAAATTCCATGTAACTG<br>ACGCCACCCTCACACAATTCAACCTCACAACCAACAACACCTTACATTACAACTTGAAGGTCAACATCACA<br>GTCAGAAACCCCAACAACAACGTGAAAGTATATTATAGAGCTATCACAGCAATAGCTTGGTACAAAGATAA<br>TGACATTGGTTTGGTGAGTTTGACACCCTTTGACCAAGGCCACAAGAACACAACCTTCCTTCAAGCAGTGT<br>TTATAGGACAAAGTGTGATTAAGCTCCAAGCTAAACAACTTGATGAGTATAAGGAAGAAACAAGTGTTGGA<br>ATCTACAATGATTTGGCTGTGGATTTGGATCTTACAATCAGAGCTAAGTTTGGAAGGTTCAGAAGCAGTCA<br>TTATAATCCACCAATTGTGCAGTGTCGTCGCTTGAAAGTTCCTTTGATTTCCAATGCTAAAACAACACCTC<br>CTTTCAGTGTCACAAAATGCAGCACTGGTTATTTCTTTAAAGATCGTGATGCTGATGCAGAAGGCTGA
CrLEA2-35	ATGTGTTGCAAACCCCGCAGCACCTTCTGCTGCATCTACTGCACCTTCTACACTCTCATGATCATCTTTTT<br>CCTTTCTGTCATCATCTTCTGGATAATCATCTCACCCTCAAATGTCAAGTTCCACATAACTGAGGCCTCCC<br>TCACACAATTCAACCTCACAAACAACAACACCTTATATTACAACTTGAAGGTCAACATCACAGTTAGAAAC<br>CCCAACAACAACATCATAGTATACTATAGGAGAATCAAAGCAATAGCTTGGTACAAAGACAATGACTTTGG<br>TTGGGTAAGTTTAACACCCTTTGATCAAGGTCACAAAAATACCACCTTCCTTCAAGTAGAGTTTAAAGGGC<br>AAAGTGTGATTAAGCTCAAAGCTCAACAACTTGGTGAGTACAAAGAAGAAACAAGTGTTGGGATTTACAAT<br>GATTTGGCTGTGGATTTGGACCTTAGAATCAGAGCTAAGTATGGAAGGTTCAAAAGTAGTCGTTTCAATCC<br>ACCAATTGTGCAGTGTCGTCGTTTGAAAGTTCCTCTGATTTCTAATGGTAAAACACCAACTCCTTTTAGTG<br>TTACCAAATGTAAAAGTGGTTCTTTCTTTGTGGACCTAATCTCACCCTCAAGTGTCAAGTTCCATGTAACT<br>GATGCCTCCCTCACACAATTCAACCTCACAAACAACAACACCTTATATTACAACTTGAAAGTCAACATCAC<br>AGTTAGAAACCCCAACAATAACATCATAGTGTACTACAGGGGTATCACAGCAATAGCTTGGTACAAAGATA<br>ATGACTTTGGTTGGGTGAGTTTAACACCCTTTGACCAAGGCCACAAAAATACAACCTTCCTTCAAGCAGTG<br>TTTAAAGGCCAAAGTGTGATTAAGCTTAAAACAAAACAACTTGGTGAGTACAAAGAAGAGACAAGTGTTGG<br>GATTTACAATGATTTGGCTGTGGATTTGGACCTTAGAATAAGATCTAAGTATGGAAGATCGGGCTTTAGCT<br>TCTGTTGCTTTTTCTGCATCTACAACACATTCTACACCTTTATATTCCTCTTCACACTTTCCCTCATCATC<br>TTTTGGATAATAATCTCACCCTCAAGTGTCAAGTTCCATGTAACTGATGCCTCCCTCACACAATTCAACCT<br>CACAAATAACAACACCTTATATTACAACTTGAAGGTCAACATCACAGTTAGAAACCCCAACAAGAAGAGCA<br>TAGTGTACTACAGTTGTATCACAGCAATAGCTTGGTACAAAGATAATGACTTTGGTCTTGTGAGTTTAACA<br>CCCTTTGACCAAGGCCACAAGAATACAACCTTCCTTCAAGCAGTGTTTAAAGGGCAAAGTGTGTTTAAGCT<br>CAAAGCTAAACAACTTGGTGAGTACACAGAAGAAACAAGTGCTAGGATTTACAATGATTTGGCTGTGGATT<br>TGGATCTTAGAATCAGGTTTAAGGAAGGAAGGTTCAAAAGTAGTCGTTTCAATCCACCAATTGTGCAGTGC<br>GTCGCTTGA
CrLEA2-36	ATGACTGGGAAGGACGAGTTTCTCTCGTACTCGCCCCTTCCCTCCAATCCAAATCCATATTCTAATAACCC<br>TAATCCTAACCCTAACCCTAACCCTTACCATTACCCCCAAAACGTCGTCGTTCTCCTTCCTTCCTATCGGT<br>CACCTTCCCTCCGTCGCCGTCGTCGCTGCATCCTCTACTCCGCTGCGCTCTTCATCTTCTTCCTCCTCGTC<br>GCCGCCGTCTTCATCTTCTACCCATCCGACCCGGAGATCCGCCTCGTCCGGATCCGACTCAACCACATTGG<br>AATCCGAACCTCTCCGAGGCCCATTCTCGACCTCTCTTTCTCCATCACCGTCAAGGTTCGCAACAGAGACT<br>TCCTCTCCCTATCCTACGATTCCCTCTCCGTCTCCGTCGGTTACCGTGGCCGCCAGCTAGGTTTCGTCACC<br>TCCTCCGCCGCCGGCCGCATTAGGGCGCGTAGCTCCTCCTACGTTGACGCCACGCTCACCGTCAATGGATT<br>CGAGGTTATCTACGATGCTTTCTACTTGCTGCAAGACATCGCCAGAGGTGTTATCCCGTTCGATACCGATA<br>CTCGAGTTGAAGGGAATTTGGGGCTTTTATTCTTCAATATTCCTTTGAAGGCTACTGTGTCATGTGAAGTG<br>TATGTCAATATAAACCAGCAGACAATTGTACGTCAAAACTGCTACCCTGAGTCACTGGGTGATCCGCTGGA<br>TCAGAGCACATATATTGGAGTTGCAGATACATGA
CrLEA2-37	ATGCACGCCAAGACCGATTCTGATGTCACGAGTTGTGATCCATCATCGCCAAGATCTCCAAAACGCGCCGT<br>TTACTATGTGCAGAGTCCCTCGAGAGAGTCTCATGATGGTGATAAATCTTCCTCTATGCATGCCACTCCAG<br>CATGTAATAGCCCCATGGAGTCACCTTCACACCACTCGTATGGTCACCATTCTAGGGCTTCCTCTTCCAGC<br>AGGGTCTCCGGTTCCTACAACTCTTCTTGGGGAAGGAAAGGGGACCGTAAACGCAATGATAAAGAGTGGCC<br>TGAATGCAAGGTGATAGAGGAAGAGGGTGGTAATGGTGATTTTTACCCTGATAATAAAGGCTTCTCAAGGA<br>GGACTCAGATCTTCATTGCTATGATTGGTTTTGTCTTGATTTTCGGTGTGTTCTGTTTGATCATTTGGGGT<br>GCTAGCAGGCCTTACAAACCTCAACTCAGTGTCAAGAGTTTGACAGTACACAACTTCTACTTTGGAGAAGG<br>GACAGACATGACAGGAGTTCCAACTAAGATGCTGACAGTGAATTGCTCAATGAGAATGATGGTGCATAACC<br>CTGCAACCTTTTTTGGCATTCATGTCAACTCCAAGGCAGTGAGTCTTATGTATTCTGAGATCACAGTTGCA<br>ACTGGTGAGCTGAAGAATCATTATCAGCAGAGAAAGAGTCGCAGGACAGTGTTTGTGAACCTGCAAGGAAG<br>CAAGGTTCCATTATATGGTGCTGGTGCAAGCTTAGCTGGTTTGGTGGACAAAGGAAAAATACCAATGACGC<br>TTGTCTTTGAAGTAAAGTCACAAGGGAATGTTGTTGGGAAGTTGGTGAGGTCCAAACATAGACAGCATGTT<br>TCTTGCTCAGTGGCTATTGATTCCCACAACAACAAACCCATCAAACTCAAAAACAATGCATGTACATACGA<br>TTGA
CrLEA2-38	ATGAAACAGAATACAATAGGATCCAAAATTTTCTTGTGTACACCTGCATTGGTTTTTCCCAAAAAACAAGA<br>TTTCAAAGGTAATCCCAATTGTTCTTATTACCCGGAACCAGAAACTATGGCTAAAAGAAACCTCAAAATCT<br>GCATTTCTGTGTCTGCAGTTTTCTTTATAATTGTCATCACTGTGTTTCTGACCTTAATTTTGACCATATTT<br>AAACCCAAAAACCCAGATATATTTCTCCACCCTGTTGACCTCAAAAACTTTCAGTTATTGTCATCCAATTC<br>AACTAATGCACCACTAGGCATGGTGGTCACAATAGTGAATCCAAACTATGGAAGCTTCAGGTACATAAATT<br>CCACTGGTTATCTTGAATATCGTAATACTACAGTAGCCAAAGTTCCCTTGGAGACAAAATCTGTTCCTGCG<br>CGTACCAGGACCAATGTGAGCACTTCTGCAGGTATTATGACAGAAAAATTGATTGATGATCCAAAATTTTG<br>GTCAGAAATTGAAGGTGGGACCTTCAATTTGACAGCAAAGGCCACATTGCCTGGGAAAGTGAGTATGTTCA

	ATATTTTCAAGCTTAAGGCCACGGTTCATATTTCATGTGATATCTCTATTAACATAATTGCTATTGATTCT
	GGTTCCACCTGCGTGGCCAAACTCAAGTTATGA
	ATGTCTAGCAAGGACTGCGGCCACCACGAAGAGGAGCGCCGGCAGCTTCTCCGCCGCATAGTTGGTGCCAT
	CGTAGCCTTCATAGTCTTAATCCTCCTCATAATCTTCTTAATCTGGATCATCCTCCGACCCACAAAACCCC
	GATTCATTATCCAAGACGCCACCCTCTACACCTTCAACTTCTCATCCACCTCACCCTCTGTAACTTCCCCC
	ACACCCAACATCCTCACCCTCACAATGCAAGTCACGCTTGCCGCACACAACCCCAACCACCGCATCGGCAT
	TTACTACCACAATCTCTATGCCTATGCCTCTTATCGTAACCAACAGATCTCCCTCGCCACTTCCATTCCCA
	ACACCTATCAAGGCCACAAGGACTTCATCATTTGGTCTCCGTTCATCTACGGTAACGCCGTTCCGGTTTCG
	CCGTTTCTGTTGAGTGCTCTGCAGCTCGACCAGAGTACCGGTACCGTTTTGGTCAACGTCAAGGTCAATGG
	AAGAGTCAAGTGGAAGGTTGGGACTTGGATCTCCGGGAGGTACCATATTTATGTCAACTGTCCTGCGTATA
	TTAGGTTCGCCGGAGATCGGAGTAATGCCATCGGCGGGGTGACTCCGGCGGTGAAGTTTCAGCTTTTGCAG
CrLEA2-39	AGTTGTAGTGTTGATGTTTAG
	ATGTTGAGCAACATACTTACCTGGACGGGGCCAATGGGTGATCCAGAAGGCCTATGGCCTAGGGAAGTAAC
	CTCCATTGCACTTTGGAGGGGTGCTTCTCTAAGGTCTGCTCAAATCTCCGTGCTGATCTTTGGTCTCAATG
	TCTCTCATCTCTTCCTGAATATCCCCCAACTCATTCACAGCTTCTTGATTCTCCAAGTGGCGATGGGCAAA
	AAGGTAAAATGGAGTTGGAGCTCGGCGTTTGTGGGAGCAGCCTCAGCGGTAGCAGCAACAGCGATACTATC
	AGCGAAACCCAAGGACCCAACTTTTCACCTCATATCAATCAATTTCACTTCCCTCAAGCTCAACCTCCCCC
	TCCTCGACGCAGAGCTCCTCCTCACCGTCCACGTCACCAATCCCAACATCGCTCCGATTCACTACTCCTCC
	ACCACCATGTCTATTTTTTACGAGGGTTCCTTCCTCGGCTCGGCTCAGGTTCAAGCCGGCTCACAGCCTCC
	GCGTTCTTGCCAGCTCCTCCGCCTCCCCGCTCGCCTCCATGCGCTGCAACTCGCTCACCACGCCACTCGCT
	TTGTTCACGACGTTGCGCGCCGCGAGATGGTCCTTGACGCCGCTGTTGATATTGCCGGCACCGCCAAGGTT
	CTTTGGTGGGACCACAACTTCAAGGTCCACGTGGATAGCCGCGTCACCGTCGATCCCGTCTTTCTTGACGT
CrLEA2-40	CATTGATCAGGAAAGTACCTCGGAACTTGAAGTCTTCACCGCAGCGTAA
	ATGGGAGGAAGAATGCACACTAAGTCCGACTCAGAGGTCACCAGCAACAGCATCGACCACTCATCCTCGTC
	GCCGCCGCGTTCCCCTCCCCGCCGGCCACTCTATTACGTCCAAAGCCCTTCAAATCATGACGTTGAGAAGA
	TGTCTTACGGTTCAAGCCCCCTCGGTTCCCCTCACCACCATATCCATTACTACCTCTCTTCCCCCATTCAC
	CATTCCCGCGAATCTTCCACCTCTCGTTTCTCTGCTTCTCTCAAAAACCCTCGCCCTTTCTCCTCCTCCTG
	GAAGAAACTCCACCCTCACCCTCACCACGGCGTCGACGACGACGAAGACGACGACGACGACGACCTTCACG
	ATTCCTCCCGCAACCTGCGCTTGTACCTCTGCTTCTTCTTGCTTTTCGTCTTGCTTTTCACCCTATTCTCT
	CTCATCCTCTGGGCTACCAGCAAGACCTACAAGCCTCAACTCGCTATCAAGAGCATAGTGTTCGAGAACTT
	GAACGTACAATCGGGAAACGATGGAACTGGGGTGCCAACAGATATGTTATCGTTGAACTCAACGGTCAGAA
	TTTTGTACAGAAATCCTGGCACTTTCTTCGGTGTTCACGTCACTTCCACACCTCTTCAGCTTACCTATTAC
	CAGCTTGCACTAGCCTCGGGTCAGATGCAGAAGTTCTATCAGTCAAGGAAGAGTCAGCGGAAGGTAGTGGT
	GGTGGTGTTAGGACACCAGATTCCGCTCTATGGTGGAGTGTCTGGTCTTCTTGGAAATTATAAAGAGCATA
	TCGACAGTGTTGCATTGCCACTGAACCTGACATTTGCAGTGAGATCAAGGGCTTTCATCTTGGGAAGATTG
	GTTAAGTCTAAATTCTATCGAAGAATCAGATGTTCTGTCACTTTGCATGGCAACAAACTTGGAAAACCTCT
CrLEA2-41	TAATTTGAAGGATTCGTGTGTCTACAAGTGA
	ATGTCATCTGATCCTTATGCCCATCAAAGATATGTCTCCCTGCAGAACCAAGGCTATGTATCAGGGCCTCC
	TCCTTATGGGTATGGCCGTAAGCCTCCACGTTACCATCACCACAGTGGTTCTGGTTGTTTATGTGGATGCC
	TAAAGTTCTTCTGTTGCTGCTTCTATAGTTGCTGCCGTTGCTGTATTTGCGCCTTCTTTATCTTCATTCTC
	ATTTTGGTGATCATTATTATGGTTTTATATTATCTTCTTAAGCCTGACATACCTTCCTACAATATTCAAGG
	AATTGATATCAACACTTTTGATTTAAAAATTGATAATAAGCTCTTTTCTAATATTTCCATTGTTGTGAAAG
	CGGATAATCCAAATGAAGGGATTGGGTTGAATTATTTAGATAATGAAGTTAGGTTATTGTATTCAGGGTCC
	CAACTTTGCTCTGGGGCGTTCCCTCCTTTCTTGCAACCAGAGAGGAACGTAACCACGTTCAATGTAACATT
	GAAGGGGGAGAGTGAGTTTGGCCCTGAAATGCAACAGCACTTAATGCAAGAACAGGATAAAGGGACAATTC
	ATTTGTTAATAGCAGTGAGGCTTCCCATAAGGCTTGTGGTTGATGACCTTATTCATCTGAGGAAGTTCGTG
	GTTAATATAAATTGTTCAATGATTATTGATCACTTGGAACAAAACAAAAAGCCTAATATTTTGAGGAAAGA
CrLEA2-42	CTTCATCTACGGAATCGAGTTTTGA
	ATGGTTTCTAGAGGCGGCAAAATTTGCATCGGCGTGTCTCTAATATTGGTTATCGCTATTGGCACTGTGAT
	TGCGACATTGTCACTGACAATCTTTAAGATCAGAGATCCTGTGATCAGTATCCACCCAGTTGGTCTCGAAA
	ACTTAAAGTTTTCTCTTAATTTTTCGTCGAATGTGAGTCTAGGCATGATTCTCACTATGGGGAATCCAAAC
	TATGCAAGCTTCGAATTCAAAAATTCGATCAGTCATGTCAATTTTCATAAGAACGTTGTAGCTGATGTTCC
	AATTCAGGGAGAGGTAGTTCCAGCGCGTGGTACCATTAATGTGACCACTTCCGCGGATTTGATGGTAAGAA
	AGTTAATACTTGATCCCAAATTTTGGTCAGATATTTTAGGTGGCCATTTGAATTTGACATCAACAGCTACA
	CTGCCTGGGGTAGCGCATGTGCTTAACTTTATCAAATTGAAGGCCACGGCTTATAGCTCATGTACCATCTC
CrLEA2-43	TTTGGAAATACGTACTAAGAATATTTCTTCTAATTGCATATCCAAAATCAAGCTTTGA
	ATGGCTACTAGAAGCGTCAAAATTTGCTTGTCTGTGTCTCTACTTTTCTTGATCATTGTTACCATTGTGAT
	TGTGACATTGTTTCTGACTCTCTTTAAACCCAAGGATCCTAACATCACTGTCCACCCTCTTGGCCTTGAAA
	ACCTTCAATTTTCCCTTTTACCCAATTTGACACTAAATGTGTCTCTAGGTATGTTGATCACCATAGGGAAT
	CCTAACTATGGAAGCTTCGAGTACAAAAACTCCACTGGTTATATCAAATTTCATGACACTGTTGTAGCCCA
	AGTTCCAATGGAGGCAGAGTTAGTCCCAGCGCGTAGCACCATTAATGTGAACACTACAGCAGATTTTATGG
	TTGGAAAGATGATAAATGATCCTAATTTTTGGACTGATGTTTTAACTGGGACTTTGAATATGACATCAACA
	TCTACACTTCCTGGGGAAGCGCGTATGCTCAAGATTATCAAATTCAAGGCCACGGCTTATAGCTCATGTGA
CrLEA2-44	CATCTCTTTTAATATAACCTCCAAGCATGTTGATACCAAATGCATATCCAAATTCAAGCTTTGA
	ATGACAAAGGACTGCGGCCACCATGATGAGGAGCGGCAACAACTCCTCCGCCGCATATTCGCCGCCATCCT
	TGGTTTCATTATCCTCATACTCCTCGTAATCTTCCTAATCTGGATCATCCTCCGACCCACCAAACCCCGTT
	TCATTCTTGAAGACGCCACCGTCTACGCCTTCAACCTCTCCTCCACCGGCGACATACCTTCACCCACCGCC
	CCCACACCCAACACTCTCACTCTCACAATGCAAGTTACTCTTACCGCTCTCAATCCCAATTCTCGCATCGG
	CATTTATTACCACAACCTCCACGTCTACGCCTCCTACCGCGGCCAGCAAGTTTCCCTCGCCACCGAGCTGC
	CACCGACCTACCAGGGCCACCGTGACACCACCGTGTGGTCGCCGTTCCTGTACGGAAACGCCGTGCCGGTT
	TCCCCCTACGTGTTGGAGGTGCTGGAGCAGGACAAAACCTCAGGGGGAGTGTTGGTCAACGTCAAGGTCAA
	CGGTAGAGTAAAATGGAAGGTGGGGACTTGGGTTTCTGGAAGGTACCATATCTATGCCAACTGCCCCGCGT
	ATATTAGGCTCGCCGGAGATCGGGATACCGGCATCGGAGTTGCGGCGCCGGCGGTTAAGTTTCAGCTTTTC
CrLEA2-45	CAAAATTGCGTTGTTGATGTTTAG
	ATGACTGACAGAGTCTACCCTTCTGCCAAGCCCGCCGCCAACGGAAACGGCGCCGCCGCTAATCCATCTTT
CrLEA2-46	CCCGGCGACAAAGGCTCAACTCTATGGCGCCACCCGCCCTACCTACCGTCCCCAACCTCACCACCGCCGCC

	GTAGCCGACGCCGATGCTGCTGCACGTTCTGCTTCTGGCTCATCCTCATCCTCCTAATCCTCCTCTTCCTC
	CTCGGCGTCGCCGGCACCGTCTTCTACCTCCTATACCGCCCCCACCGTCCTTCCTTCACCGTCACATCGCT
	GAAACTGTCGTACCTGAACCTCACCACGTCCTCCTCCCTCAGCTCCAGATTCGACCTCAACGTGAGCG
	CCACCAACCCTAACAAGAAGATCCTCTTCGCCTACGACATCACCTCCATCTCCATTCTGTCCGGCGATGTG
	GACGTCGGCGACGGCACCATTCCGGCGTTCCAGCACGGAAAGAAGAACACGACGCTGCTAAAGGCTTCAAT
	CTCAAGCAGTGGACAGGCACTGACGAGTGACGATGCGTCGCGGTTGAAGGCAAACATGAAGAGCAAGAGTG
	GATTGCCATTGGAAGTGAAATTGGAGACAAAGGTGAAGGCAAAGATGGGAAACTTGAAGACTCCAAAGGTA
	GGAATCAGAGTGTTTTGCGACGGAATAAGAGTCACTCTTCCCACCGGTAAGAAGCCTGCAACAGCGTCCAC
	CAAAAATGTTAAGTGCGACGTCAATGTTCGATTCAAGATCTGGAAGTGGACCGTTTGA
	ATGCTGTCTGCAAAATCTGAATCAGATATTACTAGCTTAGCTCCATCTTCACCTTCAAGATCTCCAAAGCG
	GCCTGTGTACTATGTGCAAAGTCCTTCAAGAGATTCACATGATGGAGACAAGTCATCTTCAATGCAGGCTA
	CTCCAATATCAAACAGTCCTATGGAGTCTCCTTCTCACCCTTCTTTTGGGCGTCATTCTAGAAACTCTTCT
	GCTAGCAGGTTTTCAGGGATTTTCAGGTCATCTTCTGGAAGGAAAGGTAGTAGGAAGAGGAATGATAAGGG
	GTGGCCTGAGTGTGATGTGATTCTAGAAGAAGGCTCTTATCATGAATTTCAGGACAAGGCCTTTACTAGGC
	GCTTCCAAGCCTTGATTGCTGTGTTTACTTTTGTGGTTGTCTTCACCGTGTTTTGCTTGATCATTTGGGGT
	GCTAGCAGGCCTTACAAAACTGAGGTTACTGTTAAGAGCTTCACAGTACATAATCTCTATGTTGGAGAGGG
	TTTAGACTTCACAGGTGTCCCAACAAAAATGCTGACAGTGAATGGCACTTTACGCATGAGCATCTACAACC
	CTGCTACATTTTTTGGCATCCACGTGCATTCCACACCCATCAATCTTATCTTCTCAGACATCACCGTTGCA
	ACTGGCAAGCTAAAGAAATACTATCAACCCAGAAAAAGTCACCGTATTGTATCAGTGAATGTAGAAGGTAA
	CAAGGTTCCCCTGTATGGGGCAGGATCCACCATAACAGTATCACAAACTGGTGTTGAAGTTCCACTCACAT
	TAAACTTTGAAATCCGTTCCCGTGGAAATGTAGTAGGTAAGCTGGTAAGGTCAAAGCATCATAAGGAAATC
	ACTTGTCCATTGGTCATCAACTCTTCTGGATCAAAACCTATCAAGTTCAAAAGGAATGCATGCACATATGA
CrLEA2-47	ATGA
	ATGATGTGTTGGATATACGTACCATCTTCGACGTTCATAAACTGCAATAGCAAAAATACAGAGAGCGTCCA
	AAGGAAAGCCATGAAAATCTCAAAAGCTGCTTACGACCTTCTCCTACGGAAACCTCATCAGCATCAAAACG
	ACATCGTTTATTCACGCCGTTGTGTACTGACGCTCACCTGCACGCTGGGTCTGGTGCTCCTCCTCACCACC
	ATCTACGCTTTCTGGCCGTCAGATCCTGATCTGAAGATCGTAGGGTTCAGGCTGAAGCGGGTCAAGCTGCA
	CCCGTTGCCTCCCGTAACCGTGGACATATCCATGCTCATCACGCTGAGAGTGCACAACACTGATATGTACT
	CCTTGGATTTTGGAGCCGTTAACGTGATCGTTGCCTACAGGGGGGTGAGGCTCGGGCACGTGACATCCAGC
	CATGGGCACGTGAGAGCCAGGGGATCTTCCTACGTGGATGCTGACGTGGAATTTTCTGGAATCAGTGTGCT
	TCCGCAGATGGTATGGTTGCTGGAGGACGTTGTTAAGGGTATCGTACCCTTTGATACTGTTAGCCAGGTTA
	GGGGCCAATTGGGGCTTCTCTTCTTTCACTTCCCTATGAAGTCAAGGCTATCGTGTGAGGTTGTGGTGAGT
CrLEA2-48	ATACAAAACCAGACGATTGTTCGCCAACATTGTCACCGCGGGAGATGA
	ATGTGTGAGAGCAAGAGTTTCTACAAATGGCTCATGCAATTCATATGCCTTTTAGGCCTCCTTGTTCTGTG
	CCTATGGCTGTCCTTACGACCCAAGAGCCCCTCCTACTCCATTGTATGCATCTCCATAGAACAAGCCTCGA
	ACTCGAATCAAAACGGCACCATCTCTTACCAACTTCAAATTGAAAACCCAAACAAGGAATCAAGCATTTAC
	CATGATGATATAATATTAAGTTTCTTATATGGGCAGATGGAGGATGAGGTGGGGGAGGATACCATAGGCTC
	ATTCCATCAAGAAACTGGTAACACCCGAGATAAAATTGGTACTGTCAACGCCAAACCTCGACCATTCAAAC
	CTCTCTTGAACGCCATCTCAAATGCAACCGCAGAGTTAACAGTTGCTTTGAAGACAAAATTTCGATACAAG
	ACATGGGGAATTAAGAGCAAGTCCCATGGTTTACACCTCAAAGGTATTCTACCAATTGATTCCAATGGGAA
	GCTTTCACGTAACAAGAAAAAGTACCCACTTAGTCCCAATTCCCAAAAAATAGGAAGGTACAAAATAAAGC
CrLEA2-49	ACTGA
	ATGAGGTGTTCCAAAGGGTTGAAAATCTGCTGTGCTGTCTCTGCCATTCTTCTAATTATATTGGTTGTGGT
	ACTTGTGATCTTGTTCTTCACGGTTTTCAAGCCCAAAGACCCCGATATTATCCTACAGTCAGTTAAGCTTG
	AAAGGATCGAAATTGGCTTCCCAAGTCCAGAAATAAATGTGTCATTAGGCCTAGTGGTCACGGTTGAAAAC
	CCCAATCATGGAAGCTTTACTTACCAAAACAGCACTGCTTATCTTAATTATCGAGGGAATATTGTGGCTGA
	GGCTCCTATACATGAGGACACAATTCCTGCTCGTGGAGATCATAACATAAGCACATCTTTGGATATTTTTG
	CAGATATCAGAAAATTTAAGGATTTACCGAGTGATTTTTTAAGGGGCGTTATAAATTTTACCTCAACAACC
	ACCTTACAAGGCAAAGTCAAAATATTGCACCTCTTCAAGTTCAAGGCCACTAGTTACTCCACCTGTGATCT
CrLEA2-50	CTCTTTACTCGTACATGACAAAAGCATCAATTCTACCTGCAACACAAAAATCAAATTATAA
	ATGACCAAGCCACAGAGCCGCACCAACCTAGCTTCATGTTTCGTCGCCACAATCTTTTTGATTTTCCTTGT
	AATCGTCGTCTTCGTCGTTTACTTTACGGTTTTCAAGCCCCAGGATCCCAAAATCTCCGTCAGCGCCGTTC
	AGCTCCCTTCCTTTTCCGTCACTAACGGCACCGTCAATTTCACCTTTTCCCAATACGCTTCCGTTAGGAAC
	CCTAATCGAGGCACCTTCTCGCACTATGACAGCTCACTCCAGCTTCTCTACTATGGCAGACAGGTCGGGTT
	CATGTTTATTCCCGCCGGCAAGATCGACGCCGGCAGAACTCAGTACATGGCGGCCACGTTCACCGTTCAAG
	CATTCCCGTTGGGCTTTGGCCCCACCTTTATTAACGGGCCCATTAATGTGGGGCCCACGATGGAGATCGAG
	TCGAGAATATCGATGGCGGGACGAGTGAGAGTGTTGCACCTCTTCAGTCATCACGTTGAGGCCAAAGCTCT
CrLEA2-51	ATGCAGGGTCCCCATCGCCGTTCCCGATGGAACCGTCTTAGGTTTTCACTGCTAA
	ATGTCAGAAACAGTGACCGAGTCCCGGTCAAGTCCAGAACGAGGAGGTATATATACACACACAACATCAGA
	GAAATTAATCGCCATGTTCCCAATACGCAAAGAAGAAGAAAAAAGCAGCAAATGTGTGGTGTACGCTTTGG
	TTACCATTGCTACCCTATTGGGGGTTGTATGTTTCTCATCTATCTTTTTGGTGGTGCGAGATCCAAAAATT
	GAATTGATATCAGCTAGGTTGGTGGACAATATTAATCATACCGTTATTTCAGCATCATCTCCTTTTAATTT
	GAGCATGATTGGGCGTGTGAGCCTGTGGAACCCAAACTTTGGTCGCTTCTATTACGACAACACTACAGTGA
	GTGTGATGTATGGGGATTTTAGTATTGGTGCTTCCGAATTGGGAGGTGCGATTCTGGAGGCTAGAGAAATC
	AAAGAATTCAATTCCATCGTAAATATGAGGTTCAGTGAGGAATTGGTGAATCTTACCAATGATATCAGTTC
	ATGCATGCTCAAACTCAGAAGCCACTCGGATTTAAGTGGTATAGGGTACGTGCTCAAGTTTATAAAGAAGA
	GGAAGACCATACAAATGGACTGCACTATGAATCTTGACTTAACCTCTTCTTCACTTCGCCTACTCCAATGC
CrLEA2-52	TAA
	ATGGAAATGGCGACAAAACATTCTTCGTCAAGACATCACACAATTCACCAAGAGACAGCCATTTACAAACC
	CATACACCAAGAACGAAGCAGCAAATGGTTCGTGTTCCTTTTTTCTGCCTTTGTCATCTTATGTGCAGTTC
	TATTGGTCTTTGCATCCGTGTTGCGCGTGCGGAGTCCAGAGGTCAAACTAAGATCAGCTACCTTGAAACAA
	ATCAATTATAGAGCTTCGCCAGCACCTTCCTTCAACGCCACTCTGATCACCTTTCTCAGAATTAGGAACCC
	AAATTACGGTGCGTTCAGTTATGAGAATAGCACGGTGACTGTGCTGTATGCGGGTGAAAATATTGGTGATA
	AGGGAATCGGCAGTGACAGAGTTAGTTACAGAGAAACCAAACAAATGAATGTTACCGTGAATGTGAGGTCC
	TCTAAGCTACCCGTTACTGGGAATCTTTCGGGTGCCATTAATTCAGGAATGTTGAATCTCACCGGTTATGT
CrLEA2-53	CAAATTTAGTGGCACGGTTCATCTTCTCAAGATAATCAACAGCAGAAAGACCATACAAATTGCTTGCACTA

	TGAATCTCAACTTGACCTCCCACGCAATTAAGGGTATCCAATGCTAA
CrLEA2-54	ATGCCGTCCCATCATGACCATGGCCATGTGGCTGAGCATCATCATCATCATCATCGAAGCCCAATACCCAG<br>AAGACTAAATACTCCTCACAATGATAGCACCCATCCCTTAATCTGGCTAGCAGCCATTCTCTGCACAATCA<br>TAGCCATAGGTGTTGTAATTGCAGGCATAGTAGTTTTCATTGGTTATATGGTGATCCACCCAAGAATCCCC<br>ATCATAAGCATCACCAATGCTCATCTAGACCTTCTCAGAAACGACTATGCTGGTCTTCTACAAACCCAACT<br>TCGCATCATTGTGGTGGCACAGAATGGAAATGCTAAGGCTCATGCAACTTTTTCTGACATAAAGTTCAATG<br>TTAGCTACCAAGGGCAAGGCATAGCAGTGATGGTTGCTGACCCTTTTGATGTTCCAAAGAACAATTCAAAG<br>ACACTCCCTTATGTGGTTCAATCTGCTCCTATACCCTTAACCCCTGATCAGATGGAGGAAGTGGATGTTTC<br>TTGGAAACGAGATATCATTGGATTTGACATTAAGGGTAGCGCAAGAACACGGTGGAGAGTAGGACCTTTAG<br>GTTCTGTTAGATTCTGGTGCACTTTGGAGTGTCAACTCAAGTTTCACCCTTCCAATGGTAGTTACATTCCC<br>AAACGCTGCACCTCTGAATCAAAATGA
CrLEA2-55	ATGTCAACATCTGATAAACCAGAAGTAGTGGAAAGGGAAAGTAAGGATGGAAAACAAAAGGAAGATAATGA<br>TGAGAAGGGTGGATTTCTTGATTCAGTGAAGGGTTTCATTCAAGACATTGGTGAGAAGATTGAGGAAACTA<br>TAGGATTTGGGAAGCCAACTGCAGATGTTACAGGAATACACATTCCATCAATCAATCTTGAGAAGGTAGAA<br>CTTGTTGTTGATGTTCTCATAAAGAACCCGAATCCTGTGCCGATTCCCCTAATTGACATAAACTACTTGGT<br>TGAGAGTGATGGGAGGAAACTAGTTTCAGGATTGATACCGGATTCAGGTACAATCCATGCACATGGAGAGG<br>AGACAGTGAAAATTCCTCTTACTTTGGTTTATGATGACATAAAGAATACATATAATGATATTAAACCTGGA<br>AGCATAATTCCTTATAAGGTGAAGGTTGATCTCATTGTTGATGTTCCTGTCTTTGGAAGGCTCACTCTACC<br>TTTGGAGAAAACTGGAGAAATTCCTGTTCCATACAAGCCTGATGTTGATCTTGAGAAAATTCAGTTTGAGA<br>GGTTCTCTTTTGAAGAGACTGTTGCAATTCTTCATTTGAAGTTGGATAACAAGAATGACTTTGATCTTGGT<br>CTCAACTCGCTCGATTATGAGGTGTGGCTTGGTGATGTTAGCATTGGAGGTGCAGAACTTACCAAATCTGC<br>AAAGATTGTGAAAACTGGGATCACTGACATTGATGTTCCAATTACCTTCAGACCTAAGGATTTTGGTTCTG<br>CACTTTGGGACATGATTAGAGGAAGGGGTACAGGATACAGTATTAAAGGACATATTGATGTTGACACTCCT<br>TTTGGAGCAATGAAATTGCCCATTACCAAAGAAGGTGGTACTACCCGTCTCAAGAAGAAAAAGGAAGATGG<br>TGAAGATGATGAGGAGGAGCCTGATTGGAATTCAGTTTATGATTCTTGTTTGGCAGAATTTCAATATCGAC<br>ATAGTGGAGATGCTCAGAAACAAGGATGCTTCTCAAAACTCACTCTTGAAACAGTCTCCTTTAACTTGATT<br>AGAGTAGTGTTTTTGGTATATCCTAAGGGATTGAAGAAGAAATGGCAGCCAGTTATAGTATCTGTTCTTTT<br>ATTCACAGCTTA
CrLEA2-56	ATGCTTGCACTTCCACCACCCCCACCACCACCACCATCACTACCATCACCACCATCCCTAGCCATAAGGCA<br>AACCAAGCAAGTTTCTCCTGGCCAAATTGTCAAATCAAAACAGCCCATAAGACAAAATTCTCAAGAATTAG<br>AAGCACCAGATTATACTACAACAAAGTCAATTAGGCCACTACCAAGATTAAGGCAACCTCCATTTCAAAGG<br>ACCAACCCAATAATATGGTTTGGTGCAGTGTTGTGCCTCATTTTCAGCCTTGTGCTCATCTTCTTTGGAGT<br>TGCAACTTTGATCATCTTCCTTGCCATCAAACCAAGAAATCCAACTTTTGACATCCCAAATGCAAACCTCA<br>ATGGATTGTATTTTGACTCACCAGAATACTTCAATGGTGACTTCACTCTCCTTGTAAATATTACCAATCCC<br>AACAGGAAAATGGATGTGAGGTTTGAGTCTTTGGATGTTGATCTTTTCTTCTCAGATAGGATCATATCAAC<br>ACAGTCAATTCAGCCCTTTACTCAAAGGAGAAAGGAAAGCAGGTTGGAATCATTGCACTTCATATCCAGTT<br>TGGTGTTTTTGCCTGAGGATCTTGGTGTGAATCTTAAAAAGCAGGTGCAAGGTAACAGGGTTAACTACAAT<br>GTGAGGGGAACTTTTAAAATGAGAATAACTATGGGCTTCTTCCATTTATCTTATTGGCTGCATAGCAGATG<br>CCAGATAGAGATGACTAGTCCACCAACTGGGGTCTTGATAGCTAGGAACTGCATCACAAAGAGATGA
CrLEA2-57	ATGTCAGATCATCAAAGAATTCATCCTGTTCATGATGTGGAGGCACCACACAGACCCTTGGTGCCAAGTAA<br>CATTGGTAAATCAGATAAAGATGTTCAACCTCTTCCTCAACGCACCATTCCTGTGATGCATTCAAAGCCAC<br>CAAAGAAAAGAAACTGTTGCTGCAGGGTTCTATGTTGGACATTAAGCGTATTGGTGATTCTTATAATTGCT<br>CTTGCCATCACCATTGGGATACTATTCCTTGTGTTTAGGCCAAAGCTTCCCAAATACTCAGTGGACCAACT<br>GAGAATAAGCCAATTCAATGTTTCTGACAACAATAGCCTTTATGCTACCTTCAATGTGACAATCACTGCAA<br>GAAATCCAAACAAGAAAATTGGGATATACTATGAGGGTGGAAGCCACATAAGTGCTTGGTATATGGACACA<br>CAACTATGTGAAGGGTCTTTGCCAAAGTTCTACCAAGGTCACAGGAACATCACAGTGCTTAATCTGTTTTT<br>GACTGGTCAAGCACAGGATGCAACTGGCTTACAAAGTAGAATTCAGGAGCAGCTGCAGCAGACAAATAATA<br>TTCCCCTTAATCTTAAGGTTAATCTGCCTGTGAGGGTTAAGCTTGGTAAATTGAAGCTCTTCAAAGTCAAA<br>TTCAGAGTTAGGTGCAGGCTTGTGGTGGATAATATTGGTGCTAATAATGATATTAAAATTTCAAGTAGTAG<br>GTGTAAGTTCAGGCTTAGGCTATGA
CrLEA2-58	ATGACCGATCCAGTTCATCTGAAAAACTCGCCCCCACAATCCAACGACGCACAAACGTCGCCGCCTCATGA<br>AGCTGCCGTCACCTCAAATTCCTCCTCTCTGGCGGCAACCTACGTCATCCGTATCCCCAAGGACCAAGTCT<br>ACCGCGTTCCTCCGCCGGAAAGTGCTCTTCGCTACGCCAACTACAGCCGCCGGAAAAATCGTCGATCCCGG<br>TGCTGCTGTTGCCTCTGCTGTTTCATCTCCATCGTCTTCTCCCTCGTCGTTCTCCTCGCCATTGCCTCCGG<br>CGTCTTCTACTTCGTTTTCCGACCAAAGGCGCCCGACTATGCAATCGAGCGCGTGGCCGTGAAGGGAATGA<br>ACCTCACGTCGCCGTCGTTCACAGCGGCATTATCACCGGAGTTTGACGTCACTGTTCGGGCAGACAACGAT<br>AATAGCAAGATCGGAATCTACTACGAGGATGGAAGTTCCCTTGAAATGTTCTACAATGACGCTAGGCTCTG<br>TGATGGTGCATTGCCGGCGTTTTATCAGCCGTCAAACAACGTGACGGTGTTCGATACGGTTTTGAGGGGTA<br>ACGGCATCGCACTCACGACATCGGACCAGACAGCATTGGTGAATGATGTTGCCAAACAGAGCGTGCCGTTA<br>ACGTTGAAGCTGAGGGCACCGGTGAAAATTAAAGTGGGATCTGTTAAGACGTGGAAGATCACCGTTAAAAT<br>TACCTGTAATGTGACGGTAGATCAGTTAACGATGAAGGCTAGGATTGTTAACAAGGACTGTAGATACCGGT<br>TGGCTCTTTGGTGA
CrLEA2-59	ATGACTGATCCAGTTCATCTGAAAAACTCGCCCCCACAATCCAACGACGCACAAACGTCGCCGCCTCATGA<br>AGCTGCCGTCACCTCAAATTCCTCCTCTCTGGCGGCAACCTACGTCATCCGTATCCCCAAGGACCAAGTCT<br>ACCGCGTTCCTCCGCCGGAAAGTGCTCTTCGCTACGCCAACTACAGCCGCCGGAAAAATCGTCGATCCCGG<br>TGCTGCTGTTGCCTCTGCTGTTTCATCTCCATCGTCTTCTCCCTCGTCGTTCTCCTCGCCATTGCCTCCGG<br>CGTCTTCTACTTCGTTTTCCGACCAAAGGCGCCCGACTATGCAATCGAGCGCGTGGCCGTGAAGGGAATGA<br>ACCTCACGTCGCCGTCGTTCACAGCGGCATTATCACCGGAGTTTGACGTCACTGTTCGGGCAGACAACGAT<br>AATAGCAAGATCGGAATCTACTACGAGGATGGAAGTTCCCTTGAAATGTTCTACAATGACGCTAGGCTCTG<br>TGATGGTGCATTGCCGGCGTTTTATCAGCCGTCAAACAACGTGACGGTGTTCGATACGGTTTTGAGGGGTA<br>ACGGCATCGCACTCACGACATCGGACCAGACAGCATTGGTGAATGACGTTGCCAAACAGAGCGTGCCGTTA<br>ACGTTGAAGCTGAGGGCACCGGTGAAAATTAAAGTGGGATCTGTTAAGACGTGGAAGATCACTGTTAAAAT<br>TACCTGTGATGTGACTGTAGATCAGTTAACGATGAAGGCTAGGATTGTTAACAAGGACTGTAGATACCTGT<br>TGGCTCTTTGGTGA
CrLEA2-60	ATGGCCGACCGAGTTCATCCCAAAAACTCGCCCCCACAATCCAACGACGCACAAACGTCGCCGCCTCATGA<br>AGCTGCCGTCACCACAAAACCCTCGTCGCCGGCGGCAACCTACGTTATTCGTATCCCCAAGGACCAAGTCT

	ACCGCGTTCCTCCGCCGGAAAATGCTCATCACTACGCCAACTACACCCGCCGGAAATATCGTCGATCTCGG<br>TGCTGCTGTTGCCTCTGCTGGTTCATCTCCATCGTCTTCTCCCTCGTCGTTCTCCTCGCCATTGCCGCCGG<br>CGTCTTCTACTTCGTTTTCCGACCAAAGGCGCCCGACTATACAATCGAGCGCGTCGCCGTGAAGGGAATGA<br>ACCTCACGTCGCCGTCGTTCACAGCGGCATTATCACCGGAGTTTGACGTCACTGTTCGGGCAGACAACGGC<br>AATAGCAAGATCGGAATCTACTACGAGGATGGAAGTTCCGTTGAAATGTTCTACAACGACGCTAGGCTCTG<br>TGACGGCTCATTACCGGCGTTTTATCAGCCGTCAAACAACGTTACGATGTTCGAGATGGTGTTGAGGGGTA<br>ACGACATCGCACTCACGGCATCGGACCAGACGGCGCTGGTGAATGACGTTGCCAAACGGAGCGTGCCGTTA<br>ACATTGAAGCTGAGGGCACCGGTGAAAATTAAAGTGGGATCTGTTAAGACGTGGAAGATCACTGTTAGTAT<br>TGACTGTGATGTGACGGTAGATCAGTTAACGGCGAAGGCTAGGGTTGTTAACAAGGATTGTAGATACCGAT<br>TGGCTCTTTGGTGA
CrLEA3-1	ATGGCCCGCTCTCTCTCACAACCCAACCGTCTTGGTTTCATCGTTGCCCAATCTATCTCCCTAATACCTGT<br>TTATCGGCGAGGTTATTCAGCCGCGTCAGATGTTTTAGGAAGAGTTGGATTGGGTATTATTGCACGCAGGA<br>GTGGAATGGTGGGAAGGACAGAGGAGAAGCCTGTAACAAGAGATGGTTCGGAGGCCTACTCGGCTTGGGCC<br>CCTGACCCAGTAACGGGTTATTATCGGCCCATCAACCACACCCCTGAAATTGACGCGGTGGATCTTCGCCA<br>GATGCTTCTCAACCACAAGCTTAGATCACCCCACTAG
CrLEA3-2	ATGGCTAATTTCCTTCTCAGGAAGCGATGTTATGGTGTTGTAGCGGAATGTTTAAGGTTGGAGATGGAAAG<br>CACGATGGTGAAGGCGAAAGCAGCATCCCCAAATGGTAATTCCAATAAAGATGAAATCTTTTGGATGAGAG<br>ATCCTAAGAGTGGAAATTGGATTCCAGAGAATCATTTTGGCGAAGTTGATGCTGCCGAGCTGAGGAACAAG<br>TTTCTGCCAAGACACACAAAACTTCCCAATCAACTTAAGTAA
CrLEA3-3	ATGGCTCGCTCTTTCACTAACGCCAAGATTTTCTCTGCTGTCGTCCTTGATGGATTCCCTAGCGTTTTCAC<br>CAGACGTGGGTACGCGGTAGCAACACAAAGCGCAACAAGGGGAGGAACGGCCTCTATAAGCGGCAAGGTGG<br>CACCCAAGGCTGTGGAAGACAAAGTGGCCTCCAACAACAAGGTTTCGTGGGTGCCAGATCCTGTTACTGGT<br>TACTACAAACCCGAGAACATCAACGAAATTGACGTTGCTGAATTGCGCCAGACCCTTCTACGCAAAAAATT<br>CAACAACTGA
CrLEA3-4	ATGGCTCGCTCTTTCGCCAACGCCAAGGTTCTCTCTGCGCTCGTAGCCGACGGATTCTCCAACACTCTTAC<br>CAGACGTGGTTACGCGGCGGCGACACAAAGCGCAACAAGGGGAGGAGCTGCCTCCATTAGCGGCAAGATAG<br>CCCCAAAATCAGGGGAAGACAAAGTGGCCGCTACTGAAAAGGTGTCGTGGGTCCCAGACCCCGTTACTGGG<br>TACTACAAACCAGAGAACACCAAAGAAATTGATGTTGCTGAGCTGCGAGCAACGCTTCTCGGCAAAAAATT<br>CAACCTCTAA
CrLEA3-5	ATGGTTAGAAGCGGCATCACCAAAGCAAACATGTTGCTTCTCCGGTCATGCAGGGGAACTGAGAGAGCAAA<br>GAGTGGACTGAGCAAGTTGTTACCTGCTGCTACAGAAAACTCTGGAGGAATTTCAAAGAGTAGTGGGAAGA<br>TGGAAAATTCAGAATTGTGGGTCCCACATCCACGCACAGGAATTTATTTCCCAAAAGGGCATGAATGGGTG<br>ATGGATGATGTTCCAGAGGATGCAGCTCGTTTAAGTCAGACTCACTGGTTCAGAAATATTGATGGTGTGGA<br>CAATCCCAAATACCAAACTTGA
CrLEA3-6	ATGGCATGCTCTTTCGCACAAGCCAAACGCGTCCTCGCTTTCGCTCTTCATCGGCGAGGGTATGCAGTGAT<br>GTCTGATGCTTCGGTGAGAGGGGGATTGGATAGGATTGGGAGTGGGAGTAGGAGTAGGAGTGGATCAACGG<br>TGGTGGGGAAGGTTGAAGAGAAGAGTGTGTCCAAAAGTGGTTCGGAGGGTTCTTCAGCTTGGGCCCCAGAC<br>CCAGTCACGGGTTACTACAGGCCCATCAATCACACCAATGAGATTGACCCGGTGGAGCTCCGAGAGATGCT<br>TCTGAATCACAGAGTCAGATCTTCATCCCCTTAA
CrLEA4-1	ATGGCGTCGAGGCAAGAATTCAAGGAAGACAGAGCGGAAGCAGCAGCAAAGCTAGCTGCGAAAGACATTGG<br>CCATGTGAACAGAACAAACGAGCGTGAGGAAAGTTACACACTGCATTCCGATATTCACTTTAAGGAAGCAC<br>GAGCGGAGGCAGCAGCGAAGCTTGCTGCTAAGGATCTTGAAGACGTTAACAGAGCAAGAGAGAGTGCATTT<br>CGTGAAAAACAACGTTATTGTGATGAGCAGAAACCCGGTGTGATAGGGTCCATGTTTAGAGCCGCGAAAGA<br>AGCTGTGGTTGGCAAGCCTCACGAGGGTGTGGAAGTGAGGGATAGAGGTACCTCACCTGGGGTTTATGATT<br>ACACCACCCAGAAGACCACCGAAACAAAGGATGCAACGAAGCAAAAGGCTGGGGAATATGCAGATTATACG<br>TCTCAGAAAGCGAAAGAAGCAAAAGACAAAGCAGGGGAGTACAAGAACTATGCTGCAGATAAAATGAAGGA<br>AGCTGGAGATGCGACAATGAACAAAGCTAAGGAAGCTAAAGACGCGACGATGCAGAAGGCAGGTGAGTATA<br>AAGACTATACAGCTGAGAAGACAAAAGAAGGGACGGAGAGTGCAGCGGGGAAGCTTGGGGAGCTGAAGGAT<br>TCTGCTGCTGATGCAGCCAAAAGGGCTATGGGTTTCTTCGCTGGCAAGAAAGAGGAAACAAAGGAAAAGCT<br>GAGCGGAACAGAGGAAGAAGCGATGAGGAAAATGGAACATGTGAAGGTGGAAGATAGGAAATATGGAGGAG<br>GAGGAGGAGGAAAGATGGAGATAAAAGTGGAAGAGTCTCGGCCAGGGGCAATGGTGGATGTGCTACGGGCT<br>GCAGATAAGGCTCTGAACAATGTGGGACGGTTGGATGAAGAAGGTGTGATTCATGTGGAGCGTGGTCGTGA<br>GAAAATGTGA
CrLEA4-2	ATGGCGTCAAGGCAACAACCCGAGGACAGAGCCGAGGCTGCAGCCAAAGTTGCTTCTAAAGAACTCGAGCA<br>AGTTAACAAAGCCAGAGACCATAATTTGGCTATCGAGGACCAACAACAACATGACCAAGAAAAGCGTGGTG<br>TAATTAGCTCCGTTCTAAGAGCAGTGCAAGGAACCTACGAGAACGCAAAGGGAGCAGTGGTTGGCAAAAAA<br>GACCCTGCGGATCCCTATACCACAGAGGTAGTTCATCATGTTAACGTTGAGGATGAAGATGCACGAACCGG<br>AGAAGTTAGAGACATATCGCCAAACAAAGCAGGTTCTAAAGTTGGAGAGTATGCAGATTACGCATCTCAGA<br>AAGCCAAGGAAACAAAGGATGCAACGGCGCAAAAGGCGGGAGAGTACGCCGATTATGCATCTCAGAAGGCA<br>AAGGAAGCTAAGGATAAGACAATGGAGAAAGGTGGAGAATACAAAGACTATGCCGCAGAGAAAGCGAAGGA<br>AGCGAAAGATGCGACAATGAATAAGGCAGGGGAGTATAAGGATTACACCGCTGAGAAAACGAAAGAAGGAA<br>AGGACACAACGATGGGGAAGCTTGGAGAGTTAAAGGACTCTGCTACTGATGCTGCTAAGAGAGCTGTGGAT<br>TACTTGAGCGGAAAGAAAGAGGAAACAAAAGAGAAGGCTGCAGAAACTGCAGAGGCGACCAAAAATAGAAC<br>GTCGGAGACTGCTGAGACGGCGAAGAAGAAAGCTACGGAGATGAAAGATGCGGCAAAGGAAAAAGCTGCAG<br>AAACCACCGAGGCGACCAAAAATAGAACTTCCGAGACTGCTGAGACGGCAAAGAAGAAAGCTGCGGAGATG<br>AAAGATGCGGCGAAGGACAAAGCTGCAGAAACCACCGAGGCGACCAAAAATAAAACTTCCGAGACTGCTGA<br>GACGGCGAAGAAGAAAGCTACGGAGATGAAAGATGCGGCGAAGGACAAAGCTGCAGAATCCACCGAGGCAG<br>CTAAGCAGAAAACTGCAGAGGCCAAGGACAAGACGAAGGAAAATGTGAGTGGAGGAGGGGAAGAAGATACT<br>AGAAGGAAAATGGAAGAACTCAAGGTGCACGGGAAAGGATACGGTGAGGAACCTGAGCGGCGTGGTGGAGA<br>TAAGGTGGTGTTGAGAGTCGAAGAAAGTCGACCGGGAGCGGTGGCGGAAACGCTGAAAGCCACGGATCAGA<br>TGACGGGGCAGACCTTCAATGATGTGGGAAGGATGGATGATGAAGGGGTCATCAATGTGGAGCGACGTAAC<br>AAATAA
CrLEA5-1	ATGGCATCAAAACAACAAAATCGACAAGAACTTGACGAGAGGGCAAAGCAAGGAGAAACTGTAGTTCCTGG<br>TGGCACTGGTGGCAAGAGCCTTGAGGCTCAACAACATCTTGCTGAAGGAAGGAGCAGAGGAGGGCAAACAA<br>GGAAGGAGCAGCTGGGAACAGAAGGGTACCAAGAGATGGGTCGCAAAGGTGGATTGAGTTCTATGGACAAA<br>TCAGGTGGAGAACGTGCTCAAGAAGAAGGCATTGACATTGATGAGTCTAAGTTCACTACTGCTTCTAGGAA

	CTAG
	ATGAACCTTTCTTCTTTCACTTGTGTGAGCAATCATAAAGTTTTTCCCTTGGCTAACACTCGTAATTATCA
	TCCTCATTCAGTTCCCTTGCAAAGGCAAAAAAGAGTCCTTAGAAGAAAAGGTTTGAGTGTGAATAACATGG
	CATCTCAGCAACAAAACCGTGAAGAGCTTGATGAGAAGGCAAGGCATGGTGAAACTGTGGTTCCTGGAGGC
	ACTGGTGGGAAGAGTCTAGAAGCTCAAGAAAACCTTGCTGAAGGAAGGAGCCGTGGAGGGCAAACAAGGAA
	GCAGCAGCTAGGATCAGAAGGGTATCATGAGATAGGAACCAAAGGAGGGCAAACAAGGAAGGAACAGATGG
	GAACAGAAGGGTACAAAGAGATGGGGCGCAAAGGAGGGCTTAGCACTATGGACAAGTCTGGTGGAGAGCGT
CrLEA5-2	GCTGAAGAGGAAGGCATTGACATTGATGAGTCTAAGTTTAGGAATCACTAA
	ATGGCAAACAACGGGAAGCAAGTGGAACACAAAACAGGAGAGTGCCCTAATCGCAAAGAGAAAGGAAAGAT
	AGAAATGTTGCCTTTGGAAATTAGTCCATGCGTTAAATACAATGATGTACAAAAAGAATACAATCGCAATG
	CTTATGACACACAACGCCATCTTCAGCCAAAACCAAACCAAGCAGGTGGAACCACACACATTGCTCCTACT
CrLEA6-1	CCCTCAGATGCTCCATTGAAATTAACCACTAAATAG
	ATGGAGAATCAAACTGAGAAAAAGGCAGAGAGTGAATCAGGTAAAGAAGGACTTCCGATGAAGGATAGTCC
	TTACCTGCAATACAAGGATGTGGAGGACTATAAGCGTCAAGGTTATGGCACCCATGGTCATCAAGAACCAA
	ACCCAAATCAAGGAGCTGGTGCCACTGAGGCACCCACTCTCTCTGGTGCTGCTGTTTCCTCTGAATCTGAA
	TTCAAAGCCACTGATGCCATTAATCGCAAGGGACCCGATGCATCTAAAGCTATGGTAGAGAGCAGCATCAA
	ACCAAACCAAACCCGTTCCTTTCGTTACATGGCGACAAAGAACGTGAAAGGAAAATGGAACAAAGCGGAAG
	AAGAAGAAGATGAGGAAGAGGAAGAAGCATTGTCTCTTTGCGATCTTCCGATCAACCTGGAAGCCCAACCG
	AGAAATCAAGAACACAAACATGTCAACGAAGCAACACAAGAAGAATTCAATTTCCGCTCTTGGAGTGGTTT
	CTTTTCCAAAGAACCAGAAATGTGTGTCGCGGATGAAGTTTTCTTTAAAGGTCAAATTCTCCCAATGCGTC
	TCTCGTTCAGCACCGAGGCTGGTTTATTAACCACAGGATCTCAACATTATGCTAAGCGATTTATCAACGAG
	TCCTTGGATCATGCTTCTCTCGGTGATTTTCAAAGTAATAATAGCAGTAGAAGCAGTAGTGTTAGAAGTCA
	AAATTCGTCAAGTAGCACCAGCTCCACCACCACCACCACCACCACTACAACAACAGCAGCAGCACCACCAC
	GATTTAAACCTAGCGTTAGAAACCAATTTCACACGCACCCTAGTCCGAAACCTCAATTAAGAGTATCTGCT
	CCAAGACAATTAGTTAATCAAGGTAGAAAATCATCATCAGCATGGGAAATTTTCCGTTTGGGTGTGGTCCC
	TGCTCCCGAAATAGGATTGCAAGACCTCAAGGTTCGCAGTAGCAACAGTGCGAACAGGAACTGTGTGAGTC
	GCAATAGTAGTAGCAGTAGCAACAACAGTGCTTCCAATAGTAATGCGAAAAGTGTGAAAACGAGTAATGAT
	GGCAACAAGAGCAATCATGTTTTGAAACAGTTAATTGGCAAAGGCGGTGGTTTATTGAGTGGTTGTGATTG
	TTCCTTTGAAACGGTGCAATCGAACATGGTAATGATCAAAGGTGGTAAAAGCACCAACAAGGCAGAAAGTA
	CAACGCACGCCGTGAAAGAAAAAGTGGTGGAATTGAAGAAGCAGAAGCAGAGGCAAAAGCAGGGAAAGAAG
	GTTATGTCACGTCGTCGAACGTTTGAATGGTTAAAGGAGCTTCATGCAAGCCATGATCCTGATGATGAGAA
CrLEA6-2	GGCTTTGTTATCAAAACCATCATGA
	ATGGCAAATTATCAGAACCAGTACGGTGCGGTTCCTACAAGCAAGGATCTGATTCAGAAGGAAATGGACAC
	CACCGGAGACACCACTGGCGTTGGCGGTGGCAGTGGCAGTACTAAAGTGGTAGGAGATGATTATGATAGAA
	GCAGCGCTGACACTACGGGTGTGAGCGAGCAGCATCACAAGAAAGGAATAATAGAGAAAATAAAAGAGAAG
CrDHN1	CTTCCTGGAACACATCATCACAACAAGTAG
	ATGGCAGAGGAAAATCAGAACAAGTTTGAGACCACTGAGAGCAGTGAGGGGGAGATCAAGGATCGTGGTCT
	TTTCGATTTCCTCGGTAAGAAAAAGGAAGAAGAAAAGCCTCAAGAGGAGGTCATCGTCACCGAGTTTGAGA
	AGGTCACAGTGTCAGAAGAGAAAAAAGAAGACGAAGGAGAGAAGAAGCACAGTCTCTTAGAAAAGCTTCAC
	CGATCCGACAGCAGCTCAAGCTCTTCGAGCGAGGAGGAAGGAGAAGAAGGGGAGAAGAAGAAGAAGAAAAA
	GAAGGAAAAGAAGGGATTGAAGGAGAAGATCGAGGAGAAGATAGGGGATGATCACAAGCACGAGGACACAA
	GTGTTCCAGTTGAGAAAGTTGAGGTGGAAAAAGTTGAGGTTGAAAAAGTTGAAGTTGATCCAGCACACACT
	GAGGAAAAGAAGGGTTTCCTCGACAAAATTAAGGAGAAGCTACCAGGTCACAAGAAGACAGAGGAGGTTCC
	ACCACCACCACCGCCACCTGCTGCTGCATCATCAGAACATGGTGAGGGTGCTCATGAGAAGAAAGGTATTT
	TAGAGAAGATAAAAGAGAAGCTTCCTGGTTATCACCCCAAGACAGAGGAAGAGAAACAAAAGGAGAGTGCC
CrDHN2	GGTGACTGA
	ATGTCTGAAGCCCAACTACGAGACCAACATGGCAACCCTGTCCAATTCACCGATCAATACGGTAACCCGGT
	TACATTGACCGATGAGCATGGTCAACCCGTCCAGCTCAAGGGCGTGGCAACCACTGGTGGTGCAACCACGG
	GCACAGCAGGATCTGGATTTGGAACATATGGTACTGGTGCATACGGCGGTGGTGCAGCCACAGGCACAACC
	ACCGTCGCAGATCTGATAGCAACCGAGCGAGCGACTGAAATGCCAACTGCGCAGCATCATCAGAGGGAGCC
	TGGAGAGCTTCGTCGTTCCTCTAGTTCAAGCTCTAGCTCGTCTGAGGATGATGGCCATGGTGGGAGGAGGA
	AGAAGAAAGGACTGAAGGATAAAATAAAAGAAAAGCTACCAGGGGTAGGAGGTGAACGAAAAGATCATGCA
	CACACACCTACAACCACCACCACTACATCCTTTGGTACTGGTGCTACCCACCCACCGCCACCCACCGCCAC
	CCACCCTCATCAGCATGAGAAGAAGGGCATAATGGAAAAGATTAAAGAAAAATTCCCTGGCCATCACACCA
CrDHN3	ACTGA
	ATGGCAGAGGAGAAGCTGAACAAGAGTCATGAGTACGATACCACTTCCACCAATACTGAAACTGAGATCAA
	AGACCGTGGAGTTTTTGATTTTCTCGGTAAGAGAAAGGAAGAAGAGAAGCCTCAGGAGGAGGTGCTAGCCA
	CAAATTTTGACAATAAGGTCAAGCTTTCCAATGAACCCGAGACCAAGCTCCAAGAGCAAGAAGGAGACAAG
	AAGCACACCCTCTTAGAGAAGCTCCACCGATCCAACAGTAGCTCCAGCTCTTCCAGTGATGAGGAGGAGGA
	AGGAGAAGGTGGGGAGAAGAAGAAGAAGAAGAAAAAGGAAAAGAAAGGGCTGAAGGAAAAGATAGGAGGTG
	ATGATCACAAGGAACAGGACACTGCTGTTCCAGTTGAGAAAGTGGAGGTTGATTCAGAGCACCCTGACACT
	GAGGAAAAGAAGGGTTTCCTTGACAAGATTAAGGAGAAGCTGCCAGGTCAGCACAAGAAGGCAGAGGAAGT
	GTCCCCGACATCATCGGAATATGTTGCTGCACACACTGCTGAGACTCATGAAGGGGAGGCTAAGGACAAGG
	AGAAGAAGGGCCTTTTAGATAAGATCAAGGAGAAGCTTCCGGGTTATCACTCCAAGCCAAATGAAGACAAA
CrDHN4	GAAAAGGAGACCGATACTCATTGA
	ATGGCAAGTTATCAGAGTCAATACGGTGATCAAGGTCGTAAGACCGATCAGTATGGAAACCCTGTGACTCA
	AACTGACCAATACGGTAACCCTGTGAGACAAACCGGTGAAACCGGTAAGCAATATGGAACCACCGGTTACG
	GAGCTAACACTGCGGACACTGGCTCGGGTCATAGAAGTGGAACCACTGGTGGCTATGGAACTCGCACTGAG
	GGTGTAGGCGCGGGTTATGGAACCACTGGTGGCACCACCACTGGTTATGGTGCCACTGATTATGGAAACAC
	CGGTGGCACCACTACTGGTTATGGTGGCACTGATTATGGCACCACCGGTGGCACCGGTGGTACCGGTTATG
	GAAGTGGAACAGGAACTACCGGTGGCACTGGTTATGGAAGTGGAACCGGTTATGGAATCAACACTGGAGGT
	GCACATACTGATGCAGGGTATGGGAGGGAGCATCGTCATGGACATGACCAGTCTCATGGTGGGTATGATGC
	ATCGGGGGAGCAACATCATGAGAAGAAAGGGATAATGGATAAGATCAAGGAGAAGCTTCCTGGAACTGGAG
CrDHN5	GACACAACGAGAAGTAG
	ATGCAATCTGCCGCCGCCGTCAACACCGACGCTGGCATCGTTGGCCGGCAAGACATATCGGACGTGGCTAG
CrSMP1	AGAGCAAGGCGTGAGCGTATCCCAAACAAAAGTGGGTGTGAACCGTGTGATCACAGAGTCGGTGGGAGGGC

	AGGTGGTGGGGCAATTCGTGGAACCTGATGTCCCTATGAATTCCCCTGGTATGGCTCTCGAACCAGATGCC
	ATAACCATTGGAGAGGCACTGGAAGCGTCGGCGGTGGCTGGAGCGGGTGATAAGCCTGTGGACCAAAGCGA
	CGCCGCTGCTATACAAGCTGCTGAAATGAGAGCCACCGGAAAGAATGAGACAGAGCCTGGTGGGTTGGGTG
	CGAGGGCCCAATCAGCTGCCACTCACAACACTCGCACTCCCTCTTACTTTCATAAAACCACTTTGTCTCAT
	GTCTTGGCGGATGCTAAGGAGAAGTTGCCAACGGACAAGCATGTAACGCGTGAGGATGCAGAGGGTGTCAT
	TGCTGCCGAGCTTAGAAACAAACCCGACATGAAAACCACCCCTGCCGGGGTTGCTGCATCCGTAGCCGCTG
	CCGCCGCCATTAATCAAAACGCGTACCTTACAATAATATTATAA
	ATGAGTCAAGAGCAGCCTCGGAGAGAGGAAGCCATCAAATATGGAGATGTGTTCAATGTGGAAAGAGAGCT
	TGGAAGCAAAGCAGTGGCACCCGTCGACGCAGCGATGATGCAGAAAGCAGAGACTGCTATGATTGGGAAGA
	CCGAAAAGGGTGGTGCTGCTGCAACCATGCAAGCTGCGGCATTGAAAAATGAGAGAGATGGCGTTGTTGGA
	CATAATGATATCACTGAAGTTGTTGCACAGAGTGGTGTCAGTGTAACTGAATCTGATCTTCCAGGGAGGCA
	AGTTATTTCGGAGTCAGTTGGAGGACAGGTTGTTCACACACTAGAGGAAAAGGAAAGGAACATTTGTAAAT
	TAGTTGAGGTGGTGGAGCAATTTGGGCAAAAGGTTGCATCGAATACGACGATGCCATCTTCTCTTGCTCAA
	GACTTGGGCTCAAGAGTTGGTGGTGGGATCACCATAGGTGAAGCATTGGAGGCGACGGTTTTGACGGCGGG
	AAAGAAGGCGGTGGAATGGAGCGATGCGGCGGCGATTCAAGCAGCGGAAGTGAGAGCCACCGGACGTACCA
	ACATTGTTCCCGGAGGTGTTGCTGCGGCGGCACAATCAGCAGCCACCCTGAATGCTCGAACTACAAAGGAC
	GAGGACAAGACCAAACTCGCAGATGTTTTGTCGGATGCGACTTCGAAATTACCATCAGACAGACCAGCGAC
	GAGGCGAGATGCTGAAGGGGTAACGGGTGCAGAAATGAGAAATGATCCTAACCTCACTACTCATCCAGGGG
CrSMP2	GCGTGTCCGCATCCGTCGCTGCTGCTGCTAGGCTTAACCAAACCAATAATAACTAA
	ATGAGTCAAGAGCAGCCAAGGCACCCCCAACCCGCCCAAGATCCCATCAAATACGGTGACGTTTTCGATGT
	CTCCGGCGACCTCGCAAAGAAGCCCGTTGCACCCGAAGACGCCGCCATGATGCAGAGCGCCGAGACCCGTG
	TCCTCGGCCAGACCCAAGCCGGGGGAGCAGCTTCCGCCATGCAATCCGCCGCCACTCGGAACGAGCAGGCC
	GGCGTTGTCGGTCACCGAGATGTCACTGATGTCACCGGCGACCGTGGTGTCACCGTCTCGGAGACTCAAGT
	TCCCGGAAGACGCATAATTACGGAATCAGTCGGTGGACAGGTTGTGGGACAGTATGTTGAGCCGACTCCGG
	TTCAGGCGGGACGAGCCGGTGCGGTCCGAGACAGTGCGATAACCATAGGGGAGGCGCTGGAGGCGACGGCG
	CAGACGGCGGGTGAGAAGCCGGTGGATAAGAGTGACGCGTCGGCGATACAGGCGGCGGAGGTGAGAGCGAC
	GGGCAGTAATGTTATAACGCCGGGAGGGCTTGCTTCTATGGCTCAATCGGCGGCGGCCTTCAATGCTGAAT
	GCAAACGTGACCAGGAGAAGGTCAAGCTCGCCGATGTATTGACGGGGGCCACCGCGAAGTTGCCGGCGGAC
	AAGGCGGCGACAATTGAAGATGCTGAAGGAGTGGCGTGTGCTGAGGTGAGGAACAACCCAGATGCGACGGC
CrSMP3	CACTCCTGGTGGCGTGGCAGCCTCTGTGGCTGCAGCTGCTAGGCTCAATGAAAATGTTAATGTGTGA
	ATGTTCACAACCACTATTTGGAAGTTCCTCACAATCTCTTTTCGATCAGAAACAATGAGTCATGGCCAGCC
	CAAGAGACCCCAAGCAGAGGATTCTTTTCACTGTGAACCCATCAAATACGGCGACGTTTTCAAAGTTACCG
	GCGAGTTGGCGTCCAAGCCGATTTCACCACAGGAAGCCGCCGCCATGAAAGCCGCCGAGGCTGGCGTCTTA
	GGGCAGCCGCAGAAAGGAAGCCCAGCCGTAGTCATGCAGGCAGCTGCGGCAATCAACCAGGCGTGCGGTGC
	CGTTGACGGCAACGGGGTCGCCATCAAGAAGGAAGACGTTAGTGTAACTGAAACAACAACCGCTGGCAACC
	GTATCATAACAGAATCTGTGGGTGGCCAGGTCCTCGGGAAGCGTGTGGACACTCACGCGCCTGTGGTCCCC
	ACGGATTTGGGTTCTGCAGTTATAGATGGCGACCCGATAACTATTGGAGAAGCTCTGGAAGCAGTGGCCAT
	AGCGGTTGGGGATAAACCGGTGAACCAAAACGACGCCGCTGCGATCAGTGCCGCGGAGATTAGGGCGACCG
	GCGAGAAGAACGTAAGGGCCGGTGGAGTGGGGGCATCGGCACAATCGGCAGCCACTCTCAACAGCCACGTC
	ATGCGAGTCCAAGATATGACAAAACTTTCTGATATATTAACGGATGCGACGGATAAGCTGGCCATGGATAA
	GGCGGTGACGCAAGAAGATGCTGAGGCGGTGTATACAGCGGAGATGCGGTTTCCATGGCGGGGGGATGCAA
	CGGACGTGATTTCTGAACCCGGTGGAGTGGCCGCATCCATGGCCACTGCAGCAAAGCTGAATGAAGAAAAA
CrSMP4	TGA
	ATGGCTGAAGAGAAACACCACCACCACCACCACCTCTTCCACCACAACAAGGACGAGGATAAGCCTATAGA
	AACTGAGAGTGGTTACAACAAGACTTCAAAATACACTAGTAATGAACCTTCCGTTGGCTATGACTCTGGTT
	ACAACAAGCCATCGTATGAGTCTTCTGGTGATGCCTATGAAACTGGTTATAACAAAACATCATATGACACT
	TCTGGTGGTGCCTATGAAACTGGTTACAATAAAACATCATATGATTCTTCTGGTGGTACCTATGAAACTGG
	TTACAACAAAACAGCTTATTCCACTGATGAGCCTTCTGCTGGCTATGGTGGTGGTAGATACTCTGACACCA
	CTGGTGGTGCCTACACCAAAAAAACCGGTGAATATGCTGCCGGTGGTGGCTATGGCGATGATAGTGATCGA
	CGTGATGATGTTGACTATAAGAAGGAGGAGAAGCACCACAAGCATCTTGAACACCTTGGTGAGTTAGGTGC
	TGCAGCAGCTGGTGCTTATGCCTTGCATGAGAAGCACAAGTCAGAGAAAGACCCAGAGCATGGTCACAGGC
	ACAAGATAGAAGAGGAGGTTGCAGCAGCGGCTGCAGTGGGAGCTGGTGGCTTTGCCTTTCATGAGCATCAT
CrASR1	GAGAAAAAGGAAGCAAAGGAACAAGATGAGGAAGCACATGGAAAGAAGCACCACCACCTGTTTGGCTGA
	ATGGCTGAGGAGAAACACCATCATCACCACCATCACTTTCACCATCACAAGAATGAAGAACAACCTGTTGA
	AACTACCGTCTACTCCGAAACCACATTTGATGTTGCTGGAAATGTGGATGACTATAAAAAGGAAGAGAAGC
	ACCACAAGAATCTAGAACACCTTGGTGAATTGGGAGCAGCTGCTGCTGGTGCTTATGCTTTGCATGAGAAG
	CACAAGGCAGGTAAAGATCCAGAGCATGCCCATAGGCACAAGATAGAGGAGGAGATTGCAGCTGTAGCTGC
	GGTTGGGGCTGGTGGGTTTGCATTCCATGAGCATCATGAGAAGAAAGAAGCCAAAAAACAATATGAGGAAA
CrASR2	CACATGAAAAGAAGCATCATCACCATCTTTTTGGTTGA
	ATGGCTGAGGAGAAGCACCACCACCTCTTCCACCACCACAAAGATGAGGATAAGCCTGGCTATGTTGATGA
	GGTTGATTATAAAAAGGAAGAGAAACACCACAAGCATCTTGAGCACCTTGGGGAACTAGGTGCTGCAGCTG
	CTGGTGCTTATGCTTTGCATGAGAAGCATAAGGCTAAGAAAGAGCCAGAACATGCTCACAAGCACAAGATA
	GAAGAGGAGATTGCGGCAGCAACTGCTGTTGGAGCTGGTGGTTTTGTCTTCCATGAACACCATGAGAAAAA
CrASR3	AGAAGCTAAGAAGGAAGATGAGGAAGCTCATGGAAAGAAGCACCATCATCTGTTTGGCTGA
	**Genomic DNA**
	ATGCAGGGAGCAAAGAAAGCAGGAGAATCCGCTAAGGAAACTGTTGCCAACTTGGGTGCTTCTGCCAAGTC
	TGGCATGGAGAAGACCAAGGCCACCGTCCAGGAGAAGGTATACTTATTATTCAAATTTTAATTATGTTCAA
	AATTATTTTTTTAATGATATAATAATAAAATTTTAGTTTAAATATAGTTTTGTTGTTGATTTTTCATAATA
	ATTATTTTTGAAGTTATATTTGTTTGATAGTATAAAAATTTAATTTATTATTATTTTCTAATGACAACACA
	TTATATATTATTATGGTAAAATTAATAATAGAGTTTTAGAATTGAACTTTTTTATTATCATTGTTCATAAA
	ATATCTTCAGGCAGCTAGTCTAGTGGGTTCATAATACATTTTATAATGAGAACAACAAAGCTATTTAACAC
	CGAAACGTGGTCATTGTCCATGATGCTAATTAGAGTAATTCAGTTATCAGCCTTTTCAATCAACACATATG
	TCATCATATATATTGCATGGGCAACTTCCGTTTTCAAGTAACCATGTGACGAAGTATATATATATATATAT
	ATATATATATATATATATATATATATATATATATATATATTTATCAAAATTTAACGATGAAAGCTTTGGTT
	TAGAAAAAGTTTTTGGAGGTGCAAGTGATGGGTCACATCATTTGTGCTGTCAGTGTGTGTTGCTTACTCTG
CrLEA1-1	ATTCTATGTATATAGCTAATAAATGTGTTTGGGTTTAAACAGACAGAGAGGATGAGGGCACGTGATCCTTT

	GCAAAAGGATTTGGCAACTCAGAAGAAAGAGGAGAAGATCAACCAGGCGGAGCTGGACAAACAGGCCGCGC<br>GTGAGCACAACGCGGCGGCAAAGCAGTCAGCCACGGCCGGGCACATGGGACAGGGCCACCACACCACCGGA<br>ACTGGGACCGGAACCGCCACGTACTCCACCACAGGACAACACGGGCAGCCCATGGGGGCCCATCAGACGTC<br>AGCGATGCCTGGCCATGGAACTGGGCAGCCCACGGGCCATGTTACTGAAGGAGTCGTGGGCTCACACCCCA<br>TTGGAACCAATAGAGACCCGGATGGGACTGCTACGGCCCATAATACCCGGGCGGGGGGGAATCCAAATGAC<br>TATGGGTATGGGACTGGGGGTACTTACACTTAA
CrLEA1-2	ATGCAAGCAGCGAAGCAAGCGGTTGAGAACATTAAGGAAACCGCAGCCAACATTGGCGCCTCTGCTAAGTC<br>TGGCTTGGAGAAGACCAAAGCCAGTATCCAAGAGAAGGTTTTCCTTTCCTTTTTCATTTTTCAAAATTAAA<br>ATTATTCATATATTTTTCTTTTTTCTTTCCTGCTTAATTCATTTTTGTCTTCTTATCAATTTCCATTCATC<br>AACATTGTTGTTCATTAGCCTTACTTAAAGTTGTTATTTTGTTATATTATAAGATCTATTGTCATAATATC<br>ATATTATAGAAGAAATCGTTATAATATCATTGATGATTCTTAAATAGAACAATCATAGTTTCAATTAATTT<br>TATATTAGAGATTAAATTGAATGCTAATAGGTCAATCAGTCTAATAAATTGAAAGATTGATGACTTCTGTT<br>CTTTGGGCCGCCATTGATTACTGCAATATTTTGAACTCATGAATGTGATTTGCATTTGGGCCTCAACAGAC<br>TGAGAAGATGAGTGCCCATGATGAAATTCAAAAGGAGATCGCAACCCGAAAGAAAGAAGAGAGGATCAACC<br>AGGCTGAGATAGAGAAACAACAGGCACGTGAGTACAACGCCGCCGCCAAACAATCTGCCATGGCTGGGCAC<br>ATGAGTCAGGCCCACTATACTAACTCTGGGCCAGGGACTGAAACTGCCACAGATCTCACAATTGGGTCCAG<br>GCCTGAGACTGATGCTACTTACTCCACTACTGGGCCCGGGCCTGAGATTCCTACGACATATTCTACCACTG<br>GGCCTGGAATTGAGACCGCCATATTCCCTGCCACTGGGTCCGGGCCTGACACTGCAACACACCCCATTGGT<br>GAGTATGCACAACCTATGGGGATAACACCTATGGGCGTGGTAGGATCAAATCCGTCTGGGACTAACACTGG<br>CGTAGATAGTACTGCTCCTATGAGTGGACATAATTTCAGTTGA
CrLEA1-3	ATGCAATCTTCAAAGGAGAAGCTGCAAAACGTGGCCGCTGCTGCCAAGGAGCACGTCGACATTTATAAAGC<br>CAAAATAGACGAAAAGGTCTGCTTTTTCATGAACTTATCCATCTCTATACGCATTATTGAGTCTCAAATTA<br>TTTGATGTTTGTTGCATGAATTGTACGTGGTGGAGTTAAAATCATTTTATCTTCACATTTATCGTAGGTAC<br>TTTCCTAAATGATTTCAAGAATTATTTGGCAATTCACTTTGGATTTAGTTCGGTATAATAATCTATCGGTT<br>TTTTTTTTAGAAGATCGTTCAAACTGCAAAAGTATTTGAAGTTGAAGAAATGTTACTAACAGATATTTTTA<br>AAACACGTTGTTGAATTCAATTTTGATGAGTAATTTTTTAAAAGTAAAATTATTTAAAAGTTAATAGTAGT<br>CTTACTGGGTTAAATGCTGCAGGCAGAGAGAGCAACGGCAAGAACCGAGGAGGAGAAAGCAATAGTCCATG<br>AGCGTGCAAAGGCAAAGGAAGCTAAAGCAAAGATGGAGCTGCATGAAGCTAAAGCCAGGCATGCCGCCGAA<br>AAGCTAAGCAACAAACAATCACATTATTATGGTCTCCACGAGCACGAGAGTCCCGTAGTTGGGGCTCAACA<br>ACAACCACCCTTGGTAGGAAATCAGACTCAGTACCAGCAAGGGCACCACTCACTTGGAGCAGTTCCCATGT<br>CTGGAACCACTTATCCATCTTATCCACTGGGAGGAAACCCTCCCAGCAGTAAGCATATATGA
CrLEA2-1	ATGCATGCTAAGACTGACTCAGAGGTGACAAGCTTGGATGCGTCGTCCACCACACGGTCCCCTCGGAGAGC<br>GGTGTATTACGTTCAAAGTCCATCTCACGATGGGGAGAAAACGACGACGTCGTTGCACTCTACTCCTGTGC<br>TCAGCCCCATGGGATCCCCTCCTCACTCTCACTCTTCTTCAAGTCGATTTTCCGGTTCACGAAAGATGAAC<br>CATCATCGCAACAACAAGACTTGGAAAGACATTGACGTCATCGAAGAAGAAGGCCTTCTTCAATCCGAAGA<br>ACGTGACCGTACTCTCTCTAGAAGATTCTATTTCCTCGCTTTTGTTATTGCCTTCTTCCTTCTCTTCTCTC<br>TCTTTTCCCTTATACTATGGGGTGCTAGCAGGCCCATGAAGCCCAAAATAGTCATCAAGGTACTTTAAACT<br>TAATTTCACTTTCTATAAATCTTCCCCTCTCCTTCATTCAATTCATTCACAATTTTTCCATGTGCCTTTGC<br>CAGAGCATTAAATTTGATCATCTCAGAGTTCAAGCTGGTTCTGATTCCACCGGAGTCGCCACCGACATGAT<br>CACCATGAATTCCACCCTCAAATTCACGTATCGCAACACCGGCACATTCTTTGGGGTTCATGTCACTTCAA<br>CACCATTGGATCTCTCCTATTCAGAAATCGTAATTGCCACCGGCAATGTAAGATTCATCTATATATATATA<br>TATATATATATATATATATATATATATATATATATATATATGTATGTATGTATATGCTTCTGAATCATCAT<br>AGTTCTTGTCTCTTTATAAACAATAATGTGATGGCTGAATTTGATTATGCAGATGAACAAATTTTATCAGT<br>CAAGGAAGAGTCAGAGATTGGTGAGTGTTGCAGTGATGGGAAACAAGATCCCGTTGTATGGAAGTGGTGCT<br>AGCTTGAGTAGTTCAACAGGTATGCCAACAGTGCCTGTGCCTTTGAAATTGAGCTTTGTGATTCGATCCAG<br>AGCTTACGTGCTTGGAAGATTGGTGAAGCCAAAGTACTATAAGAGGGTTGACTGTTCCATCACTTTGGATC<br>CGAAGAAGATCACTCTTCCACTTTCACTCAAGAAGTCTTGCACATACGATTGA
CrLEA2-2	ATGGCGGATGGACCCCCGGCGACCACCGCCGCCACGAAGCCAAGGAGGCCACGGCCACCGTCTGGACGTAC<br>CAACCTGGCCTCCTGCGTGGTCGCCACCATCTTCCTAATCTTCATCATCATCGTCATCCTCATTGTCTACT<br>ACACCGTCTTCAAGCCACAGGACCCCAAGATCGCCGTCAGCGCCGTTCAGCTCCCATCATTCTCCGTCGTT<br>AACGGCACCGTCAACTTCACCTTCTCCCAGTATGCTTCCGTCAGAAACCCTAACCGCGCCGCTTTCTCTCA<br>CTATGACAGCTCCCTCCAGCTGCTCTACTCCGGCAGCCAGGTCGGGTTCATGTTCATCCCCGCCGGCGAGA<br>TCGACGCCGGTCGGACACAGTACATGGCTGCCACCTTCTCCGTTCAGTCCTTCCCCCTGTCGGCGCCAGCC<br>AGATTGGGGCCCACACTGGCCAATGGCGATGGTGTTGGGTTTAACTATGGGCTTAGGGTGGAGCCCACGAT<br>GGAGATCGAGTCCAAGTTGGAGATGGCGGGGCGCGTCAGGGTTTTGCACTTCTTCAGCCATCACGTTCACG<br>CCAAAGCCGGTTGCAGGGTTGCAATTGCCGTTACCGATGGATCTGTGTTAGGTTTTCATTGCTAA
CrLEA2-3	ATGGCTCAGAGTCCAGAGCAGGTTAAGCCCTTAGCTCCATTCATATCATCAAATTACCTCAGCAGACAAGA<br>AGATCAAAACATCTCCGAAAGAAAAATCATTCGCATAAGAAAGTTCGTCCGTTGCTGCGGCTGTGTCACTG<br>CCCTTGTTTTGATATTTGTAGTCATAATATTAGTCTTGGGCTTCACAGTTTACAATGTGAAAGAACCTGAA<br>GTAAGGATGAACAGGGTTATCCTTCTCAATAGAACTTTAGCTAACGGTGCTATCAATGACGTTACACTTCT<br>TGCTGATATCTCTTTGAAGAATTCAAACGCTTTCACCTTCAGGTTTGGATACACCAACACCACTGTTTACT<br>ATGATGGCACGGAAATAGGTGAGGGAACATCTCCACCGGGTAAGGCCAAGGCAAGAAGGACAATAAGGTTC<br>AACGTGACGATGGAAATTATGGCAAAGAAGCTTTTGGCTGTCCCAAGCTCGGAGAGTGACATCAGAGATGA<br>TCAAGCTTTGAATATCAGCAGCTATACCATGGTAGATGGTAAGGTCAAAATATACAATCTGTTTATGAAGA<br>AGGTTGTGGTTGAATTGAATTGTACAATTCAATACAACATCACCACTGGTTTGACCTCCTCAAACGGTGGC<br>AATTGCCTCGGACTCGTTGATCTTTAG
CrLEA2-4	ATGGCCACCAATATGCCTAAATTCAAGCGAAAACTGGGAATGGGTGCAGATGGCCGCACCAACCCCATAGT<br>CTGGCTAATAGCCATTATCTGCACCATCATAGCCGTAGCAGTGGTAGTTGCAGGCATTGCAGTGTTTATAG<br>GTTACATAGTGATCCACCCCAGGGTTCCTATCATCAGTGTCACAGATGCTCATTTAGATTTTCTGAGAAAT<br>GATTATGCTGGTCGACTCCAAACCCAAATCAGAATTGTTGTGACTGCAAAGAATGGGAATGCCAAGGCTCA<br>TGCAACATTTTCTAAGATGACTTTCAACATTAGCTACCAAGGCCAAGATATAGCTGTGCTTGTTGCAGACC<br>CATTTGAGGTGCCCAAGAACAGTTCCAAAGAGCTCCATTATGTTGTACAATCATCCTCCATACCATTAAAT<br>CCTGATCAGATGGAGGAAGTGACTAATGCATGGAAACAGAATGAGATTGAGTTTGATTTCAAAGGAGCTGC<br>AAGGACACAGTGGAGGGTAGGGCCTTTAGGCTCTGTTAAATTCTTGTGTCATCTGGATTGTGACCTCAAGT<br>TTCGCCCCTTGAATGGGTCTTACGTACCCTCGCATTGCACCTCCAAATCAAAATGA
CrLEA2-5	ATGATGAGTGGAAGAGAGAACGTAGTTAGAGTTTCAGAGGAGGGTGAAGCAACAAGTAGGCAGCAGCAACT

	GTACTACGCGGCAACGCCACCATCGTCTCATTCCTCCACCTGTAAAGGGTGCTGCTGCTGCCTCTTCCTCC
	TCTTCTCTTTCCTCGGTCTCTTAGTCCTAGCAGCACTGCTACTCATCGTGCTGGTCGTTAAGCCCAGGAAC
	CCTCACTTGGACCTTTCCCAAGTGGGCTTCCAGTATATGGCCATCAGGCCCAACCCCAACGATCCAGCAAC
	AGCCAGTCTCTACCTTATCATCAGGTTGGTGTTGTCGGTGGTGAATCCCAACAGGGTTAGGATCAGGTACG
	GGGAGTCCAGGTTAAGCCTCATGTTCCGCGGTGTGACGTTGGGGCGGGCCTCGGTGCCAGCTTTCTTCCAG
	CGTGCTCACAGCGTGACGGAGGTGGTGGCCACCGTCGCCGTCGATGGGGTCAACTTATCTGAGGCTGATGC
	TGCTGACTTCACCAGAGACGCTTTGTTGAATGACCGGGTCGAGCTTCGGGTCTTGGGCCATGTTGCCGCCA
	AGATTCGCCTCTTCAACCTCTATTCTCCTCCTCTTCAGGTAATTCAATTCCTCTTCTCTCTTTCTGAATTG
	TTTTCATTTTTGTCCACTTTCGCCTTCACCTTTTTATTTTTCTCAATGGATAATTATTCTACTCTAACAAT
	TTACAATTTAAAACCTTCATGACAAAAAAAATTATATAAGAATACCATTACCATTTTAAATTAGGTTTTAA
	TTATTATTAGACTTTAAAATTTGTTTGGTTCAGCTTATGTTAATTTTTTGAGATGATTTTTTAAAATAAAC
	GGTTATTTTTTCAAATTAAATTGTTACTAAACTTGAACTTAATCGATTAAGATCAAACAGGTGCGGATGTT
	AACACGAACATCGGATACAACACGAAAATGATTATTCTAACATGGTTAATGTTTGAAATATAGAATATCGA
	AACACCATATATGCCTTATAAAAATATTATTAAGTTATATTATTTTTTATTACATAAACATTTGATTTTTT
	TTTTATATAAAAATCCGAAAAATGAAAGTATATCTTTAAAAAAAAGTGTATTTATCTTCTTAAACTAACAT
	GTTAAGTTTTATACTTTTTATGATAATGAGTTTTTTTAAATAGAGATATTCTTTTTAAATTTTTCTATTAA
	ATTTTAAGTTAACTTTTCTAAACAATTTTAAAAAGTAAATATTTTTAACTATATTAATATATATTCATTTA
	TAATAATGTCAATTTTAATATTCTAACTATATATAGAGGTGTGAACTTAAGTATTTAAATTAATAAAAAAA
	AATTTATATTTGAACATGTAGTTAAAAAAGTGTCTAAAATATATTAAACAAATAATATATTAAACAAACAA
	TACTTTAAATAAAAGAGGTATATTAAAGAAAGCTATGAAACATGCAACCATGCAAGTGTAGACATTCCCTT
	CCGTGGACCAGCATATGAACTTCTCACCTTAAATACAGAGTCTCCCTTAACTTTTACTTCATTCTTCAGTG
	TCTTAATTTAAAAAAAAAATATATATTTTATATTCGGTTTTATATTTAAAAAATATTTCTTCAGTTTCTTC
	AATTTTATATTCAATTTTTTGTAATAAGTTTGATTGAATTTTTAATTTCTCATTTTGTATTCAATTTTAAT
	TTTAATCTTTACCATATTTTTTTATAATTTTGATCTTAGTTTTAACTTTTTCTATCAAAATTTTCTATAAT
	TTTAGTCTTAAAAAATTGATAGTTTAAACAGTTTGATTTCTGAATTTATTTATTTATTTATACTCTGCACC
	TTTAGAAGACTATTTAGTTTTAAAAAAATGTTGACTTATATATTTTGATATATCTTAATATGTTAATTTTA
	TTTATATATTTTTTTCATGTTTTTATGAAAAAAAAAACTAAAATATAATTAACTTAATATTAAATTATACA
	TACTAACATCTTTTAAAATAAAAAATATAGTAAATGTAATTTTAAAGATTAAATTATATATTTATTTAAAT
	AACATCATTATTTAATAAAAAATTAACTCTAAAAGTTTAAATTAATAACTTTTATAAATAAAATAAAATTT
	TAAAAAAAATGAACGAGACTAAAATCTAGAATAAGTAAAAATTAAGGGATTAAAATTTATTAAACCTCTTA
	GTACATTAATTGTGATGTTACGTGCATGATTAACATCCTCCGCCCATTTCAAAACATAACCGTCACCATTC
	ATTTCTCATTCGATGGGATGCACATTTTCTTACTTAAAAATAAATAAAAAAATAAATCAAGCTTCTGCTGT
	ACTGCATCACCTGATCAGTCACATACATACACGTTCTCAGATTCGGTTGGGTCCCTTTCCACTTTTGTGTT
	TTTGGTTTGGTTGTCTAATTTATGGGCCAGTTTGGTAGAAAGTTGAGAAGAAATGATTTAATAAAATTAAT
	TTTGATAGAACTAATTGTGGTAAAATTGATTTTTGTGTTTGATCAAATTTATCTTCCAATTCCCACAGTAA
	AATGCAAATATACAAATGATTCAATTTTACAAAATCTTAGTTTTGAAAATCTGACCTCCATTTCATTATTA
	AGTTGAATTTCATCCAAAAACAAATAAAAAATACAAAACGAAGTTTGGGACAGCAAACCAAGGTTCAGAGG
	TTTTTAACAGTATAACAAAACTCTAAATCTTAAACCCCATAAGATATAAGAATGGGGTTTTTTTATTTAAG
	GAAACATTTGTTACACATTTTCATTTAGGATATATTGGATAGGATGAAAGAGAAAGATAAGATATGCCATA
	TAATTTGATAGAAAGAAAAAGATAAAAAAAAATACAATATAGTATGATAACAAATATTATATATGTATGAA
	TATCATTACTCTTTATCTTATTCCCATTATTTACCTTTTTATAGATTTTATTTTCTCACTCATATTGAGGT
	ATATAGTTTTAAGTCATATATTTTGTAATTTAAAGTTATAATGGTTGTATATTTTATCAAAACTGCATGGG
	AGAGATCACTACTTTCATCATACTTTTAGTATTCACTTTAATCATTAAGATTTTAGAAAGTCTCAGTCTCA
	GAATCATATTCTATCAAATGCTTTCGTTTCACGCTCCTTTATGCGTTTCTCTATAATATACCGTGCTGAAA
	TCTTCCAAATTTCTATTAGCCTCACTTTTATGTACAAGATTTATTTTTTTATTTACCCTATTTTGAGTACT
	TCTCATTTTTGGTCATTTCTATTTTCTTTAATGAAATCTTTAATTTTAGCAAATGATTAATAAAAAAATAT
	TTACGTCCAGCTATATTTGAAGGTAAGTTGTTAAATTATTTTTAATAAGATCCAAACTACTTTTGATACGA
	AACTCAATTTTTTTAAATTTTTAATTTTTAATTATCACCGTGTTGTTTTTAAAAAAAATGTAATAATTAAA
	CTAATAGGCGGAGGAAAGTGAGAAAGATTCGGGCAAAAATGACTAGAGAGGGTTCACTCTAGTATTCTTTT
	TCCCATGTACGTTCAGGAATGCAATTTTTGGTATTCTGTTTTTCAGGCAAGGTGGTGCAATATGTGTGTTT
	ATGTGTTATAGATAAGGGTAGGTTTATTTTGAAAGTGAAGCTCACAAATGTGCCAAACACTGCTGCCATTA
	CAGGTGTCGGTGAATTGTGTTATAGTGATAAGTCCAAGAAAGCAATCTCTTACTTATAAGCAGTGTGGATT
	GGAGGGATTGAATGTTTGA
	ATGACCGAACCATCATCCAAACCCAACGGAAACGGCGCAATTAACGGTGCTGCCACCACTACTAACGGCAA
	CCCTGGTCCCGTTAAGTCCCAACTCTACAACCCAAACCGTCAACTTTACCGACCACAATCCCACTACCACC
	GTCGTCAACGCTCTCACCGAAACCTCTGCTGCTGCTGCTGCTTCTGGACCATTCTCACCCTCCTCGCCGTC
	GCTCTCCTCGCCGCAATTGTCGGCGCCGCACTGTACGTACTGTACCGCCCTCACCGTCCCGAATTCTCCGT
	CACAAACCTCCGCATTGCGAAGATGAACCTCACAACCTCGAGCGACTCACCCTCCCACCTCACCACGCTCT
	TCAACCTCACACTCATCTCCAAGAATCCGAACAACCACCTCGTCTTCTTCTACGATCCCTTCACCGTCAAC
	GTCTTCTCTAACTCCGTCCCCGTCGGAAACGGTTCCCTAACGGCGTTTACTTCGGACAAGAACAACCAGAC
	TAGTATTCGCACGGTGCTGTCGGGCTCCCAAGATCTGGACACGGAGTCGTTGACCAGCCTGAGATCGGGTC
	TGAAGAAGAAGGGTTTCCCCGTGGAGATTCAGATGGACACTAAGGTGAAGATGAAGATGGATTGGCTCAAG
	AGCAAGAAGGTCGGGATTAGAGTCACCTGTGATGGAATCAAAGGCACCGTTCCCGCCGGTAAGACGCCGGC
CrLEA2-6	GGTTGCTTCCGTTATCAACTCTGACTGTAAGGTCGATCTTCGAATCAAGATCTGGAAGTTCTCTTTCTAG
	ATGGAGGAGCGGGTGTCGTCGCAGCTATCGCCGCCGCCACCAGTAGTACAATCTCCTCCTCAAGACCCATC
	TATACAGAACCATAGTAGCAAAGCACTCCAATTATCCCCGAACTTGGACCCCGGCACATACGTTGTCCGGG
	TTCCAAAGGACCAAGTCTACCGTGTTCCACCGCCGGAAAATGCCGAAATCGCGGAGCTCCATCGGAGCACT
	CCCCAAAAGAATACAAAGAGGACGCGTTGCTGGTGTGTCACGTGTTTCATTGCTGTTATAATTGTCATTTT
	GATCATAATTGGAGGCATTTTGGGTGGGCTTTTCTCTGTTGTACTTAAACCAAAAGACCCAAGGTTTTCGA
	TCCAACACTTTAATGTCCTAAAGGGCACTCCCAAACCCAAATACAACATCACTTTACAAGCCCACAATCCA
	AATTCTAAAGTAGGCATTTGGTACAAAGAAGGAGGCCACGTTTCATTGTCTTTAAGGCCGAAAGAGATTGC
	TTCAGGATCCTACCCAAGTTTTCATCAAACCCCTCATGATTCAACACCCTTTGGCGTTACTTTAAAAGCCT
	CAAAAGCTGGTTTCCCTAAGGAGGTGGAGGAAAGCATCACAAATCATAAACACAAAGTGCATGTCGCATTC
	TCATTAACCATTCACGTCCCAGCACGAATGAAAATGGGATTGTTGCGTAGTGGGACAATGCAATTCGACGT
	TACGTGTCAGGTGACGTTGGATTCCTTGGCCAAAACCCCTCGTGTACTTTCTCAACAATGTCAAACTAAAC
CrLEA2-7	GACACTAG

	ATGTCTCAAATCACAATAAAATCTCCAAAACACTGTGCTACCAAGCAAGGACTGAAAATTGAGAGGAACTA
	CAAGAAACTCTTCTATGTTTTTCAGCATTTTTCACCTCAATCCTATTGTTAATACTTCTTATCTGGCTCA
	TCCTTCACCCTTCCAAGCCTCAATTCTCCCTCAAACAAGTTGACATCTACCAGCTGAACTTGTCAGGACCA
	AACCTCAACTCCTCCATCCAACTCACTCTTCTATCCAAGAATCCAAACCAGAAAGTTGGTATTTACTATGA
	TGAATTTCAAGTTTATGCAACCTATAAGGGTCAGCAGATAACTGGTGACACCCCTGTGCCTCCCTTTTACC
	AAGGACAAGAAGAGAATAATCTCTTAACAGCCTCTTTGGTAGGAAATGGGTTACCAGTGGCTCCTTCTCTT
	GGTTATGAAGTGGGTCGTGATCAAACTGTTGGAAGACTAGTTTTGAATGTCAAAGTTAATGGAAAGCTTCG
	TTGGAAGGTGGCAACTTGGGTCTCTGGACGTTACAGGTTCAATGTTAATTGTGTTTCAATCATTGCCTTTG
CrLEA2-8	GACCTTCAGCTCCTCTCACTTCAAAGCAGGGAGCTCGGTGTTCTACCACAATTTAA
	ATGAAAGCAGGATCTGGTAAAGGGAGAAAAGTTTGCCTGACAGTGACAGGTGTTGTGATTGCAATTGTAAT
	GCTAATGGTGATACTAGCATTGACGGTGTTCAAAGCCAAGCATCCTGTTACCACAGTGGACTCAGTGATGC
	TGCAGGACTTCCACATGAGCTTGGATATAGCAAAACTAAAGGTAGATTTGAATGTGACCCTGGACGTGGAT
	GTCTCTGTGAAGAACCCAAATAAGGTGGGATTCAAGTACTCAGACAGCACCGCCCACCTCAATTACAGAGG
	GCAGCTGATAGGTGAAGTCCCAATCTCAGCTGGAGAGATTTCTTCTGGTGAGACCAAAGGATTCAACCTCA
	CCCTCACCATTATGGCCGACCGTTTGATCTCCAATGCCCAGCTTTACTCTGATTTCACATCTGGTACATTG
	CCCCTAAACACTTTCATTAGGATTTCTGGCAAAGTCAGCATCTTAGGCTTTATCAAAGTCCATGTGGTTTC
	CTCCACATCTTGTGATTTTGCGGTTAATCTTTCCAATAGAACAGTTGGAAACCAAGAGTGCCAATACAAGA
CrLEA2-9	CGAAACTTTGA
	ATGATCTTATCGACACGTTTGAGACCGTTGATTAGTGGCAACAGAGAGGTCACGTGCGACACGTATCATCA
	CGTGTCGGCATGGGACGAGACGTGTGGTATACGTGTCTGGAGCAGGAACCTCGTATGGACATAGTAGGACA
	GTGTTTGGTTTCCCTCTTGCTATAAATGTTTGTGACTTTCGCAATGGCAGCGATAAGTGTCATCGATAATT
	TGAGATTGAAAAATGTCACAGTTGCTGAATAAAGCCAAGGACTTCGTGTCCGAGAAGATCAACAACGTGGC
	CAAGCCTGAGGCGAGTGTCACCGACGTCGACTTCAAGCGCGTGAGCAGAGATAGCATCGAATACTTGGCCA
	AGGTCTCTGTTCGCAATCCTTATTCAACTCCAATACCAATCTGTGAGATCAAGTACTCTTTCAAAAGCGCG
	TCAAGGTACTCTCCACTCAATTTCTTCATTTAATTCTGATTATTAAAAATTGAAAAATGTTTCTTGAATAA
	CAAATTATTTTCCTTTTTTTATCTTGTTATCTATTTTTTAGAGGTCTGTGTACGAATAAGATAAAAAATAAT
	TTTATGTAATATCATATTATATGGTGTCAGTTTTTTGTTCCTCTAATGAATATTATAAAAAAAGCTACGAA
	AAAAATAGGCCAAAATTAAGAGATACTGAATTTTTATATTAATCAAGCAACGTATAAGATACATAAAATAA
	AATAATAATATATACTTGCTTAATCACATCTATATATTTAAAAATTTAATTTTAATCTACTGTTGCTATAA
	AAATTTTAATCACAATAAAATAATAATACTTGTTTAAGAAATGTTTGTGTAATATTTCTTTGGTTTAAATA
	ACTCAATACATTTATCAAATTTTGATGTTATTGTGTTTGGTAGGGAGATAGCATCAGGCAGAATGCCAGAC
	CCAGGATCGCTGAAGGCAAAGGATACGACAATGGTGGAGGTACCAGTGAAGGTGCCATATAGCATATTAAT
	GAGTTTAGCTAAGGATATTGGAGCTGATTGGGATATTGACTACCAACTGGATCTGGGTCTCGTTATTGACC
	TTCCTGCCATTGGCAATTTCACCATTCCTCTCTCTCACAACGGAGAGGTCAAGCTACCAACCCTCTCTACT
CrLEA2-10	ATGTTCGCTTAG
	ATGGCAGAGAGAGAGAGCAGGTTAGACCCTTAGCTCCATCCATAGATCGTCCAAGCAGCGACGAGGACGACAC
	CACTCTACACCGCACATCACCTGCTCGTACAAAATTCATCAAATGGTGGGCTTGTCCATTAGCTTTCTTAC
	TTCTTCTAGCAATTATAATCATAGTTTTAATATTCACCGTGTTTCATGTCAAGGACCCTGATATCAGAATG
	AACGGTGTCAAAATCACAAAGCTAGAACTTGTCAACACCTTAACCCCTAAACCTGGGGCTAACATGTCCTT
	AATTGCAGATGTATCGGTGAAAAACCCTAACGTTGCATCCTTTAGGTATAGTAACACCACCACAAGTTTGT
	TCTATCATGGCATCATGGTGGGAGAGGCTAGAGGACCCCCAGGGAGGGCCAAGGCTAAACGAACAATTAGG
	ATGAATGTTTCAATTGATGTCATCACGGATCGTATTGTGTCTAATCCTGATTTAGGACAGATTTCGTTTC
	AGGGTTGTTGACTATGACCAGCTTCTCCAGAGTTCCAGGGCAGGTTAAGATCTTGAACTTGTTCAAGAAAC
	ATATTGTGGTCAAAATGAATTGTACCACCACTTTCAATATTTCTACACAGGGAATTCAAGAACAGAGTTGT
	AAGCGCAAGGTTAAAATTTAATCATCCATGTATAATTAGTATTAGTATTAGTATTAGTTTAGTTTCTTTTT
	TACTGTATTTGTATAATTCTTGTATAAAAAATAATGAAAAATTTCCTTTGTTTTTATGTTTTCATGTTATG
	GGAAAAGGAGTGATTTATGGGAGAAATTTTTGGGCTTGTGAGTGTTTTCTTTTTTTTAAGCAAAAGACAAC
	TTGCTCTACAATCTCTCCATTTGGGGCTACCAATTCTTTGAAAGTTATTATTTCATATGGCACTAAGCAAG
CrLEA2-11	ATTTGCGTGTAATCTGA
	ATGCAAATACACGATCAAGAAGAACCAACCCAACAACACTCACAAAACCAACCTCATCATTTCCGTGTCAA
	AGTGCCTCAACAACAACACTACAACATGGACAAAGGTATCCCTCCTCGCTTCAAACCCAATGCCCCAAAAC
	GTGAACACTGCATTTGCATCACAGTTTTTCTCCTTCTACTTGGCATCATTATCCTCATCCTCTGGCTCGCT
	TACCACCCTTCCAAGCCACGCTTCACGGTGGCCAGCGCCGCCATCTATGGCGTCAACACCACATCACCGCC
	ACTCATGTCCATCACAATGCAATTCAATATCCTCATTAGGAACCCAAACAAGCGTGTCTCAATCTACTTTG
	ACAGACTCTCTGCTTACGTGTCCTACAGAAACCAACCAATCACGCCACGTGTCATGCTGCCACCACTCTTC
	CTCGAGAAACACAGCGCCGTCTCGCTGTCGCCGGAGATCGGAGGCGTGCCGGTGCCGGTGTCGGAGGAGGT
	CTCAAATGGATTGGCAATGGATGAGAGTTATGGGGTGGTGGCTGTGAAGCTTATGTTTCTTGGAAGGTTAA
	GGTGGAAAACTGGTGACATAAACTCTGCACATTATGGATTGTATGTCAAATGTGACGTGTTAATGGGTTTG
CrLEA2-12	AGGAAAGGTTTTGTGGGTCAGGTTCCTCTTCTCGGAGCTCCGGTTTGTGAAGTGAATACTTGA
	ATGACTGATAGAGTCCATCCCTCCGCCAACGGCGGAAAAACCGCCACATTTCCCGCCACAAAATCTCAACT
	TTACGGCGCCACCCGTCCCTCCTACTGCCCCCAACCGTATCACCAACGCCGTAGCAGCAGTCGGGGATGGT
	GTTGCAGCCTCTGCCTCTGCCTCATCCTCATCCTCCTCTTCCTCCTCCTCCTCATCGGCGGCGCCGGCACG
	GTCGCGTACTTCCTCTACCACCCTCAACGACCTTCCTTCTCCGTCACCTCTCTGAAACTCTCGTACCTGAA
	ACTCACCTCTTCCTCGACTCTTAATTCCAAGTTTGAGCTCAAACTCTCAGCCACAAACCCTAACAACAAAA
	TTGTCTTCTCCTACGACCCCACATCCGTCTCCATCCTCTCCAACGAAGACATCGACATCGCACACGGCACC
	ATTCCCTCCTTCCGCCACTCCCAGAGGAACATCACGATCCTCAAAGTTTCCATTGCGAGCAGCGAGGAAAG
	CGTGGAGAGCGACGCCGCGATGCGGCTGAAGGGGATCATGAAGAGCAATAGCGGGTTGGCGCTGAAGGTGA
	AATTGGAGACGAAGGTGCAGGCCAATATGGGTGTGCTTCACACCCCAAGTGTCCCCGTTACTGCTTTCTGT
	GATGGCGTCGCCGTCACACTTCCCGACGGAGATAAACCGGCGACGGCGTCCATCGCGAACACGGAGTGCAA
CrLEA2-13	CGTGGATGTGAGGTTCAAGATATGGAAATGGACCGTTGGATGA
	ATGTATGAGAAACAACCCCAATTGAACGGAGCCTACTATGGCCCTTCAATTCCGCCACCAAAATCTTACCA
	TCGCCCAGGGCGCGGCGGCGGCTGCGACTGCTGCTGTGGCTGTATCTTCAGCCTGATCTTCAAACTGATCC
	TGACGGTGATCATCATCGTTGGAATCGCCGTCTTCCTGTTCTGGCTCATCGTTCGACCCAACGTGGTGAAG
	GTTCACGTGACGGAGGCGACCCTGACGGAATTCAACTACACGGGGAACACCCTATACTATGACTTGGCTCT
	GAACATGACAATTCGCAACCCCAACAAGAGGCTGGGTATCTACTATGATCGCATCGAAGCCCGTGCAATTT
CrLEA2-14	TCCAGGATGCAAGGTTGGACTCCAAATTCCTGGAGCCCTTCTATATGGGCCACAAGACCACCAAGCTTTTG

	AATCCTGCGTTCAAGGGTCACCAGGTGGTTCCACTCAACACTGACCAAACTGCGGAATTGAAGAAGGAGAA
	CAGCACCGGGATTTACGAGATTGATGTGAAGCTGTACCTCAGGGTGAGATTCAAGTTGGGAGTGTTCAAGA
	CCAAGACGCTCAAGCCTAAGGTTACCTGTGACTTACGCGTGCCTTTCACTTCACCTAATGGACCCTCGCCA
	GCGCTCGGTGCTTTCCAGACTACCAAGTGCGATTGGGATCGTTGA
	ATGGCCGATAAGCAACCTCAGCTGAACGGTGCGTTTTACGGTCCTGCCATTCCCCCACCAGCGCAGCAACC
	ACGCTACCATCGCCACCACCGGGGCCGAAGCTGCTGTTGCTGCCTCTTCGGAATCTTCTGGAAGATTCTGG
	TTGCGCTCATTGTCCTGGCAGGCCTGGCAGGCCTCATCTTCTACCTGGTGGTTCAACCCCGCTCCTTCAAG
	TTCTACGTCACGGAAGCCAACCTTAACCAATTCGAATACCACAGCAACAACAACACCCTTAACTACGACAT
	GGTACTCAACTTCACTGCCCGCAACCCCAACAAAAAGCTGAGCATCTACTACGACAAAGTGGAGGCTTTAA
	CATTCTACGAAGGAGCCAGGTTCAGCAATTACGATGTCATCACGCACTTCAACTCATTCCGGCAGTATAAG
	AAGAGCAGCAGCCCCATGAGCGGCGTTTTCAAAGGGAACAGAGTGGTGGTGCTGGACAATGACCAGCTTTC
	TGAGTTAAACAGAGACCAGAATGAAAGGGTTTATGATATCTATGTGAGGCTGTACTTCAGGATTAGGTTCA
	GACTCGGTGACGTCATATCCAGGGACTTCAAGCCCAAGGTCAAATGTCACCTCAAAGTTCCCTTAGCTTCT
CrLEA2-15	ACCACCACAACTCTGTTTCAGCCCACCAAGTGCGACGTCGATTTCTAG
	ATGTCGGTGAAGGAGTGTGACCACCACAAGGGGAAGAAGCACAAAATCTTCCGGCGCATCTTCTGGGGCAT
	AGTGATCTTCGTGTTTATCGTGCTGGTAACAATTCTGATAATATGGGCAATCCTAAAGCCTACGAAGCCAA
	CCTTCATCCTCCAAGACGTCACCGTTTACGCCTTCAACGCAAGCATCCCCAACCTCCTCACCTCCAATTTC
	CAGGTGACACTCTCCTCTCGTAATCCCAACGACAAAATCGGCATCTACTATGACCGTCTCAATACATATGT
	CACTTACCGGAACCAGCAGGTCACCTACCGCACTTCCATTCCTCCCTCTTACCAAGGTCACAAGGAAGAGG
	ACGTCTGGTCACCGTTTGTTTACGGTACTAATGTCCCCGTTGCTCCCTATAATTTCGCTGGCCTCAGTCAA
	GACCAGTCTAACGGTAACGTCCTCGTTATCGTTAAAATTGACGGCAAGGTCCGTTGGAAGGTCGGTGCCTT
	TATCTCCGGCCATTACAACCTCTATGTACGCTGCCCTGCTTTCATCACATTTGGCCCTCGGAGTAACGGAA
CrLEA2-16	TTGCCATTGGCGATAACGCTGTTAAGTATCAATTGGTTCAGCGTTGCACCGTTGGCGTTTGA
	ATGGAAAAGGACAAAATTTCCGGTTCACCCAAACGTGCAGTGTGCACATGCATCACAATCTTCCTTCTGTT
	AGTCGCCATCACTCTCCTCGTCCTCTGGCTCGTCTACCGTCCCCACAAGCCTCGCTTCGTGGTGGTCGGCG
	CCGCCATCTACGGCCTCAACACAACCACCCCTCCCCTCATGTCTGCCACCATGCAGCTCACCGTCCTCATT
	AGGAACCCAAACAGGCGCGTCTCCGTTTACTATGACAGATTCTCCGCCTTCGTTTCCTACAGGAACCAAGC
	CATAACGCCTCAGGTTGCGCTGCCACCGCTGCACCAGGACAAGCACAGCACGGTGTCACTATCGCCGGTGA
	TCGGAGGCACGGCGGTTCCGGTGTCGGTGGAGGTGTCAAATGGGTTGATGGTGGACGAGGCTTATGGGGTG
	GTGGGTCTGAAGCTGACTTTTCAGGGAAGGGTGAGGTGGAAGGCTGGAGCCATCAAAACTGCACACTACGG
	ACTGTATGTCAACTGCAACTTATTGATGGGTTTGAAGAAAGGTTTTGTTGGTCCAGTTCCTCTCCTCGGAG
CrLEA2-17	CTCAACCTTGCGATGTCGATTTATGA
	ATGGATGTTTCCGCTTCTAATTCCGATTGCTTCTGGTGGAAAGAACTTCATCCCGGATTTTCTTTTAAAAA
	ATGCTGCTTCTTTCTGCTTATCTTCCTTTTGATCACATGCATGGTGGTTGGATTAGCCACTTTTTTTGTGA
	TCCTCATCGTTAGACCCCACAAACCGGTTTTCTCTGTTAGAGAGGTTCAAATAAATTTTTACAAGATTGAT
	GATCGTTCTAATCTGACACTGTTGGTTTCATCTGTAATCTCCTTAACCCTCAATGCAGAAAATCATAATAA
	GTTTGGTATAGGTTTTAGTCCATCAAGGTTCCTTGTTTATCAAGAAGGCTTGCATATAGGAACTATTCGAG
	TTCCATGGTTTTTTCAACCTCCTCACACTGAAAATGTGACCGTACCATCTCGTGTCTTATTGCAATGTGTT
	AATCTCAGCAAAATTGTCGCCAACACATCCTTACAAGAAATGTCAAAGCAAAACACGGCGCCGATCAAGAT
	CACAGGGGATGCCAGAGTCCACGTGTGGGTACTTCATATAAAACTTTTTGAGATTAAGGTTAGCTCTGCAA
	TTCTTCTTTTTTCCTTTCTCTCCAATTTTTAGACTCAACTATATTTTGAGCTACAATACTTAATTTCTACT
	AAAAAACACTACTATATCTTTTTCATTGAAATTTAAACCTATACATCTAACTCCTAAAGGAATACTTATTG
	CTTAATCAACTAAGTGTGATCCATTGGTTTGGTCTCGCTGTTAGTTAGCACAATAATTGAAGCTAGAAAAT
	TCGAAATGAAATGAAAAAAAATTACACAAAATAAGGTACCAAAGAAGTTCCTTATTTGACTTTTTTTTTGT
	TTGTATTGATTTATTGTCACGCATTAAATTTAGTATTATCATTATTTAAATATCCACCGGCATCAATCATA
	GACCTATATATACATAAAGGTGAGGGAATATTATTTGCTGATTGTATTTTTGCAGATTGCACTAGATTGTG
	GTATGAATTTTAATATAAGAGAGTTTGCTTTCACAAATGAAGCGTTTGGTGCCAAAATTAGCAAGAGTGAC
	TTGGTATTGACTATTAATTTGTTGAACTTGTTCGTTTATGTCTTTATTTGGTAGGCTTAGTAAGTATGATT
	GAAAGCCTTATATACCTATAAAGTTTTGTTTCAGGGTTCAGTTCTCAGTGATTCAAAAGGCATCTCTATGA
CrLEA2-18	AATGTGCTTCATCTGTCTACATATAA
	ATGTCTGCATTAGCACCAAAACAAGAAATCATGACAGAAGCACAAAATAATGAGGATCAAGAGCAAGTGGT
	GGTGATTTCACAAAAAAAGCTCAAGAGAAGAAAAGTGTGTGTAATTACCACAGGAGTAGTGTTGTTACTTC
	TTGTGGTGCTAATCATAGTGGGGCTTGTCCTTGTTTTGAACTTGCTCAAGCCTAAAGATCCAAGGGCAGAC
	CTTGTTTCTGCCACTTTGGAGGGTATAGCACCTCGTGTCACACTTCCTGCTATTGACATACAAATCAATGT
	TACTCTTGATCTCAAGATTCAAGTTGAGAACAGAAACCATGTTAGTTTCAAGCATCAAGGAGGGAAGAGTG
	TTTTGTTATATGGAGGAAAAGAGGTTGGAGAGACTCAGTTATACCCTGGTGTGATTCCTTCAAAGGGTTCT
	ACTATACTTCCTTGTAGACTCACCCTTCAGGCAGAGAAACTTGCTTCCAATTTTTCTGGTTTGGTTGAGGA
	CTTGATGGGAGGACAAATCTCCATGGGAGCTGTTACTAGGATTCCTGGGAGAGTTACCTTCCTTGGATTCA
	TAAAAAAACATATTATTGCTAAGTCCAATTGCCAATTCACTGTTAGTGTTTCTGACTTCAAGATCATTAGC
CrLEA2-19	CAAGCTTGCAAGAATAAGGCCAAACTATGA
	ATGATAGAAAAATATGGGCACCGCAAAAAGAGTAACATGTGCCTGGTGGTAACATGTATTGCAGTGATAGC
	AATTCTGGCGGTAGCAACGATCTTAGTATCAACGATGTTAAAACCAAGGCAACCTATAACCAAAGTGGAAG
	GCATAAGGCTTGATGACATGAATATGAACATGAATATGTTTAAAATGAGGATGGATATTAACGTGACATTG
	AAAGTGGATATGTCGGTGGAGAACACAAATAAGTTTGGATTTGTGTACAATGATAGCCTTGCTCAACTCAA
	CTATGGAGGGGAATTAATTGGGGAAGCCCCAATACCCAATGGAGAGATATTATCTGAGGAAACTAAAGGAA
	TGAACTTGACACTAACCATTATGGCTGATCGCATGTTCTCCAATTCTCAAGCTTTCAAAGATGTTACAACG
	GGTTCATTGCCCCTCAACACTATTGTTAAACTTTCTGGCCAAGTCATCATCTTGGGCCTCATCAATTTTCA
	TGTGGGTTCCACCTTATCTTGTGATTTCAACCTCAATGTTTCCAATAGAACCATTGACCACAAAGAGTGTC
CrLEA2-20	ACCATGACACAAAGATTTGA
	ATGAGAGTGGGTTTGGGTGTGAAGGTGTCGTTGGGTGTGATCGGTGTGAGTCTGATATTGGCAGCGTTTAA
	AATGTCGGAGTTGTTGGACAAAGCCAAAAACTATGTTTCAGAGAAAGTGGCCAACATGCCCAAACCTGAGG
	CCGCTGTCGCTGACGTGGATTTCAAGCGCGTGAGTCGAGAGGGCGTCGAGTACTTGGCCAAGGTCTCTGTT
	TCCAATCCTTATCCCTCTCCCATTCCAATCTGTGAGATCAAGTACTCCCTCAAAAGTGCTGGCAGGTTCGC
	ATTTCTATTTCTTTTTAACTTTTTTCGTTCCTAAGCGAGGAAAACTGGAAAGGTCAGAAAGTTTATTAATT
	AAAGATATAAAGTAATATATCTTAGAAATTTAAACTAATTCAAATCAATAATATATCTTTCCTTAGACCAA
CrLEA2-21	ATCGGAATTTTTATTTATTTGATGAATTGTTGGGATAATTGGATTTGTTTTCGATTGGTTTTGTGTTGGGT

	GATGAAAGGGAGATAGCATCAGGGACAATACCAGATCCAGGGTCGTTGAAGGCAAAAGACACAACAATGCT<br>AGATGTGCCAGTGAAGGTGCCTCATAGCATATTGTTAAGCTTGGCAAGGGACATTGGTAGAGATTGGGACA<br>TAGACTATCAATTGGATCTTGTCTTAATTATTGACCTTCCTGTGGTTGGCAACTTCAGTATTCCTCTTTCT<br>CAGCAGGGAGAGATCAAACTCCCCACCATCTCTGATATGTTTACCTAA
CrLEA2-22	ATGTGCAAAACCGTAGGCAGATTATTGGCCTTACTCTTGGTCATTATCCCCGTAACGGTGACCGTTACGGC<br>CTTTCTTTTTTGTCTCATTGTGCGACCCAACGTCGTTAAATTTCACGTGACCGATGCTACCCTCGCCCAAT<br>TCAGTTACACTAATAACACCCTTTACTACGATCTCACCCTCATCGTGGAAGTTCGCAACCCCAACACAAAG<br>ATCAGAATCCACTATAACTACATGGAAGAAGTTGTGTTATACCAAAATGTGAAGTTCTGTTCGCTGATCTT<br>AGGAACCTACATTCAGCCACACAAGAACACTGCCATGTTGACTCCTGTTTTTAAAGGTCAACAAGTCATGT<br>CCCTCAACAAAGACCACATTTTGGAGACTTACAATAATGAGAAGCGTTCTGGGATTTACCACATCGATTTT<br>AAACTTTACTTGAAGGTTAAGTTCGATTTGGGTGTGTACAAAACTAACAATATGAAGTCCACGGTGACTTG<br>TAACCTACAACTCCCTCTTCAATCTTACAATGGAACCTCAACCGCTGGTAGGTTTCAACTTACCCGCTGCA<br>ACTTCGATTACAAACATATTTTGTTTATTTTCTGA
CrLEA2-23	ATGGCTGATTATAAGCAACCCCAACTAAACGGTGCGTATTATGGCCCTTCCATTCCTCCAGCGGAGCCACC<br>ACGCCAACGCGCTCACCGCGGGAGAAGATGCTGTTGCTGCCTCTTCAGCTTCTTCTGGAAGTTTCTCCTCG<br>CAATCGCTGTCCTCATTGGCCTCGCCATCCTCATCTTCTGGCTCTTAGTTCAACCACGCGCCTTCAAGTTG<br>CACGTGACGGAAGCGAGTCTCACTCAATTCAACTACACCTCCAACACTCTCCAGTACAATCTCGTCCTCAA<br>CTTCACGGCCCGAAACCCTAACAAAAAACTCAACATCTACTATGACAAAGTGGAGGGTCACGTGTTCTACG<br>ACAGCGTCAGGTTCGCTTCAACGGACGTTATCACAGGGAAAAACTCGTTCCGTCAATACACGAAGAGCACG<br>AACCGCATGAGCGGCGTTTTCTCGGGGAAACATGTGGTTGTTCTTGATGCCTCTGATTTGAAGGATGATAA<br>GAGGAAAGGGGTTTTTGACATTGATGTGAAGCTCTATTTTACCATTAGGTTCAGGCTCGGTGACTTCATAG<br>GTGGTGACACCATGGCCAAGGCTAAATGTGAGCTTGAGGTTCCTTTCAGGTCTAAGGGGACTGAGGTTACT<br>GCGTTTCAACCCACCGAGTGTGATGTCGATTTTTGAGGGTCCCTATTTCACGCTCTTCCCCTCTAATTTAA<br>TTTTCTCATTCCTTCGATTCCTTGGTTGCATTTTCTACTTGCTTCAGGTTTGTTGTGTAATTGCAATTTT<br>GTAAAAAAAGAAAAGAAAAGTATATGGTCCTGTCATTTTTCTTTGCCCGTAGCTTTTTTCTTTTTATGTGG<br>AATCTTGTTTATTGAATACAATATTATAACAGTAGATATTTCATCTTAAATATTTATTTTATTTTTTATTT<br>TTATATTTATAAATATTTAATTTAGAGAATGTATATTGAATAATGGGTATTTTTTATTTGGTTTAAATACA<br>TTTTTAGTTTTTATAAAGTGGACTACTTTTTAGTTAATTTTTTTAAATTAATTAAATTCTTATTTTTAAAG<br>TTTAGTAAATTTAGTTTTTGTAGATTTTATTTTACTTTAATTTTTAAATTCTTTTATTTTTATCTAAATTT<br>TTCAAATTTATAATGAAATTGAAATAAAAAATAAAAATTTAGTGATAATCTAATAAAATTTAAATAAATTT<br>GTAAAATATGATATTAGAGTTTGAAGTTGGTAATATGATATTTTGGATACAAGTTCATATTTTAATTGGTA<br>AATTTGAAAAAAAAAGACAGTTAAATTTATTTAATAAATAAAAATTTTAAATAAAATAATAGAATTTAAGA<br>ATTTTAAATAGAAAAATGGTATAAAGAATTTAAAATTTAAATATGTTTGTAAAATAATTTAAAGAAATTAA<br>ACATATAATTAAATTTTTTATTTTAAAAAGATAACGCTGAAAGGATGGTATAAAGAAAATAGTGTGTGGAT<br>ATCATTATTTTGACTTTTATATATGTACAAAATTGTATGCATGTGCATTAAAAAATATTATAAATTGATAA<br>ATAAAGTGATTGGTGAGTCAAAATGAAATTGAAACAACTGCCACCACATGGTTTGGATATTTTCCCAACAA<br>CTCAAGAAAATTTTAAATTCCAAACTTAAACTCAATAAAAAATTAAAAAAAAAAACCTTTTACTTTAAGTC<br>AACCAAGATTTTCAGATAAATGTTTTTCAAAGCAAAAGATAAAATTTTATATCATACTTAAATATCACAAT<br>ATAACAATTAAAGAATTGATGTAGTAAAAATAAATAAATCTTTTTTTTTAGGTAAAATTACATTTCATTGA<br>AACACCAAAATAGATCAATTAAAAAAAGAACGTGGACCATTTATCCAGCACATGAAGATGTTAATGCTTAT<br>CCTTTTAGTTTTTTATTTTTAAAAATTATTTTGTAAATAAAGTTTAAAAACTGTTTTTTTTAAGAAAGTGA<br>AAGAGAGTAAGTAAAAAATAAAATAGAAATATAAAACTAACAAAAATAAATAAAAATCTGAAACAGTTGGA<br>TTTTTAATTTTTAAAAAATTAAATTTATCAAATATTTTTATTTATAAAATAAAAAATATTAAAAAAAAAAG<br>AAATAAACAAGTTCTAAGTGATTCAGTCTCCTTTCCCAATCAATGGTATGGGCATTTGAATTTTCTGCCCA<br>TAGTGTCCAATAATACTCATACAATTGAAAATGTCTACTAAATTTTCCAAATGGCTCTATATTGAGATGAA<br>TATAAATATCTTTTAAGTATTTTCAAGTATGAAGTTTTTTCTTATCTTGAACTAGAAATCGTTTCCTTGAT<br>ACATACAAAGACATTATTTAGGGATAAATATTTTTTACATGAAACAATCATAGGTACTCGAGTTAAAAATT<br>ATTTTTTCGTTCCTTTTTATTTTTTATTTAAAATTATTTTATACTATATTAAAAAATTAATTATTTTTATT<br>TAAGTTAAAATTATTACATAATTTTTATTATATTCTTAATGTTTCATCATTTAGTTTTTATGTTCAATTAA<br>TATTCTCATACAAGATAAAAGGTATAATGAAATAAAAAGAATAAATAATAAATAAAAATATATTTTTTAAA<br>TATTGAAAGCTGAAGTTAAAAAAATAGAAGTAATTATAAGACTCATATAATAGGATAAAGACCCTTTTCAA<br>TTATTTTTCTCCATTGAAGAATGTACATAGTTGTTGAATAAAAATTAAACAATTTAAATTTTAAGTTCATA<br>TTTATAATAATTAAAAAAATTAAATCTAAATTATCCAATCTATATTTAACAACGTGATTTTAAAACTGCAC<br>GTTCTATGATTTTCTTAGCTAAAAAATATGATGGCCCATTCTTGTTAAGTGAGTAAAAAATTCTTAGGTA<br>TCAATTTCCCACAAATACTTCTATTGATAATGGATATGTATAAAAATAAGTGGGACGTAGACATGCTAGTG<br>GAATTTTCTTTAGTTCTACGGTCTTGGACAAACTAATTGAAAATGTCCAATAATACTCATACAATTGAAAA<br>TGTCAACTAAATCCTAAATGTCCTCATCACTCTAAATTGAGATATATACAAATATTTATAAGTATTTATTA<br>AAAAGTTTGATTTTTTTCTTATCTTGAACTAGAAATCATTTTCTTCATATATGTAAATACATTATTTAGGG<br>ATAATCTTTTTTTAACATGAGACAATCATATATATTTGTATGAAAAAATCACTTATTTTATTTCTTTTTAT<br>TTAATATTTAAAAATATTTTATACATATAAAAAAAGTAATATTTTTAATTCAAATTATTATATATAATTATTA<br>ATTATATTTTCAATCTCTCATCGTTTAATTCCTCTCTTCAATCTCTCAATAATTTCTCCACCCTTAAGACT<br>GCTCCATTAAAGCCGTCACCGTGGTTTGTTTTGACTGGAAACCACAGAATTATGTCAAAATGTCAAACATA<br>ACTCGACATGAAGCAGCAATCAAGCATAAAAAAGTCAAAGCCGCGTTAGTTTCTGTTATATCAAGAAATAA<br>AGATTGTTCAAAACGCATCTTTATCCTTCATATATATACACGTGACCCTCTCTTAGCCTCAAAACAGAAAG<br>CATTTAACTACACAACCGTCGCAATTGACTCACAACATGACCGACAAACAAACGCAATTGATGAACATTGG<br>AAACAACGACCCCTCAGTTCCGCCAACGGAGCAATCACGGCACCTTGGCAGAAGATGCTGTTACTGCCTCT<br>TCCGAACCTTCTGGGTCATTCTCGTCTCCATCATCATCCTCATTTGCCTCATAATCCTCGTGTCGTACATC<br>ATCATCCAACCCCGTTCCTTCAAGTTCCACGTGACGGAAGCCAAGCTCACCCGTTTCAATTACACCGCTAA<br>CACCCTCCGTTACAATCTTGTCCTCAACTTCACTGCCCGAAACCCAAACAAAAAGCTCAACATCTACTACG<br>ACGAAGTGGAGGGTCACGTGCTCTATCACGGCGTCAGGTTTGCTTCAACGGACGTTATCACGTGGCAAAAC<br>TCGTTCCGTCAATACACGAAGAGCACGAACCGCATGAGCGGCGTGTTCTCGGGGCAACGCGTGATTGTGCT<br>TGATCATAATCGGGCCTCAGATTTGGAAGAAGATAAGAGAAGTGGGATTTTTCACATTGATGTGAGGCTCT<br>ACTTCACCATTAGGTTCAGGCTTGGTGACTTCATATGGAATAGTGATATCAAGCCCAAAGCCAAGTGTGGG<br>CTTAAGATTCCTTTCAGTTCGAATGGGACTACGGTGAATGAGTTTCGGCCCACCAAGTGCGATGTTGATTT<br>CTGA
CrLEA2-24	ATGTCGGTGAAGGAGTGCGATCTTCACAAGGGGAAGAAGAGGAAGATCTTCCTGCGAATATTCTGGGGAAT

	AATTGTCTTCCTTTTCATCGTGCTCGTTACAATCCTTATAATTTGGGCAATTCTAAGACCCTCAAAACCTT
	CCTTCATTCTCCAAGACGTCACCGTTTATGCCTTCAACGCCACCGTTCCCAATCTGTTAACCTCAAATTTC
	CAAGTCACACTGTCTTCACGGAACCCAAACGATCACATAGGCGTCTATTACGACCGTTTGGATACTTATGT
	CACTTACCAGAGCCAGCAAATTACTTACCGCACCGCAATTCCTCCCTCCTATCAAGGCCACAAAGAGATCG
	ACGTTTGGTCGCCGTTTGTTTACGGCACCAACGTCCCCGTCGCGCCTTTCAATTACGCCGGTCTTAGTCAG
	GATCAGTCCAACGGTAACATCCTCGTCACCATTAAAATTGACGGCAGAGTTCGTTGGAAGGTTGGCGCCTT
	CATCTCTGGCCGTTACCACCTCTACGTCCGGTGCCCTGCTTTCATCAATTTTGGGACCCGGAATAACGGAA
	TTTCCGTTGGTGAAAACGGCGCCATTAAGTACCAGCTTGTTCAGTCATGCTCCGTTAGCGTTTGA
	ATGGCAGATCATCAGAGACAAAGGATCCACCCTATGGAGGTAGGAGCACCACCACCACCAACCATCCCATT
	GGTACCTCCAGGTTCCTCAAGATCAGAAAAGGGTAACCCTTTACACCGTCCACCACAACTACATGCTAGGC
	CAGCAATATACCCTTCACCAAAAAAAGGAAACTGCTTTTGCAAGTGCATATGTTGGACTCTATGCTTGGTT
	TTCCTCCTGCTCATTATCTTGGCAGCAACTGTTGGAATCCTTTACCTAGTTTTCAAACCAAAACTTCCCAA
	TTACTCAGTTGAAACACTGAGGATAAGTGATCTTAGGCTCAACTTTGATTTGAGTCTGTATGCAAAGTTTG
	ATGTGAAGATCACAGCAAATAACCCAAATAAAAAGATTGGTATTTACTATGAGGAAGGTGGGAAATTGAGT
	GTGTGGTACAGAAAGACAAGACTTTGTGAAGGGTCATTACCAAAGTTCTACCAGGGTCATCAGAACAAAAC
	AGTGCTGAATGTGTCCTTGACTGGTCAAGTGCAATCTGGAAGCACCTTAATGACGGCACTGCAGCAACAAC
	AGCAAACAGGACGCATTCCATTGGATCTCAAAGTGCATGCACCAGTTGCCATCAAACTTGGGAGGTTCAAG
	CTGAGGAAGGTCAGAGTCTTGGGTGAATGCTTGTTGGTTGTGGATAGCTTATCATCTAATAATCTCATAAC
CrLEA2-25	CATCAAGGCTAGCAACTGCAAATTCTCAATGAAACTTTAA
	ATGCGCAGCAATGACCACATACCCGTTCATCATGTCGCGGACCGAACCCGAAACCCATGAAACGACACCG
	TACCGCTCGGTACTACGTTCATCGGGTCCATGAAAGCCTCACAACCCGCGTGTCCAAGATAATCTGCGCCA
	CTTTCTTGGGCCTACTTGCCATTGTGGGCCTCATCACCTTCATCCTCTGGCTCAGCCTCCGGCCCCACCGG
	CCCAGGTTTCATATCCATGAATTCAACATGCCGGGCCTGACCCAACAATCCGGGTTCGAAAATGCCCGGAT
	AACCTTCAATGTAACTGCCCGAAACTCCAACCAGAACATCGGGGTTTACTACGAGTCCATGGATGGAGCGG
	TTTATTACCGGGACCAGAAAATCGGGTCGACATCGTTACTTAATCCGTTTTATCAAGAGCCCAAGAACACG
	ACGGAGGTGGACGGTGATCTCAGTGGTGCCACGTTGACCGTTAGTAGTCAACATTGGGCAGAATTCCAAAG
	CGATAGGGCTGATGGTAGCGTGGTGTTCCGCTTGGAAGTGACGTCTGTGATCAGATTCAAGATATCAACGT
	GGGACAGCAAGCGCCACACGATGCACGCTAACTGTGATGTGGGCGTGGGACCCGATGGTTCCCTCTTGTCC
CrLEA2-26	AATTACAAAGACAAGAGGTGCCCCGTTTATTTCTCTTGA
	ATGAAACCCAATCACAACATAACAATGCTCGCCAAGACCGATTCCGAGGTTAGTAGCCTCAGTCAGTCGTC
	CCCGGCGAGGTCTCCCCGACGGGCCGTCTACTATGTGCAAAGCCCATCACGTGACTCCTCCAACGACGGCG
	AGAAGACAACCAATTCATTCCAGTCCAGCCCGCTGCAAAGCCCATTGGGCTCCCCTCCCCACTCCCACTCC
	AATTCATCTTTAGGCCCGCATTCTCGCGAATCTGCCTCCACCCGCTTCTCGGGCTCCCGAAAGACCAACGA
	GTCCAACCGCAAGGGCCCATGGAGGCCCTGGAAGGATCAGTTCCACGCCATCGAAGAGGAAGGCCTCCTCG
	ATGCCCATGACGCTGCACAACATGGCTTCCCTCGTCGCTGCTATTTTCTCGCATTTGTTGTGGGCTTTGTA
	GTGCTCTTCTCTGTTTTCTCCCTCATTCTGTGGGGCGCAAGCCGCCCCCAGAAACCCGCCATTACGCTTAA
	GGTTCCATTCTCAACCAAACTTTGTAAAAATTAATTGATTTTATTTATTTATTTATTAAAACATGTGTGTG
	CTTTGACAGAGTATTACCTTTGATCAATTTGTGATTCAAGCGGGTGCGGATGTGTCAGGAGTGGCCACAAG
	CTTGGTATCCATGAATTCCTCAGTGAAACTAACCTTCCGTAACACGGCTACATTTTTCGGAGTCCATGTGA
	CATCAACTCCTCTAAATCTCAATTATTATCAGCTTACTCTGGCCACCGGAAGTGTAAGTTATATTATATGG
	TTGTGACTCTTGAATAATTATAATTTTGGTTTATAATGTGAGATTTGATATGCAGATGCCAAAGTTTTATC
	AATCTAGGAAAAGCCAGAGATCTGTTAGGGTGATGGTGAAAGGGAGTCATATTCCGCTGTACGGAGGGGGA
	GCCAACCTGAACAGCGTGAATGGTGCGCCAGTTGAAGCGGTACCATTGAAATTGAGCGTGATGGTAAGGTC
	AAGAGCTTATGTTTTGGGGAAATTGGTGAAGCCCAAGTTCTACAAGAAGATAGATTGCTCCGTCGTTATGG
CrLEA2-27	ATCCGAAGAAGATGGGCAAACCCATTTCGCTGATGACCAAGTGCACTTATCAGTAA
	ATGACCAATAGAGGGGGAGAGAGAGAGAGAATGACCACAACAGGTAACCAACCACAACCACACCCACATCC
	ACAGCCACAGCCACAGCCACAGCCACAGCCACAGCCAAACGGTGAGCGAAGACAAAGGCCAGCACCAGCAT
	CAGCACCAGAAACACCGGCACAATATTCGGCTTCTTCAAATGGAAATGGTTACAGGCAGTACAACCCAACG
	ACGCCGTCTCGTTCGTCGTCCTCGGCGTCGTTTAAAGGGTGTTGTTGCTGTCTGTTCCTGCTGTTCTCGTT
	GCTGGCACTGTTGGTGTTAGCGGTGGTGCTGGTAATAATCCTAGCGGTGAAGCCGAAGAAGCCACAGTTCG
	ATCTTCAGCAAGTTGGGGTGCAGTACATGGGCATAACACCCAATCCTCCCTCCACCGCCTCCCTCTCCCTC
	ACCATACGCCTCCTCTTCACCGCCGTTAACCCAAACAAGGTCGGCATCAAGTACGGCCAGTCTAGCTTCAC
	CGTCATGTACCGCGGCATCCCCCTCGGCAAAGCCTCCGTTCCCGGCTTCTACCAAGACTCTCACAGCGTCC
	GACAAGTCGTCGCCACCATCGCCGTCGATCGGGTCAATTTGCTTCAGGCCGATGCCGCCGATTTGATCCGA
	GACGCTTCCCTCAATGACCGCGTCGACCTTCGTGTCCTCGGCGATGTTGGTGCCAAGATCCGTGTCATGAA
	CTTCGATTCTCCTGGTGTTCAGGTACTCTTTACCTTTTCCTCTTCAAATTACATCTTCCTCTTCCCGGAAT
	CTCCACATTCTTCTCCAACAACAACTTTCCCAATTCATCTCACATTTCCCCTTTTCTTTATTAATCAATTA
	AACTAATTAATTCAACTTAAAGTGATTTTGATATCTCTTACGCTTTTTCTTTCTACCTCTCTCTCTCTCTC
	TCTTAATCTACAATCTTCGCCTGTGTTGTTCAGACAGAGAGAATGAGTGTGGCGGAGGGTGGCTCCTCTAC
	CTGTAACTGCGAATTCCACTACCCTGTGTCGCACTCTGCTTTTTCTTTACTACTCTTCTTTCTTTTCTTTT
	TCCCTCTCGCTAACGTGCGCCCCCGAGGACACTGCTCTACTATTTCACTCCCTCTCCCTCCTCCTCTGTCA
	TAACGTGCGCCGAAAGTTATTTTGTTTTAATATAAAGGATATTATTGTTATTTTATATTACTGATAGTGAT
	GTTTTAAAAACTTTATTTGCTATTTTGTACCAAATTTATCTTCAACATACTAACACTTACTCGTACACCCT
	TCTTAACTCCCCATCTCCTAAGATTTGATGCATTTTCTATTTTTTGGGCACATTTTTTCAGTTTCTGCTGC
	AGCAGCACCGCTGCATCGCATTCGCATATTTTTTGGAGACATCTTTCGGGGGTGTAACTTTCCACTTTTGT
	GTTTTGGCCTCTTTCCTTATGGTTTTTGGTTTATTTTTATTCCCCCTGTCATTGTGTGCGAATGCTGCCTC
	TTTATGAAAGGAATTTTTTTCCATCTCTGGCTACTATTTTGGCTCTCATCCCTATTTTTTTCTTAACCTTT
	ATAGGTTTTCTTTCGTATGAGTCATGTCATATAGTAGATTACCCTTTTATAAAAAATATTGATATATTATT
	TTTTTTCTCTGTTAGTTATAAAAATTATGAACTATAGTTCATAAAATCGAATCCTGGGGCTCTGTCTTTTT
	TTTTTTGCCCTTAGTTATGGTTTCTGTTTAAGTGCTTTTTTTTCACGGTCTTTTATGCATTTGTCTGGATA
	GCATGTTGAGACATTCCAATAGGTTCGGTGTTGTGTGCTTGTAGAATTATTTTTTTTCACCTTGATTGGGG
	GACTTTCTCACTTTTTTTGGGAGGAGGGGGATAACTATAGAAATCCTTAATTTCGAGGGTTCGCATATTGT
	TTTTAAAAAATGTACTCCTTAAATAGGGGTGAAATGGGAATTTGGAATAAAAATAAAACGGTGGAATTTGG
	GGGTTAGGGCTCAACTTTAGCCAATACCGAATGATTAGAATGAGCGAAGAAAATGATAAAATTGTTAGGTC
	CCACCAGAGTTCAGCCTGGGTCCAGGACGCTGGATTCGTAGTCCAGAGTACCAACCACTACACTATGGTGT
CrLEA2-28	CAACACCCTTTACTATACTGGCTGAAGCTCAATAATGTTCATTTTGGTGTTTGTCCTTAATGCCCTTTTAT

	TCTTCATAATATTTCGCTTTTCTTTTCCTTTTAATGCAGGACTCACTTTTCTTTTCTTTTCTTTTCTTTTC
	TTTTGGGGGGTGGTGGAGGGGTGTTCATCTATGGTGATGCTATTAGATGTCTCAGATTAATTAAACTATGC
	ACTCCTCATTTAACTTTGTTAAGAATAAACCAAAAACGGAGATTACAAATGTGAAATGGCACTTTCATTTT
	ATTTTTCTTTTTGGGCCAAGAGACGTCTTCAGCATGTGACTACACTGTCTGTGGGGCACATGGGGTGGGAG
	TGGAATCACCCTTCTGTGTCCACATTGTGCCAGTGTGAACATTTTAACTCCCTACACTTCTTTTTGTACTG
	TAAGCAATAAAACTGTCTTTGGGTGCCCATATAATTACTCCAACTTCCTATTTTCGTTGTCTCCCCTGTTC
	TCCCTCTTTGTTATAGAAATCCTTTTTTTTTTTTAATTTAAATCTATTTTACTTTTACATTAGATTAACGA
	CATGAACTCAACTGCACAGGTCTCCGTGGATTGTGCTATAGTGATCAGTCCAAGAAAGCAATCTCTGACTT
	ACAAGCAGTGTGGATTCGATGGATTGAGTGTTTAA
	ATGGCTCATCCTCCACCACAATCACAGCCTCAAGCAGGGAAGCCAAACCGCTCAATCCTGCTCCGTTACAT
	TGCAATGATAATTCTTTTCTTAATTATCCTTGTGGGTATAGCTGTGCTCATAATTTGGCTGGTTTTGAAGC
	CGAAACGGTTGGAGTACAGTGTGGAAGATGCTGCAATCCACAAATTCAACTTAACGGATGCAAATCACTTG
	TATGCGAACTTTGATTTCACCATAAGAGCATACAATCCCAATTCAAGAGTTTCTCTGTACTATGACTCTGT
	GGAGGTGGCAGTGCGTTATGAGGATCAAACATTAGCCACCAATGCGGTTAAGCCATTCTTCCAGTCACATA
	AAAATGTGACTAGGCTGCATGTGGGACTCACTGCTCAAACCGTGGCTTTGTATGATTCTGTGCCTAAGGAC
	CTTAGACTTGAGAGGTCCTCAGGGGATATTGAGTTAGATGTGTTGTTGAGGGCAAGGATACGCTTCAAGGT
	TGGCGTCTGGAAGTCAAAGCATCGAATTATGACCATTTTTTGTTCTCCTGTGTTCCTCACATTTTCCAAAG
CrLEA2-29	CCAAGAGTTTCCAAAGGACACCTTGTGAAGTACAACTCTGA
	ATGCCATCCCTCTCACCCTCTCCTCTTGAAAAACTTGGCATACCAAAACAACCAAGGAATCAACATTTCCG
	GGAATTAGAAGCACCAAATTCCCTTAGTAACACTCTGGTAATACTTGGGCAACCTCACCTTCAAAGGACCA
	AACCAATTATATGGTGTGCTGCAGTGCTGTGTTTAATATTCAGCCTTGTACTTATTTTCTTTGGAATTGCA
	ACATTGATTTTGTACCTTGCTATGAAACCACGAAACCCAACATTTGACATTCCCAACGCAAGCCTCAATGT
	AGTGTATTTTGACTCACCACAGTACCTCAATGGTGAATTCACTCTCCTTGCAAATTTTTCTAATCCTAACA
	GAAGATTTCACGTGAGATTTGAGTCCTTAAAAGTCGAGCTTTTCTTCTCGGACAGGCTCATATCAACACAG
	TCAATCAAGCCTTTTACTCAGAGGCCAAAGGAAACAAGGTTGCAACCGGTGAACTTCATATCCAGCTTGGT
	GTTTCTGCCTCAAGATGTTGGGGTGAAGCTTCAAAGGCAGGTGCAGAACAACAGGGTCAATTACAATGCAA
	GGGGTACTTTTAAGGTGAGGGTCAACATGGGCCTAATCCATTTATCTTACTGGCTCCATAGCATATGCCAG
CrLEA2-30	ATAGAAATGACTAGTCCACCAACAGGAATTCTTGTAGCTAGACAATGCATAACAAACCGATGA
	ATGTCGACATCTGATAAACCAGAAATAGTGGAAAGGGGTTCTAAGGATGAAAAGCACAAGGAGGGTGAGAA
	AGAGGAAGGCAAGGGTGGATTTATTGAGAAGGTGAAGGATTTCATTCATGACATTGGTGAGAAGATCGAGG
	GAGCTATCGGGTTTGGGAAGCCAACCGCTGATGTTACTGCGATACACATTCCATCAATCAATCTTCACAAG
	GCAGATCTTGTTGTTGATGTGCTCATAAAAAACCCAAATCCAGTGCCAATCCCTCTGATTGACATAGATTA
	CTTGATTGAGAGTGATGGAAGGAAACTGGTTTCAGGATTGATACCTGATGCTGGTACTATCCATGCGCACG
	GAGAGGAGACTGTCAATATTCCTGTTACCTTGATTTATGATGACATCAAGCAAACATATAATGATATTCAA
	CCCGGAAGCATCATTCCTTATAGGGTGAAGGTTGATCTCATTTTTGATGTTCCTATCTTGGGAAGGTTTAC
	ACTACCTTTGGAGAAAACTGGAGAAATTCCCATACCATACAAGCCTGATATTGATCTGGAGAAGATTCATT
	TTGAAAGGTTCTCTTTTGAGGAGACAATTGCAACTCTTCATTTGAAATTGGAAAACAAGAATGATTTTGAC
	CTGGGCCTCAATGCGCTTGATTATGAGGTCTGGTTTGGTGATGTTAGCATTGGAGGTGCAGAACTCACCAA
	ATCTGCAAAAATTGAAAAAAGTGGAATTAGTTACATTGATATTCCTATTACCTTCAGGCCAAAGGATTTTG
	GTTCTGCTGTCTGGGACATGATTAGAGGAAAGGGAACCGGTTACACCATGAAAGGAAATATTGATGTTGAC
	ACTCCCTTTGGGCCTATGAAATTGCCCATCAGCAGAGAAGGTGGTACTACCCGTCTTAAGAAAAAGAAGGA
	AGATCGTGATTATGATGATGATGATGATGATGAGGTTTGTTAACTTCTTCTAACTATATCTTGTTCCTTTC
	TATGTTTTCAATGTTCTCTAGAAAATGAAAAGGTATTGGTATGCAACTATGCATTTGTATGCTGGTTAACC
	CATCTGCATGTGCAATCATTTATTCTTTAATCATAAATAACAAAATGTCTTCTTATTTGTTTTTGTGCATG
	CGTATGTATGGTTTCTTCTATAGGCAACACCATTGTTGATAGCTAGTGTCAGGCATATTTTCAATATGATA
	GATATGTTTCAGAATCAAGTATCATAAAAGTTTACCTTTGCTTAGTCCACCATGTTATGAATGTTCCAGAC
	TCACATGATTGTTTTCTGTGAAGGATGCGAGTAGTCCAGACCAAAACTCTGCTTAATGTGAAAGCCGAGAA
	TAGATTTTGTGAACAATTTGTTGTCTTAAATTTTCTATTGAAAAAAAAAAAACAGGAATTCTTGTATAAAA
	TCAGGCTGGTTCTCTAAAATAACTCTTGGTAATGTCCTAAGAAAATGAATATATTTTCTCTATCTTTTTGT
CrLEA2-31	ATTTGTTTGTGTCATAAGTTCATAACATTTGTCTATAATCTCTTTCTTTGCAGGATTAA
	ATGGCCGACCGAGTTCACCCTCAAGACTCGCCTCCTGTTTCCGGAGAGTCGCAAACGACGTCGTCTCAGCA
	GCCTGCCATCGTTCAGAATCCGGCGCCGCAACCACTGTCGGAGAAGCCTGTTCCTCCGCCGGGAACCTACG
	TCATCAGGATTCCCAAGGACCAAGTCTACCGCGTCCCTCCGCCGGAGAACGCCCGCCGTCAAGACCAATTC
	ATTCGCCGGAAACACCGTCGGAGCCGATGCTGCTGCTGCTTCTGCTGGCTCATCGGATTTATCTTCATTCT
	CATCGTGCTCCTCGGAATCGCCGCCGGAGTTCTCTACCTAGTTTTCCGTCCGGAGGCGCCGAAATACACCA
	TCGACGGCATCGCTATCAGAGGAATGAATGTCACGTCGCCATCTTCTGCGGCGGCAGTTTCGCCGGAGTTC
	GACGTCACCGTCAAGGCCGACAACCCCAACGACAAAATTGGAATTCGTTACGAGACTGATAGCTCCGCCGA
	AATCTTCTATAACGACGTGAGGCTGTGTAACGGCGCTCTTCCGGCGTTTTACCAGCCTTCAAATAACGTAA
	CGGTGTTTAAGACGGTGTTAAGGGGAAACGGAATCAAGCTGAGGAGCGGAGACCGAAGCGCGTTGCTGGAG
	GCCCAGACCAAACGGAAAGTGCCGTTGACCGTTAGTCTTAGGGCGCCGGTGAAAATAAAAGTGGGGTCCGT
	TCAGACGTGGAAGATTACCGTTAAGGTGGACTGTGATGTAACGGTGAATGAGCTAACGGCGCAGGCCAAGA
	TTGTTTCGAAGCATTGTAATTATAACGTGGATCTTTGGTGATGAAAACGTGAGCACTATTGGGTGTTTTTT
	ATTTTTTTATTGTAGAGATAGGATGAAACCCTTTTGAGATTATAAATTCTTCTTAATAACATATTATAGTG
CrLEA2-32	ATATTTGCAG
	ATGGCAGATCCTCAGAGAATTCACCCTGTCCATGATGTGGAGGCACCAAACCACCCTACAGCACCATTGGT
	TCCTAGAAGCGTGTCTAAATCTGATGCAGGTGATCCAGAGAATGTGGTGCACCACCCTCCTCCTCCTACTT
	CTCTTCCTTCACAACCTGTGATGCATTCAAAGCCACCAAAGAAAAGAAGAAGTTGTTGCTGCAGGTTTCTG
	TGTTGGTTTATAAGCATATTGCTGATTCTGATTGTGGCTATTGGCATTACCATTGGGATACTGTTCCTTGT
	GTTTAGACCAAAGCTTCCAAAATACTCAGTTGATGAACTTAGAGTCACACAGTTTGATCTTGCTGACAACA
	ATAGCCTATCAGTTACTTTCAACTTGACAATAATTGCTAGAAACCCAAACAAGAAGATTGGAATAGACTAT
	AGAGGTGGAAGTCACATAAGTGCTTGGTATATGGACACAAAACTATGTGAAGGGTCTTTGCCAAAATTCTA
	TCAGGGTCACCGCAACATCACAGTGCTTAGTATCCCTCTTATAGGCCATACACAGGATGCTAGTGGCTTGC
	AAAGTACCATTCAGCAGCAGCTGCAACAAACGGGTAATGTTCCCCTCAATTTGAAGGTCAAACAGCCTGTG
	AGGATTAAGCTTGGCAAGTTGAAGCTGTTCAAAATCAAGTTCAGGGTCAGGTGCAGGCTTGTGGTGGACAA
CrLEA2-33	CCTTAATGCTAATAATTCTATTAGAATTCGGAGCAGTAGTTGTAAATTCAGGTTTAGGCTGTGA
CrLEA2-34	ATGAATTGTAGACCTCGTTGCAGCTTCTGCTGCATCTGTTGCATCTACACCACAATCTACGTCCTTATGTT

	CATCTTAATGCTTCTTCTCATCATCTTCTGGATAGTAATCTCACCCTCAAGTGTCAAATTCCATGTAACTG
	ACGCCACCCTCACACAATTCAACCTCACAACCAACAACACCTTACATTACAACTTGAAGGTCAACATCACA
	GTCAGAAACCCCAACAACAACGTGAAAGTATATTATAGAGCTATCACAGCAATAGCTTGGTACAAAGATAA
	TGACATTGGTTTGGTGAGTTTGACACCCTTTGACCAAGGCCACAAGAACACAACCTTCCTTCAAGCAGTGT
	TTATAGGACAAAGTGTGATTAAGCTCCAAGCTAAACAACTTGATGAGTATAAGGAAGAAACAAGTGTTGGA
	ATCTACAATGATTTGGCTGTGGATTTGGATCTTACAATCAGAGCTAAGTTTGGAAGGTTCAGAAGCAGTCA
	TTATAATCCACCAATTGTGCAGTGTCGTCGCTTGAAAGTTCCTTTGATTTCCAATGCTAAAACAACACCTC
	CTTTCAGTGTCACAAAATGCAGCACTGGTTATTTCTTTAAAGATCGTGATGCTGATGCAGAAGGCTGA
	ATGTGTTGCAAACCCCGCAGCACCTTCTGCTGCATCTACTGCACCTTCTACACTCTCATGATCATCTTTTT
	CCTTTCTGTCATCATCTTCTGGATAATCATCTCACCCTCAAATGTCAAGTTCCACATAACTGAGGCCTCCC
	TCACACAATTCAACCTCACAAACAACAACACCTTATATTACAACTTGAAGGTCAACATCACAGTTAGAAAC
	CCCAACAACAACATCATAGTATACTATAGGAGAATCAAAGCAATAGCTTGGTACAAAGACAATGACTTTGG
	TTGGGTAAGTTTAACACCCTTTGATCAAGGTCACAAAAATACCACCTTCCTTCAAGTAGAGTTTAAAGGGC
	AAAGTGTGATTAAGCTCAAAGCTCAACAACTTGGTGAGTACAAAGAAGAAACAAGTGTTGGGATTTACAAT
	GATTTGGCTGTGGATTTGGACCTTAGAATCAGAGCTAAGTATGGAAGGTTCAAAAGTAGTCGTTTCAATCC
	ACCAATTGTGCAGTGTCGTCGTTTGAAAGTTCCTCTGATTTCTAATGGTAAAACACCAACTCCTTTTAGTG
	TTACCAAATGTAAAAGTGGTTCTTTCTTTGTGGACCGTGATGCTAATGCAGGAAGAGCCTGATATACATTC
	CTCTATACTCTCTAAAATCTTAGTTTACACAATCAATTTTAAACAATTTTTTTTCCATTACCTCAATTTAC
	ATAGTTGTATGAAGTGTTACATGGATATAAATATATAATAAAAACAAATCCTAGATCATGTTATGTACCAC
	TTCTATTTGGTCTATTTCTTTTGGTTGTTTAATTACTTGTTCTAGTCCTAATTAAGATCTCTTGTGTATGA
	TCAATATTATATATATAGTGCAGAGTTCATAAATGTGAATTCACACATACGCCGATTATATAGAATATATT
	CGATATTGATGGCGTCCCACTCAACCAAAAACTAAAGTCTCAACCACGACATTATAGGATGGTTTTCAAAG
	GGTATAAGAACTTTTTTTTTAAAAAAAAATTGACAATTTTAACAACAATAATATTTTTTTTAAATTATTA
	TTTTATTTAAAATTTATATTTCAACTTTTTTGTGATACAAAATTAATTATTGAATAGTTGTAATAAAAAAT
	TGTATTTTTTATGGAGAATGAAACCAATACATTTAAACTTAATTTCTTGTAATGTTATAAAGGTTAAAAGA
	ATAAATTATTTAATAGATATTAATAGCTAATTAATAGAAATAGAAATAGAAAAACAAACTATAAAATGAGG
	TTTGATATTATAAAAAATTATTATTATTTAATAATTAATAATTTTTATTTTAAAATATTAATAGTAAGTAA
	AAAAATTATGAGGTCTAGAACCAGACTTCTCAGCTTGATAAAGAATATTTGATGTTGACATGAATGAGTTT
	AAGAAGAAAAAAATTACAAACTGAATTTTTATGTAAAACTAATCGTTTGAAAATGATATTGACTCATTTAC
	ACATCATTTTTATTTTTATTTCTTAAGATGAGTTGATTTCAAGTTTATAATTTATTGTTGTCCAGTTTGTT
	AATAAAGCTAGAGAACCCTCTATATGATAAGGATCAATATCATGCATTGGATTAAGCATTCAATTACAAGA
	ACTAAACATTAAATTATTATATGGCTATATTTGTTAATAATTTTAAAAATGAATCTAAAACCCGCCCACCC
	TTTTTGATAAATTATTCTGTTATATATAAATAACTACTCAAGTTGTCTCATGTTTCAACTCTTTTTAGAAC
	AACCGAATGATATGATCTATTCTAAATATATATATATATATATATATATATATTTCAATTTGAAGATTCTG
	AATTGCTAATTCTGTGGAAATTTTTTTATAACTTGCTTGTTTAACCGTTTTCAAAATTAGAATTGATTATC
	AAACAAAATTAACATTATTAGATTATTTTTTTCAATTTTTAGGAAATCAGTTTGTAACATTTATAAAAAGT
	ATACAAGCGCTAATAGGCAATGATTCATTATTATTCAATGGACATTTAAATATATACCAGATGGGGTTGCC
	CTCCTCATTACGCGTAGGAAGAGACTAAAATTTGTTTAAAGCAGATTCATTATCGTAAAAATAGATAAAAA
	GACCTAAAGTTCAAGGTAAAATTTTCCAAATATGCATATACTTTTAACCAAATTATTGCATTGGGAATTGC
	CAAATTATATAATTATAATTAATTTTACTATATTTTCTGCATATATATTTTAATTATGTTACTTGACTTAC
	TTTATAATGTTTAAAATTTTTCTTTATTTCCTTTTCTTTTTATTTCTATATTTTTCTCTTTTATATACCCA
	TTAAATTTCATATATACTTTTTATCTCTTTCTTTATCCTAAATTAACTTCTCCACGTTATAGAAGATCCTA
	CTCCTTTATAATGATACCAAAGACAAGTTAATCACTAGAAAAAGAAAAAGAGTTAACTAAGTATGCTGAGA
	TATGTTCCTGCTATTTAAAAGTCTTAGCATGCGGTGCCTGATTTAATAAACCAATCATCACCGTGTCAAGT
	TTTTCAAAACAGAAAAATGAAGAGAAAATGCTCCCTAAACACACAAGCATATCCACACTTATTTTATGCCT
	ACTTGTCCATCCTATATAATATTACTAGGATATTAGGAAGCCAAGATGCAGAAGTTCTCAACTTCTACAAT
	TATTAAGAATTGAATATCCAACTTAAGCTTGACTAATTAAAAATTAATAATAATATTTTTTTTTATTTGAGA
	TTGAAGTGCTGATGTGTAAGTTTATTCAATTAAGTCCCATATTGAAAAACTGAAAGAAGAAAAAATTATAA
	ATGCACCATTTGAGCCCAACCCTTCTTAGATGCTGCCAAACATATTTCTCCCAAATTTGAGTAAAGCTTGA
	ACACAAGAAAGAAGTGAATGAACACGAAATGATACCAACAATTTCTAATGCATTTTCAAAATATAATATTT
	TATAGTACTTTTATATCCTCAACGGAATTAAGGGTTCCATGTTAATCAAAGCTTTGAATAGTTCTCCAATC
	TTCATTACTCAGCTTCAAAACTCAAAAGTCAATGCCTCTCTGTACCTTGTATAATGCAAGGAAATCACCAA
	GAAAAAGCTAAGCAACAAACTATACACCAAAACCATTAACCATCAACCATCAACCAATAACCCCTACAAAT
	ACCTATCAACATTTGACACATCAATCACATAAAGCTTCTCCTACCAATCATCACACACACTATCTATCCAT
	CACAAATCTCTCTCACCATGTGTTGCAGATCGGGCTTTAGCTTCTGTTGCTTTTGCTGCATCTACAGCACA
	TTCTACATGTTTATGTTCCTCTTCACACTTTCCCTCATCATCTTTTGGATAGTAATCTCACCCTCAAGTGT
	CAAGTTCCATGTAACTGATGCCTCCCTCACACAATTCAACCTCACAAACAACAACACCTTATATTACAACT
	TGAAAGTCAACATCACAGTTAGAAACCCCAACAATAACATCATAGTGTACTACAGGGGTATCACAGCAATA
	GCTTGGTACAAAGATAATGACTTTGGTTGGGTGAGTTTAACACCCTTTGACCAAGGCCACAAAAATACAAC
	CTTCCTTCAAGCAGTGTTTAAAGGCCAAAGTGTGATTAAGCTTAAAACAAAACAACTTGGTGAGTACAAAG
	AAGAGACAAGTGTTGGGATTTACAATGATTTGGCTGTGGATTTGGACCTTAGAATAAGATCTAAGTATGGA
	AGGTTCAAAAGTAGTCGTTTCAATCCACCTATTGTGCAGTGTCGTCGCTTGAGAGTTCCTTTGATTTCTAA
	TTCTAAAACAGCACCTCCTTTCAGTGTTACCAAATGCAGCACTGGCTATTTCTTTGCGGATCGTGATGCAG
	GAGACTGATATAATCCTATATACCCTTAAGTCTATTTGCAATCCATTTTTTTTATTACTTCAATCTGCAAA
	CTTGATATGGGTAGAATTTAATTTCAAGAGATCATTAAGGTTATGGTTCTTCTTCAGTTATATATATATAT
	ATATATATATATATATATATATATATATATATATATATATATATATATTATAGCCTCCTACCGCAACTTTTTA
	TTTTTCTTTAAAATTTCATTCTTTCTTTAAAATTTCATTCTGGGACCAACTAATAAATTTTGCGAGAACTA
	GTATTCTTAAGTTATAGTAATAAATCTTAGTAGTCATAGTTTATTAATTGATGAATATATCTAATCACTAA
	TTTTTATACACTTCCACCGTACATCATTCATTCAACATCTTTTACTTTTCCTTTTTTAATCATAGAACATT
	ATCTGTCACATCTATATATATATATATATTTTTAAAATTATAGGACGTATATACACCATTTGATACCCAATCC
	TTCTTAGATGCTGCCGTCCAGTTCTAAACATATTTCTCAAATAGCGAGTAAAGCTTGAACACAAAAGAAGG
	TGCATGAAGACGAAATGATACCAACAATTTCTAATACATATTTAAATATAATATTTTATAGTACATTTATA
	TCCTCAACCAAATTCATCAAGAGTTCCTTGTTAATTCAAATATATATTTTATAGTGCAAGGAAATCACCAA
	GAAAAAGCTAAGCAACACACCATACACCAGAACCATTAACCACCAGCCATTAACCCCTACAAATACCTGTA
	AACATTTGACACATCAATTACATAAAGCTTCTCCTTCCAATCATCACACACTACTATCTATCCATCACAAA
CrLEA2-35	TCACCCTCACCATGTGTTGCAGATCGGGCTTTAGCTTCTGTTGCTTTTTCTGCATCTACAACACATTCTAC

	ACCTTTATATTCCTCTTCACACTTTCCCTCATCATCTTTTGGATAATAATCTCACCCTCAAGTGTCAAGTT
	CCATGTAACTGATGCCTCCCTCACACAATTCAACCTCACAAATAACAACACCTTATATTACAACTTGAAGG
	TCAACATCACAGTTAGAAACCCCAACAAGAAGAGCATAGTGTACTACAGTTGTATCACAGCAATAGCTTGG
	TACAAAGATAATGACTTTGGTCTTGTGAGTTTAACACCCTTTGACCAAGGCCACAAGAATACAACCTTCCT
	TCAAGCAGTGTTTAAAGGGCAAAGTGTGTTTAAGCTCAAAGCTAAACAACTTGGTGAGTACACAGAAGAAA
	CAAGTGCTAGGATTTACAATGATTTGGCTGTGGATTTGGATCTTAGAATCAGGTTTAAGGAAGGAAGGTTC
	AAAAGTAGTCGTTTCAATCCACCAATTGTGCAGTGCGTCGCTTGA
	ATGACTGGGAAGGACGAGTTTCTCTCGTACTCGCCCCTTCCCTCCAATCCAAATCCATATTCTAATAACCC
	TAATCCTAACCCTAACCCTAACCCTTACCATTACCCCCAAAACGTCGTCGTTCTCCTTCCTTCCTATCGGT
	CACCTTCCCTCCGTCGCCGTCGTCGCTGCATCCTCTACTCCGCTGCGCTCTTCATCTTCTTCCTCCTCGTC
	GCCGCCGTCTTCATCTTCTACCCATCCGACCCGGAGATCCGCCTCGTCCGGATCCGACTCAACCACATTGG
	AATCCGAACCTCTCCGAGGCCCATTCTCGACCTCTCTTTCTCCATCACCGTCAAGGTTCGCAACAGAGACT
	TCCTCTCCCTATCCTACGATTCCCTCTCCGTCTCCGTCGGTTACCGTGGCCGCCAGCTAGGTTTCGTCACC
	TCCTCCGCCGCCGGCCGCATTAGGGCGCGTAGCTCCTCCTACGTTGACGCCACGCTCACCGTCAATGGATT
	CGAGGTTATCTACGATGCTTTCTACTTGCTGCAAGACATCGCCAGAGGTGTTATCCCGTTCGATACCGATA
	CTCGAGTTGAAGGGAATTTGGGGCTTTTATTCTTCAATATTCCTTTGAAGGTGCCTCATCTGTGAATTTCA
	GTTACAATTTATTTATTTTTTAATTTCAATTTCGATTCTGAGTAGCTTGGGGTTTTTCCCTTGATTGTGTT
	CACATTTCATAATTCGTTTGTAGATAGCTTGTTTTTATGATTTTAATTCATTGACATGATTAATGTGAATG
	TTTTGTGTATTTGATAGCTTTATATCTAAAAAGTGTATGCTCAATCCAGGCTACTGTGTCATGTGAAGTGT
	ATGTCAATATAAACCAGCAGACAATTGTACGTCAAAACTGCTACCCTGAGGTGAGGATTTTTTGTGTCTTC
	CATCATACCATAGTTTGCGTATTCATTAACCATGCTCATGGACTCATCCTTTTCATTTATGTATAGAAATT
	AATCCTTTTGTCGTTTCCATTATTTTGCTGAATTGCCTACTTGTTTTTATGTGCAAATGCAATGTACCCAT
	GTCTTTTGTATCTTCCTGTGTAACTATTTTCAAATCTGTATTCAAGTTTTATTTCCTTTACCATCAAACAA
	TTACATCATGTTGGTTCAGTCTGAACTTAAAAAAACAATTGTGTATCTTTTAGAAGCTATTGAAGGACACT
	AAAAGAATAAGTTATTATTTCTTCAAAATAAGCTAAGCCAAACATGCACTTGCCTTTGATAACCTTAGATG
	GTTACATTAGCCACCCCTAATTTGTAAAGAGAGTTAAAACTCAGGGCGAAGCCAATATGAAAAAAGATGGC
	CAATATTGAGCAGTCCTTTTCTTTGTTAGGCTAGAAAACTGCATTTTCGGGCATTTTATGCTATTGCTTGT
	TAGCTGGAACATAACAAAAGAATTGAAGCGTTTAGTTTTACTTTAAATCATTGCTTGCCCTTCTTCCGTCA
	ATGTTTTGAAGATACAAACAAGCAAAAGGTATAAAATGTTGCCATACTATATGTAGCAACTATGTATATTA
	ACACGAGCACTTATCTTACTTTCTTACCATAGAAATCTTGTTTGTCTTAGCTATAATGCATAACTTATGTT
	CTAATACCCAAATAAGAAAACCAGAATGTTTGAGAAACTGCTTAAATTAAACTTAAATTACTATGCCTTAA
	GATTCTCCTATATGCAAACTTGTGATCATTGAGCGCATCATTTTCATTATTATAGATTGAAACACATTTGA
	TTCACACTTTGGTTGTAAATTGTAATTGGAAAGTAGTATAGAATCCTTTTCTTTTCCCCTTTCTTATATGC
	TATGATATTTTGCTGGCCATGTTTAGTTACTCTTTATTTCACTGGCTAATCTATTCGTTTTCTAGCTGCAT
	GCTTGAACTAGAGATACATTCTCATAAAACTGTTTTTTCCTATGGCTCTAAGAAATCCCGCATAACAGACT
	TAAAACCTTAATGAATATCGTGCCTTATTAGGTAGGAGCTTATTGGCCTTAAATATCATCATTGTTTCTTA
	GAAATTAAAATCCTGGAATACTGTGTCGAGGGTTTGTGTTTTCATAGACCCTCGGCACCATTCATCTTCAT
	CTACAGTTGCTTTTATGAGGTCGAGTTAAATTATGCTATTAGCAGTTCGTTTCCTCTACTTTCAAGTTTTG
	GAAATGCAGATCATCTGGCTATTAGGTTTTGTTTTATAATTTAAACTATTTTTATAATGCATGTTTTCATT
	TAATATATATAGAATTTGCTAATCCATTATTCATCTGCTTCAGTCACTGGGTGATCCGCTGGATCAGAGCA
CrLEA2-36	CATATATTGGAGTTGCAGATACATGA
	ATGCACGCCAAGACCGATTCTGATGTCACGAGTTGTGATCCATCATCGCCAAGATCTCCAAAACGCGCCGT
	TTACTATGTGCAGAGTCCCTCGAGAGAGTCTCATGATGGTGATAAATCTTCCTCTATGCATGCCACTCCAG
	CATGTAATAGCCCCATGGAGTCACCTTCACACCACTCGTATGGTCACCATTCTAGGGCTTCCTCTTCCAGC
	AGGGTCTCCGGTTCCTACAACTCTTCTTGGGGAAGGAAAGGGGACCGTAAACGCAATGATAAAGAGTGGCC
	TGAATGCAAGGTGATAGAGGAAGAGGGTGGTAATGGTGATTTTTACCCTGATAATAAAGGCTTCTCAAGGA
	GGACTCAGATCTTCATTGCTATGATTGGTTTTGTCTTGATTTTCGGTGTGTTCTGTTTGATCATTTGGGGT
	GCTAGCAGGCCTTACAAACCTCAACTCAGTGTCAAGGTATAGTTAGATTTTCTTTGCTTTTTTATACTAGG
	AATTCCCACTTGCTTTACATTATTATTATTTTTATTTAATAAACAAATGGTTGACTTAGAAAGGTGTAGAA
	TAATATCTTGTCTTGTTGTATTGTTAATCGGAGAACAAGCCTTTTTATTTTTTTAATATAGTTAAAAGAAA
	AAGATAGGGATTTGAGATGGGAAGACTATGAATCAGAACCTAATATGGTGTAATTTATAAAACAGGACTTG
	ATAGTTGAAGTCATCCTGGGTGACAGAACTATTTGTAGTGAAGACTGAAGAGTCACCACAATCGATTTCCA
	GGAATTGCAGTGATTTGAAAGAAATGTTGCCTATAGATTTTCTGTTGTCATTCAGTTTCAATGGTGTACAC
	AACCATGCAACTTGTGACTCCTTATTGATTCTTCCTATTTTATTTATTTTAAAAAAATTCCCCATTTTTCT
	ACCTGACAATTTAGAATTGCTTGTTTCTCCCATCTATCTTCTATTGGCTATATGTTATCGATGCAAGGAGA
	AACAAGTTCTATTTTTCTTTGGAACTCAAACTATCATGCTTTTATTATTCATCTTGAATTTTCTTTTACAT
	TATTAATGAACTTTCCAGTGAGCCTGATGATTCACTTTTCCAGAGTTTGACAGTACACAACTTCTACTTTG
	GAGAAGGGACAGACATGACAGGAGTTCCAACTAAGATGCTGACAGTGAATTGCTCAATGAGAATGATGGTG
	CATAACCCTGCAACCTTTTTTGGCATTCATGTCAACTCCAAGGCAGTGAGTCTTATGTATTCTGAGATCAC
	AGTTGCAACTGGTGAGGTAAGAATTAATCCCAATTTCTAACACTGACCCCTACTCTCATTTAGGCTTTGTT
	TTTGTTTTATAGTTGGAGAATCAATTCTATCACTTTTCAATAAAAAACACACATACAAATGAAAATATATT
	GTTAAGATTAATTTATGCAAGCATTTTCAATCATATTCAATGGTTCAAAATCAATTCTACTCAACTTTAAA
	CCAAACAGGTACTTATTGTGCCTCTAATGTTGACAATGATCGTCACTCCTACTGTTAAATTCCATGTGCAG
	CTGAAGAATCATTATCAGCAGAGAAAGAGTCGCAGGACAGTGTTTGTGAACCTGCAAGGAAGCAAGGTTCC
	ATTATATGGTGCTGGTGCAAGCTTAGCTGGTTTGGTGGACAAAGGAAAAATACCAATGACGCTTGTCTTTG
	AAGTAAAGTCACAAGGGAATGTTGTTGGGAAGTTGGTGAGGTCCAAACATAGACAGCATGTTTCTTGCTCA
CrLEA2-37	GTGGCTATTGATTCCCACAACAACAAACCCATCAAACTCAAAAACAATGCATGTACATACGATTGA
	ATGAAACAGAATACAATAGGATCCAAAATTTTCTTGTGTACACCTGCATTGGTTTTTCCCAAAAAACAAGA
	TTTCAAAGGTAATCCCAATTGTTCTTATTACCCGGAACCAGAAACTATGGCTAAAAGAAACCTCAAAATCT
	GCATTTCTGTGTCTGCAGTTTTCTTTATAATTGTCATCACTGTGTTTCTGACCTTAATTTTGACCATATTT
	AAACCCAAAAACCCAGATATATTTCTCCACCCTGTTGACCTCAAAAACTTTCAGTTATTGTCATCCAATTC
	AACTAATGCACCACTAGGCATGGTGGTCACAATAGTGAATCCAAACTATGGAAGCTTCAGGTACATAAATT
	CCACTGGTTATCTTGAATATCGTAATACTACAGTAGCCAAAGTTCCCTTGGAGACAAAATCTGTTCCTGCG
	CGTACCAGGACCAATGTGAGCACTTCTGCAGGTATTATGACAGAAAAATTGATTGATGATCCAAAATTTTG
	GTCAGAAATTGAAGGTGGGACCTTCAATTTGACAGCAAAGGCCACATTGCCTGGGAAAGTGAGTATGTTCA
CrLEA2-38	ATATTTTCAAGCTTAAGGCCACGGTTCATATTTCATGTGATATCTCTATTAACATAATTGCTATTGATTCT

	GGTTCCACCTGCGTGGCCAAACTCAAGTTATGA
	ATGTCTAGCAAGGACTGCGGCCACCACGAAGAGGAGCGCCGGCAGCTTCTCCGCCGCATAGTTGGTGCCAT
	CGTAGCCTTCATAGTCTTAATCCTCCTCATAATCTTCTTAATCTGGATCATCCTCCGACCCACAAAACCCC
	GATTCATTATCCAAGACGCCACCCTCTACACCTTCAACTTCTCATCCACCTCACCCTCTGTAACTTCCCCC
	ACACCCAACATCCTCACCCTCACAATGCAAGTCACGCTTGCCGCACACAACCCCAACCACCGCATCGGCAT
	TTACTACCACAATCTCTATGCCTATGCCTCTTATCGTAACCAACAGATCTCCCTCGCCACTTCCATTCCCA
	ACACCTATCAAGGCCACAAGGACTTCATCATTTGGTCTCCGTTCATCTACGGTAACGCCGTTCCGGTTTCG
	CCGTTTCTGTTGAGTGCTCTGCAGCTCGACCAGAGTACCGGTACCGTTTTGGTCAACGTCAAGGTCAATGG
	AAGAGTCAAGTGGAAGGTTGGGACTTGGATCTCCGGGAGGTACCATATTTATGTCAACTGTCCTGCGTATA
	TTAGGTTCGCCGGAGATCGGAGTAATGCCATCGGCGGGGTGACTCCGGCGGTGAAGTTTCAGCTTTTGCAG
CrLEA2-39	AGTTGTAGTGTTGATGTTTAG
	ATGTTGAGCAACATACTTACCTGGACGGGGCCAATGGGTGATCCAGAAGGCCTATGGCCTAGGGAAGTAAC
	CTCCATTGCACTTTGGAGGGGTGCTTCTCTAAGGTCTGCTCAAGTAGCAGAGCCTACGTCATAATTTGTGG
	CAGTGGGGGCCTGCGTTCGCGCGGCCCCTACCTTGAAAGTTTAAAAATTTGTGTAATTTATTAGATGTGTT
	ATTAAGATATTTATTATGTTTTAATTAATGATTTATATGATTTTCAACTTTATTTTGTTTCAAAGTACAAT
	AAGCAGCCTATTAAACACTACTTCAACGTTTTCTTTTTCTAAAACCTACTATAATTAAACAAAAATTTAAT
	ATAATTTGATGTGGCAAAATATTTAAAAAATTTGGAAACTGAAATTTTAATTGACAGAAAATACCTACTTT
	AATTTTGTTTGTGTTTTTAATTTTTTTTTCTTCTAATAAATAATAAGAAATAAATCATTATCTGTATTTTT
	TTACACCCTTGGAAAGAAGACAACAGTTTGGGATGAGTTGCTTTCTTCACTTGAATGAACGTTGAAAATTG
	ACTCAGGAGCACGTGGTGTTGATAAAAGTGAGGGCGTTATCGTAAAATGATAGATCTCCGTGCTGATCTTT
	GGTCTCAATGTCTCTCATCTCTTCCTGAATATCCCCCAACTCATTCACAGCTTCTTGATTCTCCAAGTGGC
	GATGGGCAAAAAGGTAAAATGGAGTTGGAGCTCGGCGTTTGTGGGAGCAGCCTCAGCGGTAGCAGCAACAG
	CGATACTATCAGCGAAACCCAAGGACCCAACTTTTCACCTCATATCAATCAATTTCACTTCCCTCAAGCTC
	AACCTCCCCCTCCTCGACGCAGAGCTCCTCCTCACCGTCCACGTCACCAATCCCAACATCGCTCCGATTCA
	CTACTCCTCCACCACCATGTCTATTTTTTACGAGGGTTCCTTCCTCGGCTCGGCTCAGGTTCAAGCCGGCT
	CACAGCCTCCGCGTTCTTGCCAGCTCCTCCGCCTCCCCGCTCGCCTCCATGCGCTGCAACTCGCTCACCAC
	GCCACTCGCTTTGTTCACGACGTTGCGCGCCGCGAGATGGTCCTTGACGCCGCTGTTGATATTGCCGGCAC
	CGCCAAGGTTCTTTGGTGGGACCACAACTTCAAGGTCCACGTGGATAGCCGCGTCACCGTCGATCCCGTCT
CrLEA2-40	TTCTTGACGTCATTGATCAGGAAAGTACCTCGGAACTTGAAGTCTTCACCGCAGCGTAA
	ATGGGAGGAAGAATGCACACTAAGTCCGACTCAGAGGTCACCAGCAACAGCATCGACCACTCATCCTCGTC
	GCCGCCGCGTTCCCCTCCCCGCCGGCCACTCTATTACGTCCAAAGCCCTTCAAATCATGACGTTGAGAAGA
	TGTCTTACGGTTCAAGCCCCCTCGGTTCCCCTCACCACCATATCCATTACTACCTCTCTTCCCCCATTCAC
	CATTCCCGCGAATCTTCCACCTCTCGTTTCTCTGCTTCTCTCAAAAACCCTCGCCCTTTCTCCTCCTCCTG
	GAAGAAACTCCACCCTCACCCTCACCACGGCGTCGACGACGACGAAGACGACGACGACGACGACCTTCACG
	ATTCCTCCCGCAACCTGCGCTTGTACCTCTGCTTCTTCTTGCTTTTCGTCTTGCTTTTCACCCTATTCTCT
	CTCATCCTCTGGGCTACCAGCAAGACCTACAAGCCTCAACTCGCTATCAAGGTAAACAACAACTAACAATC
	AAATCAATATTTTTTCTTTCCCCTTTGCTTCATTGCTCTTTAATTAATGAAATCATTTCTGTTGGTCTCCG
	ATAATACGATCATGATTAATTTCTAAGAATTGAATTAATATTGTGGGGCCTCCTTGGAACAATAATAATTT
	TTTTTGTTACGGGAGAACAGAGCATAGTGTTCGAGAACTTGAACGTACAATCGGGAAACGATGGAACTGGG
	GTGCCAACAGATATGTTATCGTTGAACTCAACGGTCAGAATTTTGTACAGAAATCCTGGCACTTTCTTCGG
	TGTTCACGTCACTTCCACACCTCTTCAGCTTACCTATTACCAGCTTGCACTAGCCTCGGGTCAGGTATATA
	TTTTTCAATTAATTATTTCTATTCCTATAAAAATACATTCATTGCCTTGATGAGTCGGTTTCGGTTATGTA
	GAAAAAGTTATATAGTTTAAGTGTTGCATTGTTTTAAGAAAAAATGAAAGCAAAGAAATGAAGTGAGGATT
	GAAGGGTACTTATTGATGGTTTCGCAACGGCCAAAGGTGGGGACAAATAGGTGGGGACAAATTTATGCATC
	GATAATCAGTCTGTGGGAGCATCCACAGGCTTACTGAACAACTTCAATTTTATTGGTCCTCTTTATTGGAC
	TCTGTCTAAAAGCTGCCACCTGCTTCCTCTTTCTACATCTTCTTACGTGTGGGGACCCACCCACTAACACG
	CTTCACACTTCACACCATTTTTTTAAATACAAATCTTTATGATTCTTTTCTTTTCTTTTCTTTTCATCAAT
	TGCCATTTCCCACACACACTTTATTGCATAGACATTATACAGCAGCTCGTGGTAACTGGTAACTGGTAACT
	GGCACCCACTCACGTGACTGACTTTCACATTAATTATTGCATTACCATTACCATTACCATTAACGCGAATT
	TTCATGAGTTTATTGCCAATAAACAACTGTTTAATTTTTCAGTACTCTTTTCATTTTAAACATTCTATAAC
	CAATGTGGATAATCCCGTTACCCACCACACTCCACAGACATTCCAAGTCTCCGCTGCACCAAACACGCTTC
	AACGCGGCTCCTCTACCTACTGCACTTGTTTAACTGCTCGCTGTATCACAACCTTATTGTTCTTCGAATAT
	TTTAAAAAAAAACTATTATTTTTTAATTTTTCTCCCATACATATCTAAAACCGTACATTTCAAAATGAAGA
	CATACAGCTGTTATTTTGATGTTTCACAAAACATGTTTCATATTACAAACTTCAATAACAATCATCAGTCA
	TTCACATAGCGTAAGTGTATAATAATTCCTTGAGCTTTCTAGATACGGCATAGGGGCATTCTTTTTCACAC
	TATTATATAGTCTTGGAATTTAACAACCTTTTACGATTTTATTTACACAGAAAAATTATGATAATTTTTTT
	TTTTTGTAATAATTTGCTTAGAAATATATTGATTATTAAATTTCTTTTATATTCTCATTTTATTATGAAAC
	TTATGTTAAAGAAATATTTATCAACTTATGATATTATCAATTTAAATATTTATTATTATGGAAAATTTTAG
	AATAAAATTTGAATGTACAACATATTAAATATTTTGACTACTTTTCTCATACGCTTGATAAATAATTAGAG
	AGAACACAGAAGTAAGAGGATTAGGTGGAGAATATAGCATGTTTTGTTGGATATTTGGGATGAAGATCATT
	ATGCGTATGCATTGTTAGACACAGTAGAAGGGGTGTTAGTGTATCTCTGCAAACAGTTGAAACGGTTGTGC
	TGTGTGAGGACAGTTTATTCTGTTATTATAATTAGTTTTTCTTATTCTGGTAAGTTCCTTTTTGCTCTCCT
	TGCGTGTGCTCTCTCTTCCTTGCTGTACATAATACCTTCTGTATCTCACAAATAACATGAATGAAAGTTCA
	TTCTTCTTTCTCAGCTATGATATTATGCTAACAGGGGACACTCTGCATTTGCAACATGAACCCTGTTGATG
	GTACTAATTTGACAAGATTTTATTTTTCTACTGGCTAATAGATGCAGAAGTTCTATCAGTCAAGGAAGAGT
	CAGCGGAAGGTAGTGGTGGTGGTGTTAGGACACCAGATTCCGCTCTATGGTGGAGTGTCTGGTCTTCTTGG
	AAATTATAAAGAGCATATCGACAGTGTTGCATTGCCACTGAACCTGACATTTGCAGTGAGATCAAGGGCTT
	TCATCTTGGGAAGATTGGTTAAGTCTAAATTCTATCGAAGAATCAGATGTTCTGTCACTTTGCATGGCAAC
CrLEA2-41	AAACTTGGAAAACCTCTTAATTTGAAGGATTCGTGTGTCTACAAGTGA
	ATGTCATCTGATCCTTATGCCCATCAAAGATATGTCTCCCTGCAGAACCAAGGCTATGTATCAGGGCCTCC
	TCCTTATGGGTATGGCCGTAAGCCTCCACGTTACCATCACCACAGTGGTTCTGGTTGTTTATGTGGATGCC
	TAAAGTTCTTCTGTTGCTGCTTCTATAGTTGCTGCCGTTGCTGTATTTGCGCCTTCTTTATCTTCATTCTC
	ATTTTGGTGATCATTATTATGGTTTTATATTATCTTCTTAAGCCTGACATACCTTCCTACAATATTCAAGG
	AATTGATATCAACACTTTTGATTTAAAAATTGATAATAAGCTCTTTTCTAATATTTCCATTGTTGTGAAAG
	CGGATAATCCAAATGAAGGGATTGGGTTGAATTATTTAGATAATGAAGTTAGGTTATTGTATTCAGGGTCC
CrLEA2-42	CAACTTTGCTCTGGGGCGTTCCCTCCTTTCTTGCAACCAGAGAGGAACGTAACCACGTTCAATGTAACATT

	GAAGGGGGAGAGTGAGTTTGGCCCTGAAATGCAACAGCACTTAATGCAAGAACAGGATAAAGGGACAATTC<br>ATTTGTTAATAGCAGTGAGGCTTCCCATAAGGCTTGTGGTTGATGACCTTATTCATCTGAGGAAGTTCGTG<br>GTTAATATAAATTGTTCAATGATTATTGATCACTTGGAACAAAACAAAAAGCCTAATATTTTGAGGAAAGA<br>CTTCATCTACGGAATCGAGTTTTGA
CrLEA2-43	ATGGTTTCTAGAGGCGGCAAAATTTGCATCGGCGTGTCTCTAATATTGGTTATCGCTATTGGCACTGTGAT<br>TGCGACATTGTCACTGACAATCTTTAAGATCAGAGATCCTGTGATCAGTATCCACCCAGTTGGTCTCGAAA<br>ACTTAAAGTTTTCTCTTAATTTTTCGTCGAATGTGAGTCTAGGCATGATTCTCACTATGGGGAATCCAAAC<br>TATGCAAGCTTCGAATTCAAAAATTCGATCAGTCATGTCAATTTTCATAAGAACGTTGTAGCTGATGTTCC<br>AATTCAGGGAGAGGTAGTTCCAGCGCGTGGTACCATTAATGTGACCACTTCCGCGGGTTTGATGGTAGAAA<br>AGTTGATACTTGATCCCGATTTTTTCACAGATATTTCAACTGGGTATTTGAATTTGACATCAACCTCCGTA<br>CTTCCTGGAATAGCGCGTGTGCTTAAGATTATCAAATTGAAGGCCACCACTTATAGTTCATGTAACATCTC<br>TATGGAAATACATACTAAGGCTGTTTCTTCTAATTGCATAGCCAAACTCAAGCTTTAACAAGCAGGAAATT<br>CGATTGAAGTCAGAGATCAATGTTTGCAATTTTTATGGAGTTCAATCATTGGTATTGGTAGATGTGATAAA<br>AATTATTTATTTTGTTTTAGTTTATCTTTATTTTCTTTTATGTAAGCTTTGATTCTCTCCAAATGGGAAGC<br>TTCTGCATTTGTCTTCATACAAATATCCTTTTCTAGACATAAATTATAAATCTTTTTATTATATCAATGTA<br>TAAATATTGTATATCAAAAGGAAAAATTTATCTTCATACAAATATTCTTTTCTAAAGAAATTATAATTTTT<br>TTTATTATATCAATATATAAATATTATATATAAAAAAGAATGAACATTTTTACATATATCTACTTTAAAAG<br>AGAAAGAAAGAGAAAACAAATAATGGATAGATAAAATGAATAATATAATATGAGAATAAAAATGTTAAATA<br>AGTGTTCATTATTTTTTTATTAAAATATTACTTTTTACAAAAAATTATCCATGTATAATAATATAAATCTT<br>CATATAAATCCTATTTACTATAGATAAAATAATAATTATTTTTTATATTTTCATCCACTAAATATATTTGC<br>TTTTTTTTATGCCTTTTTTATAGAATATTAAGAAAATGTGTAAAAAACATTATATGAGAATATATGTAACA<br>TGTCTATTTTCAATTGACGCGTATATGCTGTTGTTTAGTTTATATGTTGTTGTCAATTCTTTAATTTTATT<br>TGGCATTAAGCTATCAAGGAATAGTAAATAAAATAATTGAAAACATATTTCTAATTTTTAAAATATAGTAA<br>ATAGATCAGTTTAATGTTTACTAATTATATAGTTGACTCTTTTATTTATTTAAAATTTATGTTCTATTTTC<br>GGTTTACGCATAATTTAATATATATATATATATATATATATATATATATATATATTGTTGGCGAAATATAC<br>AATCAAAAATAACAAAGGAGTGCCAAGATCGTCTATATATATATTGTTGGCGAAATATACAATCAAAAATA<br>ACAAAGGAGTGCCAAGATCGTCAAAATGTATCCCACGAGAAATTTTCAAAAGTATTAAATCTATCAATTAT<br>TGACCATTTAAACAAAACAATAGAAAACATCAATGAATATATAAGATCAATGGGTTAAGGAATAAATGAAG<br>AAGAGAAAAACAACTATAAGCAACAATTGAATTATGAAGCATATGACATCTTGTTAAACTTATGTAATCTC<br>TAACATATTAATTTATTTATAACTTACAGTTGTTCATTCATAAACTATTAATACTCAACAGTTATATAATG<br>TTAATATTTTTTATTAATCTATTATCCAGAATAATGAGGCAAAATAACATTAAAAACTAATAAAGAATCAA<br>CATAATTGTTAATTTAATAATTAATTAAATTAATAATTAAGTGTAACTGTAATCTCATAAAATTTGAATTT<br>GAATTTATTTTTTAAATTAGTTTTAATCAATTAAGATAATATCAAAATTGATAAAAAAAATTTATAAATAAA<br>AAAATAAAAAACAAATTCAATAGAAACCATAAAATTACATTGAAATCAGGAAAATAATAAAAGTTGAAGTT<br>AGAATATCCGCCATAATAGGATTGGTTATTTATAATAATAAAAAAATCAGTAACCACTGATCAAAGCTTTA<br>TAAGAATTCATATATATAATAAAAGGCAATAAATAATAAATAAAATCAATAAAAAAAACATAAAATAATAT<br>TAAAAGTACGAAAATAATAAAATTTTTAAAAACAAAAGATTCATCATAATCTTAATTATTAGATAATTAGT<br>GTCACAAATTTATTTAAAAAAACTTAAAAATAAAATTTATTTTAAAAAAATTAAAAATTATATTTTTAAAA<br>TGAAGTTGACGAATCGTTTATATGTTTGTTAAAAAATATTATCTTTTAATTAATTATAAAAAATATAATTA<br>AATTTCAAAAAAATGTTTTGATTTTTTTATTTTCTTTAGATTACTGAAACAAATTATTTTTAAAAAAAAAT<br>TAATTTTTGTAATTTTTAAAAATAAATAAAAAAGAATAAAAAATTTTATTTAAATTTGATGAATTTTCTTA<br>CGCATCCTCAAATAAACTAAGGAAATCAAAATTACATAATTTTTTTTGAAAAACTTTGATAAATTTTAGTT<br>TTTGGAAAATTATTTTAAAGTTTTTAAAAACTTTAGAAAAAATTAAAGGTTAAAATAAAAATTTAAAATAT<br>TTTTGTGCTTCTTGAAGAGGTAGTTTTATTTTCTTAAAATAAAGTATTCATTTATTTTAAATAATTTATTT<br>CAAAATTTAATGATAAAATATTTTTTATTTGTAAAATTTTTAAGAAATATTTTTTATATTTTTTAAAAGAA<br>CTAATTTTAAAAAATATATTACTTTTACACAAGGTTCAAAAATAACTATTTGATAAGTTGTTATATTCAAA<br>AAGTTAAAAATAAATTTATTATTAGTTTGACTTGACCAATCTATAAATTTAATATTTAAAAAAATATTATT<br>ATTAATTAAAAAAATAAGAATAATTTTAAAAAAAATTACTTAAAACTTAAATTAAGAGATGACTATCCAAT<br>AAATTTTTATAAATGAATTAATATTAATGATGAATCAATTATTTATTATTTATTTTATAAATTAAAAATGA<br>TTTTAAAATATTTCAAAGAAATATATTGTTTTGAAATTTGAAAAGATGGATTTTAATTTTTATATGAGATA<br>AAATTTTTAATATTTTTAAATTTTAAAAATTATCTATTTTAAAATTTTGATATAAATTATTTTTTGAATTT<br>TTTTTAAAAATTATTTAGAAAATATTAAACTTAAATTTAAAGTAAAGATTATCTTTTTTATTTTGTAGAAT<br>TTTAAAAAAATTAAAAAATTAAATTTAAGTTTAAAAGATAAATGATTTATTTACTTTGAAACTTTTACAAA<br>AAGATACCATCACCTAATTATATTTTTTTATATAATTTAATAACGTTTAAATTTAAAATTTTATATAAAAT<br>TATTTTAAACCTCACTTCTAATCATTAAATCAATCTGAATAATATATCATTTAATTATATTTAAAGTTCTA<br>ATTTTTATTTATTTTCTCAGTTTAATTTTGTTAATAAATAAAAAAAGTAATATTTTAAAATAAATTAAATA<br>TTTTATATTTTAATTATATTTATTTATTTATTTTATTACCATAAAAAATTTAAAAAATTATTATGATTCAT<br>CGCATTTTCATGAATACACCCATTCGATATCTTTTTCCATACTAGTTAAGTATCAAATTCATGCAAAAGGA<br>TACAACAAACAAAATATTTGTATTTGTTTATTTATTTTTATCTTGCTTAATTTTTATATTTTTTTAATTAT<br>TATGGATATTTTATATATTTTTATTATTACGATTGAAAATTTTACGTAGATATATATTATTTTTAAATTAT<br>TAAATAAACAAATTATAAATATTATTGTTTGTATGTTTAATGTGTATAAAATTTTATCTATTATAAATGTA<br>TTTTCTAATTTCAAAAAATATTTAAAATATTTAATATCATTTTTATATAAACTATTTCAATCACAAATAAT<br>AATTCAATTTTAACTTTTAAGCTTAAAAAAATTGTTTAAATTTTTTAAAATAAATATAAAAAATAAAAACA<br>ATATTTAAAGACAAAATAATATATATACGAATTTAAAGACAAAATTTAGATACCCAAAACATTAGTTTTTT<br>TTATAATTTTATTCAATTTCACTTCATCTCCCTTATAAATAATTTAAAGTCAGTTACTAACCATTCGTTCA<br>ATCGAACTTGTTAAATTAATAATCGGTTAAAAATTAGTAATTTTGATTGATCTTTCAAAATCCTTATATTT<br>GGATTACCTTATAATTTTGTTAAGTAGGCAACTACCTTGTAGCTATGTTAGTTAGATAATGGTATATTTGT<br>TTATCCCCACTTAATTTATAAATTAACAAAAATCGTTAATTCTCACTAATTAATTAAATATGATATGAAAA<br>ATATAATTAAAATAGAAACACAGAATCGTTATAAAAGAAGGAAATGGAGTTCAATTGATATATTGTAAGTT<br>TAGTGATGATTTGAGGATTAGTTACATGTAGAGATAAAATAAAAAAAGAACTAATTAAAAAACAATCCATA<br>ATTACAATTCTTAAAAAAAAAATTATAATGTTATCAAAGTTAACAATAGGTTATAACTCACACTATGAAAG<br>AAGAATTTATAAAAGAATTTCTGCCCTCCAAAATCATATAAGGCATATCTAAACAAAGATAAGATAATAAA<br>ATAAAGATAAGAACGGAGTGATGTGTTATCAAAATAATGATAGATTTGTGACGTGTAACAAAGTATTTCTT<br>TTGTAAATGTAAATTAGAGTTTTCCTCTCATTGACCAAACTTGAATTTAGATAAATTATCATTATTGTAAA<br>AATTATCATTCTTATTGTATATGTGAATTACTATTTCTACATAAATTTAAAAATTTAGGTAAAATTATAAT<br>TAAATAATCATAAATTATGTTATCATTAAAACTAATTTTTCACAAATAAATTAACAATATTGATTTAATAA

	TTATGCAACAAATCTGTGTGTACCATTCTTTATCATCACAAATTTTTGAAATTATAAAATTTTATAATACA<br>ATTATCACTTATTTATATCCTATCCAAAAATAAATTTAAAATATTATTATAAAAATATAAGATTTTAAAAT<br>AAATTAAATAATATTAATATTAGATATAAAAAAATTTAAATTTATATATAACATTCTACCGTCCAATATTT<br>TTAATATTATAAAATTTTATGTTATAATCCTATGTCATCTTAAACTCATATACACATAAAGAATTAAATAA<br>TAATAATAATATTTTTTATTTAATTTATTAAAATTTATACTTTATTGAATTTTGTTTTCATCGAAAATTAA<br>ACCTTTCCTAAAATTTTTTAAATCTTTTTTTTTTAATTTTCTTTTCAAGTAAAATAGTCAAAACTATTTTT<br>TTCAAAATTTAATTTTAATTTTTTAAAAAAAATTTCTTTCAAATTTTAAAATTATTATTTTCTAAAACAAA<br>TTATATTTCTAAATTTTAATTTTAAACAAAATTTTTTCTAGGTTTTTTCAAATAATTTCTAAACAAAATCA<br>AATTAATAGAACTATGTTGAATCTTAAATTTTCTATCAAATCAGAAAATATGTAAGAATGAAATAAAAATA<br>AAAACAAAAATTTCAGCACTATTTATATATATATATATTATATATAAATAAATATATTATTTAAATATTTA<br>AGCGTTATATTAAAATATTAAATATTTTTTTTAAACTACTCATTTAACATTAGATACCGTTCTTCCTAAGA<br>TGATGCATCTATTCTTCTTAACAAACTTTCAACATGATTTAAGTTGCAATAAAAGAAAATACTTTTTTGTA<br>TTTTTAAATAATTTTAAAAGAAGATCAGCATACACTTTTGCACAAAATTATTCAATAAATCTTTATATTTT<br>AAAAAATATTAAATATAAATGTACAATTTAATTAATTTTACTTGTTTATCATGCAAACTTAAAATATATTT<br>TTTTAATTTTTTTAAAATAAGAATATTTTTAAATTTTTTTAATGAAAGTTAAAAAAATATAAAATGTACAC<br>TAATAATATATAAATGGAAATAATTTATAATAAAAAAATAAAAATAATTATAACAAAATATATCTATCTTT<br>AAAAGGTTTAAAATCGAAACTTAAAAATAACTTAGGAGCATCCTTTAAAGATAAAATTATATGTTTTTTTA<br>TATTTGTTTTTATGCTATCTTTAAATTTATGAATGATATTATATAAAGGGATGAGAGGTTTATTCTAATGA<br>AATTTATAAATTAAATTTTAAATTTTAAACTTTTTTTATATGAGACTTCTTTATTACTTTGTTACATTGTT<br>TTCAATTTTTATTTTTAACAATATAGATAAAATTAAATATTGACTGTTGATTATTTATAATAACTAAAAAA<br>TAAAAAATTAATAATTTTTTTAATAAAAATTCATATCTAATATAACGTATAAAGATTGAAAAAAAATTCTT<br>TTCCAAATTTAAACTTAATACCTACCGAGTAGTGTGAACCTTAAAATGAGTAAAGTTGTAATTTTTTTTTT<br>CAAAAAATTTAATTTATTAATTATATTTTAAAAAAATAAAATTAATAATTATATAATAAATAATACTAATT<br>TAATAGGAGTAACTAATTTTTTTTTTTCTAAAAAAAGATGTCTCATTTTATTATGATTTTATTCTTTTAAAT<br>ATTACTTATGTTTGTTTTCACGGGTTTAATATTTGTTCCATAGCCATGCGCCTCTTAGATTCTGTTTTTTT<br>TTTTTTTTTTTCATTTTTACTTGATAACTAAAGTTTACAAATTATTAGTATTTTTAGAATTAAAAAGGGAG<br>GAGATTCGAATTTAAATCTTATGTAAACTGTATTAATCTTTAATTTTTATTATAGAACAAATTTTATTAAT<br>CTTACGAATTACTGTTTAATATTACAAAATAACAGCAAGAATCAAACATTGTCTTTTAACATTATATTACT<br>GAATAAATAAATAAATATGTAAAAATTTAGTATCTCATTTCAATAGACGAAAAAGAAAAGTAAAAATTTTA<br>CTAAGAGTTACCACACTTTCATTCTATGGGTGCAATTTTATTTTACAAGTGACAGTTTTATAATTTCTAAG<br>CTTTCCCAAATTTTGAAAAAGAAAAAGAAAAATCAACATCGTGGTGTTCATCATCCTGTGAGACTGAGGAT<br>GTTTGGATGTGAATGTGCACATGAAATTAATAAACATTGATACAAAGTCCTACTCTTGGAAGCATTAAAAA<br>ACATGGATAGGATGAAAATGAATGCACCCGAATCCAAGTACACCATATAATAACAAAACGCAGTGGAATTT<br>CTTAATGATTTTTTTTTATTTGTTTAGTTTGCTTTTTGTTGATGCTAACCTTGCAGAGAACAAACGTAGAA<br>GTGTCAAAAATGATTTAATTTTATAAATAGGTTTGGTGTATATTTCTATAAATTCTTTTGTTGTCTTACAA<br>AGTATGTATTAAATTAAAAAATTATGTATTTATTATTTTAAAAATATGAAAAATATTTAATACTTTCTCTA<br>TTTTAATAAATAAAAATGATTTCAAAACTTAAGAAGAAGATATTTACAATAGAAGAACATTATCCACATA<br>TTTTTAAATATATTTTTAATCTTTTTCATAATTGATGATTAGTTTTGTTTAAAAAGTTAAAATAATTTTTA<br>AAAGTTAATTAACTAATTAAATTTATATTTAATTTTTTATCAAATTTATTTATTTATAAAAATTTATTAAA<br>AATATAAAGAATAGTATGTTATTAATATTTTTTTATTATATTGCGAATTCCAAACTTATCGTTTACATTGG<br>TGCCACTTAACATTTCGTTTGAAATAATTTTTAATTTTAAAAATTAAAATTAACTTTTGTCTTTAATATTT<br>AATTTAAATTTTTACAAAAAAAAATTAAATTATTTTATTATAGTTTTATATAAAGTATAAATAAAAATAT<br>TAATTAAAAAAATTAATATTAAATAATATAAATTTTAAATTTTAAAATATTAAATTAAATATTATTATTTT<br>TATTGCTATGATAATAATAATAATAAAAATAAAAAAAATATTTATAAAAAAATAATAGTAATATGATAGTT<br>ATAATATAAAAATATTATTTAATTATATAAATTTATATTTTATATATTATTTGATTATTAATATTTAAAAT<br>ATAAAATTTTATGTATGTTTATTATAATATTTTTATATATTTTATAATATTAAAAATAATTATATATAATATA<br>AAATTATATTATAATATTATAAATTTAAAATAATTTATATTACTTATTTTTTAATATATAAAAACAGATAA<br>ATAAAAAATAATTTTTTTAAATAATAAAAAATTAAATTATTTTATTTTTTATTATGATAATTATAATAATGA<br>TGATTATAATACAAATGATATTATTTATAATTTTGTATTTTATATATTATTTTATAATACATTTATAAAAT<br>ATATAATTTATATATTATGTAATATTTATTTTATATAAAATAATATATAGTATATTACAAAAATTTACATA<br>ACATAAAATTATATTATTATATTATAAATTAAATATAATTTTATATATTAATTAACATAATTCATTGATAA<br>ATTTTTAAAAGTAATAAATAATTCATTTTTTAAAATAAAAAATTCTAAAAACTTAAAAATATTATTAATTA<br>ATATTATAAAAATATTGATAATGATAAAAATAAAAATAGTATTTATAAAATAGTAATAACGATATAGTGAT<br>TATAATATAAAAATTTATGTAATTATACAAATATTTATTTTATATATTATTTTAAAATAAATAATTTTATATT<br>ATTTGATATTTATTTTATATAAAAATAATATATTATAATATTATATTATAAATAATTATATAATATCAATT<br>TAATATAATTTTATATTAAATATTAGTTTTTTATTATATAAAAATTAAATTATTTTAAAATAATAAAAATT<br>ATTATTATTATTTATTATTATAAAATGATGGTTAGTAATAATAAGATTGGATAAATTTAAAAGTAAATATA<br>TTTTAAAATTAAAAATAGTATTTAATAAATTTTTATATTTTATGATTTAAATTAAATTATTTTAAAATAT<br>GTTAGAAATTATTTTAAATTTATTTACCAAATGTTTAATTTAAAAATTAAAAATTAAAAACTTACGATCAT<br>TCAGGATTTACATAACTAATATTAAATATTTACATATTACAATAGAAATGTCTTACGATTTATATAAAAAA<br>ATTATTTTATATATGGAAATTTACACTTTAAAAAATAAAAACCGGAACAACGCTTCTACTTTAATCTTTAT<br>AAAATTAACAAAAAATTATCCCATCTAAAAAATTGTCTTTGTCCTCGGAATTTTACCTTTTCTCAAAGTCA<br>ACTTTGCTTGTAGTTAGGGATTAGAAACTTGTATGTAAGGTAAAAAGCATCTTAGAAATTTCCAAATTAAA<br>GCTGCAAATGAAAAAAAAAAAAAAAAGGTTAACGTTGGTATCAAATTATTGATAATTAATATCAAAATTGTG<br>GGTTAACATATAAAAAATAAATCAAAATTAAATTACTAATTATAAAAGAAAGGTGCATTGTATTATTGAAA<br>ATTTTAATTAACATTAATATAGTTATTTAAATTTAAAATTTACAATAAATTGTATAATTTAAAATTTAAAT<br>TAATCTAAACGATAAGTTACATTTATCTAGATCTTATATTTTTTACTGTTGCATATATGCAAAATACAATA<br>AGATAACATATTCCCTTTGAACTATAAAAATCTGGCTCATAAAAATTAATTACATAATTAAGTTTTAAAAA<br>ATGACTGTTTAGTTTTGTAAATATAAAGATGTCCATGCTACAATCTTGCATTTTGTACTTCTAAACTAAAA<br>ATCTTGAAAATAATCTCCAATCCAAATTGAGCCTAATTGAGACAAGCCATTCCTAAACTGGTCAAGTTCTT<br>TTAATTAAAACTCAGTTTCACTGCTCAAGCAAGTTAAAAAAAAACTGGATTTCTCAATTTATTACATTTAC<br>ATTAAAAATTAAAATAAAGAAAAATGATTCAAATTTAAAAAACGTTAACAAAAGAAATTATAATTTCCCAA<br>TTTTTCATAAATAAATTAACAAAATAATAATTTATTATGTATAAACAAATAAATCACTTAAATTTATTTTA<br>TTATGTTATAAATTATGAAGATATTTTTGGTGACACAACAAATTATGTAACTTTATGTCCCCGTTATTTTA<br>GAATTTTATTAATGATTTTAAATATCTTATCATTATATGTTGATAAGCAGTTTTAATTTTTTTAATATTAT<br>AAATATATTTGATAAAGTTAAAAGTTTTATGAGAATATGTTAAATATGTGTAATTAATTTTTTTATCTTCA

	TAATAATATTATATTTTCATCTTTTTCATAAAAATATTAGCAAACAATATAGTTAAAAAATTAACAATTTT
	TTTTTTAAAAAATCTGGGAATGTTTTTAAACATTCCAACCAACTATAGAAAATTAATATTTTAAATTAAG
	ATTGAAGTATAAAAGAATCTTCCTACCAAACATCTTCCTACCAATCATCTTTCATAAAAGTGGTATATCAT
	ATATTTTAAAATTTTATCATTAACTAAAACCTAGTATATTATATGCGTTAATTTTCAACACAATATTAATA
	TAATTTTTTTACACTATTAACTAAACCAAAACTAAGATAAGTATGATTTTTGGAATAATTATTTTAAAAAT
	TATTTTTTAATAATATATTTTTTTATTGGATGATAATCTAAATTTTCTTTTTTACCAATATCATTGGATTA
	CAATTAAATTTATTATTTTTTTATTGGATGATCATCAACTTAAGAATTTACTTAAAAAATATTTCATTTGA
	TAACTTTTTGAAAGATATCAATTTAGTAAATTTAACCGTTAAATAATAAATTTCTATTATATAGATTAAGT
	TTACAAATTTTTATAAAAAATAAAAAATAATTTGATAATCTATTAAATAAATTTAATTTAATATATAATAT
	ATTTTAAAATTAAATTAAATATCAATCATAAAATTATATATATAAATTTAATATGTTTGATTAACATATTA
	ACACCATATTATTCAATGATGAAACACATTAAATTTGAATTTTATAAGAAAATCCAGTGGATTATTTTTAT
	TTTTCCAAAAATATTCCTAATTTTATTCTCGTCCAAATATATTAATATAATTTTATATTTTATAATTAATC
	TATAACTATATAGAGCCGTAATTTCATTATTTTAAAACAGATTTTTAAGGTTAGGATTGTGGTTAAGGGAT
	TTTAATTTTTTAATAAATTTTTTAATATATATATATATATAGTGTCATCAAATGTTTAAAATTAATAAATT
	AATTAATATTATTAGCATGTTTGTTAAATCAGCTCATTCAAATATCATTTTTTTCATTTATTTTATACATT
	TAAACAATGGAATAAAAAGACCGCACCTTTATAAGAAAAATATTAGTAATTAATGAGAGTTGGATTAGTGA
	AAAATAGTTTTTTTAACCATAAAACTCATTAATGACATTTGGTTCTAAAATTTTGTACAAATTATTTAAAT
	TTTGTATAATTAAACCAATCCTATTAAATTACAAATTATTCATTTATAATTCTTTTAAAATCTTAATATTT
	AATTTCTGATCATATTCCGAACAAATTCAACTATCTACTAATGAAAACAAGAGAAAACAAACTAATATTAG
	TGATTCCAACGTCCGATAACTATTAGTAAAATTAATTAATAACCTAATGTAACGCTGTTATTTATTTATTC
	CCAGTGCAAGTTTGTGAATTTTTCTGGTTTTCTGGTGTTTGCTTCCGCTGCCATTTATGGTTATGATAGTA
	TTAATTGTCACATAATTGTTTCTTGTGTTATACATAATTTTGTATGGTCAAATGCAAAGTAACTAGGGCCA
	GATTAATGTAACTTGATGACTCATAATACGGAATTTCATATAAGATAAATTAGACTAAACAAGGAAAATAA
	TTTTTTCATCCTTTTATTTTTTATTAATTTTTTTATTTTTATTCATTTCATTCAAGCATACCATAAAAATC
	GATTAGATTAGGATATAAATAGTTGTGTCACTTGCTGGATTTATGTTCATTCTTTATTTGGATGATATAAA
	AATAATGAAGAAAATAAAATAAAATAAAAATAAGATAAAAAATAAATTATTTAAATAAATAAAAATATAAA
	AAAATTATTTAAATGAATAAAAATAAAAACAAAAAAATATTAATAGAATAAATTTTTATTTATATAAATTA
	AAAAATAATTTATAATTATATTATTTAAAAAAATTTAAAAAAAAATTCTTTCCTTCATTTTAATTAGCCCA
	TAATATAAAGATTAAAAATATATATGGTTCTTGAAAATTAATTCTTTCTATTCATATAAAATAAATTAATC
	TAAATAAAAAAAATTCATTCTTTTCTTTTTCTCCTATTTTCTCCTTCTCATTTCTTCCATCCAAACTTACC
	AAACTCTCTTATCTCTTTTATTATGATAAAAAAATAAGTTTTATTTTATTTATATATATATATATATATAT
	AAAAGGAAAAAATAAATTTTGAATATAAATTCTTATATTAATTACTTAATGTAAATATATTTTTAATTTAT
	TGTATACATAAAAATATTTAAACACAATAAAAATTATAATTTCGTTTAGTCACATAAACAATTATTTTGAT
	AAATTTCTCAAAATAGTTGTTTGTGTATTTTTTTAATATATAATTATAATTATTTAAAGTTATTTATAATA
	TGTAATTATATATAATAGAATATTTATGATATATAATATAATATTTTATGAATTATAATTATTTATAATAT
	ATAATATAGTATTATAATATATTAAAATATTAATTATTTATACTTTTCATTAGTCATAAAACAATATACAA
	AATAAAAATGGTGTTATGCCTTAATTCTTAAACACTTGACGTGCGCAGTTCTACTTATCTTGTTACTCGAA
	GTGCGTCAAACGTATCTATTATGAACCGAAAAAATTACAGAATAACGTTGACTATGTATATATAATTTTAT
	GATGAAAATTAATTATTTTTGTCATTCATAGTAATAACTTTCTTTCCTTCAATATACCTCTTACATATATG
	TTTCAAGTTAAAATGATTTGCTTCATGAACATTATTTCATACATATATATCAAATTTGATTAAGTTGATTT
	TCCCAAATCGCCGTTCATTTGTTTTTTTAATCTCCTGAATACCCAGAATCATAAACCATGGTTAATAGACG
	CGGCAAAAGTTGCATTGGCGTGTCTCTACTGTTGGTGATGGTTATTGCCATCGTGATTGTGGCATTGTTTC
	TAACAATATTGAAGCCGAGGGAACCTGTGATCAGTGTCCACCCTGTTGGTCTCGAAGGCTTTAAACTTTCT
	CTTACTTTTTCCTCAAATGTGACGCTAGACATGTTGATCACCGTAGGGAATCCAAACTATGCAAGCTTTGA
	ATACAAAAATTCCATCACTTATGTCAGTTTTCGTGATACAGTTGTAGCTGATGTTCCAATCCAGGGAGAGT
	TTGTTCCAGCGCGTGGCGCCATTAATGTGACCACTTCCGCAGATTTGATGGTAAGAAAGTTAATACTTGAT
	CCCAAATTTTGGTCAGATATTTTAGGTGGCCATTTGAATTTGACATCAACAGCTACACTGCCTGGGGTAGC
	GCATGTGCTTAACTTTATCAAATTGAAGGCCACGGCTTATAGCTCATGTACCATCTCTTTGGAAATACGTA
	CTAAGAATATTTCTTCTAATTGCATATCCAAAATCAAGCTTTGA
	ATGGCTACTAGAAGCGTCAAAATTTGCTTGTCTGTGTCTCTACTTTTCTTGATCATTGTTACCATTGTGAT
	TGTGACATTGTTTCTGACTCTCTTTAAACCCAAGGATCCTAACATCACTGTCCACCCTCTTGGCCTTGAAA
	ACCTTCAATTTTCCCTTTTACCCAATTTGACACTAAATGTGTCTCTAGGTATGTTGATCACCATAGGGAAT
	CCTAACTATGGAAGCTTCGAGTACAAAAACTCCACTGGTTATATCAAATTTCATGACACTGTTGTAGCCCA
	AGTTCCAATGGAGGCAGAGTTAGTCCCAGCGCGTAGCACCATTAATGTGAACACTACAGCAGATTTTATGG
	TTGGAAAGATGATAAATGATCCTAATTTTTGGACTGATGTTTTAACTGGGACTTTGAATATGACATCAACA
	TCTACACTTCCTGGGGAAGCGCGTATGCTCAAGATTATCAAATTCAAGGCCACGGCTTATAGCTCATGTGA
CrLEA2-44	CATCTCTTTTAATATAACCTCCAAGCATGTTGATACCAAATGCATATCCAAATTCAAGCTTTGA
	ATGACAAAGGACTGCGGCCACCATGATGAGGAGCGGCAACAACTCCTCCGCCGCATATTCGCCGCCATCCT
	TGGTTTCATTATCCTCATACTCCTCGTAATCTTCCTAATCTGGATCATCCTCCGACCCACCAAACCCCGTT
	TCATTCTTGAAGACGCCACCGTCTACGCCTTCAACCTCTCCTCCACCGGCGACATACCTTCACCCACCGCC
	CCCACACCCAACACTCTCACTCTCACAATGCAAGTTACTCTTACCGCTCTCAATCCCAATTCTCGCATCGG
	CATTTATTACCACAACCTCCACGTCTACGCCTCCTACCGCGGCCAGCAAGTTTCCCTCGCCACCGAGCTGC
	CACCGACCTACCAGGGCCACCGTGACACCACCGTGTGGTCGCCGTTCCTGTACGGAAACGCCGTGCCGGTT
	TCCCCCTACGTGTTGGAGGTGCTGGAGCAGGACAAAACCTCAGGGGGAGTGTTGGTCAACGTCAAGGTCAA
	CGGTAGAGTAAAATGGAAGGTGGGGACTTGGGTTTCTGGAAGGTACCATATCTATGCCAACTGCCCCGCGT
	ATATTAGGCTCGCCGGAGATCGGGATACCGGCATCGGAGTTGCGGCGCCGGCGGTTAAGTTTCAGCTTTTC
CrLEA2-45	CAAAATTGCGTTGTTGATGTTTAG
	ATGACTGACAGAGTCTACCCTTCTGCCAAGCCCGCCGCCAACGGAAACGGCGCCGCCGCTAATCCATCTTT
	CCCGGCGACAAAGGCTCAACTCTATGGCGCCACCCGCCCTACCTACCGTCCCCAACCTCACCACCGCCGCC
	GTAGCCGACGCCGATGCTGCTGCACGTTCTGCTTCTGGCTCATCCTCATCCTCCTAATCCTCCTCTTCCTC
	CTCGGCGTCGCCGGCACCGTCTTCTACCTCCTATACCGCCCCCACCGTCCTTCCTTCACCGTCACATCGCT
	GAAACTGTCGTACCTGAACCTCACCACGTCCTCCTCCTCCCTCAGCTCCAGATTCGACCTCAACGTGAGCG
	CCACCAACCCTAACAAGAAGATCCTCTTCGCCTACGACATCACCTCCATCTCCATTCTGTCCGGCGATGTG
	GACGTCGGCGACGGCACCATTCCGGCGTTCCAGCACGGAAAGAAGAACACGACGCTGCTAAAGGCTTCAAT
	CTCAAGCAGTGGACAGGCACTGACGAGTGACGATGCGTCGCGGTTGAAGGCAAACATGAAGAGCAAGAGTG
CrLEA2-46	GATTGCCATTGGAAGTGAAATTGGAGACAAAGGTGAAGGCAAAGATGGGAAACTTGAAGACTCCAAAGGTA

	GGAATCAGAGTGTTTTGCGACGGAATAAGAGTCACTCTTCCCACCGGTAAGAAGCCTGCAACAGCGTCCAC
	CAAAAATGTTAAGTGCGACGTCAATGTTCGATTCAAGATCTGGAAGTGGACCGTTTGA
	ATGCTGTCTGCAAAATCTGAATCAGATATTACTAGCTTAGCTCCATCTTCACCTTCAAGATCTCCAAAGCG
	GCCTGTGTACTATGTGCAAAGTCCTTCAAGAGATTCACATGATGGAGACAAGTCATCTTCAATGCAGGCTA
	CTCCAATATCAAACAGTCCTATGGAGTCTCCTTCTCACCCTTCTTTTGGGCGTCATTCTAGAAACTCTTCT
	GCTAGCAGGTTTTCAGGGATTTTCAGGTCATCTTCTGGAAGGAAAGGTAGTAGGAAGAGGAATGATAAGGG
	GTGGCCTGAGTGTGATGTGATTCTAGAAGAAGGCTCTTATCATGAATTTCAGGACAAGGCCTTTACTAGGC
	GCTTCCAAGCCTTGATTGCTGTGTTTACTTTTGTGGTTGTCTTCACCGTGTTTTGCTTGATCATTTGGGGT
	GCTAGCAGGCCTTACAAAACTGAGGTTACTGTTAAGGTAACTAATTTTTCTTTTCTCAGTTTTGAAAGGAC
	TAGTAATTCTGTGTTTTATTTTAGACCATTAAATACTTGCTCTTAGTTACTCATACATCATGTCATACCTG
	CGGTGGGGAGTAAAAATTTTTGTACCTTTCTTGGTCAATCACATTTCTCATAAAGTCATCAAGATGATATG
	AATCATAATTCAGAATCTTGTTTTCATAATCTTTTATGAATCCATTTTAGATGCTGCTGCAATAGTAACAC
	GAGGCCTCTCCATGTCAGAAATGTATTTTCAACTTGAAAGAACTGTATAGTCTTTATAAATTGACAATAAT
	AAGTTTATCTATTAATGTATTTTATTTATTTTTCTGATCCTGTCAATGGGTTTGTGTTATTCCGGAAATTC
	AATTTTATTTCAAATTAGCACATGGGATATGATTACTGTAAATCTTCACCAAACAATATGATTACTATAAG
	TCCCTAAAAAGCTTGCGTAGCATTATTATATTATAATAGTGTTTCAAAGCGAATAATTTACTCATAATATC
	TTAATTCCTAAATTTGATTGGATACCCCCACTTTTTAATCTGACATTAGTAATAATAGCAGTATGCCACCG
	CTTCAGCATGTAAAACAAAAGACCCGAAGCAATGAATTTATGAAAGTATTGCTGCAATTCAAGGTATCTCA
	TAAAACTAATGCTAGGATCAAAGGAGTTCTGTAAAATTTGTGCTTACAACTGTCTCTAATTACTTGCTACT
	TACAAGCTACTACATTCACAAATTACATAATACTGCTTGACATTTGGATTGTTGCAGAGCTTCACAGTACA
	TAATCTCTATGTTGGAGAGGGTTTAGACTTCACAGGTGTCCCAACAAAAATGCTGACAGTGAATGGCACTT
	TACGCATGAGCATCTACAACCCTGCTACATTTTTTGGCATCCACGTGCATTCCACACCCATCAATCTTATC
	TTCTCAGACATCACCGTTGCAACTGGCAAGGTAACAATTCTTTCATCACTGCCCTTCTTCTGCTAAACAAC
	AAGCAATTCAATCAAGCATGACATGACCAAACCAACTTTCACTCTCACTCCAACAATGAGCTTCCCTAATC
	ACCAAGGATGTTACAAACTGTATAAACGTTCATTAACATTGAAAATATATAAAGTGACATCTACATTCAAG
	TCCTGTGTTGGCATGCATTTGTAAGTGACATGTTGTTCCTGTCCATGTAACTTTGTTGCAGCTAAAGAAAT
	ACTATCAACCCAGAAAAAGTCACCGTATTGTATCAGTGAATGTAGAAGGTAACAAGGTTCCCCTGTATGGG
	GCAGGATCCACCATAACAGTATCACAAACTGGTGTTGAAGTTCCACTCACATTAAACTTTGAAATCCGTTC
	CCGTGGAAATGTAGTAGGTAAGCTGGTAAGGTCAAAGCATCATAAGGAAATCACTTGTCCATTGGTCATCA
CrLEA2-47	ACTCTTCTGGATCAAAACCTATCAAGTTCAAAAGGAATGCATGCACATATGAATGA
	ATGATGTGTTGGATATACGTACCATCTTCGACGTTCATAAACTGCAATAGCAAAAATACAGAGAGCGTCCA
	AAGGAAAGCCATGAAAATCTCAAAAGCTGCTTACGACCTTCTCCTACGGAAACCTCATCAGCATCAAAACG
	ACATCGTTTATTCACGCCGTTGTGTACTGACGCTCACCTGCACGCTGGGTCTGGTGCTCCTCCTCACCACC
	ATCTACGCTTTCTGGCCGTCAGATCCTGATCTGAAGATCGTAGGGTTCAGGCTGAAGCGGGTCAAGCTGCA
	CCCGTTGCCTCCCGTAACCGTGGACATATCCATGCTCATCACGCTGAGAGTGCACAACACTGATATGTACT
	CCTTGGATTTTGGAGCCGTTAACGTGATCGTTGCCTACAGGGGGGTGAGGCTCGGGCACGTGACATCCAGC
	CATGGGCACGTGAGAGCCAGGGGATCTTCCTACGTGGATGCTGACGTGGAATTTTCTGGAATCAGTGTGCT
	TCCGCAGATGGTATGGTTGCTGGAGGACGTTGTTAAGGGTATCGTACCCTTTGATACTGTTAGCCAGGTTA
	GGGGCCAATTGGGGCTTCTCTTCTTTCACTTCCCTATGAAGGTAACTTAACTTACTTAATTACACTAATAC
	CATTTTATCAATAATCAAATTTCATTAATTAGCTAATACTTTGAATTTGCCGATTCTTCTCTGTCTTGTAA
	TATGATAACATCCCACAGTCAAGGCTATCGTGTGAGGTTGTGGTGAGTATACAAAACCAGACGATTGTTCG
	CCAACATTGTCACCGCGGGGTAAGCTTACACTGCTATTGAGTTTCCTAATTATGGAGTGCATGTGATGATG
	TGATACAATTTGTTGATAATTTAAACTTGTCATTTTTCAACCAATGTGCATGCTTATGTTAGAATGAATAT
CrLEA2-48	GTTTGTTATTTCACTTCAGAGATGA
	ATGTGTGAGAGCAAGAGTTTCTACAAATGGCTCATGCAATTCATATGCCTTTTAGGCCTCCTTGTTCTGTG
	CCTATGGCTGTCCTTACGACCCAAGAGCCCCTCCTACTCCATTGTATGCATCTCCATAGAACAAGCCTCGA
	ACTCGAATCAAAACGGCACCATCTCTTACCAACTTCAAATTGAAAACCCAAACAAGGAATCAAGCATTTAC
	CATGATGATATAATATTAAGTTTCTTATATGGGCAGATGGAGGATGAGGTGGGGGAGGATACCATAGGCTC
	ATTCCATCAAGAAACTGGTAACACCCGAGATAAAATTGGTACTGTCAACGCCAAACCTCGACCATTCAAAC
	CTCTCTTGAACGCCATCTCAAATGCAACCGCAGAGTTAACAGTTGCTTTGAAGACAAAATTTCGATACAAG
	ACATGGGGAATTAAGAGCAAGTCCCATGGTTTACACCTCAAAGGTATTCTACCAATTGATTCCAATGGGAA
	GCTTTCACGTAACAAGAAAAAGTACCCACTTAGTCCCAATTCCCAAAAAATAGGAAGGTACAAAATAAAGC
CrLEA2-49	ACTGA
	ATGAGGTGTTCCAAAGGGTTGAAAATCTGCTGTGCTGTCTCTGCCATTCTTCTAATTATATTGGTTGTGGT
	ACTTGTGATCTTGTTCTTCACGGTTTTCAAGCCCAAAGACCCCGATATTATCCTACAGTCAGTTAAGCTTG
	AAAGGATCGAAATTGGCTTCCCAAGTCCAGAAATAAATGTGTCATTAGGCCTAGTGGTCACGGTTGAAAAC
	CCCAATCATGGAAGCTTTACTTACCAAAACAGCACTGCTTATCTTAATTATCGAGGGAATATTGTGGCTGA
	GGCTCCTATACATGAGGACACAATTCCTGCTCGTGGAGATCATAACATAAGCACATCTTTGGATATTTTTG
	CAGATATCAGAAAATTTAAGGATTTACCGAGTGATTTTTTAAGGGGCGTTATAAATTTTACCTCAACAACC
	ACCTTACAAGGCAAAGTCAAAATATTGCACCTCTTCAAGTTCAAGGCCACTAGTTACTCCACCTGTGATCT
CrLEA2-50	CTCTTTACTCGTACATGACAAAAGCATCAATTCTACCTGCAACACAAAAATCAAATTATAA
	ATGACCAAGCCACAGAGCCGCACCAACCTAGCTTCATGTTTCGTCGCCACAATCTTTTTGATTTTCCTTGT
	AATCGTCGTCTTCGTCGTTTACTTTACGGTTTTCAAGCCCCAGGATCCCAAAATCTCCGTCAGCGCCGTTC
	AGCTCCCTTCCTTTTCCGTCACTAACGGCACCGTCAATTTCACCTTTTCCCAATACGCTTCCGTTAGGAAC
	CCTAATCGAGGCACCTTCTCGCACTATGACAGCTCACTCCAGCTTCTCTACTATGGCAGACAGGTCGGGTT
	CATGTTTATTCCCGCCGGCAAGATCGACGCCGGCAGAACTCAGTACATGGCGGCCACGTTCACCGTTCAAG
	CATTCCCGTTGGGCTTTGGCCCCACCTTTATTAACGGGCCCATTAATGTGGGGCCCACGATGGAGATCGAG
	TCGAGAATATCGATGGCGGGACGAGTGAGAGTGTTGCACCTCTTCAGTCATCACGTTGAGGCCAAAGCTCT
CrLEA2-51	ATGCAGGGTCCCCATCGCCGTTCCCGATGGAACCGTCTTAGGTTTTCACTGCTAA
	ATGTCAGAAACAGTGACCGAGTCCCGGTCAAGTCCAGAACGAGGAGGTATATATACACACACAACATCAGA
	GAAATTAATCGCCATGTTCCCAATACGCAAAGAAGAAGAAAAAAGCAGCAAATGTGTGGTGTACGCTTTGG
	TTACCATTGCTACCCTATTGGGGGTTGTATGTTTCTCATCTATCTTTTTGGTGGTGCGAGATCCAAAAATT
	GAATTGATATCAGCTAGGTTGGTGGACAATATTAATCATACCGTTATTTCAGCATCATCTCCTTTTAATTT
	GAGCATGATTGGGCGTGTGAGCCTGTGGAACCCAAACTTTGGTCGCTTCTATTACGACAACACTACAGTGA
	GTGTGATGTATGGGGATTTTAGTATTGGTGCTTCCGAATTGGGAGGTGCGATTCTGGAGGCTAGAGAAATC
CrLEA2-52	AAAGAATTCAATTCCATCGTAAATATGAGGTTCAGTGAGGAATTGGTGAATCTTACCAATGATATCAGTTC

	ATGCATGCTCAAACTCAGAAGCCACTCGGATTTAAGTGGTATAGGGTACGTGCTCAAGTTTATAAAGAAGA<br>GGAAGACCATACAAATGGACTGCACTATGAATCTTGACTTAACCTCTTCTTCACTTCGCCTACTCCAATGC<br>TAA
CrLEA2-53	ATGGAAATGGCGACAAAACATTCTTCGTCAAGACATCACACAATTCACCAAGAGACAGCCATTTACAAACC<br>CATACACCAAGAACGAAGCAGCAAATGGTTCGTGTTCCTTTTTTCTGCCTTTGTCATCTTATGTGCAGTTC<br>TATTGGTCTTTGCATCCGTGTTGCGCGTGCGGAGTCCAGAGGTCAAACTAAGATCAGCTACCTTGAAACAA<br>ATCAATTATAGAGCTTCGCCAGCACCTTCCTTCAACGCCACTCTGATCACCTTTCTCAGAATTAGGAACCC<br>AAATTACGGTGCGTTCAGTTATGAGAATAGCACGGTGACTGTGCTGTATGCGGGTGAAAATATTGGTGATA<br>AGGGAATCGGCAGTGACAGAGTTAGTTACAGAGAAACCAAACAAATGAATGTTACCGTGAATGTGAGGTCC<br>TCTAAGCTACCCGTTACTGGGAATCTTTCGGGTGCCATTAATTCAGGAATGTTGAATCTCACCGGTTATGT<br>CAAATTTAGTGGCACGGTTCATCTTCTCAAGATAATCAACAGCAGAAAGACCATACAAATTGCTTGCACTA<br>TGAATCTCAACTTGACCTCCCACGCAATTAAGGGTATCCAATGCTAA
CrLEA2-54	ATGCCGTCCCATCATGACCATGGCCATGTGGCTGAGCATCATCATCATCATCATCGAAGCCCAATACCCAG<br>AAGACTAAATACTCCTCACAATGATAGCACCCATCCCTTAATCTGGCTAGCAGCCATTCTCTGCACAATCA<br>TAGCCATAGGTGTTGTAATTGCAGGCATAGTAGTTTTCATTGGTTATATGGTGATCCACCCAAGAATCCCC<br>ATCATAAGCATCACCAATGCTCATCTAGACCTTCTCAGAAACGACTATGCTGGTCTTCTACAAACCCAACT<br>TCGCATCATTGTGGTGGCACAGAATGGAAATGCTAAGGCTCATGCAACTTTTTCTGACATAAAGTTCAATG<br>TTAGCTACCAAGGGCAAGGCATAGCAGTGATGGTTGCTGACCCTTTTGATGTTCCAAAGAACAATTCAAAG<br>ACACTCCCTTATGTGGTTCAATCTGCTCCTATACCCTTAACCCCTGATCAGATGGAGGAAGTGGATGTTTC<br>TTGGAAACGAGATATCATTGGATTTGACATTAAGGGTAGCGCAAGAACACGGTGGAGAGTAGGACCTTTAG<br>GTTCTGTTAGATTCTGGTGCACTTTGGAGTGTCAACTCAAGTTTCACCCTTCCAATGGTAGTTACATTCCC<br>AAACGCTGCACCTCTGAATCAAAATGA
CrLEA2-55	ATGTCAACATCTGATAAACCAGAAGTAGTGGAAAGGGAAAGTAAGGATGGAAAACAAAAGGAAGATAATGA<br>TGAGAAGGGTGGATTTCTTGATTCAGTGAAGGGTTTCATTCAAGACATTGGTGAGAAGATTGAGGAAACTA<br>TAGGATTTGGGAAGCCAACTGCAGATGTTACAGGAATACACATTCCATCAATCAATCTTGAGAAGGTAGAA<br>CTTGTTGTTGATGTTCTCATAAAGAACCCGAATCCTGTGCCGATTCCCCTAATTGACATAAACTACTTGGT<br>TGAGAGTGATGGGAGGAAACTAGTTTCAGGATTGATACCGGATTCAGGTACAATCCATGCACATGGAGAGG<br>AGACAGTGAAAATTCCTCTTACTTTGGTTTATGATGACATAAAGAATACATATAATGATATTAAACCTGGA<br>AGCATAATTCCTTATAAGGTGAAGGTTGATCTCATTGTTGATGTTCCTGTCTTTGGAAGGCTCACTCTACC<br>TTTGGAGAAAACTGGAGAAATTCCTGTTCCATACAAGCCTGATGTTGATCTTGAGAAAATTCAGTTTGAGA<br>GGTTCTCTTTTGAAGAGACTGTTGCAATTCTTCATTTGAAGTTGGATAACAAGAATGACTTTGATCTTGGT<br>CTCAACTCGCTCGATTATGAGGTGTGGCTTGGTGATGTTAGCATTGGAGGTGCAGAACTTACCAAATCTGC<br>AAAGATTGTGAAAACTGGGATCACTGACATTGATGTTCCAATTACCTTCAGACCTAAGGATTTTGGTTCTG<br>CACTTTGGGACATGATTAGAGGAAGGGGTACAGGATACAGTATTAAAGGACATATTGATGTTGACACTCCT<br>TTTGGAGCAATGAAATTGCCCATTACCAAAGAAGGTGGTACTACCCGTCTCAAGAAGAAAAAGGAAGATGG<br>TGAAGATGATGAGGAGGTTTGTTAACTTCTATTATCTATGTTTCTTATTTACTCAATTCTTTTTTGCTTGT<br>ATACCAGTGTATTATACATTTGTATGTGGAATATTGTCCTTTAATCATAAGGGCATAGTACATTCTACCCT<br>TTGAAGTTCAACAAACTTCATCTGAAGTTTTATTTTTATTTTTACATTACTCTTACCTCCCTTATTCTTAC<br>AACTTCATAAAAAATTTGAGAAAGGTTCTATAATCTAGAAAGATATATAAGGTTATGTTGTTAGATAAATTA<br>AGCAAGATTTGTGTAGGGTAAAAAAAGCTATCAGATAAAATTTTATATCTCTTTAGGAGGTATGAATATAA<br>TTTGCCTTAATCATAAATGACAATAGCTAATCTTTCTTACTTATGTTTATGCGTATGTTTTGCTTGTTGAT<br>ACCGGAAAGTTCATTTCAAAAATAGATTTAACTGGCCTAGTTTTGACGGCATCAGTATATAAATCTGTCTA<br>GGATGTCATTTGCAACCCTGACAATAAGAATTGTATCAGTCCTTGACGTTTTACTTTTTAATTCACTCACT<br>ATTATCTTGAATTTGTGTCTCAGTAGAACTTTGATACCACCACCTGCTATCCTGTAATTTGTAGAAATTTG<br>ACTAAAACTGCATTCATTACTGTAGGAGCCTGATTGGAATTCAGTTTATGATTCTTGTTTGGCAGAATTTC<br>AATATCGACATAGTGGAGATGCTCAGAAACAAGTCAAATAATATTTTTCTGCTTAGTTGGAAATCCGAGAA<br>CCGATTTAATGAATAATTTGTTGACTGAATCTCATATTAAAAAAAAAAAAAAATCTTAAAATGAAAAGGGAT<br>GCTTCTCAAAACTCACTCTTGAAACAGTCTCCTTTAACTTGATTAGAGTAGTGTTTTTGGTATATCCTAAG<br>GGTATTCCTAGCTTAAGACTAATCTCTTCCTTGAGGCGTTTACCTTTTCCAACAAGTCTTTTTACTTTTTA<br>TGTTTAAAGTATTTTGAAGTATTTATTATCTTTTCTTTTAACTGACTAAGCAGGTCAATAATTTGTTATCT<br>ATATCTTCGCTTGTGCTATAAATTTAAGCCTTCAATCTTTTCTTTTGCAGGATTGAAGAAGAAATGGCAGC<br>CAGTTATAGTATCTGTTCTTTTATTCACAGCTTAG
CrLEA2-56	ATGCTTGCACTTCCACCACCCCCACCACCACCACCATCACTACCATCACCACCATCCCTAGCCATAAGGCA<br>AACCAAGCAAGTTTCTCCTGGCCAAATTGTCAAATCAAAACAGCCCATAAGACAAAATTCTCAAGAATTAG<br>AAGCACCAGATTATACTACAACAAAGTCAATTAGGCCACTACCAAGATTAAGGCAACCTCCATTTCAAAGG<br>ACCAACCCAATAATATGGTTTGGTGCAGTGTTGTGCCTCATTTTCAGCCTTGTGCTCATCTTCTTTGGAGT<br>TGCAACTTTGATCATCTTCCTTGCCATCAAACCAAGAAATCCAACTTTTGACATCCCAAATGCAAACCTCA<br>ATGGATTGTATTTTGACTCACCAGAATACTTCAATGGTGACTTCACTCTCCTTGTAAATATTACCAATCCC<br>AACAGGAAAATGGATGTGAGGTTTGAGTCTTTGGATGTTGATCTTTTCTTCTCAGATAGGATCATATCAAC<br>ACAGTCAATTCAGCCCTTTACTCAAAGGAGAAAGGAAAGCAGGTTGGAATCATTGCACTTCATATCCAGTT<br>TGGTGTTTTTGCCTGAGGATCTTGGTGTGAATCTTAAAAAGCAGGTGCAAGGTAACAGGGTTAACTACAAT<br>GTGAGGGGAACTTTTAAAATGAGAATAACTATGGGCTTCTTCCATTTATCTTATTGGCTGCATAGCAGATG<br>CCAGATAGAGATGACTAGTCCACCAACTGGGGTCTTGATAGCTAGGAACTGCATCACAAAGAGATGA
CrLEA2-57	ATGTCAGATCATCAAAGAATTCATCCTGTTCATGATGTGGAGGCACCACACAGACCCTTGGTGCCAAGTAA<br>CATTGGTAAATCAGATAAAGATGTTCAACCTCTTCCTCAACGCACCATTCCTGTGATGCATTCAAAGCCAC<br>CAAAGAAAAGAAACTGTTGCTGCAGGGTTCTATGTTGGACATTAAGCGTATTGGTGATTCTTATAATTGCT<br>CTTGCCATCACCATTGGGATACTATTCCTTGTGTTTAGGCCAAAGCTTCCCAAATACTCAGTGGACCAACT<br>GAGAATAAGCCAATTCAATGTTTCTGACAACAATAGCCTTTATGCTACCTTCAATGTGACAATCACTGCAA<br>GAAATCCAAACAAGAAAATTGGGATATACTATGAGGGTGGAAGCCACATAAGTGCTTGGTATATGGACACA<br>CAACTATGTGAAGGGTCTTTGCCAAAGTTCTACCAAGGTCACAGGAACATCACAGTGCTTAATCTGTTTTT<br>GACTGGTCAAGCACAGGATGCAACTGGCTTACAAAGTAGAATTCAGGAGCAGCTGCAGCAGACAAATAATA<br>TTCCCCTTAATCTTAAGGTTAATCTGCCTGTGAGGGTTAAGCTTGGTAAATTGAAGCTCTTCAAAGTCAAA<br>TTCAGAGTTAGGTGCAGGCTTGTGGTGGATAATATTGGTGCTAATAATGATATTAAAATTTCAAGTAGTAG<br>GTGTAAGTTCAGGCTTAGGCTATGA
CrLEA2-58	ATGTCAGATCATCAAAGAATTCATCCTGTTCATGATGTGGAGGCACCACACAGACCCTTGGTGCCAAGTAA<br>CATTGGTAAATCAGATAAAGATGTTCAACCTCTTCCTCAACGCACCATTCCTGTGATGCATTCAAAGCCAC

	CAAAGAAAAGAAACTGTTGCTGCAGGGTTCTATGTTGGACATTAAGCGTATTGGTGATTCTTATAATTGCT
	CTTGCCATCACCATTGGGATACTATTCCTTGTGTTTAGGCCAAAGCTTCCCAAATACTCAGTGGACCAACT
	GAGAATAAGCCAATTCAATGTTTCTGACAACAATAGCCTTTATGCTACCTTCAATGTGACAATCACTGCAA
	GAAATCCAAACAAGAAAATTGGGATATACTATGAGGGTGGAAGCCACATAAGTGCTTGGTATATGGACACA
	CAACTATGTGAAGGGTCTTTGCCAAAGTTCTACCAAGGTCACAGGAACATCACAGTGCTTAATCTGTTTTT
	GACTGGTCAAGCACAGGATGCAACTGGCTTACAAAGTAGAATTCAGGAGCAGCTGCAGCAGACAAATAATA
	TTCCCCTTAATCTTAAGGTTAATCTGCCTGTGAGGGTTAAGCTTGGTAAATTGAAGCTCTTCAAAGTCAAA
	TTCAGAGTTAGGTGCAGGCTTGTGGTGGATAATATTGGTGCTAATAATGATATTAAAATTTCAAGTAGTAG
	GTGTAAGTTCAGGCTTAGGCTATGA
	ATGACTGATCCAGTTCATCTGAAAAACTCGCCCCCACAATCCAACGACGCACAAACGTCGCCGCCTCATGA
	AGCTGCCGTCACCTCAAATTCCTCCTCTCTGGCGGCAACCTACGTCATCCGTATCCCCAAGGACCAAGTCT
	ACCGCGTTCCTCCGCCGGAAAGTGCTCTTCGCTACGCCAACTACAGCCGCCGGAAAAATCGTCGATCCGG
	TGCTGCTGTTGCCTCTGCTGTTTCATCTCCATCGTCTTCTCCCTCGTCGTTCTCCTCGCCATTGCCTCCGG
	CGTCTTCTACTTCGTTTTCCGACCAAAGGCGCCCGACTATGCAATCGAGCGCGTGGCCGTGAAGGGAATGA
	ACCTCACGTCGCCGTCGTTCACAGCGGCATTATCACCGGAGTTTGACGTCACTGTTCGGGCAGACAACGAT
	AATAGCAAGATCGGAATCTACTACGAGGATGGAAGTTCCCTTGAAATGTTCTACAATGACGCTAGGCTCTG
	TGATGGTGCATTGCCGGCGTTTTATCAGCCGTCAAACAACGTGACGGTGTTCGATACGGTTTTGAGGGGTA
	ACGGCATCGCACTCACGACATCGGACCAGACAGCATTGGTGAATGACGTTGCCAAACAGAGCGTGCCGTTA
	ACGTTGAAGCTGAGGGCACCGGTGAAAATTAAAGTGGGATCTGTTAAGACGTGGAAGATCACTGTTAAAAT
	TACCTGTGATGTGACTGTAGATCAGTTAACGATGAAGGCTAGGATTGTTAACAAGGACTGTAGATACCTGT
CrLEA2-59	TGGCTCTTTGGTGA
	ATGGCCGACCGAGTTCATCCCAAAAACTCGCCCCCACAATCCAACGACGCACAAACGTCGCCGCCTCATGA
	AGCTGCCGTCACCACAAAACCCTCGTCGCCGGCGGCAACCTACGTTATTCGTATCCCCAAGGACCAAGTCT
	ACCGCGTTCCTCCGCCGGAAAATGCTCATCACTACGCCAACTACACCCGCCGGAAATATCGTCGATCTCGG
	TGCTGCTGTTGCCTCTGCTGGTTCATCTCCATCGTCTTCTCCCTCGTCGTTCTCCTCGCCATTGCCGCCGG
	CGTCTTCTACTTCGTTTTCCGACCAAAGGCGCCCGACTATACAATCGAGCGCGTCGCCGTGAAGGGAATGA
	ACCTCACGTCGCCGTCGTTCACAGCGGCATTATCACCGGAGTTTGACGTCACTGTTCGGGCAGACAACGGC
	AATAGCAAGATCGGAATCTACTACGAGGATGGAAGTTCCGTTGAAATGTTCTACAACGACGCTAGGCTCTG
	TGACGGCTCATTACCGGCGTTTTATCAGCCGTCAAACAACGTTACGATGTTCGAGATGGTGTTGAGGGGTA
	ACGACATCGCACTCACGGCATCGGACCAGACGGCGCTGGTGAATGACGTTGCCAAACGGAGCGTGCCGTTA
	ACATTGAAGCTGAGGGCACCGGTGAAAATTAAAGTGGGATCTGTTAAGACGTGGAAGATCACTGTTAGTAT
	TGACTGTGATGTGACGGTAGATCAGTTAACGGCGAAGGCTAGGGTTGTTAACAAGGATTGTAGATACCGAT
CrLEA2-60	TGGCTCTTTGGTGA
	ATGGCCCGCTCTCTCTCACAACCCAACCGTCTTGGTTTCATCGTTGCCCAATCTATCTCCCTAATACCTGT
	TTATCGGTCAGTTCCATTTTTTGAAATATATTTTCAATTCATTTCACTTAAATACTCTTACTGCCTGTCTC
	TGTCTGCCCTCATAAAATTAGCAATTTAGCACTTTTGTAATATTTTGGTAAATTATCCAAGGGATTAGTTA
	TAATTTTATTGGCAATATGACGTTTGTGCCCTACATGGTTGTAATTTTAATGAAGAGTAAATAAATATTTC
	CTGAGTCCTTTTCTATATATATATCATTATTGTGGTACATAAACTAATTTCACCGTTTGCAACACTTGCTA
	TGATTATTATGATAGGCGAGGTTATTCAGCCCGCGTCAGATGTTTTAGGAAGAGTTGGATTGGGTATTATTG
	CACGCAGGAGTGGAATGGTGGGAAGGACAGAGGAGAAGCCTGTAACAAGAGATGGTTCGGAGGCCTACTCG
	GCTTGGGCCCCTGACCCAGTAACGGGTTATTATCGGCCCATCAACCACACCCCTGAAATTGACGCGGTGGA
CrLEA3-1	TCTTCGCCAGATGCTTCTCAACCACAAGCTTAGATCACCCCACTAG
	ATGGCTAATTTCCTTCTCAGGTAAGTCTAGCTCTATTCTTTTTGCTTTGTATGTGCCATTATTTTTGGTGA
	CAAACACTATATACACTGTTAGCATTTAATTCAAATCAATATTTCATCTTAATAACTCTTATTAATTTAAT
	TACTGATATGAAACATCAATTGTAATTGGGCATAAAATGTATCTAAACTTTCTCATTATTTTTTCTCCTTT
	ATTTAATTTTTTTTATACTACGAACTTATATATATATATATATATATATATATATATATATATGTTAAT
	TAACTATCAAGACAATGATGCAGGAAGCGATGTTATGGTGTTGTAGCGGAATGTTTAAGGTTGGAGATGGA
	AAGCACGATGGTGAAGGCGAAAGCAGCATCCCCAAATGGTAATTCCAATAAAGATGAAATCTTTTGGATGA
	GAGATCCTAAGAGTGGAAATTGGATTCCAGAGAATCATTTTGGCGAAGTTGATGCTGCCGAGCTGAGGAAC
CrLEA3-2	AAGTTTCTGCCAAGACACACAAAACTTCCCAATCAACTTAAGTAA
	ATGGCTCGCTCTTTCACTAACGCCAAGATTTTCTCTGCTGTCGTCCTTGATGGATTCCCTAGCGTTTTCAC
	CAGGTACCGTAATTTTGATTGATCTTTGTTGTTATGAATATTAAAAGGTAAATTAGGGAATGGCATGATTT
	ATAATATATGGTTTTTATACAGACGTGGGTACGCGGTAGCAACACAAAGCGCAACAAGGGGAGGAACGGCC
	TCTATAAGCGGCAAGGTGGCACCCAAGGCTGTGGAAGACAAAGTGGCCTCCAACAACAAGGTTTCGTGGGT
	GCCAGATCCTGTTACTGGTTACTACAAACCCGAGAACATCAACGAAATTGACGTTGCTGAATTGCGCCAGA
CrLEA3-3	CCCTTCTACGCAAAAAAATTCAACAACTGA
	ATGGCTCGCTCTTTCGCCAACGCCAAGGTTCTCTCTGCGCTCGTAGCCGACGGATTCTCCAACACTCTTAC
	CAGGTAAAAAACAGAGACGGGTTATCTATTTTTAAATTCAAACTTGCTTTCCCTTATGTGCTATGCGATTT
	GCGCAAGTTTTAATCCGAAGAGGATTCTTGTTGTAACGGTTTTTATTTATTAAATTGAAATTGACTCGGTA
	TGATTTTGTGTTGAGAGCAGACGTGGTTACGCGGCGGCGACACAAAGCGCAACAAGGGGAGGAGCTGCCTC
	CATTAGCGGCAAGATAGCCCCAAAATCAGGGGAAGACAAAGTGGCCGCTACTGAAAAGGTGTCGTGGGTCC
	CAGACCCCGTTACTGGGTACTACAAACCAGAGAACACCAAAGAAATTGATGTTGCTGAGCTGCGAGCAACG
CrLEA3-4	CTTCTCGGCAAAAAATTCAACCTCTAA
	ATGGTTAGAAGCGGCATCACCAAAGCAAACATGTTGCTTCTCCGGTAAAATTGAACTTAATTATTAACTCC
	CTTAATGATCAATTCAAAGTTGTTGTCACTTTAAATACTTCAATCGGTGCCCCCAAATGGATCCCATTTCT
	TTTCATTTTAGCAGTTTAATTGTTTGAATCAATATTTCTTAACCATGATTAATTTGTCCATTTACATGTT
	AATTATTGATTGGAGAGATTTTTTTATAGCCAAACCCCTTTTCAGATTTTTCATTGTGTTTGAAAACTTGA
	AGATACATAACAAACGCAATGTGAAGGTCATGCAGGGGAACTGAGAGAGCAAAGAGTGGACTGAGCAAGTT
	GTTACCTGCTGCTACAGAAAACTCTGGAGGAATTTCAAAGAGTAGTGGGAAGATGGAAAATTCAGAATTGT
	GGGTCCCACATCCACGCACAGGAATTTATTTCCCAAAAGGGCATGAATGGGTGATGGATGATGTTCCAGAG
	GATGCAGCTCGTTTAAGTCAGACTCACTGGTTCAGAAATATTGATGGTGTGGACAATCCCAAATACCAAAC
CrLEA3-5	TTGA
	ATGGCATGCTCTTTCGCACAAGCCAAACGCGTCCTCGCTTTCGCTCTTCATCGGTTCGTTCCTACTTCATG
	CAATTAAACGCCCAGTCTTGTTCCTTCTTGCATGTTCCTTTAACAATTTTATCAATATCTCATGTGCGATT
	TCACGTTCTTTTTTTTCTATAACATACATATTAAATAAAACTAACAATTTTGCACGTTCGTTAATTTTTAT
CrLEA3-6	ATTTTAAAATAAAATAGAGAATATTTTTTATTTCTCTGCTATCGCAATTATTATTGTTGTTAGTGTTGAAT

	TGGTGTGTATGGTTATGGATGTATTTATATAATGTTATGTATGCATGTGTATTGGCGGTGTTACAGGCGAG<br>GGTATGCAGTGATGTCTGATGCTTCGGTGAGAGGGGGATTGGATAGGATTGGGAGTGGGAGTAGGAGTAGG<br>AGTGGATCAACGGTGGTGGGGAAGGTTGAAGAGAAGAGTGTGTCCAAAAGTGGTTCGGAGGGTTCTTCAGC<br>TTGGGCCCCAGACCCAGTCACGGGTTACTACAGGCCCATCAATCACACCAATGAGATTGACCCGGTGGAGC<br>TCCGAGAGATGCTTCTGAATCACAGAGTCAGATCTTCATCCCCTTAA
CrLEA4-1	ATGGCGTCGAGGCAAGAATTCAAGGAAGACAGAGCGGAAGCAGCAGCAAAGCTAGCTGCGAAAGACATTGG<br>CCATGTGAACAGAACAAACGAGCGTGAGGAAAGTTACACACTGCATTCCGATATTCACTTTAAGGAAGCAC<br>GAGCGGAGGCAGCAGCGAAGCTTGCTGCTAAGGATCTTGAAGACGTTAACAGAGCAAGAGAGAGTGCATTT<br>CGTGAAAAACAACGTTATTGTGATGAGCAGAAACCCGGTGTGATAGGGTCCATGTTTAGAGCCGCGAAAGA<br>AGCTGTGGTTGGCAAGCCTCACGAGGGTGTGGAAGTGAGGGATAGAGGTACCTCACCTGGGGTTTATGATT<br>ACACCACCCAGAAGACCACCGAAACAAAGGATGCAACGAAGCAAAAGGCTGGGGAATATGCAGATTATACG<br>TCTCAGAAAGCGAAAGAAGCAAAAGACAAAGCAGGGGAGTACAAGAACTATGCTGCAGATAAAATGAAGGA<br>AGCTGGAGATGCGACAATGAACAAAGCTAAGGAAGCTAAAGACGCGACGATGCAGAAGGCAGGTGAGTATA<br>AAGACTATACAGCTGAGAAGACAAAAGAAGGGACGGAGAGTGCAGCGGGGAAGCTTGGGGAGCTGAAGGAT<br>TCTGCTGCTGATGCAGCCAAAAGGGCTATGGGTTTCTTCGCTGGCAAGAAAGAGGAAACAAAGGTTTTCAA<br>GAGTTGAATATTTAATCATAATGTAAATGTTTAATTATTGTATAATTAAGATGATGTGATTGTGGTGCAGG<br>AAAAGCTGAGCGGAACAGAGGAAGAAGCGATGAGGAAAATGGAACATGTGAAGGTGGAAGATAGGAAATAT<br>GGAGGAGGAGGAGGAGGAAAGATGGAGATAAAAGTGGAAGAGTCTCGGCCAGGGGCAATGGTGGATGTGCT<br>ACGGGCTGCAGATAAGGCTCTGAACAATGTGGGACGGTTGGATGAAGAAGGTGTGATTCATGTGGAGCGTG<br>GTCGTGAGAAAATGTGA
CrLEA4-2	ATGGCGTCAAGGCAACAACCCGAGGACAGAGCCGAGGCTGCAGCCAAAGTTGCTTCTAAAGAACTCGAGCA<br>AGTTAACAAAGCCAGAGACCATAATTTGGCTATCGAGGACCAACAACAACATGACCAAGAAAAGCGTGGTG<br>TAATTAGCTCCGTTCTAAGAGCAGTGCAAGGAACCTACGAGAACGCAAAGGGAGCAGTGGTTGGCAAAAAA<br>GACCCTGCGGATCCCTATACCACAGAGGTAGTTCATCATGTTAACGTTGAGGATGAAGATGCACGAACCGG<br>AGAAGTTAGAGACATATCGCCAAACAAAGCAGGTTCTAAAGTTGGAGAGTATGCAGATTACGCATCTCAGA<br>AAGCCAAGGAAACAAAGGATGCAACGGCGCAAAAGGCGGGAGAGTACGCCGATTATGCATCTCAGAAGGCA<br>AAGGAAGCTAAGGATAAGACAATGGAGAAAGGTGGAGAATACAAAGACTATGCCGCAGAGAAAGCGAAGGA<br>AGCGAAAGATGCGACAATGAATAAGGCAGGGGAGTATAAGGATTACACCGCTGAGAAAACGAAAGAAGGAA<br>AGGACACAACGATGGGGAAGCTTGGAGAGTTAAAGGACTCTGCTACTGATGCTGCTAAGAGAGCTGTGGAT<br>TACTTGAGCGGAAAGAAAGAGGAAACAAAAGAGAAGGCTGCAGAAACTGCAGAGGCGACCAAAAATAGAAC<br>GTCGGAGACTGCTGAGACGGCGAAGAAGAAAGCTACGGAGATGAAAGATGCGGCAAAGGAAAAAGCTGCAG<br>AAACCACCGAGGCGACCAAAAATAGAACTTCCGAGACTGCTGAGACGGCAAAGAAGAAAGCTGCGGAGATG<br>AAAGATGCGGCGAAGGACAAAGCTGCAGAAACCACCGAGGCGACCAAAAATAAAACTTCCGAGACTGCTGA<br>GACGGCGAAGAAGAAAGCTACGGAGATGAAAGATGCGGCGAAGGACAAAGCTGCAGAATCCACCGAGGCAG<br>CTAAGCAGAAAACTGCAGAGGCCAAGGACAAGACGAAGGTCTCACCTTTAATTTAATTATTAACCATATTA<br>ATCAGTTATAACAAACTGATTGAATCAGTTATAACTACCTGATACACGTATGTGCCACAGATGGTAGCATA<br>GTAACACGCTGTTTTAACATATTTTAAATGTTTTGATGTGGATGCAGGAAAATGTGAGTGGAGGAGGGGAA<br>GAAGATACTAGAAGGAAAATGGAAGAACTCAAGGTGCACGGGAAAGGATACGGTGAGGAACCTGAGCGGCG<br>TGGTGGAGATAAGGTGGTGTTGAGAGTCGAAGAAAGTCGACCGGGAGCGGTGGCGGAAACGCTGAAAGCCA<br>CGGATCAGATGACGGGGCAGACCTTCAATGATGTGGGAAGGATGGATGATGAAGGGGTCATCAATGTGGAG<br>CGACGTAACAAATAA
CrLEA5-1	ATGGCATCAAAACAACAAAATCGACAAGAACTTGACGAGAGGGCAAAGCAAGGAGAAACTGTAGTTCCTGG<br>TGGCACTGGTGGCAAGAGCCTTGAGGCTCAACAACATCTTGCTGAAGGTTATCATCAATCTATCAAATATT<br>ATAACCTTAATCTTTTTCACTTTTTCGTTTTCATATTTATGTTATATATCTCACATCTCTCTTGCACCTAT<br>ACTTTTTTTACATCATTATTATTTATTAATTTATTTCTGTCTCTTTTGTTTTTATATCACTGTTATTTATT<br>AAATTTATAATTTTTTATTTTTATCTTAAAATATTATTACATTTTAATAACTCTATTACGTCCATATTTAT<br>TACCATTCATATTTATTGTTTAAGATACTATTACAATTTTATATTACTATTATTTATTAAATTTATTTTTC<br>TTTTTATCTCTTAAAGAATGTTACATAATTACATCATCATTTGTTATTTTTCTTTTCTTTTACGTCACTGT<br>TCTTCCAATTTATTTTTTATTTTTATGTCACTGGTCTTCCAAATTTATTTTCTTATATTTTTTTTATCTTT<br>TATTAGATACTAATACATTTATCATTTTTCTATTTTTATCTTTTCCTTTTTTGTCACTGATCTTCATTATA<br>TTTATTTCTTCTATATTTTTCTGTCAAGATATATATATATATATATATGTATAATTATTATTCTCACTCAG<br>TGTGTGTGAGTGATGGAAGTTATCTGTGTTTTGCTCTTTGGTTTGGTAATAATGATTTGGGTATTTTTGTG<br>ATTGTGTAAATAACAGGAAGGAGCAGAGGAGGGCAAACAAGGAAGGAGCAGCTGGGAACAGAAGGGTACCA<br>AGAGATGGGTCGCAAAGGTGGATTGAGTTCTATGGACAAATCAGGTGGAGAACGTGCTCAAGAAGAAGGCA<br>TTGACATTGATGAGTCTAAGTTCACTACTGCTTCTAGGAACTAG
CrLEA5-2	ATGAACCTTTCTTCTTTCACTTGTGTGAGCAATCATAAAGTTTTTCCCTTGGCTAACACTCGTAATTATCA<br>TCCTCATTCAGTTCCCTTGCAAAGGCAAAAAAGAGTCCTTAGAAGAAAAGGTTTGAGTGTGAATAACATGG<br>CATCTCAGCAACAAAACCGTGAAGAGCTTGATGAGAAGGCAAGGCATGGTGAAACTGTGGTTCCTGGAGGC<br>ACTGGTGGGAAGAGTCTAGAAGCTCAAGAAAACCTTGCTGAAGGCGTTGATTCAACCCCTATATCTTGCAA<br>GAACACACATATGTTAACCTTTTTTTGTTCATTGAAGTAATGTGTATTTATGATGTGTAGGAAGGAGCCGT<br>GGAGGGCAAACAAGGAAGCAGCAGCTAGGATCAGAAGGGTATCATGAGATAGGAACCAAAGGAGGGCAAAC<br>AAGGAAGGAACAGATGGGAACAGAAGGGTACAAAGAGATGGGGCGCAAAGGAGGGCTTAGCACTATGGACA<br>AGTCTGGTGGAGAGCGTGCTGAAGAGGAAGGCATTGACATTGATGAGTCTAAGTTTAGGAATCACTAA
CrLEA6-1	ATGGCAAACAACGGGAAGCAAGTGGAACACAAAACAGGAGAGTGCCCTAATCGCAAAGAGAAAGGAAAGAT<br>AGAAATGTTGCCTTTGGAAATTAGTCCATGCGTTAAATACAATGATGTACAAAAAGAATACAATCGCAATG<br>CTTATGACACACAACGCCATCTTCAGCCAAAACCAAACCAAGCAGGTGGAACCACACACATTGCTCCTACT<br>CCCTCAGATGCTCCATTGAAATTAACCACTAAATAG
CrLEA6-2	ATGGAGAATCAAACTGAGAAAAAGGCAGAGAGTGAATCAGGTAAAGAAGGACTTCCGATGAAGGATAGTCC<br>TTACCTGCAATACAAGGATGTGGAGGACTATAAGCGTCAAGGTTATGGCACCCATGGTCATCAAGAACCAA<br>ACCCAAATCAAGGAGCTGGTGCCACTGAGGCACCCACTCTCTCTGGTGCTGCTGTTTCCTCTGAATCTGAA<br>TTCAAAGCCACTGATGCCATTAATCGCAAGGGAGTCCCTTAATTAACAATATCTACCGTTCCTTTATGTAA<br>TAACAAATACTTTTTCCTATTCCTTGTTTCCTTTAGTTGTTTGTTAGATCATTACATGTTTTAAATTTGCA<br>TTTAGTATTCACCCCAAAAAAATAGAATAACATTTGCATTTAGTATGTTGTATTTGCTTATAACACGTTGC<br>GGATGTTGTAGAACTTTGCTATGTAAGAATTGAACGAGCAATTATCAATCTTTTCTTTTTTCCCTTCTCTC<br>TCAACTCTTAAGGAAATACTATTTATCGTTAGCAGTGGATAGAAAAATATCACTTTCTCTATTGTATTTTT<br>CGCTCTGCTGTTGTTTTTACAATTTGGGAAGCTATTTTTGCAATTTTTTCCTTTGATGAAAGGATGCTAAG

	TAACTAATAAACTTCCATAGTTGTAAGTTTTAAACATGTTAACAATGGTATAAATTTTAACTGTATGTGTG<br>GAAGAGATTTTGTTGGTAAAGATAAAGTTTATTTTTATGATGTTTACTATTAATTATTTGTTGGAGGATAT<br>TTTAGTATATACATAGAAAGAAAAGAATGCAAAGTAATTAAAAAATATAAATAGCTAATTTGAATGACATA<br>TTAAAATAATAGTTTATATAATTCAATAAAAAAATTAAAATTTGTATGAGATTTTTTAATATATATATAATAAA<br>TTATTACATAGGATAGAATTTAGTTAAATATTTTTCAGTAAAGTCATCTAAGATAAGTTAGTAAGAGAGGT<br>TAAAAATAAAAGTTTTGTAAGTTAGTAAATTATTTAATATATATGCAGATGTAACAAGAAAATCAATGTCG<br>GTAACCGATTTAGACTTTCATTTTGTGAATGGATTCGGAGGAAATTGCTAAATTTATATAGATATCATATA<br>CTAAAGGGTAATAGAGTATGATAGACTTTCTAACGTGCCATATAAGACATAGTTGGAGAATACAATTGGTC<br>TAGAGAATGATACGAAGTTTTTGAAGTCATGTTTTGTTCACTGCAATAAAGGCTACAGAGTAGTAGCCATT<br>TATTTGATGGAAACTTTCGGGAAGAGAACATTGGCTAAACAGAATTTTCTGATAAAAAAATACAACTAAAC<br>AGAAATGCAATTAAATATTGTAGTATTAACATAAATTCATTTGATATGCATATAAAATTTTCATTTACTAT<br>GCATATAAAATTCTTATTACTTAAGCACATAGCACCATCCAAAAATATATTGTGAAAAAAAATTATTTATA<br>TTTTTGGAAAAATTATCTAATATTAACATAAAGAATGTTCCCTATAACCTTAAAACTTCAAAACTTCTGTA<br>TACATAAAGGGAAATAAGAACATATACATAAATATATTCCTTAAAAAAATATTAAATATGTTTCTATTGCA<br>GCAAAATTAGTTCCGTAAATAAACAAAGAAAAGAAGAAAAAAAAAAATTATCTTACGATAAGAGAACATTCA<br>AACCATTTTTTACGGTGAGGGTTTTTAGAGATGAAAAAGACTACAATACGGAGAAAAAAATCAAAGAATAC<br>GAGAAGTAGAGGAGTAAAAAATGGCACAGATTTAATAACACTAGGGGCAGCTATGACATAAATTTGAGAAG<br>AAAAGGAACAAAAATTGAATAAATGAGAGAATGAGTATTTTCAGTTTGTTCTTTCTTAGTATTCAAAAACC<br>AATTAATTCAACAAGGGAAACACAAATAAAAAGTGCCATAAAATTAAGTTTATTAAGATCGAATACGGTTA<br>CTAAATAAAAATATTTATTATAAATTAAATAAAATAATTAGTGACCAAAATTAACTATTTCCATTCTTTTT<br>TAATTTTACGATCAAAAATTTGATTTTTAATTTAGCAGCAAAGTAACTTAATAAATTTTTTTATTTTAAAT<br>GACATCAGTCATTAAATTGATTTTTATTGACAATTAAATTTATATAATTATCAAATTTGATTTCTAATTAT<br>ATATATATATATATATAGTAAAATTACTGTCGATATTTTTAATAAAAAAATTTACGATATTAAAATTTTCT<br>ATGAAAACAAAACTAACATTCGTTAGAGATATTATGTGCACTTACATTAAAATATAAGGATGTCAATGTAA<br>TGATTTTTAATACTTAATATTTAAATTAAAACATCTAAATTTTAATAATTTTATTTTATTTTTCTATTTTT<br>TTCTTTCTATAATATTTTATCTATTTTATAGATGTCTTGTAATAAATTTTTTTAAATAAATAAAAATACAT<br>TCATTCTACATTCGTTATACGGTAAATAATTCAAAAGATACATCTTATTCGATAAGTAATGATTTAAAAGA<br>TTAAAATAATCTAAAAAATTTGGTTATTGTGATTAAAATTAAGAAAAATTTTAACTTTCAAATCATAAGAA<br>GAAATGGGAAAAAAAAAATGTCAACAAATGAAGTAAAAGAGACATATGATAAAACTATTGGAACGGTCATGC<br>ATTTATTCGGTATTTTTTATTTATTATAAAATTTTGATAAATTTGTTTTTATTATATTCAAAGTTATTTTA<br>AATAAAACAAAATATAATTATAGTTAATTATAAAAATATTAATATCATTTAATACATTATGTATTATATTA<br>ATTTATATTTTTATATAAAAATTAAAATATATGAAATATTATATATCTTTAAAATAAAATAAATTATTATT<br>TTGTTGGTTAAAAATTTGTAATTTAATATATTTTAAAATAATTTATAAATAAAAGATATTTATATAAAGAT<br>TAATATATTAAATAATTACAAAATGAAGAATAATCGAAATATGTAATAATGTCATTTTTACTATTTTATAA<br>AATTCTATTGATATAAAAGATAAATATTAAAAAATATCATAAAAAATTATAAAATGTTCGTATAAAGTTAT<br>ATAATTGAAAAGAAAAGTATATAATGTTATATTTTATATAAATTATGAGAAACTTATGTAAGGTAAAAAAT<br>AAAGAAATATTAAATATATTTTTAATAAATTTTAAGAATATTAATATAATAAACTTGGTGAAGATATGTCA<br>TGCTAATTTGTATCACCAAAATAGAGCAAGATCTTTCTATTTTTAAATTTTGTGTTAAAAAAAATATAAAG<br>TATCATTTTTTAAAATATTTTATTTATTTTTATTTTCTTGTTTCCATTTTAACTCTCCCTTTAAGAGATTA<br>ATTAGGAGCGGAAAATTGATTTTTTAAAAGAAAAATGAAACGTAATTGGTTTTGCCTTATTAACATTAAGT<br>TATACTAGTATGGGATAATACGGTAGAGTTACATTATGAAAGATACACAATCAAAGTAAAATACAAACTAA<br>TTGATAATTCAAATTTATTTCATCTAATCAAACTTCAGATATTTTGAGATATGTTTATATATATTTAATGT<br>GATGAATATCTAACTTGTATGTATTATATTTGCTGTAATAAATTTTAAAAATATAAAAATTGCTAATCAGT<br>TGTTGTATTTATTTCTATTTTCACAAATTTTTTTGATAAATTTTATTATTATATTGTTAATGTATTAATAT<br>ATTTTTTATTTGATAGTTTTAAACCTATAAAAACAAGCAAAAATATTTGACATTAAAATTAAATTTAATAT<br>ATTTTATCAAGTTTAAAATATTGACATTCATTTAATAAAATTGACTAATATAAATATTGAACAACGTTATG<br>ATTGAGAGACATGATATTGGCTTCAATGTTTAAACAATAAGAAAAGGATGACGACGTCTACAACAAAATTG<br>ATGGGAAAGGGGAAGTACACACTTGCTTCTCAGTTTAAATCTCTTATGATTGGGATTTCCAACCCTACAAA<br>CAGAATCTTGCTTTGGTGAGAGTGTATGCGCATATCGAGTGATGCCTTCTGAATACAACGAAGGATGAGCA<br>ATGATGATAAATGATATAATAAAAAATTATTTTATAAAAATTGTTATGCATTTGTTATATTTTTGAGATTT<br>ATTGTAGTATGATAAATATAAAATAAATGTTGATCTTATTAAATATAAATATACTGATGTTAAATTATTAT<br>TAATTTAGATATGTTATCCTTAAATTTATTAAATCAAATCATCTTTCTATAAACTATATTACAATTTAATT<br>TTATATTTAAAACAATTAGGTAGTAGCTTGGTTTTAGAAATAGTTTTCTATTTTTTATCTTTAATATTTTA<br>TATAAAAATAGTAAAAATAAAAGATATTTATTTTTTATTTTATTTTTTATTTTATTTTATTATTTTCAGTA<br>TAAAACTTTAAAACTAAAAACATAATAAAAATAATGTAATATTTTTGTAAATAAAAACAGAAAATATTTTT<br>TAAAATCAAAAGTTACCTTAGTAAACTTCTATTTTAGTTCCAATTTTATAAAATTTTGTGTTCTTTAATTT<br>TATATATTGTTTACTTTATTTTTTTATCAAAAAATATTAAAAATCATATTTATTTCAATTAATTTTGGTAA<br>AAATATTATAAAATAAATAAGATTTTTATTTAGTTCTAAATTAAATAAAAATAAAAAATAAAGTGACAAAT<br>TATTCTGATACCATAGATTATCATCCAGGCTTTGCAGTCATCTAGTTTAATTTAGCTTTTATTATTTTTAT<br>TTTATCAGCCAGTTTATTTTAGCTTTTGTCATTTTCAATTGGGAATTCGCTAAATTGAACTTTGCTCATGT<br>GCTTTTGTGAGAAATTGGCCAATATATAATCTTTTACCTTAAGAAATTAGAATTAATATTAGGATTAAGAT<br>TAATTTATTTTTAAAAAATTATATTAAAATAATTTTATTTAATATTTTTTATAGATATTAAATTGCACTAA<br>TTTAGCTCTTGAATTAAATTTTTATTATATTATTAAATTCATAAAAATTAATAAAATTAAATATTATTTAA<br>TACTTTAATTTAATAGATAATATATCTTTTAAAATATAAGTAAATGTTAATTATAAGATTATATACCAGTA<br>AGTTTTTAATTTTTATAAAAAATTAATATGTATAATCATCATATTAATATTATATTATTTTTAAATTTGTA<br>TAATTTACATAAAAAAAAGTTACTCAATGAAATCATTTTACAAGAATTAGTTCGTAAATAAGTAAATCCAC<br>ATCCTTAAATTTTAGAAGTATTATTATAAGACATGTTTTTATACTTAAATTTTTAAAGTATATTTTTTTTT<br>ATGGTGGGATAAATTTAAAAAAAAAAAGTCGAATAAAAAAAAAGGTGTGAAAAGTAAGGTCTTTTTATTTT<br>TTACCTTCGAAGCCAACTGGGCTTGAGCCGAACACAATCACAGACTGGGCTCTAACCTAAGTCCAAGGGAA<br>TTAGGTTTGCTATGAAAAGAAAAAAAGGAGGAAAGATTCCTATCCTTACGTTAAATGTTATTGACCAGAAA<br>AGGTAAAATAAGGAAAGACTCGCTTTACAAATAAAGATAATTTAAGATAAAATATGCCTTGTCATATGAGG<br>ATGTTAGGATATCCAGTAATGTCCCAATCTACAAATACAATGTACGAACATCATACAAATTAATCTTCACT<br>TATAGCAATTTCAAACTATAATAATTTTTTTTAATCAAACAATGAGGATAGAAAAAGGATTGTTACTACCT<br>CATGAATGAATCCTTCATTGAGGATGGTGATCAACACACTTTATATTATCTTTTAAATTCATTCGTTACTA<br>ATAAGACGAAAATTTATCTAATAATAAAGAATGTTAAAAAAAATGTACGAGATAATACAGTACATCCCCAT<br>GTAATTCAATTTCAAATATATACCAGAATTCACAGGTGTCCCCACCCGCACTTGGGCGTAAACATTTGACA

	AACCCACTTGCGTAACTTGCATTCATCCAAATTTTACAACAACAAGAATAGCATCTTTTTGTTTTTGTTTT
	TTAGCCCGATGCATCTAAAGCTATGGTAGAGAGGTAATGGTTCCACTAGTGGAAGGCTCAAAAAGGGACAA
	TTTCTTTCTTCAGTCCCAAACAATAAAATTATGACGTAGATAAAAAAAGAAAATGAAGACACCGTTTTGTT
	GGGTCTCTAGACTGATAAGTGATGACTACTTTAGTGCTGGTCAGCACCTCCTTTACCTCTTTTTATATTCC
	AAGTGTCTTTGTATTTGTGTCACTGTGTCACACACATCCACCTTCCCCTGTTCAAATTATCATTAACCTTA
	TAATCTTATTCATCCTTTCCCCCAACACCCAGCAGCATCAAACCAAACCAAACCCGTTCCTTTCGTTACAT
	GGCGACAAAGAACGTGAAAGGAAAATGGAACAAAGCGGAAGAAGAAGAAGATGAGGAAGAGGAAGAAGCAT
	TGTCTCTTTGCGATCTTCCGATCAACCTGGAAGCCCAACCGAGAAATCAAGAACACAAACATGTCAACGAA
	GCAACACAAGAAGAATTCAATTTCCGCTCTTGGAGTGGTTTCTTTTCCAAAGAACCAGAAATGTGTGTCGC
	GGATGAAGTTTTCTTTAAAGGTCAAATTCTCCCAATGCGTCTCTCGTTCAGCACCGAGGCTGGTTTATTAA
	CCACAGGATCTCAACATTATGCTAAGCGATTTATCAACGAGTCCTTGGATCATGCTTCTCTCGGTGATTTT
	CAAAGTAATAATAGCAGTAGAAGCAGTAGTGTTAGAAGTCAAAATTCGTCAAGTAGCACCAGCTCCACCAC
	CACCACCACCACCACTACAACAACAGCAGCAGCACCACCACGATTTAAACCTAGCGTTAGAAACCAATTTC
	ACACGCACCCTAGTCCGAAACCTCAATTAAGAGTATCTGCTCCAAGACAATTAGTTAATCAAGGTAGAAAA
	TCATCATCAGCATGGGAAATTTTCCGTTTGGGTGTGGTCCCTGCTCCCGAAATAGGATTGCAAGACCTCAA
	GGTTCGCAGTAGCAACAGTGCGAACAGGAACTGTGTGAGTCGCAATAGTAGTAGCAGTAGCAACAACAGTG
	CTTCCAATAGTAATGCGAAAAGTGTGAAAACGAGTAATGATGGCAACAAGAGCAATCATGTTTTGAAACAG
	TTAATTGGCAAAGGCGGTGGTTTATTGAGTGGTTGTGATTGTTCCTTTGAAACGGTGCAATCGAACATGGT
	AATGATCAAAGGTGGTAAAAGCACCAACAAGGCAGAAAGTACAACGCACGCCGTGAAAGAAAAAGTGGTGG
	AATTGAAGAAGCAGAAGCAGAGGCAAAAGCAGGGAAAGAAGGTTATGTCACGTCGTCGAACGTTTGAATGG
	TTAAAGGAGCTTCATGCAAGCCATGATCCTGATGATGAGAAGGCTTTGTTATCAAAACCATCATGA
	ATGGCAAATTATCAGAACCAGTACGGTGCGGTTCCTACAAGCAAGGATCTGATTCAGAAGGAAATGGACAC
	CACCGGAGACACCACTGGCGTTGGCGGTGGCAGTGGCAGTACTAAAGTGGTAGGAGATGATTATGATAGAA
	GCAGCGCTGACACTACGGGTGTGAGCGAGCAGCATCACAAGAAAGGAATAATAGAGAAAATAAAAGAGAAG
CrDHN1	CTTCCTGGAACACATCATCACAACAAGTAG
	ATGGCAGAGGAAAATCAGAACAAGTTTGAGACCACTGAGAGCAGTGAGGGGGAGATCAAGGATCGTGGTCT
	TTTCGATTTCCTCGGTAAGAAAAAGGAAGAAGAAAAGCCTCAAGAGGAGGTCATCGTCACCGAGTTTGAGA
	AGGTCACAGTGTCAGAAGAGAAAAAAGAAGACGAAGGAGAGAAGAAGCACAGTCTCTTAGAAAAGCTTCAC
	CGATCCGACAGCAGCTCAAGCTCTGTAAGTTTTTTCTTATTTATTTATAAAACAATAAGCGCATGCATGTT
	TAAATCAGTTTAGTTTTTTAAAAATAATCGTTTATTTTAAAAAAGTTGAAACAAAATGTTGTAAGTTGTTG
	ATCATGGTGATTAGATTGATAAACTTCTTGACGAATATTTATAAAAGAAAAAAAAATAAAGTTAATATAAT
	CATGCTCATGCATTTCACTTTTGAAGAGATCTTTATAGTTATTTATAAATTTAATTAATAGAGTTAGATAT
	TCTTATCTCGTTTAAATCTTAATTTTACATTTAAAAAGCTTTTAAACATAGATATTGATTTATTATATTAA
	AAATGCTAATGTCGTTTTGATTGGTATATATGGTGATTATGTTTGCAGTCGAGCGAGGAGGAAGGAGAAGA
	AGGGGAGAAGAAGAAGAAGAAAAAGAAGGAAAAGAAGGGATTGAAGGAGAAGATCGAGGAGAAGATAGGGG
	ATGATCACAAGCACGAGGACACAAGTGTTCCAGTTGAGAAAGTTGAGGTGGAAAAAGTTGAGGTTGAAAAA
	GTTGAAGTTGATCCAGCACACACTGAGGAAAAGAAGGGTTTCCTCGACAAAATTAAGGAGAAGCTACCAGG
	TCACAAGAAGACAGAGGAGGTTCCACCACCACCACCGCCACCTGCTGCTGCATCATCAGAACATGGTGAGG
	GTGCTCATGAGAAGAAAGGTATTTTAGAGAAGATAAAAGAGAAGCTTCCTGGTTATCACCCCAAGACAGAG
CrDHN2	GAAGAGAAACAAAAGGAGAGTGCCGGTGACTGA
	ATGTCTGAAGCCCAACTACGAGACCAACATGGCAACCCTGTCCAATTCACCGATCAATACGGTAACCCGGT
	TACATTGACCGATGAGCATGGTCAACCCGTCCAGCTCAAGGGCGTGGCAACCACTGGTGGTGCAACCACGG
	GCACAGCAGGATCTGGATTTGGAACATATGGTACTGGTGCATACGGCGGTGGTGCAGCCACAGGCACAACC
	ACCGTCGCAGATCTGATAGCAACCGAGCGAGCGACTGAAATGCCAACTGCGCAGCATCATCAGAGGGAGCC
	TGGAGAGCTTCGTCGTTCCTCTAGTTCAAGCTCTAGCTCGGTATCATTAATTATCATATCGTCGATTAATA
	ATTTTTAACCATAGCTCTTTTCGTTTCTTTTTTCTCTCTCTCTCTCTCTCTCTCTCTTTCTCTCTCTCT
	CTCTCTCTCTCTCTCTCTCTCTCTCTCTCTCTCTCTCTCTCTCTTTTCTTATAAATCATATTCGCACGCAT
	TACGGCCCATCCGAGTTTATTTCCTTTTAATGATTTTTTTACACAAAGTTATTAAGATTCATTTATTTTTC
	AAGAATAAAGAAAGAATATATTGTTTTTATAACTGAAGTTGTTGAGACTAGAGACCTTTTCAAGTTTCAAT
	CGTTGCTTTAAAGTCTGTAATCTCCATGACAGATTGTGATGGTCGCGGGAATTACCTTTTCATAGGCATAT
	TACTTTATGGAAAATTGGTAATTTTTTTTTTAGTAACATTTATTTATTTATGTTATTTTTATAGATTTTTAA
	ATAAGGTTATTTTTTTTAAAAAAAATTTCTGCAGTTTTTAATTTATTTTTTAAAAACTGAATTGACCAATTTT
	CTAGGGAAACATCAGAAAAATAATATCCTTTGTGGAGAATCAATATTTTCTTTGAATAAAGAAATATTTCC
	TGTGTATCTAGAAAAATGCTCATTCAAAAATTAATTGAAGTTTTATTTAGTCAATTTTTAAAATTTTTTTT
	TTACTAATTTTCAAAACTAGTAATGTTCATAAAATTCGAATATAAAATTTGAACCGAACTATCCAAAAACG
	GAAAATATTAAAATAATAAATTATTTTTTATTGAATTATATTAAATTTTATATATACATAATATTTTAGTT
	ATAAATTTTATATTTATATTATATAATTTTAGATTTGATAAAATTATAATATTTTATTGTTTTTCATTATT
	TTTTTATTATTAACTTTGAAAATGTATTTTATTACTTTAATGTTTATAAATTTTAGATTAATTTTATTGTA
	TAAATTTTATGTTATTTTACAAATATCTTTAATTAATAATAAGAGGAGAGAAGAAGAGATTATAAAGTTAT
	TTTTTACCTTATTTAAACATTTTTGTCGTATAATGTTTGACACGTCACCGAATTGTAGTGTATTTCCTAGA
	AATCAATCCAAACGAGCATCCTTCATCATATTATTTTCTTTCTTTAATTTGAACATGTTACATTATTTGTA
	TTTTAACAATTTAAACGTTTTTTATTTATTATTGAATAATTTTTACTTTCAATATTTATTAGTACCTAATC
	CAATAAATTGAGTTGGAGTGAAACAAATCATAAGCATCAGATTCCTTTACAAACTCACTTTGGGATAGTTG
	GTATATTGTTTATTTTGAACTTCGTCCATCAGCACAAATATTTTCTAGGAACAGGTAGTAGCTAGTGTGAT
	GTTAATCTTACTTTGGTTCAACATTTTAATTAATACGTAGTCTGAGGATGATGGCCATGGTGGGAGGAGGA
	AGAAGAAAGGACTGAAGGATAAAATAAAAGAAAAGCTACCAGGGGTAGGAGGTGAACGAAAAGATCATGCA
	CACACACCTACAACCACCACCACTACATCCTTTGGTACTGGTGCTACCCACCCACCGCCACCCACCGCCAC
	CCACCCTCATCAGCATGAGAAGAAGGGCATAATGGAAAAGATTAAAGAAAAATTCCCTGGCCATCACACCA
CrDHN3	ACTGA
	ATGGCAGAGGAGAAGCTGAACAAGAGTCATGAGTACGATACCACTTCCACCAATACTGAAACTGAGATCAA
	AGACCGTGGAGTTTTTGATTTTCTCGGTAAGAGAAAGGAAGAAGAGAAGCCTCAGGAGGAGGTGCTAGCCA
	CAAATTTTGACAATAAGGTCAAGCTTTCCAATGAACCCGAGACCAAGCTCCAAGAGCAAGAAGGAGACAAG
	AAGCACACCCTCTTAGAGAAGCTCCACCGATCCAACAGTAGCTCCAGCTCTGTAAGTTTTCTTTCTTAATT
	TATTTTTTATTGAGATTTGCGTATGAAAGAACAGTGCTAATGAAAATTGAAGAGAGAGTACTACTCTAGTA
	TCGAGTGTTTTGGATGTTGTAGTTGTAGTGGTATGTGCTGCTTCATATGTTTATGCTTCAATTTATTAATT
CrDHN4	ATATTAATATGCTTGATATTTATATGTTTTTGCAGTCCAGTGATGAGGAGGAGGAAGGAGAAGGTGGGGAG

	AAGAAGAAGAAGAAGAAAAAGGAAAAGAAAGGGCTGAAGGAAAAGATAGGAGGTGATGATCACAAGGAACA<br>GGACACTGCTGTTCCAGTTGAGAAAGTGGAGGTTGATTCAGAGCACCCTGACACTGAGGAAAAGAAGGGTT<br>TCCTTGACAAGATTAAGGAGAAGCTGCCAGGTCAGCACAAGAAGGCAGAGGAAGTGTCCCCGACATCATCG<br>GAATATGTTGCTGCACACACTGCTGAGACTCATGAAGGGGAGGCTAAGGACAAGGAGAAGAAGGGCCTTTT<br>AGATAAGATCAAGGAGAAGCTTCCGGGTTATCACTCCAAGCCAAATGAAGACAAAGAAAAGGAGACCGATA<br>CTCATTGA
CrDHN5	ATGGCAAGTTATCAGAGTCAATACGGTGATCAAGGTCGTAAGACCGATCAGTATGGAAACCCTGTGACTCA<br>AACTGACCAATACGGTAACCCTGTGAGACAAACCGGTGAAACCGGTAAGCAATATGGAACCACCGGTTACG<br>GAGCTAACACTGCGGACACTGGCTCGGGTCATAGAAGTGGAACCACTGGTGGCTATGGAACTCGCACTGAG<br>GGTGTAGGCGCGGGTTATGGAACCACTGGTGGCACCACCACTGGTTATGGTGCCACTGATTATGGAAACAC<br>CGGTGGCACCACTACTGGTTATGGTGGCACTGATTATGGCACCACCGGTGGCACCGGTGGTACCGGTTATG<br>GAAGTGGAACAGGAACTACCGGTGGCACTGGTTATGGAAGTGGAACCGGTTATGGAATCAACACTGGAGGT<br>GCACATACTGATGCAGGGTATGGGAGGGAGCATCGTCATGGACATGACCAGTCTCATGGTGGGTATGATGC<br>ATCGGGGGAGCAACATCATGAGAAGAAAGGGATAATGGATAAGATCAAGGAGAAGCTTCCTGGAACTGGAG<br>GACACAACGAGAAGTAG
CrSMP1	ATGCAATCTGCCGCCGCCGTCAACACCGACGCTGGCATCGTTGGCCGGCAAGACATATCGGACGTGGCTAG<br>AGAGCAAGGCGTGAGCGTATCCCAAACAAAAGTGGGTGTGAACCGTGTGATCACAGAGTCGGTGGGAGGGC<br>AGGTGGTGGGGCAATTCGTGGAACCTGATGTCCCTATGAATTCCCCTGGTATGGCTCTCGAACCAGATGCC<br>ATAACCATTGGAGAGGCACTGGAAGCGTCGGCGGTGGCTGGAGCGGGTGATAAGCCTGTGGACCAAAGCGA<br>CGCCGCTGCTATACAAGCTGCTGAAATGAGAGCCACCGGAAAGAATGAGACAGAGCCTGGTGGGTTGGGTG<br>CGAGGGCCCAATCAGCTGCCACTCACAACACTCGCACTCCCTCTTACTTTCATAAAACCACTTTGTCTCAT<br>GTCTTGGCGGTAACTAACTCCTAACAAACTCATAACAAACTCCTAACTAATTATATTAATTAGTTGAGTAA<br>TTTCCATGTGAATGCAGGATGCTAAGGAGAAGTTGCCAACGGACAAGCATGTAACGCGTGAGGATGCAGAG<br>GGTGTCATTGCTGCCGAGCTTAGAAACAAACCCGACATGAAAACCACCCCTGCCGGGGTTGCTGCATCCGT<br>AGCCGCTGCCGCCGCCATTAATCAAAACGCGTACCTTACAATAATATTATAA
CrSMP2	ATGAGTCAAGAGCAGCCTCGGAGAGAGGAAGCCATCAAATATGGAGATGTGTTCAATGTGGAAAGAGAGCT<br>TGGAAGCAAAGCAGTGGCACCCGTCGACGCAGCGATGATGCAGAAAGCAGAGACTGCTATGATTGGGAAGA<br>CCGAAAAGGGTGGTGCTGCTGCAACCATGCAAGCTGCGGCATTGAAAAATGAGAGAGATGGCGTTGTTGGA<br>CATAATGATATCACTGAAGTTGTTGCACAGAGTGGTGTCAGTGTAACTGAATCTGATCTTCCAGGGAGGCA<br>AGTTATTTCGGAGTCAGTTGGAGGACAGGTTCTTTATAAAAACCTATCTCTTTTCTTATTCTTTATTTATG<br>TATAGGTAAAATATATATTTTTAAAAATTATAAAATTTTAATTTCTAAAAATAACAGGTTTTGATTGCTTA<br>TTTTTTTTTACATAAATCAATATTTGATAAATGTTTATCATTTTAATGATAAAATGTTAAAAATGATTTAT<br>TTTAAAATTTGTTGTTTTAAAAATGGAAGATTAGAATTAAAATTAAAATTTTAAAATTTTAGGGTTTAAAA<br>ACATATGCTATCTTTTATGGAGTGGCTGTTTATTTTAATTCTTGTTTGGTTAATTGGTTTCGTAGGTTGTT<br>CACACACTAGAGGAAAAGGAAAGGAACATTTGTAAATTAGTTGAGGTATTAAATCTTTCAAGATAATATCA<br>TATACTGTACTAACTAATTAAAAAGTGTTTTTTATATATGAAAGAAAAACAGAGTTTAATTAAAGTTGATT<br>CGTTTAAAAAACTGTGAAAAGCAATTAAATAAGCGTGTTATTTGTATTAACTGTTAGGCACTGTATGGTGG<br>ATGAACACCATGAATCAAAGAAAGATGTTATAATATATTTACCAATTTGATCCTCTTCTTTTACATGTACA<br>TTACCCAACTAATTAATATAATAATGATACCAATTACGAACAGGTGGTGGAGCAATTTGGGCAAAAGGTTG<br>CATCGAATACGACGATGCCATCTTCTCTTGCTCAAGACTTGGGCTCAAGAGTTGGTGGTGGGATCACCATA<br>GGTGAAGCATTGGAGGCGACGGTTTTGACGGCGGGAAAGAAGGCGGTGGAATGGAGCGATGCGGCGGCGAT<br>TCAAGCAGCGGAAGTGAGAGCCACCGGACGTACCAACATTGTTCCCGGAGGTGTTGCTGCGGCGGCACAAT<br>CAGCAGCCACCCTGAATGCTCGAACTACAAAGGACGAGGACAAGACCAAACTCGCAGATGTTTTGTCGGTA<br>CGCAAACTGAACCTCACTCGAACCTTGTTTGTGTGCGTCCCACTGCAATGTAGGACATAAGGCTCAAGGAT<br>CATGGAGTTCACTTTAGAATAATTTTAATTTTCCTTTTTTATAAAATTTTTAATTATTAAATAAAAATATT<br>AATTAGTTTTATATTTTAAAAATAATAATACATTTTTATTTAATACTTATTTTATAAATAAAGAATTATAG<br>GATGATATCAAAAGAATAAGTCGGTTTTAGATAAATATATATTTAATTAAGTATTTATTTAATATAATTTT<br>TTCTTAAATGACAATTTATTAATAAATTGTAAGAAGAGGTTATTTGATAAATTTTTTCATGAAATTATTAA<br>CATAGCTTAAAGTAAGTTTTTGGACCTGTCTTAATGAGCAAATCTTCAAGTGAGATTTTGAAATGGTAAGA<br>ATAAAATATTCTAGTCATTAAAATTTTAAAGATTTTACTTTATTAGTTTTTGACTTTGTATAAAAATCATT<br>TATTTTATTTTTTAACTTAATTTTGTAAAGAAAAACTAAAATGACTTTTTTTAAAAATAGAACTAACGAAA<br>GTTTTTTAAAAATAAAACAAACGGAAAAATTTTAAAATTTCTTATCTAAAATTTCTGATCCATATAAACTA<br>GAGACAACCCACTCTCAAGCTTAGGGGGCAATTGCTTCTCCCAAAAATATTTTTTGTTTTATATTGTATGT<br>ATATGAATTTATATACACAGCTGTTGCATGATCGTACTGTTACGACCTTTTAGATCCATCAGTGATTTTAA<br>GGATTAATATAAGTTTCTTTTTTAAAAATAATATTACAATTATATTTATATATTGCAGGATGCGACTTCGA<br>AATTACCATCAGACAGACCAGCGACGAGGCGAGATGCTGAAGGGGTAACGGGTGCAGAAATGAGAAATGAT<br>CCTAACCTCACTACTCATCCAGGGGGCGTGTCCGCATCCGTCGCTGCTGCTGCTAGGCTTAACCAAACCAA<br>TAATAACTAA
CrSMP3	ATGAGTCAAGAGCAGCCAAGGCACCCCCAACCCGCCCAAGATCCCATCAAATACGGTGACGTTTTCGATGT<br>CTCCGGCGACCTCGCAAAGAAGCCCGTTGCACCCGAAGACGCCGCCATGATGCAGAGCGCCGAGACCCGTG<br>TCCTCGGCCAGACCCAAGCCGGGGGAGCAGCTTCCGCCATGCAATCCGCCGCCACTCGGAACGAGCAGGCC<br>GGCGTTGTCGGTCACCGAGATGTCACTGATGTCACCGGCGACCGTGGTGTCACCGTCTCGGAGACTCAAGT<br>TCCCGGAAGACGCATAATTACGGAATCAGTCGGTGGACAGGTTCGGTTTTTCAGTGTGTGAATGTGTTTGC<br>TTTTAGGAATTTGAGTTTGGATTATCATCTTTTATCTTTGCCAAAACCCATAAAGAATAATTTTAAATCCT<br>TTTATTTTTCTACAACTTTTTATTTTTCAAATAAATAATTTTAAATAATTCGTATCGTATTTTGAAAATAA<br>TAAATATATATTTTATAAGCTACTTACCAGAAGAATTGATAGTAAGATGTATCAGAGTTTTGACAAGAAAT<br>GGGATATACTGCCCGTAATCTCTTTAAGAGTTGAAATTTATTTCATCAATTAATAAGCAATATATTCAAAA<br>TAGTAAAATAAAATAAATAAACATATATTAAAAAGTTTACAAAATTAATTTTATAAACAATATTTAATTCA<br>TTAATATTTTTTGTAACTTTATTTTTTGCGAATTTCACATTTTAACAACGTAACGAGGATTGTTTATATTT<br>TTTATTTTATATTTTGAAGGTTGTGGGACAGTATGTTGAGCCGACTCCGGTTCAGGCGGGACGAGCCGGTG<br>CGGTCCGAGACAGTGCGATAACCATAGGGGAGGCGCTGGAGGCGACGGCGCAGACGGCGGGTGAGAAGCCG<br>GTGGATAAGAGTGACGCGTCGGCGATACAGGCGGCGGAGGTGAGAGCGACGGGCAGTAATGTTATAACGCC<br>GGGAGGGCTTGCTTCTATGGCTCAATCGGCGGCGGCCTTCAATGCTGAATGCAAACGTGACCAGGAGAAGG<br>TCAAGCTCGCCGATGTATTGACGGGGGCCACCGCGAAGTTGCCGGCGGACAAGGCGGCGACAATTGAAGAT<br>GCTGAAGGAGTGGCGTGTGCTGAGGTGAGGAACAACCCAGATGCGACGGCCACTCCTGGTGGCGTGGCAGC<br>CTCTGTGGCTGCAGCTGCTAGGCTCAATGAAAATGTTAATGTGTGA

	ATGTTCACAACCACTATTTGGAAGTTCCTCACAATCTCTTTTCGATCAGAAACAATGAGTCATGGCCAGCC
	CAAGAGACCCCAAGCAGAGGATTCTTTTCACTGTGAACCCATCAAATACGGCGACGTTTTCAAAGTTACCG
	GCGAGTTGGCGTCCAAGCCGATTTCACCACAGGAAGCCGCCGCCATGAAAGCCGCCGAGGCTGGCGTCTTA
	GGGCAGCCGCAGAAAGGAAGCCCAGCCGTAGTCATGCAGGCAGCTGCGGCAATCAACCAGGCGTGCGGTGC
	CGTTGACGGCAACGGGGTCGCCATCAAGAAGGAAGACGTTAGTGTAACTGAAACAACAACCGCTGGCAACC
	GTATCATAACAGAATCTGTGGGTGGCCAGGTCCTCGGGAAGCGTGTGGACACTCACGCGCCTGTGGTCCCC
	ACGGATTTGGGTTCTGCAGTTATAGATGGCGACCCGATAACTATTGGAGAAGCTCTGGAAGCAGTGGCCAT
	AGCGGTTGGGGATAAACCGGTGAACCAAAACGACGCCGCTGCGATCAGTGCCGCGGAGATTAGGGCGACCG
	GCGAGAAGAACGTAAGGGCCGGTGGAGTGGGGGCATCGGCACAATCGGCAGCCACTCTCAACAGCCACGTC
	ATGCGAGTCCAAGATATGACAAAACTTTCTGATATATTAACGGTAACAATACTCTTCGACCCAGTCTTCAA
	TCTAATATTTGACACGGAAGTTACGGAATAAAAAAAAAAAACAGTGATTTACGGAAGAAAAAATTGAAATT
	TAAATAAAAACTTAGAAGAAAAAGAAATTTTGAAATTTAAATAAAAAGAGAAGGAAATGAAATTTTTTAAA
	TTTGAATATTAAATGCTAATCTTTTGAAAAGAGAAGGAAATATTTAACTTTTTTTTTATTTTTCTTCCTTA
	AAATTCTTGGTATTGTAATATTAATTTCAATTTTTTATTTTATAAAAAATTAATTTAATCTTTTACTTTAT
	AAAAAGTTTTATTTTCAAATCATTTTGTTATATTTTGTTGTTAACATTTTATAAATTTTCCAAATAAATAT
	TTGTCGATTATTTATGGTAATTTCTTTTATCTATTGTTATTTATTTCATAATTTTATATTTATTATAATTT
	TTATTAGTTTAAAAAATTTTAAAACATAAATATAAAGTTAATTAAAATAAAATATAATAGAGATTCTAATA
	AAAAATTTATAAAGTTAAAATAAAAGTGAATAGTATGAATTACACCTAAATTAGTTAACATCAGTCAAAAC
	TTATAAAATCTTGCTCTAATTACGACGGCGGTAATTAGAAATATTGGCTCTAAAGTAAGAAATTTCTTTTT
	TTTTTTTCATAAAACTTTGGAATTCCAAAGTTGTGCTGGATCTCGGATCATTTTGAGTTGACTTATGAAAA
	GCTGGTAGTGTCTATTTTTTTCTCTTTACAAGATTTAAATTCGAAATTATGAAATAAGGTCCATATTATCA
	TTAATCAGCCAGCGGCAGGTACGTTTGGCTAGAAATTAATTAATGTGTAACACTTTACATACACTTTATAT
	CAGTTTGATTTTAAATATAAAGTCACAAGATTATTAGTGAGATTTGAATGAAAATTAATTATTAAAATAAG
	TCATAAAACTTTTGTATTTTGTAATTGGTGATAAAAATATGACTTCAAGAAGTGTGTAGTGTCGTATATGT
	GTCATTACTATTTGAAATGGGATGGTGATTGTAGGATGCGACGGATAAGCTGGCCATGGATAAGGCGGTGA
	CGCAAGAAGATGCTGAGGCGGTGTATACAGCGGAGATGCGGTTTCCATGGCGGGGGGATGCAACGGACGTG
CrSMP4	ATTTCTGAACCCGGTGGAGTGGCCGCATCCATGGCCACTGCAGCAAAGCTGAATGAAGAAAAATGA
	ATGGCTGAAGAGAAACACCACCACCACCACCACCTCTTCCACCACAACAAGGACGAGGATAAGCCTATAGA
	AACTGAGAGTGGTTACAACAAGACTTCAAAATACACTAGTAATGAACCTTCCGTTGGCTATGACTCTGGTT
	ACAACAAGCCATCGTATGAGTCTTCTGGTGATGCCTATGAAACTGGTTATAACAAAACATCATATGACACT
	TCTGGTGGTGCCTATGAAACTGGTTACAATAAAACATCATATGATTCTTCTGGTGGTACCTATGAAACTGG
	TTACAACAAAACAGCTTATTCCACTGATGAGCCTTCTGCTGGCTATGGTGGTGGTAGATACTCTGACACCA
	CTGGTGGTGCCTACACCAAAAAAACCGGTGAATATGCTGCCGGTGGTGGCTATGGCGATGATAGTGATCGA
	CGTGATGATGTTGACTATAAGAAGGAGGAGAAGCACCACAAGCATCTTGAACACCTTGGTGAGTTAGGTGC
	TGCAGCAGCTGGTGCTTATGCCTTGGTAATTTTCTTTACTAATATTTATTATTTCCTATCTTTTTTCTCTA
	ATTACGATTACATATAAATAGTAAATATTTTCACAAATTAAAGTGAAAGTGAAAGAGACTGGATGTCAATT
	AATCTAAGCACTTCAATGTTTTACATGGCACAAAACAATATTTCATTTGTTTCTTAATTTTTGTTTAATAG
	AGGCCTGCGTGACTGAAGACTAACACCTTCAGGTTCAAATCTCGTATATTTGTCATATTATATTTTGAAAA
	GGGCTAATCAGTTCTCTAAAGAAATCCCAAAAAAAATCAATATGTAAACATATTGATTAAAAAATTAATAC
	CCATCTTTATTAAAAAATATCGTTAGTATATTGTCATTGTATTCCTAATGTATTTTTTTTATTAAAACATA
	TATTGGCAAGACCCTTATATTTTATACGTGAAATTAGGCATAAAAATTTTACAGAAATAAATCTTATCATG
	TTAAATTAAACATGGCAAATTAGTCACATTAAAATTTGTATTAATCTCTATTTTAATAATATTAAATAGGT
	GAGATAAATGCTAAATTTAAATTTTGCCCTTATGATACTTGGAAAGCAAAATTTAAAGTGACTGATATGGC
	TTGTTTGGTGAAATGGTTTGAAATATATATATAGCATGAGAAGCACAAGTCAGAGAAAGACCCAGAGCATG
	GTCACAGGCACAAGATAGAAGAGGAGGTTGCAGCAGCGGCTGCAGTGGGAGCTGGTGGCTTTGCCTTTCAT
	GAGCATCATGAGAAAAAGGAAGCAAAGGAACAAGATGAGGAAGCACATGGAAAGAAGCACCACCACCTGTT
CrASR1	TGGCTGA
	ATGGCTGAGGAGAAACACCATCATCACCACCATCACTTTCACCATCACAAGAATGAAGAACAACCTGTTGA
	AACTACCGTCTACTCCGAAACCACATTTGATGTTGCTGGAAATGTGGATGACTATAAAAAGGAAGAGAAGC
	ACCACAAGAATCTAGAACACCTTGGTGAATTGGGAGCAGCTGCTGCTGGTGCTTATGCTTTGGTGAGTTAT
	ATGATCATGATTAAGATTTTTTTGTTTTTTTGTATTGTTATACACTTGTTAATTTGATGTCAGCAATTTTT
	CATTTTGGTTCGATTATTAAATTAATTTATTCTTCTTGTAATTTTAATTTTGACATATAGTCTAAAAATTT
	ACTATTTACTTGTGATATTTTTTTACAATGATTAACATATATTTACATTTTTTTATTCTCCGCCCTTTATT
	TTTTTTTTCTTTCAATCACATCACTGATCACATCTTTTTTTTTAATACTTTTTATGTACACTTAATTTTTT
	TTTCTCTTTTTTTAATATCCAAAAGAAAACTTCAAAGTCCTCTTAGATTACTCAGTTCATACTAAGATGTT
	ACATACCCAATGAGTCCCTGCACCATACCTCTAGCCTAGTTACATCTCATCACCTATGCAACCTTGACTTT
	TCTAAACATTGCAATTGAATTTGATGAGACTAACAAATATAGTTTGATAAGAAATAAAAGTATAACTTGAT
	TATCATTTTGATATCACTAATTTTTCTACAATTATATAGTTGGTAATTAAAATTTCTTTAAAACCTTAAAA
	TATTATTATCATTACTGGAATATATGAATTTTTTTAAAACCGAATCTCCCCACTTGACCAAAACCATGTCC
	ATACAAGTACACATTGATTATATTTGTTAATTTCTTTTTCATGTAAACATTGTGAATCATTTTCCATTTGG
	TTATGAGGGGGGATACCAAATGTTAAATAAAATGGTGATTAATTTGTTTAATCTTTTTTATACATTAGCAT
	GAGAAGCACAAGGCAGGTAAAGATCCAGAGCATGCCCATAGGCACAAGATAGAGGAGGAGATTGCAGCTGT
	AGCTGCGGTTGGGGCTGGTGGGTTTGCATTCCATGAGCATCATGAGAAGAAAGAAGCCAAAAAACAATATG
CrASR2	AGGAAACACATGAAAAGAAGCATCATCACCATCTTTTTGGTTGA
	ATGGCTGAGGAGAAGCACCACCACCTCTTCCACCACCACAAAGATGAGGATAAGCCTGGCTATGTTGATGA
	GGTTGATTATAAAAAGGAAGAGAAACACCACAAGCATCTTGAGCACCTTGGGGAACTAGGTGCTGCAGCTG
	CTGGTGCTTATGCTTTGGTAATTATATATCTTTGCTGAATACTATTTTGTTCACCATCGCTTATCCTAATT
	TCAAACATATTTATTATTTTTGTTTAGATAATTTTCCCTTTTATGATATATTTTAACTTTAAAGTGCAAGT
	TTCGATAAAATAAAATAAATAAAGTGCAAACACTTTAGCGATCTAGGGAAATATATCTCGCTCATGCATCT
	CTGAGACTTGGGCTTGTGTAAAGTGCTTAAATTTCAAAAAATTTTAAAATTCTTTTATCATTGCATATAAA
	AGTTATCATGTGTTTTAAATTGGGACAAGATCTAATTAATCCCCTGAAACATGTGTACCAATAATTTCAAT
	TTAGCTAGCGGAGTATATATTCTGTGCAAATTTATACTTGTGAAACTTTGATTGTAATAGCTAGAATATAC
	CAAAGTTGTGTAGATAGATAATATTATTTCATGAGTTTGATTTTGATTAATGTTAATATAAATACGTTTAA
	CCATACATCTCGTACACTAGCCAACTGTGATTTATTTTGTCTTAAATTAATATTGCTGAGTGTGTATATCT
	TCCACTTTTTCATGACGCTTAAAAATAAAATAAAATTAAATGGATTATCTTTGTTGCAATATTTTATGATA
CrASR3	GCATGAGAAGCATAAGGCTAAGAAAGAGCCAGAACATGCTCACAAGCACAAGATAGAAGAGGAGATTGCGG

	CAGCAACTGCTGTTGGAGCTGGTGGTTTTGTCTTCCATGAACACCATGAGAAAAAAGAAGCTAAGAAGGAA<br>GATGAGGAAGCTCATGGAAAGAAGCACCATCATCTGTTTGGCTGA
**Promoter (ATG upstream1000)**	
CrLEA1-1	CCGCAACGACAACTATATCTTCCAATCTACTCTAAAAAATTAGAGGCTTTCAAATACGTGGCACGGTAGCA<br>GTAGACACAAGGCAACCTAAAGATTATTATTGGGAAGCTTTGGCTTGCATGAATAAATTAGAAACACTCAT<br>GCTTTAATATTAATTTTTATATTTATATTTATCCTCCTTGAAAAAATGTCTTGCTCTGTCTCTGCTGGTAA<br>TTAAGTACATTTTCATTGTATTTTTGTTGATAATATTATAAATTATTCATTCTATTTTTTATTACTTATTA<br>TATATATATATATAATAAATAATGCATCACTTATTTTGTACCCATCCCATTCATTCATGAACAATAAAATT<br>TAGTTTTCTCATCTTCTCGCTAACTTTTAACTTTTTCTTTTCTTTGAACTCAAAACCATCTTTCTCAGTAG<br>CTATCTCTATTTTTGAATGCACCTTCTCTTATATCTCTCCACGTCTTCTTTCGTCAACTTTCCCTTTCTAA<br>AACCAAGTTAACAATGTTTTTTATTCACAATTTTGTGACTTGAGTTCATTTCAGACGAATTTATTAAATCA<br>CGAGAACTTTAGTATTCTTTATATTTATTTGACAGAGTAATTTTTAAAAACAACGTTCTTTAACATGTCTC<br>AAGCAACCAAATCTTCATTTATCTTCAAAATTTTCCAACAAAATGTGGAGTTCTATTAAATTTATACGTAT<br>TACGAAAGAATTAAATTAAGAAGTTTTAAGAATTTTGAGATACATGAATCGATTCCAACGTTCGTGACAAA<br>ATTTAATTCATCTTGTATTATTATTTTCATAACTAGACACAAATAAACCCCAATACAAGTGGTTGTTTGTC<br>ACGTGTCCCTGATTTCCCGAAACTGCGACGCCGTGTCACTGTTCACACGAGTTAGTTACAAATTGACACGC<br>GGCAATACGTGTCAGATGTAAATTCAATTACCCTCAGAAAATGACACGAGTAACCCCATTTGACACTTGTG<br>CAGAAG
CrLEA1-2	TAATGAAGTTGTAGGAGCAATATTAAGGAGACACTTGCAAATGCATTAGAGTGAAGAAATTATGCAATACG<br>CTATCACTATAACTGTCATCTCCATTGTTAATTTTGTTATTGGAATTTTTTAAATGGGTATCCTATTCACA<br>TTAGTCTTTTAGTTTAGTAATCCATTGTCTGTTCGTTTTAAATTGTGACAATTACTTATACATAAATGAAC<br>ATGTAAGCATTTTTTTTTTTGAATATCTAGTCATCATTGAAGAATATCTTATTAAATGATTTTAAGTTCTT<br>GTTTGAGTAAACTTATATTTCTCCTAAATAAATGTTGATCTAATGAAGTTTGATAACCACAATATAGAAAA<br>TCTTAAAATCTATTTCACATTTGTTATTAGTTTGTGATACACATGATTTCGTATGTCTAACTCAGAGGTTG<br>CTCAAATTATTTAGGAAAGTAAATCATTTATTTTTGGAGGAAATCATTTAACAACAACGTCAGTTTATTGT<br>ATGAACACCAAATTATGTCTGAGGGTTCAAAATCCAAAATAAATTACGATTTAGTCTAAAATGGTTCCAAA<br>ATAAAAATATAAAAATAAAGATACAGAATAAAATCAATATGGAAAATTAATAATAAAAACACAAAAAAAAG<br>AGAGAAAATTTTGGAAAGTTAGTTAAGAGTTATATAAGTTAGTTAAGGTTAGTTAAGAAGTATTAGGATAA<br>AAAAAATAAAGAGCAAAAAGAGAAAAAATAAAAAAGGAAAAATTAAATTTAAAATAAAGAATATAAAAACA<br>TAAAAAATAAAATCATAAAATGAATAGAACCATAAAAACAAATAGAATCAGAAATAATAATAATAATAATA<br>ATAATAATAATAATAATAATAACGAACCAATCAAAACAGAAAAAGAAAAGAAACTGCAAATAAAATAA<br>GAAAGGAAAAGGAGCCCCGCCAAAATTCTTGCCTATAAAAAGGGTTGAAATCTGAAAAAAAAAAAAAAAAA<br>AGAGCG
CrLEA1-3	TTAACTATGATACAAGTTATAATTTTTCTATTTTTTAATAATATTTAACTTCAACTTATAAAACCTATTTT<br>AAATATCTAAAAATAATAATTTTATTTAAAATAAATATTTATTATATTAAAATATAAATTATAATTTTTAT<br>CATAAAACTGTTTAAAAATGGGTTATTTAATTTGCTTTTAGTTTAAAATTTTTAAAAAATTGTAAAATAAA<br>TTCTTAAAAATTGTTTTGAGAAGAAAAATGAATGAAAAATTAAATAGTACATGAAAATACAAATAACAAAA<br>AATAAAAAAATAAAAAAGTAAACAGTTTAAAGTTATTTATTAAAATAACTGTCAAAGATTTAATTGATTAAA<br>TGATTTTTAATTTTTAAATTTTTAAAAAAGTATTTTTGAAAACAAGAAACAAACAAATCTTATAAATTTAT<br>TAAAAAATGATCTTTTAACATAGTACTAAAACAACTAGCACTTGAAGGCGAATATATCTTTTAAAAAAGTG<br>ATAAAAAAGATGAGATAAAACTATAAAATTAAGGTATAAAAATAAAAATTAATAATAAAATCAATAAGTAT<br>TAATTAAAATATACTAATTATATAAATATGTTTATATTATAATTAAAATATTAATTTTTACAATAATTATT<br>TTAAAATTAAAATTAAAATATTTCTTAATTAATTATCCGAATAGAATTTCCATATTAACAATATATTAAAA<br>TTAAACTTTTAATAATCTTGATGTCAAATTTAATTTAATAATCTTTATCTAAAAATAGTCTAGATAAAAGG<br>ATTGAGCGATGATTATGTCCCAGTTTGTATGAACGTGGCGGGCAGTTGATGGGAATAGCTCTGCATGTTGA<br>ACAAGTGGTGCGGTGCCATGCGCTGGGCTTTGTCTTAGCTTGCTTTGCCTCTTCTCTTTCTTTCCCATATA<br>AATCGTCCCTCACCTCCAATCTTATGTCAAAGTGGCCGAACACTCCAACTTGTTCATAGAAACACATAAAC<br>ACAACG
CrLEA2-1	CCAGGGCCCCTCTACCCAAAGTTTTCTCCTTTTTGCAGTGTTTTTCCCCCTCGTTTTTTATTGATGCACGT<br>TTGGCCCCTTTCATTAAAAAAAAAAAAACATATAATAGTAATATGTTAACGAAATTTAAAAGAAAATAAAC<br>AGTTTAATTTTTTTTTCGTGAGTGCATAATATAATTATGCCACGAGTTCGATGCAGTTGATGATAACAAAT<br>TCAACAATTTCTTTTTTAAAATGGTTTTTGGTGCATAAATTCAACAATTTATAAGTCAATAGGTTCATGGG<br>TTTATCTGATTACTATCATCACGGCACCGACATACCACACCCCATAATTAATATGATCGTTAAGCAAGTTA<br>GAACCTTCTAAACAAATAATAATAATAATAATAATAATAAGGAAAATGCTAATGAATTAAAAATAAATATT<br>TTATTAAAAATTATGACTATACACTCAATATAAACTTCATTTATTAATTAATTTGTTTAATATACACCTAA<br>AAATATAAAATAATAATTTATATATAATAAATTTTAAAATATTGAGAACATTAAATATTATAGTTTTTAAT<br>AAAAAATTATTTATTAAGATTTTATTGTATATTAAATAAATTACTTAATAATATTTAACCAACATCTTTAA<br>TTATTATTAAATTCTTAATAATAAAATTTAAATTTCGTTAATTTCATCATTATAAAAAATTTAATAGTAAA<br>TGTTGGGTTAAGACGATTAATATAAATACATAAATAGAAGAAAACCCCTTCCCTATCCTGTGATTCTGTCC<br>TCATACCAAATTATACATGTGCTTATTATACTTTTAATTGAATTATTATTCCAATTAATCCTTGTTTACCA<br>GCACAATCTGTTCTAGACTTGTAGCAAATAGTATTAGTAGATTCATATTCTGTTGGGTGACAACTGTAATG<br>TAGCTGTCGCTGTCGCTGTCGGTTCATTACCTGGTCACCTTCCCATTCACCTTCCTTCTCTTCTCACTCTG<br>AAAAAT
CrLEA2-2	ATTTTATAGGTAAACTTTTATTTTATATATTTAAATTATATAATTACTTAATTTAAAAAATATATTAAAAT<br>GATTGTTCACATAATTCAATAATTAAAATCATAATATATATATAAATTAATACTCATAATAAAATTGAGCT<br>AAATATTTATATTTATTATATATCAAGTATTTGTTTGATTGATTGATTTAGAAGAAAAAACAATTTGTTAT<br>TTATCTCCATAAAATTAGGAAAGAGATTTGTTAAAATGAATATATTTAAATTAGTTTTAATTATAAATTTA<br>TATTTGAATGTGTATTTTTATTAAAAACATTAATTTTGATTTCTCAGTATTAAAATAATTAGGAGCTTAAA<br>AGGATTAATCGATCCTTTGATTTGATGCGACTGTACTCAAACGGATTATTTATATTTTTTTGTGTTTGTTT<br>TGTAAATAATAATAATAATTAGCAATGGAATGGGTACACTCTGCTATAGTTTGGGCGAAGGACCACCGCC<br>CTGACTCCATTTCGTCCACACCACTCCAAACAGCCGATAGTCAAAAAGTGTCCACTTCAAGACAAAATATA<br>GTTAATAAAAATCAACACTCTCATCAATACTTTATTTGTTTATTTTTTTATTTATATAAATTACACATTTT<br>AATTCATCCTTTGTCAATAGCAAACAATTTTATTCACATCCTGCAGACTAAAGGTAAAAAAGAAAAAAGAA<br>AAAAAAAAAAGAATATGAAAGAATTCATTGTGTTTCTATTACCAATAATAGCACTTAATAAAAATTAAAAA<br>AGCAGTAAAAATAAAAAATAAAAAAGGTTCTACCCTATGGTATGGTATGGTCCTACGCATCTTTGTCGTTC<br>ATACCAAAGAAATTTGCAGTTTCTTCCTTTTTCTTAAACCATTGTGGGGTTTGGTTTTTGCCTCATCCTTT

	ATATTATGTGCATCCACATCCACATTCACATCCTGCTCTCAACATAATAAAACACATCAATTCCACCACCG<br>CAGGCC
CrLEA2-3	ACTGAAAACAGCCCTTTGTATGGATCTAGGGTAAAATTCCAGCCCAATGGGCCAAAATGATTGTTATTTTT<br>CTTAATTCAGTAGTGTAACCATTTTCATAATGACAAAATTTTAGATGTAATATTATATATTTTTTGTGTTA<br>TTTTTATTACAAAATAGTGTTTTATTATGGTAATCGACATATTAAATATCAGTATCTATACAAAAATCATG<br>AAAAGCAGTCAATGACGGAAATAAAAACGGCCTAATAAGATCGGTGTAGCATCTGTTTTTGACTGATTCAC<br>CAATTAAGGGAAATCATCGGATGGATATAATGGTGGAGTAAAATTTATGGTAACTGCTAATGTTAGAATTT<br>TACTAGGAACCTGGTCACTTGATTTTTATTTTTTCAACTCCTCTCCCCTTCTGCTGAAGTGTTAAGAAAAT<br>CATTTTTCAAATGCCAAGCTGGGACTTCATGACCAAATCAAATATATTTAATTGTCATACTCACAACCGGC<br>GAAAATCAATAAGATGGTGGTATGAAACTTGGCCTAATTTAATTTGTTATAACTCATTTGCTGATCTATCA<br>TTCTGCATTTCAATATTCATTTTAATGAGGCCGGATGTATCTTTTCCTGCAAGGCTACTTCTAGAATCCTT<br>CAAACTCACTTGTCTTAAGTCATCATCTCCATTTTTAATAGATATCCCAAATATAATTTCATCATTCCCTT<br>CCCATCAATCCAAGCAAGACCCACCCAATTTAGTCAAAGTCAAGCCGTGCCAATACTGGAAACGCGTATAG<br>GAAAACATCAACCATAGACTGACTCCATTCTTCCTTAATGCATATAAAAGTCAAGACACGTAGGTATCATA<br>TCAACGTAGAATCAACCATTTGAAAACATTTCATTATGGAAATGGATCTAAATACCCACTACCCCATTAAT<br>GACTAATAAACCATAATGTATAGTAGCATCAAATGACCACACTAAGTTAAATTGAACCCAACCACCACATT<br>TCAGGT
CrLEA2-4	TTTTTTTTAGTGTACCATGTGATTCAATTATCCATAGTTACTTTAGAAAGACATTTATACGAGGGGGCCCT<br>TTATGTTTCTAGTAATTATCTGCCTCGATGGACTAATTTACTTTCAGCAATAGGCCCCTCTTCGCTTCGGC<br>TTCTATCTGCCATTGTTTCATCTTGTATAACCAACTATTAACAATTGAAAATTCAAACGGAACATAATTCA<br>TAATGTTATATTCCGAAAAAATAAAGTGATTTCTTGCAACTACATGCAGCTTACATCTTAGGTGCTTGAAA<br>GATTGCCTGTGTTGGGAGATCTCTTGTTCGTAAATGGTTAAACATTCTTATAATTCAATTCTTCGTCGTGA<br>ACCATATTATGTACACCTACGAAAATATAAATTGTTCCTGAAGTCGTCATGAACCATATTATGTTGAATTT<br>GTACTTTAAATTTATAAAATGCAAATGATAATAACATTACTCTTAAAGCGGTGAAATTTTTACTCACTGGA<br>AAATTATATAATACAAAAAACTGTTCTGGTAAAAGCTGTATTAAATTTAGTATATAATCGTAATAAACTTT<br>AAATGCAAAGTCAGCTCTATTTTATTTTATAAAATATGAACTAGTAAAAAAGCTTTTATAACTGATTGAGA<br>TTCACACTTGTTTGGGCCTTCATAAACAAAGGGCAGAATTTGCTACCTAAACAGTATAAAGAAAAGGGGTA<br>GGATTTAAGTGATGCAAGTTTGGTATTTTACTTTTTCTTAAATAAAATAACGGCTAGGATTCGTACTTGTT<br>GGGAAGCTTAGGCCATTTAAGAAAATTATACATGCACTAGATCTAATGCTTTGAAGTGAGGAAAGGAAGAT<br>ACCAAACCTTCAAGCCAAATAGTGTTAAAGAACTCCAATTTATTTCCCTCATTGCTCAAGCTTCTCAAGCT<br>AAAAACTTTCCTTCACCCTCAACATTATCAGTAGAATCAAGAAGCATGTTTTAGTCCCTGGTTGATGCCTA<br>TTCTCT
CrLEA2-5	TATAGGTGGGATTTATTTATTTTTTATATATTTGAAATCGTTGTTTGATGCAAGTACCTCTTCACATGTGT<br>GTATCACTTTATTTCAACAACTGAGGTTAGTAAATCCGTTCCAATGTTTTTCTAATAAAAACCTTTAGATT<br>CCATTTATATTAGAGAAATGTTAGTATAATATATTCTTTTTAACATACTAATCAACAATTTTTTTAATATG<br>AATAGTTAGTTTTTTTAATAAAATTAAAATATTTTTAATTTTTAATTAAAATTTAACTAATTATAAGATTA<br>TAATAAAAAATGTGTTCAAGAGGATATATTGTTAATATTCCTCTTTATATTGTAATTGAAATGCAGTTTGA<br>TCACCAACAAAAAAAAAAATCAAGTATCATTGAATGTATCCTGTAAATTATGTAAAAGTAATATCTATTGT<br>TTAATTTACATAAAACAATGAAGTAGGATGACGCAATATAAATATAAAAATAATTTAATTTTCATTTCTGT<br>TAAAAGTTATTTCTTAATTAAGATGAAGTATTTTTACAATATTTTATATTATAATTAAAATAACATATATT<br>TTTCTATCATATTTATATTTTTATTTATTATAACTTTTGTAATTTGTATAAATAACAAGAATAGAATTTCT<br>CTTCTTCTTTTAAGTATCTTATAGTTTTTATATGGTATTTGGTATCAATTATAAAGTCTCAATTGCTAGAC<br>ACACAAAAGGCGGAATGGAAAAAAAAAAAAAAAAAAAAAGGTTCCAGCTTAAGTATACAAGAAGATAAAAAT<br>ATCTTAAACAACTACCATCACATACAAACACTTACCTTTTGTAAGTACCATCACATATATGCATTATGATT<br>GTTTAAATACAAAAGTTACTTAATAATCTATTCATATTCTAAATTTAACGATTGGATAATTTTTTTATTTT<br>TTATAAAAAAATACGAAAAAGAATCATAAATCTATAAATATAGAATATTAATATAGGATGAAAATAAAATGT<br>AAGGTA
CrLEA2-6	ATTAAAAATGTTCAAGAAAATATATGTAATATCTCTTAATTTGTGTACTTGTTACAGAAGATAAAAAATGA<br>TTAAAAATAAAACATGGAGAAAAAAGTGTGACCACAAAATCTTGAGGCTTGTAATTGTCTAGCAGATCATG<br>CTGTCAATTGAATGCAAGTCTTTATTTGCAATCATATATTTGCGTTTCATTTTGTTATTTTTTTAAAAAAA<br>TTATACTAAAATATCGAAACACAGTATTAAAATATCTGTCCAATTTGATTTGAATTAAGTAAAATTATATC<br>AGATGAATAGGCTCTTCTCATCAAAATTTACCAGAGTTAAATTTTTAAGAATTGAAGCTGAAAACTAATAA<br>ATTAAAAAAGTCCATTAATAATTGACGATGGCTCTAATACGATGTTTATTAAAATTGATAATTGATTGAGA<br>GTGAGAGACGAGAAAAAGAATAGAAAAATGGCAAGTATATAGAAAACGTTGGTGAAAGAAGGGATGGAAAA<br>CGAGTGGACCCAATGTTACTGATAATTCACTCAATCACATGCTACGAAGGCAACAAATCCTTCGGAAAGAA<br>ACAGAGTAGAGCGAGCGAGACGTGTGTGTAAGATAAGCAAAATTACCACTGGACACAGCACACAAACCTTG<br>ACTTGGCATCCAAGTTTTTATAAACACCAATTAAATTAACCACCAAAGGTTAGATTAGGATTTAGGATTAG<br>ACAATAGACAGATAGAATAGAATAAACCAAGAAACCCAAATCAAGTAAATGAAAAGAAGAAATAAATAAAT<br>AAATAAATAAATAAATAAATAAAAGAGATTCCAATATTGGTGAGGTGGAAGCATCCACTTTTGGTTCTGGG<br>TACCTATCTCTTCCCCTCACCTCACTCTTCTCTTCTCTTTTCCCTCTACAAAGCTTCATCCTTTCACAACC<br>TCTTCCTCTCACTCCCTTCCCTTCACTCCCTTCCCTTCACTCCCAATACCACCTGGATCTCATTTCCATTC<br>ACCAAA
CrLEA2-7	TAGTATAGATTTTTAAAAAATTAATTTAACTTAAAAAATTTATTTTTTATTTTTTTTAATTAATATATATTT<br>TATTTTTATTTTTAAAATATTTCATAATTTTAAGATTTTCAAACAAATAAAAACATAAAATTAAAAAATAT<br>CCTCTCCATATAATACAAATACAAACAAATAAAAATAGAAAAATAAAATTAATGTAACTTCATAAAACAAT<br>TCTTTGCCCATCATTTTCCTCTAACCAATAGGTAGTCTTAGTTGGAAAGAACTCCAAGATTCTTTTTGGGA<br>ATTTCTGAACCAAATAACAAAAAGGCCTAGAATGTGATATATTTTTTGGGCTCAACCCAGGATGTGATCTA<br>TCATACTATTAACAAGCATACCTCAACCGAAGGCCGAAACAAAAAGAAGTTCCAAGATAGGGATTTTGCTT<br>GTTGTCCAATGTTAGGCACTTAGGCTTGATTTATTCTTTTAAAATAAATAAATTACTCTTAAATGCCATCT<br>TACTTTTTTTCCCCATTAGGTGTGATGTAGAAAATCAAACCTGTAATATGATGGTTAAACTAAATTTTACA<br>CTGATAGCAGCTGAGTTACAAACTGATTGATGGGTGCATGATGATAGCATTATCTAATTTGTATGAGATAT<br>ATGTAAGGGTAATTTTGGAATCCTATTTTTGGGGTTTTTATTTTGATCTTGACGGAGATCCAATTAGAACC<br>CGGATTTCTTTTTTTAACAGAAAGAGTTTGGATAGAATAGAAGAGATGTAAAAATGAATGTTAAAGTGACA<br>AAGTTGACCCGTTAAAAGTAATGATCCCAACCTAATATTGGGATGTATCCTGAGTCTCTCATTATTAACGA<br>AAAGATGTTTACCCATTTCTCTCTGAATCGCACATTGTCCTGTGTCATGGAAATTGCATAGACTAACGCCA<br>CTTTGATGCCCCCATGCAAACAACGTCCCATTATTGCTCTCTAATAACGCCCCCACATTACTGCTCCCCAT

	ACTTCC
CrLEA2-8	AATTTAGTGCTAATTACTTGAAAGTTATTAAATATCGAAGTTCATATGAATTATTAAAAGTAGATTGTGAG<br>TTCAATTCAGTTGGCACTTTATTTTTTGGCTAAAAATGCTCAAACAAATTTCTATCTAAAATATATATATT<br>TTTCATTTTCAGTAAATTTATGCGGACCCAATATTGCGCCAAAAAGAACAGGTTGGATTGGGGACAGTTAT<br>TGCCACAAATTTGGTTCTTAGCATTGTATGGCAGTCTCAATCATCTCATTACACAAAAGTTTGACAGTGAC<br>CAAAGTGTTTTATTGAAACAACCGTAAATACCTTAATACCTTAACATGACTAGGTTACATTATAGGCTTTC<br>TACAGCTCAAGTGTGAACTTTATGTTGCTGTTAATGTGCCGAATTGTTTTCTGGCGATAGGCTAAAAGCTT<br>ATGTTGTACACTGAAACTTCGCTCCGCAATTCATTGCAAAAGATTAAAGAAAAACAAACTTGTTAGAGAAA<br>GTGATTGTGTCTTTAAGGTGCACCTAATTACCGGTAAACCAAATTATACGTTAAAAATAGGCCACTTTGTG<br>TCCCAAAGTGCATGCATATTCAAGGGGCGTTTTCCTTATCTTTTGATGGGTTTTATTGTTTGCATATTACA<br>TTCGTTCACTAATATGTTTTCTTGCATGTTGTATTAGTAAACCATTACTATATCCTTTTGAAAAGAAGGTG<br>AACAAGCCTAAAATTTTGCAATTTTTCAGTAAAATTTAGTGCTGAATTTAACCACCCTAATGAGATGCGTG<br>TGCGTGTGACAATTTTGCAGAAGTTTATCATTTTAATCTTTGATAATTATATACCAATCAACACAAGGGAT<br>GGCCCAGTGGTGCGAGTCAAAAGTATGGTAGCAGGCATAATAATTGTTGCTTTCTGCTCTCTTTATAAACC<br>CCTGACCCCTCTTCCTTAACGTTGTAAACATCCCTCCTTTCAAGCTTCAACTCTCTCTTCCTATCACTATT<br>TTACAA
CrLEA2-9	TAATATAAATATTATTGTCTTACAAAATTTATATTTTTTACTATTTCATTTTAATTTTAAATAGTCTCTTA<br>ATTAAATAATAATAATTTTTAAAACATATTAATTCAATTTCTTAAATTAAACTACATCCTTATACAATCAA<br>ACATATTAAATATAATTAAAAATATAAAAAAATATTATACTAAAAACACATGCTCATCCTCCATCCAAATT<br>AATTTTAAAACTTTAAGTTAAATCTAATTGTTTTTTATTGTTAAAAGAATGATAAAATTTAAATTTCATGT<br>ATAATTTTTCAACTATTCACTACTAAAACAATATTAATAAATTAATTAATATAATCCTTTCTTAATAATAA<br>TAATAATAATAATACAAATCTTAATCTAATATAGAATATTTCTAGGTTAACAAAAAATTACCGATTTCAAT<br>TTGGTCTTATTTAATTTACTTTAATAAAAAAAATTTCATCAATTAATACTTTGTTCATCCATCAAGTATGC<br>TTAACTTATCTAATTTTTCGAACAAACTTTACGTTCATTTCCATCTTCAATTATTTCCTCGTCCCTGGATC<br>TATTCTTCACTAACATAACTAGATTCATGATGATTTTCAAAATTATCGTAATAATTTTATCACTTCAATGT<br>TAAAATTATGTTATTTACTTATATTTTAAACTTTTAGGTGAAACAATTAATAATTCTCCTTTATATCATTA<br>TTTTATTAAATGTCATGATTTGAGTAGATTTAATCTTATCTTAAAAAAGTATCTTAATTAGATTCATACAT<br>CATCTAATTTCTTCACTTTAAAGAAGTTTGAATTTAAAAAATTAGTCACTCAAAATTTTTTATCAAATGAG<br>ACATCCTCTTACAACATATGTTAATCATGTTATTTTGATATATAAGTTGAGTTATACACGTACTTTAAAAG<br>ATTATATAATAAATATTTAAAAATTATTAAATATATCTGTCAATAAATAATATACTTAACTTGAATAAAGA<br>ATAAAC
CrLEA2-10	TATGCTTTCTAATTAATTAATGTGATTGATATGTACAATATACTGTTATTGGTTTGATTGTCAACTTAATT<br>TGTATATGAATCTAAGTGAGATTAATTTCTTTCACTGAAAAGCTGTAAGTTTGATAAGTTTCATTCTTTTC<br>ATCTAGTAATTTTGGTATCAGAAATCAACTAAATCCCTACTAGCTCAAGTCGATTCGAGTGATTTATGTGC<br>ATAAGAGAACAAAACTTTCACAAGCTCGTATACAGTGAAGGACTTCATTTATAAGGCAGACAATAAAAATA<br>TGCTGCGGAAAGCATGACAAGTACACTTTTAATTAAAATGTTCTTTGTAAACTAAAATATAACTAAATCTT<br>TATATATATATATATATATATATATATATACACAATATAAAAATTATAAAATTTGAACTAGAATCCTTATC<br>TAAAATTATTTTAATCATCAATTATTTATTGTTTACCAATCTTAATCATTTATACGTAACCTATTTAGAAA<br>GAACGATTTTAGAAGGAAAGAAGGCAAAAGGCTGAACTTTTATTTTAAATAAATAAGCCTACTTGTATTTT<br>TTTATTTTTTTTTAATTTAATGTATGTGTAATTTTTGTTCTTTTAAGTGTTTTCAACAATTTAAATTTTTT<br>ATTTTTTAAAAAATTAACGAATTTAATCTTTCATTAATTTTTAAAAATAAAATTAAAAATATATAATTTTA<br>AAATCAAGAATAAAAATATTGTCAGCCCTTCTTTTCTCCCTTACAAAACTCCTCCTAATCGATCTTAAAAT<br>AATTTGTAAATGTAAATATTTTAAAGAAAATATTTACCACTCCATCCTATTATATCTGAATAAAATAGTTT<br>GATGATTAATTATAAATTATTTAATTAATTACTGATAATAGAAAAAATATAATTAATAATTTAATATAAAT<br>AATGAATAAATAACTAATTATTGTTAATTAGGCAGTGTTGATTGATATCTACCAAGTATTTTAATTAATAT<br>TCGAGT
CrLEA2-11	TATACCTTCTTCTTCTTCTTTTTGTTAAAAGCCTATTGACCAGCAAAGCTGTCCTTAAAAAAATAAAGGAA<br>ATTATCATGTAATGCCAAAAAAAATGAAAATTATATATAAAAGACATGGTTTGATATTTTGTGTAACTTAA<br>TAATTTTTCTTATTTTTTTAATCTAATTTTTACCTACTAAATACAACCACATCACTTTATCCTGGATTAATA<br>TTAATTTTGAATATTAAGTACTATTTATCTGAAAAAAAATCTTTAGTTATCTATACAGTAATTTACATTCA<br>GTTATATTTGAATGCAAATTGTCTAATTATTAATTTTTTTAAAAAAAATTAACCATTTAATGGATAGAACA<br>GAATAATTTATGCCCTATTCATTTTCTTCATTCAACAATCATTTATGCCCTATTAATTTTTAAATTAAGAG<br>AATTATATTATTTACAATGATAAATAAATAAAGAATATATTAAATATTATAAAAATATATTATTTATGATT<br>TGGAGGTAACTATTTACCAACGTAAAACTTACGCCATCTAAGCTCATTCAAGTCAAATATACCATAAGTGT<br>AAGATCTAATTAACAAATATACGAACATAATGTATGAACAGTAGTCCATTGATTTGTATTTAATAATTAAA<br>ATAAAAAAAATTAATTAGACGGCTTACATTCATATGCAAGTCACTGTATATACCTGACTCAAACCTTTCAG<br>CTATCAATGTACATGAATAAAAAAAAAATTGAATAGGGTGGAAAAGGTTAGTGAATGGCGTTGGAATTTCAA<br>CATTAGAGCACAGAAATTCAAATTGTTGAAATTGGACTTATTGCTACTAAAGTCAACTAATTTTAAGGAAT<br>ACAGGCCACACTCACACTGCTACCTGCACAGTCACACTTCACATCAATTCCTTAGGCCTAAAGTGAATGAT<br>AAATGAAGCAAACCAAAACAGAATACACACAATTTGTGTTCCCAAAACTAACCTGAATTTCATATTCCAAA<br>ACAGGC
CrLEA2-12	ATAATTTTTTTATTTAAAATTGAACTAAACCGTACTGTAAACCTCTCAAGTAACCAGTATTTGATGGTTTG<br>ATGTTGACACTGCCTATTCATTGTTAACAACTATGAAAGAAACGCTACTTAAACTTTCATGTTATCATCTT<br>ACACTTGATAGTTGATACTCCCAACAAGAAACAACTATTTTATTATTTCTAATAGATTGAAATGATTTGTT<br>TCTTTTCTAATCTCTCTAAATAATTAAATGACACGTCATAAAAATCTTTCCCCTTTTAAATTGTAACATTT<br>TATTACTTTTTCTTTGTTGAAGTGGCAAGGAACTGAACTGAACCAGATAAGGGAGGAGTGAATGCAAATGC<br>AATGAGATGCATGCAAATACTCCTTACATATCCATCCACATTTAAGTTACTAACGAAGCCCATCGGCATTG<br>CTTTTTGTTGTTATCTTCATGCATTAGCAATTAAGCCCTCATTATTTCACATAGCTTTTTTATACAACCAA<br>TCAACTCTATTTCGAAAAGAGTTTCTTTAACTCTTCCAATGCATCCAATGATCGACCTATCATCTACAAGG<br>ATATAAATGATTCGCATTGTTAATTTACGTCATTCTTATTCACATACGTAAGTTAGTAAAAGCAAAGAAAG<br>AAAAGAAGCTTGAATAAAGACAAAGCAAGGCATAAAACAGAAAAAAAAAAAAAAAAAAAAAGTAAAACACAT<br>AGCATCATATACATGAGTGAGGAAGTACTTTATTTTGCTTGCTAAAAAAGAAGATATACACGAGTAAGGTG<br>GTCACTGATCAGCCATTGCTCTAATAATACTTTGATTCCAAGTTTCCCCGTTAATTCCATCTAGATTTCCT<br>TTCACTTTTTCACCTATAATTCTCACTACCCTCAAACCCTCTTTCTTTTTCTAGCTGTCACAACATAACCA<br>TATTCATATTCATACATACACACTTAGACATACACAAACCCCTTAAGAGATCAACGAGTCCTCTCACCATG<br>GCAGAA

CrLEA2-13	TAAGCCTAACTTTAACTAAGATAAATTTTATTTAATACCATTGCTCGATAGAAGAGTGTTTCAGAACATTT<br>TCAATCATCCCCAAAGTTGTGAACGGGGAGACATCTGTAATTTCTACGAAACATGTTTGGATCAAGCACAC<br>ATGTCATTTAATTCAACTGAAACAATGAAACAGACTCAAGAACTTCCTTCTTTATTGTTGCTGCTATTAAT<br>TGTTTTTATCCAATCAATGTCGCATCCATTTGACACAAAACGATGCCATTTCTAAGTAAAATTAGTTTCAA<br>CTTGATTCTAAAATCTATGATTTTGTAATTTGATTTCTACCGGCAGATCCGAAAACTGACAAGGCATCATA<br>TACCGTTCGAAACAAGAAGGAAAAACTGAAAAACACACTTTCTTCAAAGAAAATATGTAGCAGAACAATTT<br>TGTGGGTTTGAGAAGGGGTTTAATAAAAAGCTTAAATTGTGAGAAAAGGTCACAACATGGTAAAGAGGTCA<br>ACCATCAGTTAATGAGAAATAATATTTTTCAAAAATGGGAAAATTTCATAGCAGTTTAATGATGAATAGGT<br>AGAAAACTAAAAGAAGCAATGGAAGGTATACAATCAATCTCAGGGAGAAAAATAACAATAAGAAGGTAATA<br>AATCACTTTGGCATTAGCTGGTTGATTATGTGAATGATTATTGAAATAATATGATTGTAAGGAAGTAGAAG<br>TTGCAACTGCTTACCTTGTGTGGAGAATAGAGAGGAAGAGAACAATTTGAGTGTGATTGTGTGTAGAGTGT<br>AGACACAAGTAAAGTGAGTGAGGTTGGGATGTTGGTATCTGTTGCTTTGGTAATTGAAACCCCACATTTTC<br>CTCTTCACCAAAATTCACTATAATTGCATCTTTTAACTTCAATCCACACAATTCTCCTAAACCCCTTTCTA<br>TCTCTCTCTCTCTCTCTCTCTCTCTCTCTCTCATTTCATTCCATACACTAAAACTCCTAACACACCATCTT<br>CCTTTC
CrLEA2-14	TTATTATCACTATAGTTATAAAGACATGGTATGGTTTTCGTAAATAATAACAGGGAAGATTGGTTGTTTGA<br>TAAAACTGAAAAGGGCAGGGGAGAATAGTCTTCTTAAGCGAAAACATGTTATAAAGTCTACACCTAAATAC<br>ATGTTATAAAGTGGAGTAGTTACTAAACTAAGATTTCTGCTATTGATCTTCCGCTTGATGCGTTGGCAAAT<br>ATTGACATGATATATTAAATTACAATGTAGAAAGAAACTGCTGCTACAAAATATACTAAATTAATTAATTG<br>CCTATTGTAGAAACTCATTTGTTTGAAGAATTTAACCATGGAGAAAATTTGTGGTTCTTTTATGTCATTGA<br>GTTATGCAGACTTAATTTATTTTCTCTTACTTTAATTATTTTAATTATATTTCTTTAACATTAAATAATTT<br>AATAATATTTTAAATTTTAAATATGAATTTTATAGAAAAAATAGTTAATATGTATGATTAAAAGAATAATA<br>AAAAAAAGCAATGCAAAGTGATGAAAAAGAAAGCATGGAAGAGTCAATGCCTTCTGAAGTGGAAACTGTGG<br>AAGAGTCCTCGAAGCAGCAGATTGAACGCACAATCATCAATTGAGGATGCACACGCCAAAATGGGATAAAT<br>TGATACATGCGAAAGCAACCGCGTTTTGAAACGGAAAAACTCCGCAGTCCACACCTTGAGAAGCACTTATT<br>CAACACACTTTGCCGTAGCCTCCCCAGTTAATTTTAATTTTGTTTTTGTAGATTATCACGCGCTTTTTCAA<br>TTTCTGCTCCACAGGCCCACACCACGCATCCTCTTATAAATATACTATTCTCCCACTCTCGGTTATAAAAT<br>CACACACAAGAGGAATCTCCCAATCTCTTCCATTCCATTTCTCATAACCTCTTTCGTTTCCTTGTTTTATA<br>ATATTCATATTCTGATTATTATTATTATTATTATTATTATTATTATTGTTTAGCTGGTCACTCATTAGTTA<br>TCAACC
CrLEA2-15	ATATAAGAAAATTTAATTCCCAATAAAAAAAATGGAATTAAAATTACTCACAATCTAAATGTGTGAGATAG<br>TAGAGAAGGGAGGTGTTAATCAAAGATGAAAAGAAGAAAAAAAAAGTTGGAATTATCAAAGTTGGCAATGA<br>GATGCCATTATTAGGTTATAACTTCCATTATCTCATAAACTAAGATGAGGAATAATCCTTAATATAATCTT<br>TTAGAAGGCATGCATTAGGATAACTGTAAAGATTTTGAATTGTCAAGTTTTGTAATAATGTCAGATTTATG<br>TCCATATAAAAGTTGAACCAGAAACAATCCCTAAGAATTACTTTTATATATAAATTTATCAAGTTTATATA<br>TAAAAACATGAATGACGCATTTATATGTATATTTATTTGAAAATGGATTAATATGAAGGAAAATGTTAGAA<br>TTAATTTTAAATATTTAAAATAATAAATATTTATCATATTAATATAAAAATATAATAAATATTAATTTTAA<br>TAAAATAATTTGTTATATTTAAAATCAATCTTAAAAATTGAGGATTTGAATGAGTATATTAGCTAATGTAA<br>TTAAATAGATTAACAAAAGAAGGTTACGTGGAGGCAATGAGAAGATGAAAAGGGCGTAATTTTGGATAAGA<br>TGATGAGAAGCATTAACGATATGGAGAATTAAAAATGGTTACCCTGGCGGCCTTCTATCTTAAAGGGAGTT<br>TTGTTCCGTACGAATATTTCTTGTTCGTGGTTGTTTTGGGCAAAACCCATTCTACAATTTACATAAAAGTT<br>TGCACCAGTCAACACAAGCCGCTATTCCTTTCATAGGTTGAATTTGAATTAGGCAAATGCGGAGGAACACG<br>CATGAGTACTCGCAAGCGCGTTGTTTCCATAGAATCTTCAATGCGCTTCCTTCCTATATATACCCTCTCCA<br>CTCGCTTCTCATTACAATTCACAGCATATTTCCCTTCATAATTTCTTCCTCTTCATTCACTCATCTCATCT<br>ACAACC
CrLEA2-16	TCCAAATGATACATAAGGATAGTGAATTCACGAAAGAATTAATCTTGACAGTATACTTAAAACGAAATTTA<br>AATAAAATAATAATTTAAACCCTTTACATATAATTACAGTATCAAATTTTATTCCTCTGTTCTACTTCTCA<br>CGATTTCACCAGGTATTCTTAGTTTTTTTGCCACCACACTCTGCTGAGAAATGATAACGAACAAAGTCACG<br>AAGCGGGAGGGAGCCACATGCATTTATTAGGTGGCAATTCTAAAACATGCAGCCGAAGAGACATTGTTTGC<br>AAATAACACGCTAACAAGTCAGCAATATTCTGATTCCTGGTTGTAGGCCATGAATGATCAAAACTTCCCAA<br>TTATTTTTGCGTAAGGTTTGTATGGACTGAACAATGTTATGATAAGGGTGGGATAATTGAACACCACTCTC<br>TACGGGCTAGCTAACTGGACAGTTTCACTTTCAATTCAAAACTTGTTAATATCCCTTTCCTAAACTATCCA<br>GAAGCCTCTATAATTCTATCACTATGACAATAATGAGCCAAAGTTATGCAAGGGAAGCTGCTGCAGAGTCC<br>TCAAAAATTCCTTCACAACCAGCATAATTGAATTGGTACCAAAATGATGTGACATTTTAATTGACTTAGGT<br>ACTTTTAATTGAAAAGATAAAGAATAAAACAATAATTGAAATAAAAAAATTTCAATGGTCCGGTTACCACG<br>TATACGTGTCTAGCCAGCAAATTAACCTCAATCGTTAATAATCATGTAATATAATAATGTAACATGATCTT<br>ATCATATCATTTCAAACGTAACTATATTATTTATTTATAAACTTTCAAAGTAATAAAGTAATAAACTTCCT<br>ACCCCCCCCCCCCCACCTTCTTTCCTACTCTCATTTCATCACAAAACCGGTGTCCATCACTTGCGCTCCCA<br>CTTCTTTCATAGCCTTATTATACTCTGTGTGACTCTCTGTTTGATTTAAAAAAAGTCGATCGCACAGGACA<br>GTAACG
CrLEA2-17	AAATATAAAACCGTTTACAAATTTCGTAATAATTTATCATTTTTATTTTAAGATATGGTATATTATTTACA<br>ATAGAAATAAATAATTATTATTTGATTACATATTTAAATAAAGTAAATTTTATATTATACATGAATATCAT<br>AATAATATTACATGATTTAAGAACTAGATTAATAAAATTTACTATAAATAAATATTATTGGATATATCATA<br>TATCCTTTTACTAAATTTGATGTCTAAGATAATTTGATTGTCTCGAAAATTGTTAATGATACATTATTTAT<br>ATCTTTATTATTAAATGAAATCCCGTGTGAAACATATTAAATAACGTATTCAGTCTTCATATGTGAGTCAG<br>TATGAACCATGAAATTTCATCTTAGAAGACCGAAAAGAAAATCTTGCCTGCATGTATCATTAGAGTCTCTT<br>TAAGATTTTAAATTACTGTCCCCAAGCACACCGGAATATTATTTTTTTTAAAAAAAAACACTTAGGAATTT<br>GGAGATCTCAACAATAACAATTGTATCTGCATGAATGTATTTGCCTACAATTTTCTACAATTACAACCACA<br>ATTCGAAATCTTACTTGTGTGCACAATTCAAACCGGTTTAAGATTATTTTTGAAATGCAACCTCTTTTCTG<br>ATTTTTTTTTAAATTATTATTGCTCATTCAATTCCGCATGACAGTAAAAAGAAGTGAATAGAGAAGCCGAA<br>ATGAGGGAGAAAAAACGTAGAAAGTTAGGAATGGAAAAAATTAAAAAAGTAAACGACATTGGTTGTGAAAG<br>TGAGGAGAATTAAAATGAGAAGCTGGGGCCTTAAACTTTAGTCGCAAATCAGCGAATTCTATTCCAAGTCT<br>CCTTTATAACTTTTATCTGCTCCACTCCATATCCATTACAGTCTTGCTCCTAAGTCCTATCCCGCTGTCTC<br>CTCCAATTTCGTTTTCTCTTCTTCACTACCCAAATCCTCTTCTGTCTTCTCAAAGTTCATACTTGCCAATA<br>CCCAGA
CrLEA2-18	CATGATGTTGATTTAAAAGATAGAAGCTTTTTTTTTTTTCTGATAGAGTAGACCCTTTTCTTTATCAGTTA

	AAGACTTTTCAGATACAGAATTTAAAAGTTTCTTTTGTTGGATAAAGATTCACGAATCTTTGTATATGAGA<br>AGTGTCAGTTGGGTTTATTTTATAAAGTATATTTTTTCTATTAAAATAATAAGACGTTTCAAGATGTTTTA<br>ACACATTTATATTTTTTGTTTTTTTAAGTGTAAAAATTATATATATTTTTTTATTAATAAAAATGTTAATT<br>AATCATTTTATTTTGAGATTTGATTAAAAAAATTATTAGTATACGTTATTAATGCATTAAACATTAATATA<br>ATATATTATCAATTATTAAAAATTTTAATTTTTATACATAAAAATCTATATTAATCCTGAATAAATTTTTG<br>TTTGTTAATAACTTTTTTTTATAATGGAGGATCACAACGTGTGTTCTAAAAAAATCTATTGCTAGCCTGTT<br>TTCATAACTGATAATATATGATGTAGAAATTGACTTCTTTAACTCATTCAGCTTTTGGTAAAAAATTCATA<br>ACCGAATAATTTTAACATAGTTTTTGCAACATTATCTTTAATATAAAATAGTGAAATTTATATGAATCTTA<br>CTAAAAGATGAAAAAGGAGGGAGTGATTAGATATATTTGGTGGAACTTAAATAAATTTTACAATAGAGATG<br>TTAAGAAGGTGTATTGGCATTCCTCCTTGGTATATATCTGAAGGAAGCCATGGTCTTGACTCTTGTGTATT<br>TTAAACATAAATAGTTGCTTCCCGCACAGATTTTCCCCTCAAAAGGTTTTGGTATCAATAATTTAGAAAGG<br>AATAATTGAGCGCCACTGTCAAAGAAAGACATCCCATTCGTCCTTCGCATTCAAGTAGTACCACTTAATTA<br>ACAATCTAAGCGAATCAAACAGATTGCATTAATTACACTCGAGTTAATTAGCCGAAACTCCAGAATGATAA<br>TTTGTA
CrLEA2-19	CAATTCAGAATTTAATGTGTTTTTTATATTTAAAGTGTAAATAAAAACACTGCCTAATAGTCACATTTTTC<br>TTTTGGTTTTTTTTGCATTTTTTAGTTCGTAACAATGACATTTGCTTTTACACTAGGAGAACCATCATTTT<br>CGGTAGGTATTATCTATTTTATCAACACGGTCATGTTAATCGCAAATACTCAAAGGATCAATTGTGGTAAA<br>GAGTAATTTTGATATAGTTCACAAGGCACTGTCATGTATAAATATTTTTTAAGTTACTGTAATTTATTCCA<br>AAAAAATAATGTATATGATATTTATTTATACATTAAAAAATAATGATTGATTTATATATAAACATCACTTT<br>ACATTCATCTACATCCTAATAATATTTTTTTATTTTTTAAATAAAAAAATTATTTAACCATAAATAGCATG<br>AAAATAAATATAATATATTTTTTAATATATGATGATCATTTTATCTTAATTATTTATATATATATTTTTTA<br>ATTACAATTATGTTATTAAAAGAAATTTCGTATTCATTCTCACATTATTAAAAATATTATATTAATATATC<br>TTCTTTCACAATTTCCAACATTATTTTTGAATAAAAAATTAACTTTTGATATGTTGGTTGGCGAGAGGGTA<br>TTTAAAAGTATAAATATTTTGCAAATGCAGTAAATGAAAAGTTAGATTGAATATGTGGTGTGAAACAGTGG<br>AAGGCAATTAATGAATTGTTTTGAATTTATGTGAAATGCATGAATTTAGAAAGATGTGTTTAGTAGCAGTT<br>GGGGACACGGAATTGGAAGGCGCGCATTAAGTTGAGAGTATGCACAATTACTAAAACGATTGGACCCTCAC<br>AACAACCAACCAACTTAGGATATTAACATTATTATTATTAATATATGGTTGGCACTTGCATATAGGTAATC<br>CATAGCTATCAAATCTCTCTTCTAGATCTAATCTTCACACTTGTTTCTGTTTTGCTAGTTTTATTGGGTCA<br>GCATTA
CrLEA2-20	TGTCTCGTGGTGTGGTGTACCATGACACAGTACTGATTCTTGTTAATAATTTGTTAATTAAGACCCCCAAC<br>AAGAATCAAATGATTGGCCTGTGCATCAAAATTCTTTTCTTAATATATCTGAATAAGGAAAGGAAGAACTT<br>TTTCCTTTTTTTTTTTTTCCATTAGGTAACATTTAGTACAATAAATATTTTCATATTTTTGAAAAAGCAATG<br>TGTAAATAAAACTCTTTTACTTCTGTACGTGAGAAAAATTTGAGCAATAATTGATTATATAATTATTAATT<br>TTTCATGACAATTAAATATAAATATATGGATATTAGATATCAAATTTAGGAAGATGATATCTTTAATAATT<br>TTATACTAAAAGTAAATTTTATACGTCTATCTGTTAAATTTAAAGTTTTTTAAATAATATATATTTTTTACA<br>AAATTTATACTTTATTATTTTATTTATTTAAATGTATATGAAACATTACTTAAAATATAAGTAAATAATTA<br>TTATCAAATTATATATTTAAAATGTTTAAATTACGTAATTTTATGTATATAATTATTAAAGATGTTAGTTT<br>AATAAAATTGATACTTGATATATTTTATCCGGACTTAAATATATTATTGTTCATAGAAAAATCAAATAATA<br>ATTAAACACATTTTTAAAAAGTTTCTAAGAATCCTATTTTCAGAAATCTCACTTCCAACAAAGCAGTCTAA<br>TTGGAAATGAAATATTTTGTGTAGCATTTCAATAAAATAATTTTCATGAAAGGTTTGTGTGGTTTCTTTAA<br>TTCCCGGCATTCCGGTGGTTGTTATAGTCCCTCAAAGTACAAACATAGCATTGATGTTTTAAGCCAAAGTA<br>CAGGTTCTTCCCATGGCATAGAATAGAAAACACCCTATAGAACAGGAAATATTCCAACTCAACGCTCTTTT<br>GGCACTGCAAAAATATACAACATTTTTAATGGCCGATTTTATTTGTTGTTTAATTACTTATTACTTATCCG<br>ATAATA
CrLEA2-21	TTAATTTAATTTACTTCAAGTATTAACTTGATTTAAATGTTAAATATGATTATATTATTTCAAATTTAAAA<br>ATATATGCTTTTAAAAAATATATATTTTTATTAAAGTAAAAAGAAATATATTTTTAAATTTATAATAGTAT<br>ATTCATATTCAAAATTTAAATTAATTTATATAAAATAAATAAAAAATTAATATATATTAAAATAAATACATT<br>ATCTGTTAAAATAAATTAATATAATAAAATATTAAATTATTTTTAAATTTTATAAATATAATTTATATAAT<br>AAAATTTGTTAATTTAAAGATTAAATTATTAAAATTTAATTATTTAAAAAAGATATTAAATAAAGTAATTC<br>ATAATAAACTCAATCTTAAAATAAATTAATTCCTCTACCTTTTTATTTATATATATATATATAATAAAAAT<br>AGAAAAGATTTGATCATTTTAAAAAAATTAGAGAATAAAAAAATAAGGATAATATTTTTATTTTAAAATTA<br>AAATAATACATATTATTTGTGATTATAAATAAATGAACAATAAATTAATTTATTATTTTATATATTTATTT<br>TAAAATTTTATTAAATTACTATATATTAATTTAAAATAAAAAGTTTATAATTTTCTTTTTTTTTTTTTAAG<br>ATTCCTTTTTCGAACAACCTTTAAATGGGATAATTGGATATATGTTTTTTAAAATTTTTATGGTAAAAAAA<br>GATTTTTATTTTCTATATTTATTTACAAATAAAAAAATAATAGTTTATAATTAAGAGTTAAAAATAAAATA<br>AAAAATCTGTTTCTTTTACTGTTATATATAATTTTTTAAAAATAAATAATACAATATTTTTATAATTAATA<br>ACAAGAGAACAAAATATAGAATATATTCTCTCTTTATTTTTGGTTTTTTACTTAAAGTAAACTCTCTCCAA<br>GATTGTCCAAATGGGCGGTACGGTATGACCTGACACGCAACATAAAGTCAGAAAGTCGAAACGACGGCGTA<br>ACGATC
CrLEA2-22	ATATTCAACAAAATTATTTATACAATTTATTTTATTGAGTGTCAACACAAAAAAAAAATTATTAAAAATATA<br>ATTTTAAGAGATTTAATCTTTTCAAAAAAAAAATTATCATAGCATAATTTTGTGATATTTTTATATTTATTA<br>CTTTTCATAAGATAAAAAATACTTGTTACACCTTTCTCTCAAATTTTAGCATGAATTAAATTCAGTTAATA<br>TAAATAAAATATCATATATTATTTTAATAAATAGTGTTGTATCATACCTCTTAACACTGAACACTGTAAAA<br>ACCAGTGTTTCAGTAGTTCGTCGTTCAATCAGAACTTGATCACCGAAATTAACAGTTGCAAAAATAATAAT<br>ATAAATTATTAAATTTGTTAAATTGAAATTAATTAAATCTGATATCTATTATATTATCTAAAATAAACTTC<br>TACCTTTGTGACTCATGTGAGTTGATTAAATTCAATTTATTTAATTGTTTCTCAGTCTCATTTATAACTTT<br>TCATTACTTTTTATTCAGCTTTATTCTTTAATTTTAATCAAAATATTCAAATATTGAAGATATAATTCACA<br>ATAAGTGTTACAATCGTCCTTTTATAACTGATATCCATTCAAATCTTTAATTAAAATTTACAATCATTATT<br>TTGTTGTCCACAAATTTATTGTCCTAAATCAACGATATACTTTTTAGATTTAGTCACCCTAAAAAATTATT<br>TATCATGTATTCTATTGATCTATTATTCTATTATAATACACAACACAAACAACATTTGCAATCCATAAATT<br>CAATTCCATCATATACATATATCATTACCAACGTATCTCCTTTAGATTAAGTAAATCTATAAAATAAAATA<br>AAAAAGAGATATATTATAAAATTAGGCCACAAGATTACGTTTATAAATTGGCTTATATTTTCTATATTTAA<br>ACCTGGTATGGTTGGCTTCACTCTAAAAGCAAAGAATTTCACATTGTGGGTGCATCTTCAACTGCGTCTGC<br>AACATC
CrLEA2-23	TTTTTTCCTATTTATATCAATCGAAACAGTTTATTTTTTTATTTTATTTTTATTTTATTTTTTTATCCTTCA<br>ATTTTTTTTTTAATTTTTTTTTAATTTTTTTCAACAAAGTATAAGAAAAAAAAATAACTGCATCCAAAGTAT

	AAAAAAATATTTGATATTTATTTTAATGTTTTGTTTTGTTTGGATCACGGAAAAAATAGAAAGAAAAGAAA
	AGTAAAGAAAGAGAAAAAAAACTAAAAAAAAAATTATTTGAACAAGTAGAAACAAAAAAAAAAGAGAAAAA
	ATAAATTTTTTTTTCATTTGAATGAATGAAAAAAAATATACATATATATATTAGTATTATAAATTTTATTT
	TATATAAATTTTTAAAATATTTTATTTTTGTACTATATTTTTTTATAAATATAATTTATTAATTTTTTATT
	TTTTTTAAAAAATAAATACTAATTTTAATAATTTAAAATAAATAAAAAAAAAAAAACTTCTCGCTCACTTTT
	TCACTCTTCATGACGAAGAAAAAAAAATAAAAACATGTGAGTTCTGGAGATATATATATCCACTTGGATACC
	GATCCTCTCATATACGCGTAGTTGGTTAGATATTTATCATGTGAAGTTGCGGAGAAAGCCACTATATAGAA
	TTAATGGTAAGATCTAGTGGGCAAGAAAGATAGATACTATGTCTATTAATAATAGGGAAGTTTTCACTTTT
	CACCAAAAAAATCAATTAATTATGATCTTTGTGGACGCAAAAGTCATTACGGTCTGACGCGTCCGTACCCT
	ACTCGTACACGTTTTGGGTCATTCTCTCTTTCATACACAATTCTTAGCTGTTAGCACCCGTCAACTCCAAG
	CGAAAATTGAATTAGGCAAAATTGACGAACAATAACAAATAAGCACAAAATGCTTACTATATTTACATACC
	CCTCCTCCCATCACTAAACCCACTTCATTCATCCGCTTCTAGTTAAAATCTACTATTTTTTCTCTAACTGT
	TATGTT
	TCCATTACCTTTAAATTTTAGAAATACACATTAACATATATTCTTTTTTTTACTTTTATTTGAAAAGTATA
	AACTATAGTGTATAAATATTAAAAAAAAAATAGTAAATCCACTGTGTTCGTAATTTGTGACAAACGCCAGAT
	AAAATGTAAGAGAAGAAACGTGCTCTTAATAAATGTCGGAATTATTGATCATTTTATTTATTTCCTTTGGA
	AAATGATTTAAAAAATAATTTTCTGGACATAAACTATCCGCAAGTTAATTCGCACCACGTGACAATAACTA
	CCGAAAATAAACTGTAGATAGTAAATCATATTTTCGTTTTATATAAAAGTTACGTTCATAAGTTTATAATG
	AATTGTCATTAGCGAGCGATCCATGTCATATATGTAAAAGTGGGTACTATATAAAGGCGTGGCATACAGTA
	CCATCACAGTAAGAAAAGAAGCATCGTAGCTACGTACTTATATAGAATTTAGAGAAAATAACAAAACTGGC
	AAGTTAAACTTGGAAAACGTTATGCTTTTGTTTTGTATAAGTAGTGGGACACAGGCGAAATGACCAAACTC
	ACCCTTCCCAAATTTTCACAAACTTAAGTAACTTCCTCAAGAGCAATTTTTTACAAAATAAAAAAATAAAA
	TAAAAGTGATTAAAGGATACTTACAATAGATAAAATAAAAATTTTGATAATGTGGATGGATGTACGATGAA
	AGCAGCCTCCACCGTTCACATAGCATCCCAAGACTTGAATATAAGATGATCTTATCATCATATCCTCCGAA
	TACAACAGTTGAACCTACAAATAGATTTAACACCAAAGGCGCACACACATTTCACAGAAGCGTCAACTACA
	CGAAACCTCTCCACCCATTCCTTCACCTCACTCATACCAACCACCATAGCATAGTTACACGCTCCTCATAG
	ATATCACTCTCTACTTCAATCTCACACAAAAGCCACCAACACAAAACAAATTAAAAAAATAAAAAGAAAAT
CrLEA2-24	CAGAAG
	CACGAGGTCGGCATTTTAAGATTTATAAATTGGTGATACACACACGAAATTCCCAATACTGAAATCTGAAG
	TGGCCCCAGTTTAAGGCAATTATGGTCGACACAATAAATTCAAGCACAAGCGTAGTAGTTGTACTGTACTG
	AAATCTGAAGTACTGAATAATTCTTAATTAAGATGGGCATGCATTACAATTTATAAACCCTCAGTGGATTG
	GATTTGTAATTGTATACATTCTTTGTTTGGTTTCAATTTTAAGGAATGTCCTATCACCCAATTAAGTAAAA
	AAAATTGCTTGTTTATGTTTTTCTTAAGACCTAGTAGTATTTACCAGTGTACCATAACGTCTTGTTGTAAA
	GTATGCGAATCGTAGCCTGCCTTAATGTGCAAGTAAGGTTTCCTTTATTGCAAAGCAAGTTCAAGAATGTC
	AGTACATCTATCTCCTCATTTTATGTGAAGAATTACAGTCACTTGCCATGCAATTGTCATAGTTGAAATGA
	CGATGCTTGGTGCTTAACGCATTGTTGTACATCATCTCACACGGGAGTGAAGTGGCAAAATCAGTAATATG
	TAAAATTTCTAGTAAGACTAATTATATAATTAAATAATAGAACTGTACAGATATGAAGTGTGTATAAGGAT
	TAAGGAGCATTGTTCTCGTAGCTTAATGCCAACGATTCTGTCACACCGACATCACTCCAGGTGTCCTAGGC
	TCAACAAAGGAAAATGTAGAAAGGAACTCTTGTCTCTGACGCAATATAACAAAGTGTCAATATTTAGATGA
	ACTACCACTCTCTATTTCGGTAAATTTACATGTTCTTTGTTGGTGGGGAAGGTAACCACACCACAGTACCT
	TTTGAGAGACTGAACGATCCATCTTCCAAATTTTTATTTTCACATTTAGAAGTCAAATAAGGAAAGTCTGA
	ATATAGCAGCAAAATAAAAGGCCATTTATCACCACAAAATTTGAGAGGAGAGAGCACTCAGCAAATCACTT
CrLEA2-25	GTATTC
	TAAATTTGTAAATCATCTAATACTTAATTTGGGCCTACATGGTGACTTGGGAGTTAGGCAATGATCTAAAT
	ATAAAATTGGAGAAATATATGCATATGGGAACTTAACATATAATATGTATATCCTTGATCTCCTTGCCTAG
	AGTTCGTATATATACTTTATGCTACATTATACGAAACCTTTATACCTTATTCCCATCTGAATAATTACCCA
	TCATTCTTTAATTTATCCTTCGCGATATATATAAGATGCATGATCATGATATAGAACCTTATTATTTCATC
	ATTAACCCTTGGCAATATATAAATAAAGTTGCTGGTCTCTATCTCTGCATAATCACAGTACTTTTAATTAA
	AGAAGTTTAAAACTTACCTAGAGTGTTAATAAGCAAAAGCAATCTATTCTCTGATGTGAGTCTAAATTCTG
	AACACTTTTACTGACGAAATACATCTAAAGGAATAAACCTTTGGGCCTTGATTAAGGGGAAGTGAGTTCAT
	ATGAAAGTGATTGCTTTTCAAATAAAATTTACATCTAAAGTGATTACTTTTAAAAACGTACAAAGAAACAT
	AGTTCATACGGAAATTCGCACCTTACTTCTGTCAAGATAGTTAATACTGCGTGATGCATGCTAATCTTAGC
	TGTTCAAAGTCTTTATGTGTACGCCAGCATTTTGAAGATAAGGTTAAGTGTTACATTGTTGGATCAAGTAG
	CTCCATTAAAAGTAACCAAGTGTGAGCTATTATAATTCCTCAAACGAGATGCAAAAGGAAAAAGCTAACAC
	AGGAAGAAAAAAAAAAGTGTTTTCTTTTCTTAATTTCGAGGATATGCAAGTGCAATACGATAGAGAAAGCTT
	ATTCTTTTCTTCGTCCATGTACCATACTCAAAGTCTGTTCTTTCCTTTTCTTTCCTCTCTGCAACTTCACT
	ACATAACCGCAACCACAACCACAAGCGTAGTTCATTTCTCAAACGCATCCATTATTTCCTAAATCACACAA
CrLEA2-26	ATCAAA
	GATAAAAATATAGAAGAAATGATCTATACAAAATTAATTTTACATATTTTCTTAGAAATATAAAAATTAAT
	ATTTCTTTTTTTATCTATCTATAAAAATAATATACACATAATATTAAAAATATAATTAATATTATTAAATT
	TTAAAATATTTATTTTTTAAAATTATTTTTATATATAAATTTTAATTTTATCTTTCTTTATTTATTTCGGT
	TATGATGATATATATATATAAATAATATTTTTTAAATAAATTAAATAGATAAACATAAATAAATTTTATAT
	TATCCTTTTGAACAAATAAAAAGATATATATATGAAATATTATTCAGACATTTTTAAGATATTAAATTGTT
	TTACTCTTATTTTTATAATTTAAAATAAAACGGGATGTAGAGAATTTTTTTAAAAAGGTATTACTTTATCA
	GGAAATGATATTGTAAAACAACCAAAACTATTCTACACATTTATCCACGTATTGAATATATATATATATAT
	ATTAATTCTAATTTTTATAAGTTCTTTTTTGACCAATGAATAAGTGAGATAATATTAAGCCGAATAGCCAA
	AGAAAGTACTTCCTTCCATCACTTTCGAAACAACAAAATACATGTTTGCCAAATTTTATGGAATTAATAAT
	ATCATTTAAAATATTTAAAATAAAACTTTATAATGAACCAATTTTGTATTTAAATTTTATGGAATAATTAT
	TTGAACTTTTTAATTTAAAAATAAGAATATGTGGCTCTTAATTTTTAAAAAACTTATTATGGTTAACAAGA
	AATCAAATTATTTAATATTTATAGCTAAAATTAAATTAAATTTAAAGTTTTAAAAACGAAAAGATTTAGAT
	ACAAAAAAGATTACCTAAGAGTGAAATATTTATAATAGTAATATAAAAAAGAAATGGGAAATCTAGTGGCA
	TCTGACAAAGTCCAACCTCCCACATATTAATTACTTGGCACGAGCAAAAGCTTCGTAAGCAGTAAAGAAAG
CrLEA2-27	AGAGAA
	ATATTAGTATTATGGTATTTTGATTTGAGAGTCAATAAGTTGAAGTTCAATTCGGATTACACAAAAAAAAA
	AGTTCAATTTTCGATTGAAATTAATTTTAACGAATTTATACTTTAAAAATTAATTTTAATATATTTTTTCA
CrLEA2-28	AAGTAACTTTAAGATGATGAGTTATTTTATTATAATTTATATAACATATATATTTTTTATTTTTTATTATA

	TTAACTATTATATCCTTTTTTTTTGTTTATTATATATATTTCAATTTGGTTAGGCATTGTTATATTCTTTTA<br>AATATCATAGAATTCTTAATAATTATTTATTAATAGTTAACTTTTAATTAATTATAAAATTATATAAAAAA<br>TATATTTATATTAATCTTGTTAACCAATATTTTTAAAATACTAATTAAAAATAATTTAATTCATTAAAAC<br>TACGTTGATATAAATTAAAAACATACTAAAGATACATTAATTATATTTTTAATAAAAAAATTTATTTATTT<br>AATCAACGCTCTTAAAATATTGGATAACATATTTCTATTTATATTAGCATTCTTCATTTTTAAAAATATAT<br>ATTATAAATATGTCAATTTTTTAATTTTCATTTATTACGTCAACAAATTAAATTAGTTATCTACTTTTATA<br>CATTATTCAATAAAGTTCAAATTCAAATAAATAATCGTATCATACAGATAAATTATTTTAATTTTTTTCCC<br>GGAAACCACAATATTTCATTATTTTGTTGAAGATAATTTTATTTGACTAACAAACATTAAATTATGATATG<br>AGCAAACTTTGGAGTAAATTTTTTCATATAATTTTTTTTGTAAGGTCCAGATATCTCCTAAAAACATACAA<br>TTTTTGTTGGAATAAAAAATAAGTAAGAGGAATTTTAAAACAATAACTATTTAGTGTGAATTAACACACTG<br>TATAAACACTAAAAATTATGAAATTGTTTTTGAGTGTTAATTTATAATAGGAGTGGTATTATTTTATCCTT<br>TCATTT
CrLEA2-29	ATCTTCATGTTAAATTATACTAGATTAATTTTAATGGCTTAATTTAGTTTTTAGTTTTTAGTTTTTAGTTT<br>TTTATGCTAGTTTTTAAACTTATCAAAAAAATTGATAAAGATGTATATATAATTTTTCTGGTGAAGAATAC<br>CTTTTTTAAAAAATATTACGAATGATGATGTTGTATTAAAAATTATTTAAATTTATGATCCAAGAATGGGA<br>TAAAAAAAATCAACCTTGTAATTTGACAGTGGTAGTGTGTGTCGTCTTTGTTATGGTTTAATCAATTTTTT<br>TAATGTATATTCCGAGATACATGATAAAAAATTAATAAATAATAATTTTAATATAAATTAAAATATTTAAT<br>ATTATCTATATATTTGATCTAATTATATATATATATATATATATATATATAGAGAGAGAGAGAGAGAGAGA<br>TTTTTTACATACCTTAGAAAAACGTAGTTTAATAACTATAGTGTGACTCTTAATACTCCATTAACAGTAAG<br>ACTGTCTAATTCAAACATTGATTTATAACCAAAATGAAATATGATAAAGGTGGCATAAATGTGATTGAGAA<br>AACATGAATAAGTTAGTAAAATATTTCCATCTTGACGGGTAGCCATTAATCTGTCACATTATGTTTCTATG<br>CATCAAAATGGTTAAACCATGGTTGCTAGAGTCTATATACACACTGCGGTGTATATTACTCCTTACCCCAT<br>CCAATTTGAATCTACATAAGGGGCACTTTATACCGACAGCTATATTACTTGCGATAGTATGTTACTATATG<br>TAACGTGACGCTTGAATCTGAAGAAAAGAACAAAATATGGTGTCCCGAAGAATCCCATAGATGTCTTGAGT<br>TTCATGCACGAAATTCTGAAAGCGAAAAGTGGTATTAAAAAAAAAGGGTTTGAAAGGTAGTTATATATAGT<br>ACATAGCAATATATAAGGATGGTATGATTTGGTTGTTGAATAAAACTCTACATGCCCTGAATAATATTAGT<br>GTTGAA
CrLEA2-30	GTGATGATTGATTTTTCAATATCAAAATAAATATAACTGTGAAAATAAAATAAATTATGTGCAAGAATAAT<br>TGTGTTATAAGAATATACATTATATATCATTTATATTTATATTTATATAAAAATAATAGGAAGAATTCTAC<br>TTGACAAAATACTTATTAGTAATTTCGAACCCCTTCTACTTGTCTCGCATGTCTAATCTTTGCCTCTCTGT<br>TTTTCAGTACATATACAATACACATTGTAATTTCTCCTCCTAGGATGCTATACGAAAATATTTAGAGGCAT<br>GGGGAATCATTTGAATATATCTTTTAGCTTTTGGTACTTCAAAGTAAACCTTTTTTTTTGCCTTATCATTG<br>TAAATAATTCAGTCAGCAATCTGCTTTTGAGGACCTTATCTTAGCCAAATAGAAGAATGAGCTATAATTAT<br>CTTAATTCAATCCGTGCTGAATTCTTATGGTTGAGATTCGGCAGAAAGCTGGCCTAAACAATTGATTGATT<br>GCTTTTAATGAAACATGCACCTTTTCCGGGTGGGTGCACTTTCTGCCTTCTACGATTTCTTAGTGCTTAAA<br>CATCCAATGTAGCACATACCTGGTTTACTTTAATATCATGGGAGGCGTTGTTGAAGCCCTTAATCTAATTC<br>ACTATCCTCCAATACATGCACCATGTAAAATTCATAGCCTTAAACAATAAGCAATGAACAAAGTGTTGAAC<br>TCATTCTTTAGGACCTTCATAAACTTAGGCGCATACCAAAAGCATGAACATTAAACACCAACGTGGCACAA<br>CTTTAGTTCACCTATAACACCTTTTGACAATACCCAGATAAACACAGTGTCCCCACTCCACTACTTATTAA<br>TTCAAGTAAAAAAACCTCTTCTTTTCCTCCTCATTTCCTTTCATCCATCAACACCCCATTTAGAATAAATA<br>AAGATATCACCACTATCTCTTCCTCATCACTTTGGTTGATCGGAGTGGCCTTTTTTTAGTAGTGTGCCTAC<br>TCAAAC
CrLEA2-31	TTATAATATGATAAAATATAATATATCTTAGAATACATCGTAATTTATTTTATTATGATGTTGTAATTTAA<br>TTTTTGAATTATTAAAATAAATATATTAAATATGATATAAACTATTATGTAACACTTTAAGTATCATGTAA<br>ATTATTATGTCAGTTAAAAAATATCTGAATTTAATTTATTTATATCAAATCTATCGTCATAATTTTTATTT<br>ATCTAAAATAATATAAATTAGTTTATATTAAAAAAAATTATCTTTAAAAATTAGTCTATCTACAATTTAAA<br>TTTATTTTGACATATATATTATATAATTATTTTTATAATAATAATAATAATATTTTCTATAATATAAATAA<br>TTATTTTATTTTATTTTTTATTTTTATTTTTCTTATTTAAAAATAAATATATTTATTGTATAATTAATTTT<br>ATATTTTTAAATAACTTTTATCTGTAAATTTTATTCAATGTTAACTTATGATCAGATTAATTAAATTTTTA<br>TAATATATATATATATTTAATTTTTAATCCTTCTAATATTAACATAATCTTATAAATTTTCATTTTTTATT<br>ATTTTATTAATTTTATCAAATAAATAATAAAATTAAATAAAAATAAGTTAAATTATTTTTTAATATAAATT<br>TAAGTTAAGTTAATAATTAATTTCTAAAATTAGATGAACATTTAAATAAAATATACTTATTATTTTATTTC<br>TATAATTTTTAATAATTTTATAAATAAATATTCATATATTTAAAATTATATAAATAATATATATATATATT<br>CATAAAATTATTAAAAAAATACAGAAAAAATAATAAATTGGATAGAAAAAATTATCTTATCCTCCTATCCT<br>ACACATTAAACCAGACACAAAATAAAATAAAAAATTATTCTGTCCTATTCTCATACCCTGTATCATCCGAC<br>GGCTCTTAAGACTATTTTCTTCACCGTTAATAACGTTAAATTTGAAATTTTGAAAAGTATTGATAGCAATT<br>GAATTT
CrLEA2-32	TTTTATATCTTTTATTTATTTATTTTTATTTCTATCGTCTTTATCACAAGAAATATTCTAAAATAAAATAT<br>AAAAACTTTTATAGTAATAATCAAATTTATAATTAAATATTAAAATAATCTCTAAATATAAGGAAAGTAAT<br>ATATTTATATATCTATAGTGATTTATATCTATATATATTTTAAATTTAATTATTATAAAAAATAATTTTAT<br>AACCATAACAAAGGATAAATATGGATATAATGGGTGTATGAAAGGAAGAAAATCATTTATGCTGAATATAA<br>AACACATACTAAAACCTATGAAAAATACTCTACCATGCCCATAGCCCGTGGACAATGGATGGACCCTCCCC<br>GGGTAGTACAAAGGAAGAGAATGAGAATGTGCCCAAACTCAAAATAAATTAATTAAAAAAAACATGGGACT<br>GCTGAGAGAGAAAATTATTTATAATATTTAGAGCAAGTTGTGTCTGTTTGGCATGAGACTATTTCAGGCAT<br>AATCAAAAGGCGGCAAGATACAAATTTAGGAGGCCTTTTACTTGAATATTAGTTTTTTAAGCAAAAAGGAT<br>TAGAAAACGTATTAATTTTGTGGAAAGACAAAGGCATGTTGAAAAGATAATTTTATTTTCTTTCTATATTT<br>TACTTTCTCATTATAAACAATAGATGACTCTATTAAATGAAGAGATCCTTCGTCTTATTGAAAAGGAAAAA<br>ATGATGTATGTAAAAAAGAAAAAAAGAAAAAGAGCATATAGGCTTGGAGGCCAATGGAAAGAAAAGAAAAC<br>CGTGCATGGCAACTGGCAAAGCGGCAATCACAATCCCGTGTGCTCACATCAAAAATCCAAATGGTACAGCC<br>AAACACTCCTTGACCCGTTCGCTTTAGTCTCTGTTTCTTCTATAATTTTTTTCCAAACTTGCCAACCTTAA<br>GCGATCCCACATTGAACTGTACCCCACTTCCCCTTCTTTATTTATTACTTATTCCACTATCCATAACATAT<br>TAACAC
CrLEA2-33	ATTCTTAGATAAATGTGTGATTTGTCATTTCATCTAACAACTATTCTTAAACCTAGCTGAATTTTGTTAAA<br>ATATAATTTTAGTATAGCTTTAAAATAATAATAATAATAATAATGATAAATAAATAATTTGTTTAGTATAG<br>TGATAGTATAGCTCTAAATCATCCAATTTGATTTATTTTTTTGTTGTATCTGATTTCACACGCCATTTATT<br>CTTTCAATGGTTATTAAGTTTTTTACAACTTAATAACTATAAAAGAGCAATTGGTTTGTGGATACTTCTAT

	ATAATAATTTTTAATTAATATTATTTATACACATTTTTATATACCTCTCATCGCACTTTCCTCTTTTTTTC
	CTTTAAAAAACTCTCACTTTCTCTCTCCCTTTAATAAAAAGAAAAGTGTAAGGTGTTTAAATAAAAGATGC
	AAGGTAATCAATTGATATTACTTAAGAAATAGTATATTAATTAGCCACCTTTTTTTTATCAGTTTTTAAAT
	AATAAAGTATATGCTATTATATACTTATGAAATACACGGCACCATAAGAAAAAAAAAAAATATCGTCCATAG
	TTTTTTATGTAGCTGAGTAATTAAATTAAATATAGAGAATAATAAAAGGTCTAGAAAGTATATAGCATAGT
	ACGTCATGCTGCAAAACCTTGAATGGAAAAGCAAGAAATTAAAGAAAGAACCTTCGGCGGCAAAACATTAT
	ATCTTATTTATATAAACACACAAATATTGAATATGGTCCCTTCGTATGACATGTGATTGAGAACAATACTC
	AAACTTTAAGATAGTTATACTTTTGGCCATTCAAGTCAACAAAGGGAAGCTTCAAGCCTTCAACGTTTCTT
	ATAGGCCATGACTTGCTCAAATCAAACGTGTTCATGTCAACCTTCTATATATTAACATACCATAGAAACCA
	CATAAAGGAGAAAAAAATAAAAGAAACCAACAATTAACTTTTTTCTCAAATTTCATTGGGATTTTCTGAAA
	CCAAAG
	GTTTACATTTTGTCTTTCTTCGACCTACAACTCAAATTTTGGCACTATTATCAATGAGAATTGGAATTTGG
	GTTGCATTGATGGAGCAAATATGTAAATTAGTACAAACAAGATATATATATATATATATATATATATATAT
	ATATTTATGTAAAAAATAATACATGATAAAATACAATGTCAAAGGTAACGATAACAACATTAAAAAAATTC
	ACAGAGATATATTATTTGAATGATAAATTTATATAATAATTAATACAACATATTCTTTTTAATTTTTCTCT
	ATTACGTTTTTTATTAGCAAATAAACTTTATCTATTATATTATTTATCATATTTTACATTTTCTTTCTTTC
	AATCACTCTTTTATTAATTTTGTACATATATTTGTAGTATTAACATTTTTCATATTCAAAATTGATTCATG
	GATGTCCTAGACTTTCATCCAAATATCATATGCATGATAAGTAAATTCTTAGTTATGTTCTATAATATCAA
	GCAAAGCGTGAACATCAATGTGTAAGAAAGAAGTGAACAAACACGAGATCGAGATGATATCAACAATTTTT
	AATATACTCAAAATTTTATAATTTTAACTTCTCAACAAGGCTTCTTGTTACCGTTAAATTAAGGTTAATTG
	TCCACCTACTTTTGATGAATACATCATAAAATACAATGAAATAATTAAAAATAAATAATATAATATAATAA
	AAAGAAAAATAGAAAAACTGATGAAGAAAATGTAAAAGAAATTAGGTGTATATATAATGATTAGTTTTGAA
	GATTCTTCAATCTTCATTACTCGGTTTGTAAACCCAAAAGTCAATGCCTTTGTGTGCCTGTATAGTGGAAG
	GAAGTCACCTAGAAAAAGCGCAAACAACACCAACATTCCAAAATCATCAACGATTAACCCCTACAAATAGC
	CACATAAAATGTCACACATCAATCACCAAGAGCTTCTCCTACCAACAATTACACACACCACACAAATAAGC
CrLEA2-34	ATCACC
	CATAAGGGACTGCTAGAAGTATTAAAAAAATATAAAAAAATAGTCTTCATAACATTTTAAATTTTGATTTT
	ATGATATTTCCTACATCTAGATCATCATGTTAATTAAAAACTAAAAAAAACTAGTTGAACTTTTTATTTTA
	CAATTTTTTAATTTTATACTTGGTTTTAACTCAAATTGTATAAACTTTTTACTTGAGTAGGCATCATCGTT
	AAATCCTCATCCGTAAATATATATATATATATATATATATATATATATATATATATATGATTTAATTTCCGTT
	TTGAAACAGTTTTAATAATATAGTAATAATTAAATAATGATACTAAATATTTATATTTTGCTAAAAATATA
	TTTTTATTATCAAGAGAAGAAAAATTAAAATAAATTATTTGTATATTTATCCAAATCCAATAAAATTGGAT
	TTCAAATTTTATACTGTATCTTTTAAATATGTTTCAATTCTTTGTAGAATAAGATGAAAAGGATAAAATCC
	ACCCTAAACTCATCCTATTATCATTATGATTATACTTAAATGAGTAAAAATAGTTTAAAACTTAATTATTT
	GAGTAATATTATATTTTTTAAAAATTTTAATACTGTAATATATATTTATATATATATTAAAAAATTTATAA
	GAATCAATACTTTGTTCATCTAACTAAAAACAAATAAGGAACTCTTTCTTGTACCTTCAAATCTAGTAGTA
	ATATTTTATACTTGATCTCCACATCCAAATTATGGTCTCGTTGTTCCTTGTTAATCAAACAATTGAAAAGT
	TCTTGTATACTCCGCTTCAAAAGTCAATGCCCCCCTCCCTCTGCACCTGTATTTTGCAAGGAAAACTCGTA
	GAAAACACCATATACCATACTCCAAAACCATCAATGCTTGGTTAATTACCCCTACAAATACCCATAAAAAT
	TAATGTTACACCATATTAATTTCAATTTCATCAATCACCTACACCTTCTTCTATTCCTTACAATCAATCAT
CrLEA2-35	CACACC
	CAATTCTCTTCACAATTTTATTCCCATCTAAAAATGATATAAAATACTGTTAATGAAAAAAATCTCATAAA
	ATATGTTCCTAAATTAAATAATCACTTAAAAGATATTTTGAAAAGGATTTTGAGTTTAGCATGAAAAATCA
	ACAAAAAAAAGTAAGAATGTAGACATATAATGGTGTTGGGTATAAAATCTGCTTAAGACCAAACAATAAAA
	TGACTTAAGTCAAATATTAAATTCATTCTATACTCTCTATACAAAATAAGATCTAAAAAATATGACAAAAC
	TATTCCAAATTACGATAGCTATTCAGATATCTCTGTGAAAAATACACAAATTTTAAAATTTATGTACTCTC
	CAATCTACCTTGAACATGACATGAATTTGAGGTTGAAGATTTGTGATAAAACAACCTTAAATGTCAGCTAA
	GAGAAATTACAATCATTCACTATCTCTAAATATAATAGAAAAATAATAAAATAAAATATTATTATTTCATT
	GACAAAAAGATAACAATAGTATTCACAAAAGAAAACAATAATAAGTAAAATACGTTTTTGAAACATGAATA
	AAATCATATGAAGCAAAACAAAAATAAAAAACTAACCTAAGTTCTTTATTCCTATTTCCTAGCTAATAAAC
	AAATCATCTGAATTATAGTTTATAAAAAACAAATTATTGATATAATATTTTTCAATAATACTTTTATGACA
	TTAATTTTTAATTATCTTGATCATATCAATAATCATCTCATTTATTCTTTTTATTGTATAATAATCATTTA
	CAAATTTATACGAGATTACGCGGAGCCTGTAAGATAGGACAGAGTTCCATTCTCCCCAAAGAAAATATTAA
	ATTATGGGTCCCACGGTCCCACGTAACGGCAATTAAAAAATGGTGACACATAAGGGTAGGCATTAATTAAG
	AGTAAAGAAAAGTCGTAATTAATTAATTAATTAATTAGGAAATGTTAAGTTAAGCAAGGATTATTATTAGT
CrLEA2-36	TTGATT
	TCCATCCTTTTGAATGGCACTCTTAAAAAAATAAAATCAGAAGTATATATGGATGTTGGATGTATAAATAT
	TGGTGTCCAATATTATAATCTTAGATGAAATATTTTAAAAATTTTGAAATATTTTAAAATTTAAATATTTT
	TATAAAAATATTTTAATTAAATAAAAGAATTTAATTTTTTCTATTTTATAAATTTTTATATTAATGATAGA
	TTTTAAATTTATTAAATTTTAATTGTTATTTATATAAAATAATTTAATTTTATTATATACAAATTTTATTT
	TTAAATATTTTTAATTTTTAATTTATTAAAAAATTATATATATAAATAATTTTACAACATATTTAAATAAA
	TAATAGTCTAATAATTATACAAAATAAGTGGGAGATTAGATTGCAATGAAACTTCCATTAAAAGTATTATA
	TTGAGAATCAAGATGGAAAATAAAGAGATGAAAAAGAAAGCACTTAAAAATATAATAAAATAATAAAAGGA
	AAAATATCAAAAAAGAGAGTTGCATTTATGAAAATGAGCCTTAAAAATTTGAAAAATTTTAAATTATACTT
	ATTTTAGGCAAAATTTAAATTGTTTCAATTTAATTTATTATACTACTTTCTTAAAATATCAAGAATATGAT
	TTTTTTTAAATATGCAAACAAAATATTGTAATATCTAAAATTTAAATTCTATTAAAAATTGTTATTTTTTC
	TTAAGATGACTTACCTAAACATATAAGTTAAAGAATTATAGTACAAGTTCTAAAAATTGTTATCATTCCTT
	TCCCATTGGCGGCCCATAAAATAAACATCTGTACAAGAATTATCTTATCAACATACATCAAAGGGTATCTC
	ACAAATAACTATTTGAAGGTTCGATAACTTTTTAATTTTTTTTTCAAATTTTAAAAAGTATTTTATGTAAT
	TTAATTCTTCTGAACGACATCGTATTTATTTATAAGTATGAAGTGTCTGTAACCGTACACAAGTTCTTCAA
CrLEA2-37	GCTACA
	TATTAAAGCTTTGTTAGGATGATTTTTAATTTTTAATTTTAATTTTTTTAGAAATTTAATTTAATTTTGTT
	TAAAAACTTGAATTGTAATTTAAAATTTTTTAATTATAAAAGTGAATTAACTTATTTTATTTTAATTTTTT
	ATTATAAATTTATTAAAAAATAATTAATTAAAATGAAATAAAAAATAATAATATTATTATTATTATAAAAA
	TAATGATTATGATAATTATAATAGTTGTGACATCAATAAAAATAAAAATAATGATAGTAGGATAAAAAAAC
CrLEA2-38	GGGAATAATAGTTAAAATTAATAATAATTAAACTATTATGATTAATAATAATGAATACATAATTAATTAGA

	TTAATAAAAATTAATTCAATATAATTAAAATATTTTATTCAAATACATTGTTTCTAATAAAAAATATCTGA
	GGTATAGACAACAACAAAGCTTGCCATTGGACTGATATAAATGCTGAAATGGAATTCGATACATCCAATGG
	ATGCATTCATTATTTATAGACTTCTATTGAGACATTATTTATGGAGGGGGTCTAATCATATCGTTCCGTTA
	TTTATGGAATTTTAGTATTTTAAAGTGATTTAAATAAGTAAATAAAATATTAACACAGTATTAGCATACAT
	AGCAGTGAGTAGGCTATCTGTTTGATAATAATTAGTACTGTTAGAACTTTTAATTTACTGCACTAATGTCT
	GGCTACAAAATTTGCACAAGAAATTTATTTCATTCATTTTCATTTGCTTGGTCTATTCTTATCATATCAGT
	ATCTATAGTTGCTTTCTAATTTTATATATACCTCTAATTTGTACACATATTGGTATTGATTTGTTGTCAAG
	GAGTTTGTTCTCTCATTAGGAATCAGTTGCCAAAGTTTTAGGCTACATCCAACGTTTGATTTTGATTTCTG
	CCAACTGAACCCTCAGAAATTGAATTTTGAAATCACACGGAATCATATCTATACAACCCTTGTCCTCACTA
	GTCACT
	TATATTTAAAATTTCAATCTTTTCATTTTAAATTAAATTATTAAAAAAATTAACCAAAATATTTTTATTTA
	AAAAAAAGTCAAATCCAACACTTAAGTCAAAATCGAATAACCCGTTCATCGTAACCCGCAACCCATTAAAT
	CTAATAAACGGCCAGAGCTAACTTAAATATGGATGGAAAACCGACTCTAAAATTTCTTATTTAAATTTGCG
	CTGGTGTTACCCGAACCCACCCGCCCGACAAGCAAGCCTAACACCCGAACCCACCCGCACCTGCGCGACAA
	GCAAGCCTAACTTCTACTATGAATGCACGTTGTACGGTTATTCACGGCAAATCCAAGCACACACTTAAATT
	TCATTTGATTTAAAGTTTGATAAAATTAATATTAAATAATTAAATATAATTTAAATGTATGTAAATTATTA
	CGAGACACATATTTAAATATTGAAACAAATATTTTTTCGGTATTTATTTTATCTAAGAAAAAAATTAAATA
	TTAAAAATATTATGAATATTGTTTTTTTTTTTTTAAATTATTTTTTTTTATAATTTATTATAATTTTATAA
	ATAATAATTTATTGCCGTTAATAATAACAGGATTGATATAAGGATAAGTTTTAAAAAATTAATATTTTTTA
	AAAATTAAATAAATAGATAAATAAGAATAAAATTTTTATTTTTTTACAGATAAAAAACGAAAAAAATATTC
	TTAATATTAGAGCGAAATTAATTTTATAATTTCAAACAGATAAGAGCTTAATTAAGAAAAGTTTGTTTTTG
	TTATAAAATAAAAGGCCAGTTTCTTTCTTAATTTCCCCCTATCTCTCTCTCTTTCTTCCCTTGATTTTTGC
	CTATATGTAATGCAAAGTTTCTACCGGAAACATTCTCTAAAGTGGTGAGTAGAAGGTGTGTACAACACAAG
	CATAACACACACATTTCCCTTATTAATTTGGGCATTACATACACTCATACACCCCCCACCCAATCAGTATC
CrLEA2-39	ACCATC
	ATTATTTTTATTTTAATTTTATATCTTTCTCTTCTGTGACATAATATTATCTTATCAATACGAGAGGATTC
	AAATAATTTTAATAAAGAGATTATACTTTTGGACGTAAAATTCAAACATTTTACTTTGAAATAGATATAAG
	CATTTCTTTCAAAATGATGCTTACGTCAAAGTTAAATGAAATTTTAAAAAAAATTATTAACTATGGTAAGT
	TTAAAATTATTTTAATTTATTTATTTTTATATGAGCAAGAAGTTTAAAATACTAGGTTAAGTTGCACGTTG
	TTGTAAGTGTTAACATGATTCGGAATTTAGACATACAAGGAACTGGTGTAATAAAATAGGAAAGGGGAGGA
	GAAATTCAGGAAAAATGTTTCATAAGTTTATTGATTTCTGCTATGTGCAATATAGAGGCGTTTTACCCAAA
	ACTAAACACAAACTCTCATCTATCCATAGACACATCGGGCCTTAGGAATGCTTGAGTTCAACAAGAAAAAT
	ATTGGTTCCTGCAACTGCAATGTGATATTTGTCTATTTGGTTGCTAATGGAAGTGCCAAAATGAATAGATA
	CATACAATTTTGGATCGGGTATTGAATCAAAGAAATATATGAATACAAGGTCTCACTGCAAGGATGTGTAT
	ATATAATACATTTTCTCCATCCTGATTATTGGAAGACATGGTTGGGGTATACAACTTGAGCATCAAATGTT
	GGAGACAAACATCCCTTCTTTACATTTGGAAGCATGATTATACTTTGGACAATGAATGCTGCAACTCTAAT
	GGAGGGTGCTCTTTTTTTCTCCGGCTTTTTCACTGGATTTCCGGAACAAAAGTTAGTAGTGATGATGTAGT
	AATTGCAAATGGAGTGCAATCTTTCAGCACCAAGAAGGTTCACAGCAAACTTGCAGCATAGGCTCGAAAGC
	TAAGTTTAAATGAGTCCCACATTGAATACATTACACAAAGTAGTGAGGAGGATGAGAGTATAAAAATAAAG
CrLEA2-40	CGAGGA
	AGTTGGTTTGAACGCAAAACCATAAAACTTTATAATATCTTTATATTTTTTTAGTTTATTGTCAAAGTTAT
	ATTCAATATCTGTTAAAGAATGATAACTTTTTAATCATATTCATTAAATATAAATTTTAACTTGTTGAATA
	TATTATTGAAATATTCTATCAATTCAATCGTGTTTCATAATAACTATTTTTTTCGTGTGACTTAATATATG
	TAGACATTTTTATTTTTTATCACTATTATTAAAATTTTGAAAAATTAAAATTAACTATTTTTTAAAATATT
	TAAAATCATATTTAACCTTAGTTTAGAGGATTAAAATATGCATATTTTATTTTATTTATTACTTAAGATTA
	TTGCACGCATATTAACATTTTAATTATTTGTATTCTTATTGTTTTATTATTTAACATTTTCTATGAACGAA
	TAAATTAATTAAAAAATAACTACTATTTTAAATTTTAAAAAAATAATTAAAAAGGAACATTTTTTTAAAAA
	AAAAATTGGCAAGCGATAAAAAAGAAAAATACTTTATATTACTTTATGAATAACATGAACTTATTTCATAA
	TATAAAATTAAATATATTTTAATTCTTCTAAAATTGTAGATATTTAATTTTAATTTTTAATTTTTAAAATA
	ATTTTTTTTAGTTATTATTTTTTTATCATTAAAACGTTTGATATATGATATATTTTAAATATTATTTATTTT
	AAAAATAATTTATTTTGAAAAATAAGAAATTAAAATAAAAATATGTTTAATTATATATTTTATGTTATATG
	CATAATATATTTTTAAAAAGTACTATTAAAATGGTATTGTTTAAAAAATGTAACGCCATTTCTCAAATCGG
	GTAAAGTACCGCCTGGATGATGTCTTTTCCCGTTTGGGTATAGATGAAGGGAATTATTTATTTCCAGAAAA
	GAAAAATATTAATAAGAAAAGCATTGACATTTGAGGCACAAGTTTGAGTTTTAAAAAATCTCATTTATTTA
CrLEA2-41	TTTAAT
	GAGTGTCTCACACTCAGACTCAGACTCAGACTCAGAGTTGGTAGCCACCATGAACACCCTTGTTCGCAACA
	CCACCCTCTCCAGAAGCCGCAAATACAGATATCAACCTCTCACACTACCTAAGTCTATTAGCTACAGACGC
	ACAAGGGCTAAGCAAAGGAAAATCTTCCTCGCAACCTACAAGTTATCTTCCATCGATTCTTTTCCTCCATC
	CCCTAAATCCCCTAAGCTCAACAAGGTTCCTCTCAAGCTCAAGAAATTTGTAGCCTCTGTCTTCCACTTCA
	TGCGAACCGCAGCTTCTTTCAGATCCAAGTCTTCCATCTCCTCCCACTTTTGAAGGTTTTTCGGAGCCACC
	TTTCCTGTGTTTAGGATTACTCACGTTTATTGCTTCCCCATGTTCTTCTTTTCCAATCATTTTTTTTTATA
	AAAAAAAAAAATACTGTGTATGGTTGATAATGGAAAATTGTACCATACTATTACTGCCATTTATTCTGTAT
	AGAATATGATGCTTAATCTTGCTCTATTTTCTATGTTTGCCGTATTAGTTTTTCATATGATTTGTCTTTGA
	AGAACTTCGTGATTATGATTACTGCTCTTTAATTTTCTAGTAGTCCAACAAAATAAATAAACTGAAGGAAA
	TATATATATATATATATATATATATATATATATATATATATATATATATATCCAAATTTTAATCTGCGCATTA
	AGCTTACCCAGCTGATTATAATTTAAGATAAGATAGCAAATGCAACCTGAATTAACGGGTGCCTAAATTTA
	GGAGGGGGAACAAAAACTGAAAGAACCAGTTTGAGCCTCACAAAAGGTGGTTAGAGAACGGTGTCCATTCA
	TACATAGCAAAAATTGTACCTTCTATCTGTCCAAAAGATCACGGAGAGGGAGATGAGAAAGAAAGAAAACT
	TTTCAATACTCTTGTCCTAGAAATTAAGTAACATGCCCCCACAATCTGAAAGGGTTGATTAATCACCACCA
CrLEA2-42	TTCATC
	TTCCCTTTCTTTAAATCTTCCACACAACACAATTTTATTAAAATTGTAAAATGATAAATAAATTAAAAAAT
	ATAATTTGGTTAAAACTAATTTTGTTAAATGTGTTTTATCTTCTTAAAATGAAAAAAAAAACAAAAAAGAC
	AAAAGTTTCCTTTTTAATTTTAAAAAATTGTTATAGAACAAATCCGATTTTAAGATATTAGATAGTATACA
	ATTTTAAAAATTTATATTATAATTAATTAATGAATTTTTTTAAAAAAATTAAAAAAGTATGTTATAAATAA
	CAATAATTGTAATATTAACCATGTTTAATACAATGATATTAATAATATATAATTAATAAATTTCTATAATT
CrLEA2-43	ATAGTATACATATTATAACAATATAATTAAATACACTATATATAATATGTACAGTATTTAATGTATATAAT

	AAATATATAGAATGAATTTATGTTGTATTTAATGCATATTATTATAATATGTATATATAATCATAGAATTT
	TATTGATTTTACATTATTAATAATATAGTATCATTTGTGATTTATTTAATTATAATTATTTATAATATGTA
	ATTATATCTAATATAATATTGAATTAATTATAATTATTTATAATATAATTATATATAAGATGGTATTACAA
	TATATAATAACTTTTATGTATTATTTTCAGCATAAAAATATTATAATATATTAACATTTAATTTTTTTTCT
	ACCATACATAATAATAAAACAATGTAAGCCTTTACTCCAAGTGTTTCAAACGTATCTCTTATGAATCAAAA
	AATTTAAGAATAATATTGACTATTTATGAATAATTATATCATAAAAATTAATTATTTTCATCGTTTATAAT
	AACAAATTTCTTTCACCTGATATACCTCTTATCTATATATATATATATATATATATATATATTTCAAGTTA
	CAATGATTTGCTTCACGAACATTTATCACACATATATCTCAAAAATTTGTAGGTTGATTTACCCAAAATCA
	TAAACC
	ACAAATTAAAAATTACAGTAAGTATGATTTTTAAAATATTATTATAAAAATTAACAACTTTTAATAATATG
	AAAATTTTTTATTGGATGAGAATTTAAATTTTTTTACCAATATCATTGGATTCCAATTAAATCTATTATAT
	ATTATATTAGATGAAATTAATTTATTTTAAAAATAATTTATGTTAGAAATACTTTATTCGATAAATTTTTA
	AAATATATGAATTTAAAATTTTTAATTACTAAATAACAAATTTTTATTATATAAATAAAATTTATAAAATC
	ATTTAGTATTTTATTAAATAATTTTAATTTAATGTATGATATTTATTTTAAATTAAAAAATAACGACCATA
	AAATTATATACCTATAATTTATTATATTTTTTAAAAATTTAATATACTTGATTAGTATATTAATATTAAAT
	TATTCAATGATTAAACATATTAACTTTACATTTTACACAAAATAAAATAAAATCCAGTAGTGAATTGTCTT
	TGCTTATCCATAATACACTCCTAAAATAATCTTATCACGTCCATGTATTTTTATATATTCTTATATTTTAT
	AATTAATCTATAATTATATATAGCCGTAATTTCATCATTTTAAAACAGTTTATAAAGTAACTGCCAAATTA
	ATGTAACTTGATGACCCATAATACAGCATTTCATTTGTTCATGCGTTGTGTTCTAAATTAGGAGTCAACTG
	CGGTACCGTTATTGACTAGTATTCAAGAAATTTTCAAATTCGACAATCTCCCAGCTGAATCAAATTTACAA
	ATATCAAAATTCACGCAGTCACATGAGGAATGCAAAATCGGACAGAATCACAAATTTCCGAGTATATATCA
	GCGCTAATTATTTCTTTCCATCCAGAAATTAAGTCTTGAATCGACAAAGATATAACATTATTCCATACTTA
	TATCAAATTTTAATTAGTTGATTTTCCCAATTAATCTCCATTTACTTTTATTCCCTGAATAGCCAGAACCA
CrLEA2-44	GAAACC
	CCAAACAGAGCCTTTTATATTATTATTATTATTATAACAGAAACTTGGAAGTTTATCTCAGTATACTAGGA
	AAGGAAGTGAGAATCAAATATACGATAAATTTCAAGTATAGTATTTTAGGTGTTTTGAATATATTTGCACT
	AGTAATTTGGTTCTTATTTTTATCTATTTAAAGAAAGAAGATAAACATTACTTTTGTTTATCTATTTATAT
	AAAGTTTAAGATAATACTAATTTTTTATTTTTCATTAATATTATTTTATTTATGCCAAAAATAAATAAAGA
	GAAAAAAGTAAAAATAGAACAATCAAATAAATAAAGAATAAATTAAAAAAATTGTTCAATAATTAAATTAT
	AATAATTGATTTTTTTTTGCATAATTATTTTCTCTTTAAATAAATAAGAAGAGAGGTAGCATTTCAATTAA
	AATTAAGATTTTACTTTAAGAAAAAATTTAACTAATATCATTAAAGCATTTATTAAAAAGTTAAAAGTAGA
	AAATTTTGTTAAAAAATTAAAACATTATTAATACACAACCACTATTATTAGTAATACCTCCTAATATTATT
	TTCAATAAAATCTTTCTATTTTGACCTCAACAACATTAGTTAAGAGTACCCTTGCTTTAATAATTAAGTTC
	AGGCATTAACACTAGGCTTTATTACATGAGTTATGAAAGAAAAAAGCTCGAATTTTAAGTAAAGAGCAACA
	GCAAAACAGGAAGATTAGAGCTAAGTTTTGTTGGTTAAATATAAGTATAAACAATAATTGAAATGGAGGCA
	ACTTCCCGTACACTACAAGCGTGGCTAGACACAGATCAGAAAACGAAGTAAGTGATGGTGTGATTGTGGAA
	AATAAAGCATTACATTTTCTCATGGCCTATATAATGCATATCACATACAGCATTGTTTAAGTAAGAAACAT
	TCTCCAAATTGGTGAATCCATTACACGCAAAGCAACACTGCTTTGCATTCATATATTCCCACATAAACAAT
CrLEA2-45	TAACCA
	CACCTAAACTTCATAAAATATTTGCAATTTGCTCATTCAACTCTCTTCTTCATATATAACCATGTTTGGTT
	TTGTTCTTTATTTTTTGGTTTAGTGTTTTAGGTAATTTTTAAGTTGTTGCATTTTTTAATCTTTGGTTGAA
	ATTTAAAAATAATATAATGTGAGGTAACTCAAAGTGAGTTTAAATTGACAAGTATTACGGAAGAATACTCA
	CTCTTCTACCTGTACTCAGTACTCACTGGTCAATTGATAAGGAATATGGGGATACAACTCATATACCCTCC
	TAGAGGACAAATTTCATGGAAATTTTATCTTTTAAAAAATCCGCTAATAATAAGTATAAAATTAGGAAAAA
	TACCTAGATCATTATCAATCAATTAAAAGAAAAAATGATAAAATTGGAAATTAATGTTAAATTCATAATAA
	AATTATTAGCAATTTAATGCTTGAAACTAATACTTTGTTTAGATAAAAAAATTAAAAAATAGATAACAATT
	TATTTATTTTTTGCTTTTAAAATGAAAAAAGTAATTTTTTAGAGGTAAAATTTATTTTTAATTTTTTCTTT
	CTTTCTTTCAACCAATCAAAATTTAAAGAATGTTGCTTTAATAAGTCTTTACGGAAAAAGAACAAAAGTTG
	CTTTATTTTGTTAAATCTCAATAAAAAAGCTTGAATGGACAAAACATAGATTAGATTTTATAAATTCTTTT
	ACTGTTATTCTTTAATTAAATGAAAGCTGTATCTATTAATGACATTCATTTTTTTAAGCATAACCTTATTT
	TAATTAAAAAAACTATAAGATTTGACCAATTAATTTTGTGGAATTTTAATTTGGTGAAAGAAAACATACTG
	AGCTTAAAAGTCATAAGAATGATTAAGGAGACGAAGAATTTAAGAGTGAGGAGCATTTGCATTGTGTACGC
	GCGGAACGTGAGAGGTTAGAGCTGGATACAGCTAAAAGGTTGTAGTTAGAATGTTGCCATCTCTCCCCCTT
CrLEA2-46	CTTAGT
	TTATGTATAATTTAAAAGAATATGTTATGTTATATTTTTATATAAATTAAACAAAGTTTATGTAGGATTAA
	AAAAGATAGATATTTTATATGTAATTTTGTTTAATTACAAGAATGGATGAATGGTTAGTGTAATTTTTTAA
	TAAAAATTTATAATTTATATTTGCAATTTAACGATTGAACATTTTAAAAAAAAATATCAACTTACATCTAC
	ATTTGATGTAATCCACTTCTAACGTGTAAGAGGAATTTTTTCCTTAAAAAAAAACTCTTTTAATACTCTAT
	CTATTTTTTTTATCGGTCTAGAAAGAAAAATATAGAAATATTAAAAAATACAATTAATATTATTAATTTTT
	AAAAAAATTATTTATTTTTTAATTTATCATTTATATATAAACCTTTTTTTTACTGTTGATGGATATAAAAA
	TATATATATAATAATAATAATGAATATTTTCTTAAAAACTAGAATACACAGAAAATATGAACAATTTTTTT
	TTGTACTTTTTTTCTATAAAAATGAAAAGGAACGGAGGAGAAATATATTAACATTTAATTAGAAAAACAAG
	GAAAAAGTAGACAGAAATGAGTCGAGAAAATTTTATTTCACATTTTTTACATTCAAACTGTAACCAATTCT
	GTCATTTGGCTAAACAAGTAACAATTCAGGATTTTAAATTCCTGGGTTGTGTTTCACCGTTTTGTTACTAT
	CTTCTTTGATTAGCAACTAGGCTGCGTCTATCATCCATAATCTACATTAATCCAACAGAACTAAACGTCAC
	GAATAAATTATAAAACGTACCTAAAATGATACCAAATAAAAACACATTGAAGCATAGACAAACACAAGAGG
	ACCCATAATATAATTAGATCAATTGTCCAACTCCTATAATTGAAGGTTCAAGAATTATTATCCTGGAAGTG
	CTCATGTCCCACAAAGAAAAAGGACAAACATAATAAACTGTTATTCTTTTATTTGAATACTTTTACATAAC
CrLEA2-47	AAATAT
	TTCTCTGCAAATTTAGAATTTTATGTGTTATTTTGTAAATACATTTAACCTGGTTTTATTTATGTAAAACA
	TTAAATAATAATAGTTAAATTAATATATGATGACAGATGTCTGCACGTTATTGGTTTTTGGGTGAAGGGGT
	ACCAAACCCCGTTATTTTCAGGGGTACCGGAGAAACCACCATTCTTGAATCGATCACATATAATGATTAAA
	AAGTGAATTGGTACTGTTTCTCATAAAATTAATGGCTTTTGAATAATAAAAACAAATAAGTATTTAACTAT
	AATTGTATTTAAAAAGTAAAAAATATTTACGATTTTTGGGTGAAGGGGTACCAAACCCCGTTATTTTCAGG
	GGTACCGGAGAAACCACCATTCTTGAATCGATCACATATAATAATTAAAAAGTGAATTGATACTGTTTCTC
CrLEA2-48	ATAAAATTAATGGCTTTTGAATAATAAAAACAAATAAGTATTTAACTATAATTGTATTTGAAAAGTGTAAA

	AATTTATGATTTTCTATGAATATAGTTATTTTAGAATTTTTTACATAAATAATAAAATGCATTCATACTTT<br>TATTTTTAATAATTTTGTTTGAATCATTAAATGAATAATTATTAATAATAATATAAAAACACAGAATTAGG<br>TAATATTTTATCATAAATAAAAATATTGAATTTTTGTTGCTTTGTTAATCTGTGCAAATAAAAAAAATACA<br>GGAGTACAACAAAATAAATTATTATAATTTTTACGACACTCAAAAGAACTCTGCTTTAGAAAGATAAAAAT<br>AGCGTGAAAGATTTTGATGTCTCTATCGCATTAATTATGCTGTCTCTGTCTCAGACGCATTATTATTATTA<br>TTTATTTCTCTTTTTGTTTCAGAATTTATTTACAAATACAATATTTCAGTCTATTGTAACTTGAACTTGAA<br>GCATAAGAAAAGTTGATGATTCGGCAATTAATTTTTGAACAAAGGAATAAGTGGAAAGAGAAACAGAAGGA<br>AGTTGC
CrLEA2-49	ATCCTTCTTCTTTACATTACTTATATCTCATTGTAATCACAATTGAGATTTTGATATTTTTCTTGGGCCAA<br>GTCCTCCCTCTCTCAAAATCCTCCACATCTAGTACATGATCTCCATTCTCCATCATCAATATTCTTATTTT<br>CTCATAAAACTGCTGTCACCACATCTATCTTCAACAGTGCCCTCCCTGCAAGTAATAATGTTTGCACCAAA<br>CGCTTGTAGGACCATGGAAGCGAGGCCAACAATACCAATGCTTGCTTCTTATTAAAGATTTTATCATTCAA<br>ATTTAATAATTGGCATACTAGTTGATTGATAATGTGGTTATAGAGATCTCTCCTCTCTTCCCATCTTCATA<br>CACTCTACCACAAATAGATTCTTTTAATGTTAAAACTTTAGGATTCTGAATAGGAATGGAATGCTTCATGG<br>AATCTACAATTACATAAAAAGGTTACAAAGTCATTTTTGCCTTAAGTTAGTTAGGATATTTGCTTTGCTGC<br>GGTTCACTATAGAATGGGATCATGGCACCACGAGAAACTGTCGCTTTGTAGGCAACTTAATTTCCATTTTT<br>AAAACTTTTATTTATTTATTTATCTGCGTTTGGACACATTAATTATATCAAGCAGATGACCCCTATTTTAT<br>TTTCACTCGGCTCTTGGGATCATTGTATTTCAAGATTTTATTATCCCCTGGTACAGGACAAGAATGTTTTT<br>TTTTTAATAACGTGGTTCATCGTAATCCAATGTATGTTAGCAACACAGGGAATAAGATGGAATGATCCATG<br>AAATTTCGAGCACTGAGACCAGCAATTACTGAGGGGTAGGGTATTGGTGAAGCTCAAGAAATGCATATATT<br>CCTCCCCTCATTGATACTTTGGCAACAAACAGCTATTTCACATATTCCTCATGTTGCCATCCTTTAAATAC<br>AATGATTTGCTGCACCATTCATCACTTCATAATGAAATTAAAGCGAGCTCTAAAAAATCAGACAATTTTGT<br>TAAAGG
CrLEA2-50	AACAGTTAAGGATGTTGGGCCTTAATTAAGATTGTTAAGAATATCCTTGCTTTTGGAGTTCTGAGATCAGA<br>GTTTGAATCCTGGGGTTAATAAGAGTAAGGTCAACTTTAAAATTGCAAAATCGAAGTAGTCTACAGAAGGG<br>AAGGGGGTTCAACACATGGTTCTCTTAGCCAGAGACATGTCACATGCCATTTGCCACATGGTGAAATATAC<br>TAACAAATGAAGTAAGAGGCCCCAATTATCTTCTAAATTTCTGTTGACCATCATCCACATGCTTAAGTGAC<br>CATCATCCACATATATATATCACAGGCCTGCCTATTTAATTATGACACGTTCCTTTTTCCTTTATGTTGTG<br>GATCTATCAGCTGACTATTATGTAAACTTATCAAAATCCTAATGGTCGCCATTCATTTGGCTTGGCGGTTT<br>GCCAGCACAAACACTACACTTCCACAAATCTATTTGAATCTGCCTTTTATAATATTTTTCATTTTTTGAAA<br>CATCAATTTATATAATTAATATTTGAACTTATAAATTTTTAAAATTTTTATTCTCATTAATTTATCATAGT<br>TTTTTAAAATTATTTTAGATTTATCAACTAATTTTATTAAACATTTTTTATTTAGTTAATTTAATGGATCA<br>TCTTTAAATTATTAACTAACCTCTGATTATCTTATTTCCTAATTTTATCAAACAAAGTATTAAGTTTTATG<br>TGTATGGACAAAAAGCTCGTAGAAAAAGGAAATCGTGTAACTGCATAAACAAGAGCCGGACTATGAAAGCC<br>AAATGCCCCAAGCCGTCTAGTTCATTGTTTTCATCCCCAATACTAACTAAATCAAATAATCTGATCAAAAC<br>AGACAGTCACAAACGTATAGTTACAGCTATTTCTATGTAGTATAAGTTAGCTAACTTCTAGATATATAGTC<br>CCTATATCATACACATTAATTAGATAGTTTTGTAAATTCTACATTACCCGTATGTGAAAGTCGTCATGGCA<br>TGTGGA
CrLEA2-51	TATTACAATTTACAGACAGGAAAAAAAAACCCAAGTAATAGGAACCCTGAGTTTTGTTTGATTGGGAGAAAG<br>GAGAAATATGAAAAGAAGAATATTTGTGCATAAAAATAGAAAAGAAAAAATCAACTTTTACAAATTTCATC<br>ATTTTTTTTTAAATTTGATGAAAAATGAAATAAAGCTTTTATTTTTTATTTTAAATTATGAAAATTATTAT<br>ATATATTTAGGATTTAGGGTTTAAAAATTGAATTTTATACTATTAATATATATTTTTCTTTTTTTATTCAT<br>TTAAAAAATTTCTTTTATTTTAACCCGTTCAATCAACTATTTTTTGGTTTTTATTTTTTTATTTTTTCTAC<br>GTGGCAAAAGTGTCCCAAACCCATATTGAATGCTAAGTTTCCGTGCTAAAATACATCGGCCAACCGACACA<br>ACTACCTATTCTTAAATATGAAAAATTCTCACCTTTTAACAATCACAAACCTTAGCTATATCAACCTTTTT<br>TCAGAACTCAGTGTTTCGGTGGAAAAACCTTTTTTCAGTGAAAATACTGCATTTGATTAGAATAAGTAATT<br>TTGTAAAACATAAACTGAGGTACACATTTATACATTTTTAAAAGTTAAGATGAAAATTTTCTACTGTTGAA<br>AGATGATGACCAAAACGAATTTAAAATTTCACCGCCTAGACTGCCTAGTGCACAGAACAGTGGAGTTTGTG<br>GCACATGGTAGTGGAGAAATTAAAGAAAAGGAAAGGGTTCAGTGGAAGACAAAAAAATACAGGTAAACAGA<br>ATTGGATTGAAGGGAAGAAAACAAGGTCCAATGGTCCTACCCATCTCTTCACGCTCTGCAAATGTCTTTCT<br>TTTGTTTCAACACGCACTCCATGTGCTCGTGATTTTGCTTTGCCACATTCTTCCATCACATCACATCACAT<br>GCCTCAACATAACAAACATTGCGTTCTCTTTCTTACATTCACAGTAATAGTTCCTCTCTATTCCAATCCAT<br>CAAGCC
CrLEA2-52	TTAACTTTAAAATTTATTATCTTTATAACATATTAAAAAATTAGTCCTAAAAGAAAACAATAGTTTAACGT<br>GACAAAATCTTGTGTCATCCGATCGCCGTAAACATCTCATGCCATTGTTATTACCAAGAATTATTCGATTT<br>TAACCTTGATTATTTCATATTTATTATACTTTTTATATTATTTAGGAAAGAATTTTATTTTACTTTATTTA<br>GCATTTTATCTCATTTGACAAAAGTGTAAAGGAATACGCAAAAACAAAATGTTATTTTAAGAGAAAAGTGA<br>ATGAAAAGCAGGGGTGTTAATTTTTTTCGGAGTTAGTTTTTACCTCGATTTCCGATGTATGTAAAACCAAA<br>AATAAGTGACATGAAAATATCGTAGTTTTATATGAATAAAGACTATTTTACATCGGTAAGCAATGTATAAA<br>TCCACCGGAAGTGGAGAGAGAAATAAAGAATAATAATAAAGTTCCCGGGTGATGTAACGAAAAATAAAAAT<br>AAAAATAAAGGTGAATGTAAAAGATATGAGTAAGAAAATATTAAGTTTCAAGTACATAAAATTTTGAAATT<br>TTTATATTTTCAAACAATATATATCTTATTTTTATTTTTTTTTCTTTTCTATTATATTATCAAATTTTTAA<br>TTATTTATATATATATATATATTAATATTATCATCATTTATTTAAATATTTTATTTTTATCTTTTTAATCA<br>TATCACATAATTTATTATGTGTAAATTTTTTCTTATTTTATTTCATTTTTTTTACCATCCTCATATAAAAA<br>ATGCAAAAGATATCTTATTCATATTACAAATTTATACTACAATTTATATAATAACTTCTTTTATTTTTTTT<br>CTATCACAGTACGTACTATATATTTCACACTTTTTCTATCTTTGTTATATTGGTTGTTGTATAATTTTGTA<br>GGAAAATTATGATTTGTCTAATTAAAATATGTATATAAATAGGACGAAACCGCCGTGCGTTTTCCATCAGA<br>CTTCAT
CrLEA2-53	AATTTTTTTTCTTTTAGATATTTTATTTTCTGAATCACTTATTATAGTTTCAATAGATTAATTATAATAAA<br>TAATTATAATCAAAATATTTTATAAAATATCAAATTTTTTTAACAATGATTAATTTAATTTGAGTTAATAC<br>TAAAAATCATAATTAAAATATTTTAAAAATAAAATATCTAAACCAAAGGTCTATTGAGAAAAAAAAATTAA<br>AATTTAATTATTTTAAAATATTATAGATATATTTAACCCAATTTATTTTACTGATAAAGAAAAGAGAATAG<br>TACAATTGAGAGAGTGGTATTGATATTTCCTACTGATGTAGTTTGTGACAATCATATTTATGAATTATTAA<br>CTGAAAAAATAAATATATTTAATATCTTATAAATGTAAAGAGTATTCAATTATTTTATTATTTAATAAAAA<br>AATAATAATAAGATCACCTTGTAATTTGAGGCTGTAAGATGATTCGGAATTATTTCCTTTTAAATAACAAC<br>AGTCATACAGCAATTCTTGTCCAATACTGTATGTCGGGAACCATTCCAAATGATAACAAGCCGGTGCAGTG

	AAGAATCAGAGTTAGATCCTTACGTGGCCATTACATGAAGGCTTTCCACATGGCGCTTTCGGTCCGTTTAG<br>CAGCTTTCCATTTTGTATCTGAAATGAAGCTTAGCTAATGTATTAGCTGGAAAGACCAAAATGGAAATAAA<br>AACAGGTAAAAAGTGAATGACAGATGCAAACACAAAGGTTTGGAAGCTGAAAAAACCAACCAAGTATAGGC<br>AAAGCAGATAAGTGCTTGTTATCATGATTTGATCGCGCATCTATCAGCCCGTGTATTCAACGCGCGCGGTG<br>GTAAATAGGATTCAACCAGTCGTGTATGAACTCTAGAATTTATAACAGACCCTTCATTCACCCCCAAAATA<br>AATTCCTCAACTCCTTTCCTCAGCTTCCATAACTGAAAAACATCAATTCCCGCAAGTGCTGCCCGGTCTCT<br>GAGTTT
CrLEA2-54	CAGGTTCACAATTAAAAAATGTTCTCAATATTAAAAAAAAAAAAACTCATTTAGACCTGTATCTGATTTGGG<br>CCAGACCATAAAGTTTAAACGAATGAGGCCCACTTTGAAGTTTGGGTCATCATTCTCAATCTCTCTCTTGA<br>CCAAAACAATAATCTCATCCTTTTCTCTCAGCTGATGAACCTGTTTTACTGTTAAGCTTAGATGTGAGATG<br>CTAGAATATGGGATTGAGGCCAATATTTTATTTTTTAAGAATTGACAATCTTCAACGAATTTCAATGTATA<br>TTAAGTATTAACTTTTACAATAAATTATTTAACAATCCATTAAATATAACAAATAAAAACACTAAACAAAT<br>GTTTTATGGTTTGTTAAAATATCAATATATGAAAAGAAAATGTACTAATTGATGACTTCTCCGTCCAAGTG<br>CGGACGCATTCGCTTTAAAGTCAGGAAATCTGCAAGGAGTTACTGGGTATTTGGCATGCAAGTTTGGTATT<br>TACTTTTTGTACAATACTAATTAATATAAAAAAAAACTATTTTTTTATTTCCTTATTCTCACACAATGTTT<br>TCCTTTAAGTAAATAATAAGTTAAGAAAAAAACACATCTTTCTATACGCATTCACTTTTTTCTCTTTTTTC<br>CGAAACAAGGGTAGGAAGAAGAGTAAAAAGCATGTGTAAAAATAATTTTACTTGCTTTATATTATTAAAAT<br>AGCAACTAAGCCCTTGTAAGAGAGAAGGGTGTAGTTTGTAGGAAAATATTAAAAATGAGGATTATGAGAAG<br>GTAGTAGGAGACAAATGCTTTAAAGTTGGGAAAGGAAGATACCAAAACCTTCCCACCAATTAGAGATAAAG<br>AACCCAATTTTTGTGCAGTTCCCTACCTGGCTAAGCTTGCCAACCCAAACCCAACTCCTCCCTCTCAAGTC<br>TCACCCCCACACAACAACATAATAAAACTCCCAAATAACCCCACATGGGTTGATTTTGAAATTTTCCATCA<br>ACCTCC
CrLEA2-55	TTTGATTGAACATGTGGTGTAGTAAGTATACAACATATCTAAACATTAGTTTTCTCTCTAACTTTGTGCTC<br>CATTCATCCAACTAATAACCAAACAGACACCAAGCAGTGCTTGTTATAGTAATTTGGGGAATTGATTTGCC<br>TGGAAACAAAAAAGTTATCACATCCATGGAACATGCCTTGTAAATTGCGTCAAACATTATATCTACCCTTA<br>AATTTAGTTTTCACAAAACAACCTTAATTGTGTTAAAACAATTTGGATTGTTATTTTTTCTGTTCCTTTTT<br>GTCTCTTTAGATAAAAAAAAAAAAAAAACCATGTTAAGAATGCTCGTAATTTGAAAATTTAGTAAACTAACA<br>CATTATATTATGAAATAATATAGTTATTATTCTTAATTGAATCATGAAATTACAAATTATTTACAACATAA<br>TTTTTTTCATGTATCACATAATCAATTCAATTCAAACCATTTAAATTAAAATATATTATATTCTTTAACTT<br>TAATATCTTTTATAACAATTTTTTCTAATTATCTTTCATCTTTCATCTTAAAATAAACTTATTGTTTGTAA<br>TGCGGAATATTACACTTCATTTTTAATTTTCCAGCTATGAATAATTGTTTATTAATTATCTAATATCATAA<br>TATTACCATAATCATCATACTGCAATTATTTTCACTATTTGTTTAGATTATTTTTTTAACAAAGAAATTAT<br>TATCTAATATCATAATATAACAATAAAATTTTAACAATATTAGAAAAATAAAATTATTGTTTAGATTGCAG<br>ATTTTTCTTTGTTAAAATAATTTTATTTGAGTTAAAATGTTAATTGAAATTATTAAAATATTTTTTATAAA<br>ATTTTTAACACGTTTTAAAATTCAAATTCAATATTTCTGATTGAGTTAAATCAAATTTTGGTTGCAAACTC<br>AATCAGGGATTTGAATGACAGTTATCATCCATACACGTCACACGCCTCCGTCGGAATCGAGTAATAACAAA<br>CAATAA
CrLEA2-56	ATATATTTTAATGATAAAATATAATAATCAATATTTATTTATTTATATATAAAATATTTCATTTATATAAT<br>ATGTTTTTTATGTGATACTATCTTGTAAGAATTTGTTTATAAAAAATAATTTATTTTTAAAGTAATTTAGG<br>TTAGAATTATTTTTCTTGGTAACTTTATAAAATATAAATATAATAATTTTTTTATTAAATAATATGATTAT<br>TATAAAATCATGTGCATTAAATTTTAAAAATTTAACTTATATTCTATTATTTATATGTTTAATAATATATT<br>ATATGTTAAATTAATATTATTTAATAAAATTTTTAAATTTAACTTTTATAATAAAAATTCTTAATTTAATA<br>ATCAAATTTATTAAATTCATATCTTATAAAATATTATCAAACAAAATAATTTTGAATAAAGTATTTATACC<br>CTTTAATTTATTTTTATTTCAGTAATTTATTCAAATCATTTAGATATATATGACGTAAGTTGTTTCTTTAT<br>TTTCAGACTGCTATACTTTCTTAGTCAAACCGAACGGTTTACTTTTCTTTTGGTCTCGAACTATTCTTTCA<br>CTTACAATACAATCATTTAGTTCTTACAACCTTAAATAATGTTGAGTTTATATCTTATTAAAATTGATATC<br>TCTAAAATAATATTATTAAATAATAAAATATAAAAAAAAACAATTATATATAAATAATTGTCCGAGCCTTA<br>AGAGTAAAGAAACGAAAGGATGTGTGAGCACTGCTGAACTCATTGTTAGAACATTCTCAAACATTACAATT<br>AAACATTCAAAATCCAAATCTCAATGTAGCTCAACTTTATCTCCTTTTGCCAATACCCAGAAAAACACACT<br>GTCCCCACTACTACACAATTAAAAGCTTCTTTATCCCTTCTTCACTTTCCTTAACCCATTACTCCACTTAA<br>GAATAAACAACATCTTAATCTCTTCTTCATCGCTTTGTCTGAGTAAAGTGACAATTTTTAGTAATATCTAC<br>TCAAAC
CrLEA2-57	TTCTTTTTCAAACTGTTCAAAATTAAACGCATTCTTTTCTATGAAAAAAAGAAGAAAAGTAATACTGTTCA<br>GATAGAGAAGAAAAGAAAAGTAGTACTATTGGAAAAATATATCATTATAAAGAAAGACATAGAAAAGATTA<br>TTTATTGTCAATTTAAGGAATATATTTGAGTTCGATTTTAATTTGATCGTTGATTTTACTAAATTAATTGT<br>AATATTTTTATAATAATAAGCACTAAAATATTATAATTATAACGTTGATATAAACATCAGTTATCTTAATT<br>CCTTAAGAAAATAATAACTAACGGATTGATAACATTTTTAATTATTTTATGGAATAAAAATACTATTAAAT<br>GATACTTGCCATTGTTAAAATAAGGAATATATCTCCTATCATTAATATACACAAAAAATTATTATTTATGC<br>ATCATAAATTGTTATACTATACTTTCTCATACATTTTTTTTTTCTATGAATATTAGATCATGACTTCTTCC<br>ATAAGAAAAAAATAATGTAGAGACTTTTGGTGCTGGAAATATACCAATTATATATATGCCAAGAACTAAGAC<br>AATATTAACATCTTTTATCTATAGCTAAAAATGCTTAAAAGCAAGAAAGTTAGCTCGACCACTTCGACGGC<br>ATTACATATTGATTTAGTATTATAAAGGTAATGTTGAATAATTGGCACTTGTAATAGATAAGTCTGTCATA<br>CAAAATAAAACCTGATAGTGACCCTTTCAAGTTTTAACCATTCAACTCTAACATTAATTGAAGCTTCATGC<br>CTTCTTCATATACCTGCTCCTATACGTGTTCATGTTCCCACCCTCTTGCTAGTTGCAACACCCTATTGACC<br>CATCTATATATATTTGACAGAGAAACCAAATAAAGAGAGAAAAGAAGAGATATATCATCAAGGGCTGTATC<br>CACATATATGCAGTGACCACATACTCACATATATAATATATGATATATTTCTTCTAGCTATTGTGATACCA<br>AAAAAC
CrLEA2-58	TGTGTCATCCAAGATTTCGACAACACGACATTTTAATTTATCATCTCTGCAATCGTCATTCATCATAAGAC<br>TAGGATTAGCTTCTTAATATTTTTTAAACGAAAGGGTTGTTATGAGTTATCCAACTATTTTTCTTTATTAT<br>ACATGTATCACTTACATGGAACAACCATTTCCTATTTATTAAAATGCAAGTGTTATCCGAGATCATAATTT<br>ACCTTCTTCTTCTTTTTCTGTGATGATAAATATCGCTCACCTTAAATTTACCATTTTTTTTTAATTTTTAT<br>ATATAAGTGTCATTCATCTGTCCTTCTTTGTTTTTTTTTTTTTATTTATTAAGCAATATCGTCCATATATAG<br>ATAGATAATACTTGCTATATATGAAATTAATAAAAAATGTCAATATACTACACGGAAAAAATTACCCAAAA<br>TTAGTAATTAATTTCATTTACATCCTATTTTGGTAAATGATTTAAAGAAAGACACTTTTTGGTAAAAAATA<br>TCATGCAGGTAACTAATCTTTTTACTGAAAAGAAAATTTGGATTGATATAATCTTTTATGATTGTTTTTAT<br>AAGAATAAAGAATAAGATTCGTTTTGATCTTTTTTGTAAAGCTCAATCTACTTCGATTTTGTATTAATTAT

	TTTTTATAAAATATTTTTATTTAAGTGAGGAAATATTTTTATTGATGATCGCCCAACTGGAAAGAGATTAA<br>AAGATTGTATCTCATTAACCTTAAATTTTTTTTATAAAAATCATATAAATTAATAAAAAATTTAATGTTAC<br>TCGCTAAATTAACTAAATTTCCTAATTCCACACATGCTCCCCTATGATGCGTACCATTATCCAACACAGTT<br>TTACGCTTACGCGCGCACGCTTTTTCCTTCTAAGCCTACTTAACAAGCTCACAGCTGCAACGTTGCAAAGC<br>ACACTCATCATTCTTCTTCTCAATTCTGTTTCAATTGCTGAAGACAACTCGTAACTCGTCACTCAGTGACT<br>CACCCA
CrLEA2-59	TGTCATCCAAGATTTCGACAACACGACATTTTAATTTATCATCTCTGCAATCGTCATTCATCATAAGACTA<br>GGATTAGCTTCTTAATATTTTTTAAACGAAAGGGTTGTTATGAGTTATCCAACTATTTTTCTTTATTATAC<br>ATGTATCACTTACATGGAACAACCATTTCCTATTTATTAAAATGCAAGTGTTATCCGAGATCATAATTTAC<br>CTTCTTCTTCTTTTTCTGTGATGATAAATATCGCTCACCTTAAATTTACCATTTTTTTTTAATTTTTATAT<br>ACAACTGTCATTCATCTGTCCTTCTTTGTTTTTTTTTTTTTTATGTATTAAGCAATATCGTCCATATATAGA<br>TAGATAATACTTGCTATATATGAAATTAATAAAAAATGTTAATATACTACACGGAAAAAATTACCCAAAAT<br>TAGTAATTAATTTCATTTACATCCTATTTTGGTAAATGATTTAAAGAAAGACACTTTTTGGTAAAAAAATA<br>TCATGCAGGTAACTAATTTTTTTACTGAAAAGAAAATTTGGATTGATATTATCTTTTATGATTGTTTTTAT<br>AAGAATAAAAAATAAGATTCGTTTTGATCTTTTTTGTAAAGCTCAATCTACTTCGATTTTGTATTAATTAT<br>TTTTTATAAAATATTTTTATTTAAGTGAGGAAATATTTTTATTGATGATCGCCCAACTGGAAAGAGATTAA<br>AAGATTGTATCTCATTAACCTTAAATTTTTTTTATAAAAATCATATAAATTAATAAAAAATTTAATGTTAC<br>TCGCTAAATTAACTAAATTTCCTAATTCCACACATGCTCCCCTATGATGCGTACCATTATCCAACACAGTT<br>TTACGCTTACGCGCGCACGCTTTTTCCTTCTAAGCCTACTTAACAAGCTCACAGCTGCAACGTTGCAAAGC<br>ACACTCATCATTCTTCTTCTCAATTCTGTTTCAATTGCTGAAGACAACTCGTAACTCGTCACTCAGTGACT<br>CACCCA
CrLEA2-60	AATTTTTAAATTTATATTATTATAATTATTTTAATTTTTAAGTTAATATTTTTATTTATATATTTTTATAA<br>ATTTATAACAAAATAATATAATAAAGTAATTTAATTTATTTTTTAATAATTTTTTAAAAATTAAAAACAAA<br>ATTAGTTTTAGTATTTAAAATTAAAAATTAAAAATCACCTCAAACAATGTTTAAAAAATTAAATAAAAATT<br>TCTAAACTGTTAAATAATAAAAAACTTAAACCTTAATATTATTTTTAATTTTCATACATCAATGTTTGATT<br>TTAATTTTCAATAACAATTATTTATATTTAATTTTATATTTTTAATTATTAAAATTAAACATTTCACTTCA<br>TATCCCATTCAATAATTAATAAAAATTGTTAAAAATATCTATCACATTACACTAATCATATAAATTGGTAA<br>AGAAAATTCATACAATGTTTAAATAAAAATAAAAATGTATAAAATATATTTTCATTATAATGTTACGTCAT<br>GCTTGAGTTATATTTTTGTTAATTATTTTATAGTATTGATTAACTGTAAATAATAGATATACTTTTTTTTC<br>GTTGAATTCATCAATTATTAATTATTGGATCGAATTTAAATGAGGAAATTAATTAAATAATTAAAAGATTA<br>TAAAGATTAAATTTCGATAAAATAAAGATGTCAAAATCATAGAAGGACTAAAATACGGAATCTAAAGTTGT<br>AATTAAGTCAAAACATAATTAGCGCTTCTTTTGTTGTTATGTGAATTTAAAACGTAAAAAAAATACAAAAT<br>AAAAAAAAATTCAAAAGCAGTAGTCAAGTTGAAATTCCACATATGCCCCTATGACGTAGCATCATCCAACA<br>CAACGTTTTACGCGCACACGCTTTTTCCTTCTCAGCTACTTAACAAGCTCACAGCTGCAACGCTGCAAAGC<br>ACACTCATCATTCTTCTTCTCAACTCTGTTTCAATTGCTGAAGACAACTCGTAACTCATCACTCAGTGACT<br>CACCCA
CrLEA3-1	TTGCCGTATATATCGGTATATTTGATCTGATTTTAATTAAAAATATTATCATAATTGCTTGATGGCAGCGT<br>TAATGATAACTTGCATAGGTCAGCCTCTCTTTTGTTGCCACTTATCACACAGGCCTACTACTATTGTGTTT<br>TTATCCAATTCGATTTGATTCGACCCTCCGCCAAGTGCTCGCTTGTAGTTGTTTTAACACAATCAGGCTAA<br>GCGGGAGATAATTAGGTCCAACACCAAAATTCATCTTCAATTTTAGATTTCATTGTTTAATTTGTTTGATG<br>TCAAATTTTAATTAAAAGTGAATTTTTTATTCTTATAGTTTATAAAATTAATTTTAAGTGTGATATTTATG<br>AAATTTTTAAAATTTATAATTATATTTTAAAGCTATTTTTCTTATTGTTTAAAAAATAATTCTTCCAAATT<br>TATTTTTGACTTTTTCTAAAACATAATAAATTTTTTCAACATAAACGATTTTATTTATTACTTTCTAATGT<br>CATTTTCTATCAACTTTTAAAACAAATGAACCAAATATAATGAAATTTTCTTGATTAATAATTTAATAAAT<br>AATTTTAGACTTCCAAAATTCTGATAATTATACCTTTTTATTAATTTAAAATTATATTTATCGTCATTAAA<br>TATACTTAAGAGCTGCATAAACGATAGATAGTCGTTGAAGTCCTTGCATTTGATGGTCTGCGATATAGATT<br>AGACTTATCTTATTTGTAATGATATTTATTGTTGTACATGTAAAGCATGAATGAGGATTGGAATTTGGAAA<br>GTAGAAATGTAGGTAAAGTCGTAAAGACATATCATGATAGTTGTAAGAAAATAACGCGTACACTGATTGAC<br>GAGTGTGATACAGTAATATCGTAGTGAGTAACACGTGTCATGAAGATGGCGGACCTTGGATTAGGTGTTGA<br>CACCAAAGTCGTTACAGTTGGTTCCGCCCTCAGTTTTCTAAAATAACCAACCCAGTAACGCCACCCGTACA<br>CACACT
CrLEA3-2	AAACAAACAACTTTTTTGAAGTTTATTTATTTATTATTAAATAACATTCATGAGGAGTAACAATTTTCATA<br>ACTTAAACGAGAATAGGTTTAATAGTTAAAAGAGTCCCAAAATTATTATAAGAGAAGAAACAGAAAACATT<br>AATAATATGGTTGAATAATAAATTTAATATTTACATTTGTAACAACACTTTTTTATTAAAATATGTAAATT<br>TTATTAGTTACATAGTTACTAAAAGATTATCTCTAAATATAGAAGATTATAGCCATCATTAATATTAACTT<br>TTGTTCTAAAATAAAAGAAAAAAATTACTTAAAAATATAGATGATCAATCATATTTCATCTATCCATTAAT<br>TTAATTTTATCATTTTTATTTTTTTTATGTACAAATAATATTTTTTTTATATACAAATAAGATCGTTCATT<br>ACTAAAAGATGATTCATAAATTAAAAATAAAGTAGATAAATTATTTATTTATGGATTAAGATTAAAATGAG<br>TTATTTTTATTTTTTTGAGTAAAGCTAATTTACTTTGTATTTTATTAAATAATGTAACAATCTATATCATG<br>AATCTAATATGTCTAAAAAGATAAGCATAGAAATAATAAAAATCCTGACTTTGAAGCCTGGACAAACACTC<br>AATGAAACTTGTGACATGTCATAATCTTATAATGAGGGAACTTTCCTCTCAGTAAATTTTCATACCCCAAA<br>AAAAAAAACAAGTTGGTCACCCTTAAAAAGTATTAAATTAATCTATCGTATAAGGGATATATCCAAAGACA<br>GATAAAATCCTTTTATTTTTGTTTTAAACTAAAGAATATTTATCGCAGGTATTTGGTTAAAAGAAAGAAAG<br>CATGATCATTATCAATAAAAAGAAAAAAGAAAAAAAAAAAACAGCATTATCATTTGTCACCTTGTAGTATTG<br>CCATGATTCTCGATAATAGTAACTGCTTATGAGATATAAGTCGTTTTAGAGGGGAAAAAACCACTTCTTTC<br>TCTTAT
CrLEA3-3	TTATTTAAATCTAATTTATTTATTTAAAAAAATTTAACAAAATTAAAATTATCTATTTATTAAATAAGTCA<br>ACACAAATTAGACTTTTTAACTTCTTCTTAACCTTGTTATCTTCTATCCTTATTTAAGAAAAAATCTAGAT<br>AACTATTTTCATCAATATTATCTTAAGAAGAATTTTAAATAATTTTTTATTTTCAATTCTCATATTTTCCT<br>ATACACTGCTTTCTTTTTAATAAAAACTAATTTTATAATTTTATAAATCATTTTTATTTAAATTTCTAAAT<br>ACTAATTTTAGTAATTTAAATTTTTCTAAAATTGAATTGAATTAAAAAAAATTAACTTTGAAATCATATCA<br>AATGAAATATTTCTTAATTTTTGTTAAAATTTTATTTTATCATTTTTATGAAAAACATTTTATATAACTAT<br>TTGATATTAATTTTATAAAAATTAAATTTAATATTTTATAAAAATTATTTTGCAGAAACAATTTTAATGTT<br>TTATCATTTCAATGTTATATAAATTCAATTTAAGAGTTAACAATTATAATTAAAAAACAATGTTAGGAAAT<br>TATAATTTTTAATAAAAATAATTATATTTTATTTTAATAATTTTTAATCTATTTCAAATATAAAATTAAAA<br>ACATCATCTAATTTATTATCTAATATAAAAAAAAAATCTACTTTACTCTTATTATATTTCATCCTCATACT

	ATCTCATACTATGATGTGCTATTGTAAACATCAAATTGACAGATTCCCCCCTAAAGTTGTTTGCAGGCTTT<br>CCTTAACACTCGGGTTGGTATGAAAATATTGCACTATTCAATGTGGGTCCTTCAATTGAAATAGGACCAGG<br>CTCTATGAATGTAACAGGTGGCAGCAAAGAAGAAGTTGCACATGAATAATTCCATATTTTGACGCGGCTAG<br>TATTCTGCCTTAATCATGCACCCAAATTGTGTCTTTACTCAATTACGCGTCTCCACTCTCTTTCTCTGCCC<br>TATATA
CrLEA3-4	TTTATGTTTTTAGTAAATTAGGATTATATAATAGGTATAATTATGGGAATAATTATTATAAAAATTAATTACT<br>TAAATGATAAAATAAATTATAATTAAATAAAATAAAAAATATATTTATAATGTTAATATATTTAAATTAAA<br>TATTTAAAATATTAAAATTGAGCAATTGATAAGAGGTAAAACTAATCTGCATGAAGTCATAGATTTAAACC<br>TTATTTCTTTTATTAGTGTACATAATTTTATTTTATTTATTGCGATTTCATATAAAGATTTTGGTTGATAT<br>AATTAGTTTTTTTATACAACATGTAGAACGGTGTTTGAATTTAAATTTTTTATATCCAATGATTCATTAC<br>TATATATAACTTAGTTTATATTACCAATTTTAAATTGTAAAAAAAATTATAAAATTCTGATATTTAAGATA<br>ATTTTGACATTTCGTCTCTGTTGTTGTTTTTTGAATAATATTTATTTAAAATTTTTAACCACTTTCAAATA<br>TTTATTTAAACGTTTAAAATAATCAATTTGAAAAAAGAAACAAATTTTTTATCATTTTAAGAAAAAGTTTA<br>AAATAATTTAAGATATTTTAAAATGATAATTATATTTAAGATAAACATATTTTAATAAATATAAAAAAATA<br>AAATACAATTTTTTCTTAACTTTTTTTGAAAAAATTTGAAAGCATGCATTAGTTTTTTAATATTTTAAAAT<br>TACTTTGTTTTCTAAGCTTAAATAAATCGATACAAGTTAAGGTAACGTTAAAATTTCGATGCATAGAAGTG<br>TAGCAATAAAATTACTATTTGTACAGTTTGTTCATCCCCTTCTCTAATTTCCGTATATCTACACATTTTGT<br>TTAGGATTTAGCAAAATTAGTTCGAATTTATGCTTTAAAAAGAAAATAATGGACAATTTACAATTTTAAAA<br>AATGGGTATAGGAATCAAGCAACAGGGCTACTGGAAAAGAAAAAATGAATAGGTGTGAGAAAACGAATTTT<br>GTGACG
CrLEA3-5	ACATTTTTTATTAAAGTAAATTACAATCATATTTTTAAAGGTAATTAAATTTTTATCAAATAGTTATTTTT<br>ATTTTTATATCAATTTTCTAATTTATTTAATAATACAATATCATATTTTCTTTTTATTATATATAAATTATCT<br>TTAATATTTTATACAACCACATTAATAGAAAGTTGAAATATCCGATGAATTTCATGAATTTTATTGTAAAT<br>GTAGTTTATCCATTTTATTAAAACAAGGGTCATGTGAGCACTCCCTTCTCTTTTTTCATTTTACTTACTAA<br>AATTGTTTAAGCTGAAAAAAAAAAAGATACTCATACTCCATAAGGTTAAAGATTTTTTTTTTTAATTTTTA<br>ATTTTTTATATTTTTCTTTCTCTTACGTAAACACAAATCCGACTCACTCCATGGCATATGTTCTGATTCAC<br>ACGCATTCCACACAAGGTAGCAAGTAAATGGAGCAACAATTCCGTTATATAAACATCAAAACTTTAGACCA<br>TACATGAATCCTATATAATAATATTATCTTTGTCTCTCCACAATAGTAGTATATATGTTTGAAGTACGAGA<br>GCAGAGTTTCATTATATCATAAATGGAAAATGGTGTTTGCATCTCCTTTGAGGTTTTATTGAATAATAAAT<br>ATATATATATATATATATATATATATATATATATATTTGTAAATTCACCTGCGATACTCAATGAACAGAAA<br>GAAAGAAAAAAAAACATAACTTTTTCTTGTAGTTGTTAAAAACTTGTTGTAGTCTCCTAATTTTTAGAGTG<br>CCTTGCATTTGTATTTGAAGTATGGCTTTAGAACCACTGATTCATTTCCAAAATACAATCACACAACACTT<br>TCTGCCTTTGTTGACATCTAAAAAAGTCACCCTTTCTAGATGGCTCTGTGCTTCACATGCAATAGAACTTA<br>TTCTCCATTGCATCCTTCCTTACTGCAGTACTAGACTATTATAAATACCCATCATAAGGCCTCGAGGCTTT<br>GAAAAG
CrLEA3-6	TTTTAAATATCTTTAAAAATTCAAATGATAAAAGAAAAAAGCAAGAATGGAAGTTAATAAATAAGGTACAT<br>AATAACTTGTTATCTTTCCACCCTCTTATCATTGAAATAAAATTCAATGAAACTATAAAAAATATTTTTTT<br>GAGAATCAAATATGTATGAAAAATACTTCTAAAACTTATAACAAAGATGAGAATTATACATTCTAAAATTT<br>ATATCCTAAGAAATAAATTTGTGAAACTTGAAAACAAACACTCTCTAAATATACCATTGTTTCATTGTACT<br>TTTTAAAAGAAAAATGTCATTGATTTTTCATTATATATATACCTAATAAAAATAGGAAAAGAAGAAAATAA<br>TGGGAGAAAAAGTTCAAAAAGTAATTGATAAATGATATAATATGATAATAAAAAATAGTAGAAAAATAGAA<br>ATATAATATAAAGTATTATCAAACTTGGAAATTATTTAAACTATTTTATAAGTTAAACAATATTATTTTAA<br>ATTAAATAAAAAATTTATAAAAAATAAATATCATTTTTAAATCAACATTTTAATTTTTATTAAGTTGTGTAT<br>AATTGCTAATCCATCGAGGAACTTAGATAGTTCTTCTAAATTGTTATTTTTTAAATTATTAAGATTGATTT<br>AATAATAAAAATTTAAAATTAGAGTAATTTAATAAATCATGTTATTTTTTTTAAAGAAAAAAGAAGATTAT<br>AATAATTTATCTCTCTTTATCTGTAACAAAAATTATAAAAAAAAAATATAAAATACAGTTTCCCTTTCAAA<br>GTAGCACAACCAACACGATACCCATTCTCTCCCTAATCATAAACGAAGATTTTTTTCCCCTTCTTTTTTAG<br>GGCAAATACTCGGAGAGTATTTAACAGATACATTAAGTTACTTTTAATTACTTAAAACAGTAACAATATTT<br>TAAAAAAAATCAATATATCAAAATTTACATTTTTAGTAAATCTTAACTTTTTTTTTTTTGACTTGTCCTTA<br>GTTAGC
CrLEA4-1	ATACAAGACCTAAAATTGGGAAGATCTTAAAAATAGAGAGTAAAAATTTAAAATAAAAAATAAATATTTAA<br>TTAAACCTTTGATATATTTTACATACTAAAACAGCCGTTCATTCATCATATGAAATAAAAATGGTCCAACC<br>CGAGTCCTAAATATTCTTTACGAATTTATTAGTCATTGTTATATTAAGATTATGTTTATAAAAATAAGTTT<br>TGTTATATTTTTTACACACTTTGTTATTATTTTTATTATATAAAATAAATACTAAATAAAAATTTATATAT<br>TTATTATTTTTAAAAAATAAAAATATTTAATGTTTTTATTCATAAAAATATCTCATAAATTATAAATATAT<br>TATTTCTTTATAATTATAAATATATTTTTATTAGACGTAGAATATGTTTGGAAATTGTGAAAGTTCAGCTA<br>GTTGAAATTATTGGATAAAACGTTTTTAGTAATTAGAATGGTTAGTAGAAAATTTAAAATCATGATTTTTA<br>ATATGTTCAAAATTATATAATAAAAATTAATTTTTATTAAAGATTATAATATTAAATATTATTAATGTGTT<br>AAATTTTGTAATTTTCAATAAAGTTTTAGTGTATGCAATTTTTTAGTGTCTTAGTGCATAATATTAAAAAA<br>ATTCTTTAAAATTATACTGTAAAAAAAAAACTTTCTATTATGGGATAAGTTATGTTTTAAGTATAAAACAA<br>TATATGCTTAATAGTCATAGATGAATTGTGACAGATTCGATATTAAATGACTGTAAACATGAAAAGGAAAT<br>ACAAGCGCATCCTCATCTTACTAGAAATTATGCAGAGAATTTATGAAAGTGAAACAAAAGAAGGTGAAGTG<br>GTGGTCAAACCAAAACTAAAGGGAACCGCTTTAGCCTGAATACCCCACGTGGTTAGACACGTGGAGAATCT<br>GATTCTGAGATTGTCTTTGTGACACTGACAGAGATTTAGAAGATAGATGTCACCAGCGAGGAGCAACACAG<br>GCTACG
CrLEA4-2	ATGAGAAACAAATTTTATAAATAAAATTATCTATATTCATATCATTTTAGCTTGAATTGGATCAATTTTTA<br>AAATTAAATTTAAATTTGATCTAATGCAATAACAAAAATCAGCTTTTATGATTTTTTAAATTTTTATAATT<br>TATATTAAATTAAATCAATTTCTGAACATCTTTAATTATAGGAGACAGAATAATTAACTTTAGAATATACT<br>ATTCTTTTATAATGATTACTTTTTTTAATCTTTGTTATATCAACCATTACTTTTTTTTTTCTATTTACTGT<br>ATATTTTGTTATAAAAATGATATATGAATGCAATTTTCTTTGTTTTCCGGAGATGGTGATTACCGGTTTGT<br>CGATTGTAAAAAATGTTCATTTGATTATAAATGTTGGTATCATATATTTTAATATTTTATTTTTTTCAACT<br>CATTTTACCTATTTTAATACACACAAACATTTATTCTTTTCCTATATAATGTTCAAGAAAACAAAAAAATC<br>TCTGTAGTATTGAATACAGAGTAGAAATCATCATTTTTCAGAATTCCTATAAAATGAATGTTCTGGATTTT<br>TGACGACTCCAGCTGGACATATATTCCATAGAAGGATGAGAGGAATGATTTGGTTACTCCTTCCTTCCTTC<br>AGTATGTGGCACCATTTGAGTTAAGATAACAATACATCATAATGAATCATGATGTTGGGCTTGTAATGGG<br>ACCCAGTGACCTACCTCTCCTCCCTCAATCAACACTTAAATCTTCTCATATGTTCCCTCCTCCCCTGCAAT

	TCTCTAACCCATTTCAGATTAGATGTCACCACCAAGGAGCATCTCATCATGACGTGTCACACTTCCCAAGC<br>ATGCCTGCCAAATGTCCTCCCACCGCCCCTTCCCGCCAGGTGCCACTTTCTCATACCCCATGTGTCACTCA<br>CTCCCTCATTCCTTTCCATTCCCTCCCCACTCATATATATCATCAAACCCTCATTTCCATTTCTCATAACA<br>AATTCG
CrLEA5-1	ATAAAAAGAATATTTATTTAAAAATTGTGTTGTTAATATAATTCATAATTTACATGTATTAAATATTAGTT<br>CAAAATTCTTAGACTCCTCCTTAATAATTTTTCTACAGTAACTATCCAGCAAAAAACCTAATTGCATAAAC<br>GACTCAAAAATAATATATATATATATATATATATCAATCAGAATTTATTGATATTATGTACCAAATTTAAT<br>GTTTATATGATTTAATAATTTTTAAGAAAATATAAATTTTATTAATTTAATAATTAATTTAAATTTTTATA<br>TTTATAAAATTTTATATAAAATAAAAATTAATAAATATTTTATTATCATATAATTTTATTTTAATATTTAA<br>TATATTACTTAAATATTAATAATATATTTATTTAAACTTTAATATAAATAATCATAAAATCGATTAAATTA<br>AATAATCTTATATTCTTTAAAAAATTAATAAATTAATATAAATACTAAGTATTACATCTAACATAGATTTT<br>GAAACATCCCTAGTTAAAAAATCTTATACTATATAAAAAATTATTAAATTAATATTAAATTTAATATCTAA<br>CGTAGATCTTGAAAACCCTCTCTCTCTCTCTCTCTCTGTAATACTGAACTTACCATAGCACCTGAAAGAAA<br>AGGTTGAGACTTGAGAGAACCGCAACCGAGGCACTTAAGCAGAGTTCATTATGCTCTAGAAGAACTGGTAC<br>GTGTCATTTGGGGAGTTGCAGCTGAGTGTCATTTTGGCCATTAGCCACGTGTGTGTCTCACGTGTCAATGC<br>ATGGCCACTGTTACTGTTCCATTTTAACCTGTTGTCAGCTCCTGGGATTTGCCTCTACATAGATATGGGTT<br>ACGGAGCATTAATTTAATTATATGTTACATTTTGGTGTCCATATTATATAAAGCCTCAAATAACCCTCCTT<br>TGCTGTCATATAGCTTGAAGCCTTCAACACATAGCAAGTTTCACACATAATTGGTATTGCAAATAAGTGCG<br>TAAAAG
CrLEA5-2	CAATAATTTACTAACCAATAAAAATAAGTAATTTCCTCCACAACACAATCCTTTTGTTACATTTTGTTTTA<br>TTAATAAAAAATTAATCCAATTTATCATCATTATCATTATTTTAATTATTTAATAATTATAATAATATCAT<br>ATAATAAATAATAATTAAGAATAAAAATATTATAATTTGAATTTATAATATTATATATTTAAAACAAACAC<br>AACCAGCTGCATGCCATTGTAAAGAAATTTTTTTTATTTTTTTTTATTTTTCTATACTATAATATAGAATA<br>TTAATATTTTTCTTATTTCCTTTATTAATAAATGGTTTTTATCAGCATATTTTTAATAAAAATTAAACTAT<br>TAATATAAATATTATAAATTTGTACTAATAATAAAATATATTAATAAATAGTTTGATTAATATATTATCAA<br>TACATTAAATATTAATATAATATATTATTTATTAATATAATTTCTATAATTAATTAGTATTTGAACATTTA<br>ATTAAACAAAATATATACTAATAAGAATTATTCAAACACTTAATATATTTTTATTTAATAATAATTTTTAA<br>TTATTATTATTGCTATTAACATCGTTACTAATATTACTATGAATATGAGAATGTCTTAGTTTTTCTAGTCA<br>TTTAATATGAAACCAATCAATGATGGAATAACCGTAATTCCGCATTTTGGACTTCCATATTTTTAATTTAA<br>ATTTTAATACCGTTAATTAATTAAAGTAATTATACAATTAATAATTAATGATAATGTGTACCACAACCGAT<br>TGGTACATGCTAGAAGTGCTAGAATTACCGTGCAAAGATTAGTATGTGTCATGATATGCCATTTTAACAGT<br>GGCACCTACGTGCGTGTCCTCTTTCACCTTTAGCCACGTGTCCATGCATGCATTCCTATGCTTACTTCTTC<br>CCTAGCTATATTTATTACTCTGCATTAGTTTACTACTTTAATCACAACACATGCATCTTTTTTCACTCTAT<br>AAAAAC
CrLEA6-1	AACTAATTTTTCTAAAAATAATTAAATTAAAATTTTTAAAATAACAAAAATAAAATTATTATTATTATTAT<br>TATTGTTATTATTATGATAATAATGGTATTATTATATATTATTTAATAATTAATATATATAAAATAAATAATT<br>CTATATATTATTTATTTTATATAAAAATAATATTATATATAATAATATTATATTATGAATAATTTTATAAA<br>TTAAATATAATTTATATATCAAATAATATTATTTATTTAATTATAAATTTATAAAAACATATAAATAAAAA<br>ATAAATAAAATTATTATTATTATTAAAATATTGATAATAATAATAATAGTTGTAGTAGAATTATTGTTTAA<br>CGAATATAATGATTATAATGGTGATAGAAAATGTTATTAGATAAAATTAAAATTAAAAATATTTGAAAATT<br>AAAATTAATAATTAATAGATTTTTTATTTTTATAAAAATTAAATTAAAATTATTTTATAAATATGTTAAAA<br>ATTAATTTAAAATTATTTTTGAGGAACACGTTTTAGTTTTTACATTAATTTGAAATCAAAATATAAAACCA<br>CGTCAGATGCGACAATATTAAGACCCTTGCATTTGGAATAAAAACATAAAATAAATAAAAGTTCATGATAA<br>TCTAAACCATGAATTAATGTGGAAAAGAAAGCGAGGTTGATTAATTACCCTGTAATTCCAAGGTGAATCAG<br>GTGATTAATTAGGTAACGGAAGTGATTTGGACGGAGACGAAGGTATTTGACGCAGAATTTAACGAATCAGG<br>TGGCGTGATTTACGGTATAATATGTGTGTCTGACATTAATTGCGGTGGAAGGTAGCTTTTGGATGTGATTA<br>CTTTGATGCCTGCACAAGTAAATATCCTTAAAAGGAAATGTAACTGATGGATTAATAATAATAGAAGCAAT<br>GAAAGTGATGGAGATGATCTCGTATATCGGGTACATAACATGGTGTTAATTATTTAGTTAGTTAAATGATA<br>ATGGCA
CrLEA6-2	TGGGTATACAGTCCATCGTTTGAATTAGGGATACCCAAAAATGGTATAAACAAATATATATATATATATAT<br>ATATATATATATATATATATATATCTTTATCTTTTGATTTTCTAGGGACAACTTCATAATTTTTCTACAAT<br>TCTTAGCTTCCACCAATTCAGAAACGCAAAATCAGTTGCTTTACCTTTGATAAATTTATTAAAAAAGAAGA<br>GCATTTAGCGTATTTAATGTACAAAAAGGAGGAAAAAAAAAACAGAACATTTTAAGACAAGGAAGAGAATT<br>GAATATGGAGCATTTGGTTTTATGTGCCTATGTAAAATGCCTAATACTTTGACATTGTAAAATTAAGATGT<br>AGGCAGAATTTGATATAACTATATATAAAGCTTTTCATAGAAAGTAATGCAATAGAGAGTGAGGCAGTAAC<br>AAATTTTGGCAATGTTACGCATTCACGATGTAAAATGTACCAACTATTAAGATTCTGCTTCAATTCGTTAA<br>ATATGAAAGGAATAGAGTGAATCGTGGGATGTAAATGATATCTAAAAATCTGCATTTTACAACAAATATAT<br>AAATATAAAGCATCTAATAATTTTCAGTTCTATTTAAAGATTGACGACAAAAATAGCTAAATTGGCCTTCA<br>CCAAAAAAAAAAGAAACACGTGTAGAAAAGAGGAAGTCATTGCACAGTTCCTCTCTTTTAATAGATAAGGA<br>GAGATAGAGGAAAAACATTGGAGAGTTTCAACACTGCATAAAAAATAATATCAGCATAAATAAAAGTAGGA<br>AAAAAGAAAACTCCAAAGAATAGGTGGAGGAGAGAAGTATGAGGACATTTGGGTGGTTCATCGATTATCAT<br>GTGGGTAGGAAAGGTAGGTTTGTCCTAAAGTTGCCACGTTTCCTGAAATGTCTGGAGGAGCAACACGTGTA<br>AATATAGGAAAATAAAAGTGTGTTATATATAAAGAAAGAATGAGGGAAGAGGGTGAGTCTAAGGAAGTGAA<br>AGAAAG
CrDHN1	CGTATGCAGTGTAATCATTCTAACCAGCCAGCTGGACATGATTTGAATTTGAGTAAATGTTATACCAACCA<br>CCACAACTCTCCATCCCCAACTTGTGTACAGATTCTCCCAAGGGATGAGTAAAATTATGTAAATTCAATAT<br>TTTTCTTTTGCATCTCAATACTTAGATGTCAGAAGGGTTGCGAGTTCTTGTAAAATTCTCTTCTATAAGAT<br>TTGCTTAGTTGTTATATTCAGAGGAAGAGGTTGCTGCTTAATAATTGCTTACACCTCATTGGTAGAACGGG<br>TGATATCAACATTTGCTATATGTTTCTTTCTTCCTACACGCATGATTCGTTCCTTTTCCATATCATACTGA<br>GAAGTTAGAATATTACAGCCATATAAAACTAGTTAAGTTGGTAATATATATTGAAAAATTGGAATATTGAT<br>GTAGAATGTGATCAGATTTAGCAGACTCTAACGCATTAGTCAGAAATGCGTTAACATTATAAATTTTTTAA<br>TCAAATTGATAAATTCTCAATTCTTTTTATAAGCAGATAAATTTCTCAATAAGGTCATACAATCGTACTAA<br>GATATATGTTAAAACTAAAATTAAACTTAGTGGATTAAATATATTATATAGTTTTTAATCTATTAACTAGT<br>AATAATGAAATATTCTGTTTTAGAAAGTATTTAAATAATGGTATCTGCAAACTTGTTTTTTTTACTTTACC<br>CTATACTGTTATGCATTATGTTGTGAGTAAGGGAGAATGAAAAAGGGACACGTTGCGGCCATTGCCTCAAA<br>TTATGTGTAGGTTTGGCATGGTCATGGAAGTGACGTGGCTATCACACAAGCAATCTCAAGTGACATTTGTC

	AACATTTGGTAGCAATTTCAGTGACTAGGCACCATAACTCCCTATCTGCATATACAGTGTGTAAGTATATA
	TAATTCACCTCGCTAGCACCAAAACTTTGGAACTATCGAATAGAAGGGGTGGTATAATTATAAATTAAATT
	AAATTA
	AATAAATAAAGAAAGTAATAGCATATGTTACTCATAAATTTAAATTGATCATAGCTTGTAAATGAATAAAC
	TCTTTTATATTTTATTTATTATTATTATTTATAAAAATTAAAATAAATGTTATTTTATAAAAAAGTTATAA
	ATTTAAAACTAAAATAAACAATATTTTCAATTTAAAAAATTAAAACGAAAGATTACTCATTTTTAAACCGT
	CCATCTTCAAAATACAACTATAACGTATGCATTGGAAACGACAAGCAGCACATGGAGCGGATAAAAACGAC
	GTCCCCATCTCGCGTGTACAATGCACCATTCTCATTGCACACAACTCTCCTTCTTCACGACGCAAGTGCGA
	CACACGCACCGACCTCACAAAATATATCCTTTCGAGACCGACATTTCCGGGTCCTGAATACACTCCCCACG
	TTATTCGTCCACCTGTCTCTTTTCTATTGGCTGCGTTACGATCCACCCATTGCACCGTGTCTCCCTCATCT
	TTTCCATCCTCTCCTTTTAGTTTCCATCCTCGCGCGTCCTCAAAATATTGCTTCACTCGCTCCATTCAGCA
	AAACCTCTCTCTCCCTATTTCTATGTTAGTCAAAAAAAGGACAGTCTATAAATTTGATTCATTGACACAAT
	TAATTGTGTTAAATTTTGTTTTTTGAAATTATTATTAGAGTATCTCATTAACTTTTTATCATAAATTTTTT
	TAATAAATAAACATATCTCCTAAACAGGTTAACACATTTAAATTTTTTATGAAAAATAATTTTAATAATTT
	TTAAAGTTTAAAAAGTTAATGTTTAAAAATGGAAAATAGAAAAACTCATTAACTTTCCAAATAATGTATAA
	ACGTTAAGATTAAGAAAGTGAATTATCCAATAATTGTGTTTTGTACAGATGCATCCTCATTGAGGCTTATA
	ATGGGGGCAAATTCATAGGCGTGATGCAGCGAGTGCATAATAGGCGCGTGGTGAACTCAATTTTATTTTCT
CrDHN2	TAACTT
	TAATTAAATTTGATTTTAATGCTTTAAAATTTTTGCATTAGTTTGAAATTTTATAAATTGTTATTTTTTAA
	ATAATTTCTGCCATCATTTAATCTAACAAGCATCTAACTATATTATCATATATTATCAACTTTAGTGATGA
	TATAAAATGTATCTAATTATCCTAACGAGTCATGTAACATGATTTGTTTAGATATAGTAAGTGATAATAGA
	GATTATTTATAAAGTTAATGAAAAAAATCTAAGACACCAAATGTCCATTTTTTAAAATTAGAAGTATATTA
	ATAATTTTTTAATAATAAAAATGAATAATATAAGTATTTATAAATTGTAAAAACATCTAAAGAACTACAAT
	AATATTTTTTTAACATATTTTTTGAACTTGTTAAATTTTAAAAATGTATAAAGAACAATCGATTTATAAGA
	TGAGTGTTATGATTTTTTTAAAAATTAACTAACTACAAAAAGTATGCTGCGTCGTTTGCATTTCTTAAAAT
	ATCGTTAATAAAAAAATAATTAAAAAAATATTATTAAGTATATAAAAAATAAAAATAAAATAATAACTTTTA
	TTTTATAATTAAAATTTTGAAAATTAATAAAAAAAATTTAAAATAATATTATTTATTTCATTTAATTATTA
	AAATATTTAATTCTATCAAACAGAATTAAAATTATCTTAAAAAAATTAATTTTATTTAAATTCAGTACTTG
	CTAAACACATTCTAATAAAAATTATATATTAAAAAATACATTATTTATTATATCTTTCAATTTTTTTTATT
	TTATAGTTCTTTGGAATATTTCCCTCACGATTTTTCCGTATAACATATAGGCCGTGGCCTTACAGAGGGAC
	GGGTGTCTTCATCTTTTTTACGTGGCAACTGGTGTCTGTGTCTTCTCGTGCCACGTGTTGGCGCCAAGTTA
	CATGCATATTTCCACCGCCACGGATTCTGCGGTTTAATAATTAAGAGGAACCAGAGTGAATCAATTCCAAA
CrDHN3	TTATAT
	TATACTTAAAGTGTATATAAATTTTAGATGTAATAAAATTCACTACATCTATCAATTTTTTTATTTTATTT
	TCACTCAAGATATATACATCCAAAATTTAATAAAATAGTTAATTTTTATTAAAAATTATAAATATTTATTA
	TTATCAATATATCAAATTATATAATTTTTAATAAAAAATTACTATATATAAATTTTTCATACATAAAATAT
	ATTAGAAAATTTAAAATAATAAAATTAAATTAGTAAAATCAATAGTTGATTAAAATTCATCCTGGCTAGAA
	GGCTTGCTTTAGGCCCAATTGGATTCGGATTGAGGAAAATCCTTTTTTGTATTGCAGGGAAACACTTTACA
	CATTTCTTGTATTAATCGCACCATTTTTTTTTATCAATTTCAGAAATTAAATATCAGAATATCTTATATAT
	TTGTGTGAATATTAATTAGATATAATTATATAATAATTTTAATATAAATAAGAAGGTTAACTAATTTATTA
	ATTTATATAAAATAACTTTAAATAACAGATAAAAATACGAAGCGAGTATAAGTTATATTTGTCATGTAATT
	TACTTAATTACTAGTACCAATGAAAGCCAAGAAAAGGTTATCATTAATGATTAATTAATGAAGTTTTATGG
	TAGAAGGAGGAGGGGAAGCGAAAGGGTTGGAAAAGCAAAGATGTATGTAGTGGTTAGAAACGACAAATATG
	AAAGAGATAAAAGTGAGGCCCCACGGTGACGGTGACGAATCAAGTTTCCACCATGCATCTGCCACGTAGCC
	TCACAGCACACCGTCCATACCGACATCTCCGGTCCCACATAGTACACCTCCCCATGTCTTGATTACTTTAC
	TTTAGCTTTGAACTCTGCAATTTTGACTCATTTGTTATGTCGGTCAAGTTACACATATTCTACTCTCATTG
	GCCTTTAACTTTCTTAATCGTGTAAGATGCGCGTAACTCCAACTATAAAATGCTGTCCTAACTTGCATTCT
CrDHN4	TCAGAT
	ATCATTTTTTATTTTTTTATTAACTTTATACACATTTTCTTTAATATTTAATTTTATAAAATATGTTAAAA
	TTTCTTCCATTTAAATATTTTATTATATTTTTAAAGTTATAAATTAAGACGAAAGAAATATAAAAAAATTA
	AAAATTAAAGTGACAAGTTAATCTTAACCCACCCCTGAGATTTCAGGTTTATTGGTTTTGTAATGAAAAAT
	ATAGGATTTTGTTACTTTCCCAACAATTTCTTCATAAAGGTAGGATATTTTTTTCCAAGCACTTATTTGTT
	GAGGAGAAACTGATCTAATTCGAAGTTGTTGATGTTTATATTATCAATCATAGAATCAAAATTCTGATTAG
	GTCCATCCAATTAAGTTTTATGTATTTAATTATTACTGATTAACCGTATAAAAATATGATTAATAAGTGAA
	AAATAAAAAACATAATAATTAATCAATTTAAAATATAATAATTTAAATTATTTATGTGATTAATCGATTAT
	TTATATGATTAATTAATTATATGTGTAGTTAATAATATAAAAATATATATTAATTAAATTAATTATTAAAG
	ATGATTAATTGAATATTAATGAGTGTAGGTGGAAGGAATCAGCGATTTTTGTCACAGTTGCTGCGTTTTGA
	ATGATACAAAATGGAAAGTCACTAACCCCATCAAAATTCATATCACCTTATCCATTTGAGCCTTTCTGCTC
	ATCCACAAAACTCTGGTACGTGTAACGTGTCATCCTATGAATTCGAAAGCGACTACATGGCTTTCAAATGA
	AAAGGCAGCAACACGTGTCAATGCACGTGGCAAGTACATTGCCAACGCCACATGACACGTGTCATACCATT
	AAACAATGAAATAGGATTCACCGACTTGCTCATTGACCAGATTTTTATGTTCCAGTTCCCTATATATACGC
	CATGCTCCCAAAACCCAACCACACAATCATCACAACAAGTTCTGTCATTATAACAGTAAAAACCGAAAACA
CrDHN5	GTTACT
	TCTATAAAATGTTTTAAAATATATGCTGGAATAGGTGAATTTTGTAGTAGTGATTCAAAGCTTGCCTCAAA
	TGGAGAAGGTATGGGTATACTTCCATCAAGAAATCGAAACTTATTTTTCGCTATCAAAGCACGCTTAATCG
	ATCTCGCTTAGACATGATAGTTTCTTCCATCTACGAACCATGGAACCATCACGGTTAAGGGCTAGACTTAT
	TCTATGTAGGATCCATGTTTGCTTGATTTCTGGCTACATGGGAATTTCCCTATTGCTATATATATATTTAG
	GTAAACTATTGTTTTTTCAATAGAACTAGCACCCATCGTTATCACTAAATATATATATATATATATATATA
	TATATATAATAGTGTTCCTCTAATTAATTTCAGCATTTGGTGGCTTCCTCTAACTATCTTAGGCCATAGAT
	AAAAACATCTAACATTTTCCGCTGTCTCTCTGATCTCTTTCACTCGCTCACATAGATACGTTGAAAATAAT
	GAAAAAGAATTGACACCTCCAGGTTTAACGGAGAACCAGTTAGATCTTTAGAGCATCTCCAATGGAAGTTG
	TTTAAGGAGATTCTTAAATTTTAAGCTTTAATGCTTATTTTTTTATTATTGGAGCTGACTTACTTGGAAAA
	ACTGTTAGAAGAGAGTTGCTTAAATAAGCAACTATTGCTTATGAAAAAAATGAATTCATTTTTGTTAGTTT
	TAAATAATAATAAAAAAAAATTAAAATAAAAATATTATAAATATTAAATATGGAATAGTATGTGAAATATA
	TTTAAAAGTTTTATAAAATTATTATGCTGAAGTGAAAAGTTACTTAAATTATAAAAATTAAATAAATAAAT
CrSMP1	AAATATATATATATATTTGTAATCTACAAAAATAATATTAAAATAATTTAAAACAACTCTTAATCAAATTC

	TAGGTTGAAGATGCTCTTAGAATTTAGATAAATATAATACATTGTTTGTTACACAGCCCAGTGGGTGGAGG<br>TCCATA
CrSMP2	CTCATAGATATCAAAAACACGTTCCATCAACTTCCATTCCATTTATCCCTGTACGATTTTTTTAATACTTT<br>TTTAAAAAAATATTATTAACCGTTAAATTTAAAATATAAATAACTAGAGAATAATTTTCATATATAAACCC<br>ATCATTATTCTAACATAATTGACATCATATTTTGCGTACAATGTTTTTTTATCTCTTTTTTTTTTTCCTTTA<br>TATGTACGTAAGTCTTAAAAGTTAAAACCACATCTCCAAAAATTAAAGACAACAATACAAATTTCTCACTT<br>TAATTATAAATACAGTATTTACCATTCGGCATAGGTTACTTAAAAGTAAGGTTTAATTTTGTTTTTAATTT<br>TCATATTTTAGTCTATATATAATTTTAATTTATCTAGTATTTTTAGTAATTCTAAATCATCTAATTTTAAT<br>TTCTTATTTTCATTTTTGATTATTCAAATTAAGTTTCCATCCAAGTATTTCTTTTAAATAATTAATAGGAA<br>TTAACGAATATGTTATATGTCATGTGTTATTATGTTCTGTCAATAGTTGGAGTTATTACTTTAATTGTATA<br>AAAAGTTATTTTTTTATAGATTTATAATATATATAATATTTAAATCAAATATTTTATATTCACAAATGTCC<br>ACAACACAAATATATTTATTATATTTTTGCTTATTAGATAAACTTTTAGAGTTTAAAATATCCTAATTTAA<br>ACAAATAAAAAATATGAAAAATTAAAATAAATATAAATAATAGACTCAGTCATGATGGAAACGCTTAAACG<br>ACATGTGTCTTCGGTTTCTCAATCGAAGGTCAAAAACTCAACGGTAGAACCACAAAACAAGTCCTTGTTTG<br>GCGCCAAGTGTTGCTGAGAAGCTTACCGTGTTTCTCTCCAATGTTAGCCACGTGTCAATTCCCAACCATGC<br>ATTTACAATCTTAATCCTTCCTATCCCATTTAAAAGTTCCCATCCACTTGAAAGCACGCATTATATTTAAA<br>TAAAAA
CrSMP3	CATAAATTTTTTTCATTTTTCTTTTCTTTTTTGAACAATGAAATTTTATAAAAAAATAAAATGTAAATTTA<br>AACTTTTATATAAACTATTCAACTTTCTACTATACTAACCTGATACTGATCTTAGATCATTTGCTTTTTTT<br>TTTTTTCAAACTATGGCAAAATGATAACTGAATCAAAAGAAAATATAAAAGCATGGTATTAAAATAATTTT<br>ATCACCCCTCTCTAAAACGGATTCGTAGACCTTTCTAGCTTAAATAATTTTGCATACATGTTTTCATTTCC<br>AGTGTACACATTATTTGTGATAATACATATCAAGCTTCTTTATTTGAACGAGTCTTAAAACAAACAACAAT<br>TTTAATTTTTTATACTCATATATTTATGTTTATTTATATATTTTTCCATCATCACATCATTCGTTACGTTT<br>ATATCCCCCCCCCCCCTTTATGTCTTGTAAGCTCTCAAAAGATATATACATTTTATTGGATACGACTGAAA<br>CATCTTCCAAAGGAAAATGTGAGCGAAAACATACTACTGTGGAAAAAGGCAATCAAATGGAAATGGGACAC<br>TAATTTATTTTATCATTTTTTTCACGAAGGTATAGAAATATCGGGAATATCTTAATGGCATTGTCATTGAC<br>ACCGCCAAATATATTAGAGAAATAAGTTTTAATTTTGTATTTATTATTAATAAATATATATATATATATTT<br>AAATTTTATAAAAAAAATTAATTTAATAATGATATAAATATATATTTATATAATATTTGAGTGTTTCTTAT<br>ATTTATTTATTTTTGTTTTTGAATAGTATTATATTATTATTTTGTATCCCTTAACCGAAAAATGGTGTAAA<br>ACAATAATTGTAAGGATTGGTATTGGATATTTTTGTTGAAAAAGGGAAAAAAGAAGCTAGATGGTTGTGAA<br>ATGAGCATGCCACGTCCCCTGGTACCACTCTTGTTGTTGCCAAGTGTAACGCTTGGCCACGTATTCTCCCG<br>TTCCCA
CrSMP4	TTATTTATTCATTTGAAATCAGTGAATACTTGGCAAACGCAAAACCATTGGAAAATGACAATTGAGCATCT<br>TCTAGATATTCCAACAAGACAAAATCGTCAATTTAAACCTTGAGAAAATCAACCACTGCGAAATGACTTAA<br>ACTGGTTCCGCCAAACTTTACTCCCAATTCCATCCATTAAAACAAATTAAGATCAGAGCAATACAATAAAT<br>TAATCTCTAAAATCATAAATATTAATTAATTAGTCTTTAAAATTATAAAAAATAATAATTTCTTAAATTAA<br>AATATTTTATATTTAATAATTTTAAAAATTAAAATAATATTTTTATAATTTTAAAATTAAATTAACTTATA<br>TTTCTAATTTTAGTGACTAATTTATTAAATTATTTTATATATATATATATATATATAAAAGTTAAATTTGT<br>TGATTTTTAGCATTTACATTAATTTAATAATTTTATATAAAATATAAAATTAAATTAAATTTTTTATAATA<br>TTTAATAAATTATATATATAAAATTTTATATAAATAAAAATTAATAAATTTAATGATTTATATATTTAAAT<br>ATATATATATATATAAATATTAATAATTGAATAAACTATCATTTTTAAAAAAATTTAAATTTAATATACAT<br>AATTTATCAAATATTATAATATTATACAACGGTTAATAATTATATTAATAAATTTTATATTTATATAAAAT<br>ATTAAATTAATATAAGTATCTATAATTTAACATCTTCTATTATTGGATTTTTCTCTAGCTGAGAAGACGTA<br>CGTTTCGACCCGTAAAGTGAATATATTTTTATTATATTTTACATTATATATTTTTTTTATCAGTATCCTTC<br>TTTTTGAATGGTACAACCAGAAGCCAGATGTATGATCAAAGTGATCTACACTTCATAAACAGTGTCTCCTG<br>AGGTGGACTTAGAACGAAATGCCACCTTCACCTTTTGTCTGTGCACAAACAACACGTGTTCTGCATGCACC<br>ATCCAA
CrASR1	AGAAGACAACAGACGAAAACAAATGTGTTATTATATTATATTATTATAAAATTTATAATAATATCTCTAGA<br>TTAAATCTATTTAATTTATTTAAATCTAATTCTTAAGACGCATATGTTATTACTTATTTCATATTAAATAA<br>TTTTTATTAAAATGAGTTTTGCATATAAAATCAATACTATTTTAATAAAGATTAAATACATTAATTTTATT<br>ATCTATAAAAAAACAAATTGATAAATTAAATGGATTGTAATAGTTTAATAAATAATTTTATATTTAAAAAT<br>TTTAAAAATAAAGGTTGACTAAAATTGTAATAATTATAATAAATTAACCTTTTTAAATGGGTTAACTTTTA<br>ATGGTGACATTGTCGTTTTTTTTAATAGATAAAAAATAATTATTTTTAAACATTTATACCTGTTGTTACAA<br>AGAATTAAAATAAATATTTTTCTTTTAAAAATTTAAAAAACATTTTTTAAAATTCACATGATTTATCATAA<br>ATTTTTTAAAAACTAAAATCATGGTCACTTTCTTTCAATAATAGGAGAAAAAGGAGTAGCAAAATTTGTAT<br>TTTAAATTTATATAATAATTTATAGAATATATTTTTTATTTATATATCTTATTATCATATTATTTATTATA<br>TTTTATATTGCACTCTTTTTTATTTTTTGTTTTCCTATAATATATTATAACAATCTTTTGATACGGAATTA<br>TAGATTTTTATTAAAGATAATTTTATTTAAATAAAATTAAATTATTAAAAATTAAACTTTCTTTAAGATTT<br>CAAAGCAAATGTATATTCAACAAAATTTGGAACCGTTTCTTTCAAGTACAAGTAGAAATGTTTTTGCAAAG<br>AAATTAAACACACAAAGTATGTGGAGGACCTATATTTTGCTATCTTCCACGAGATCATTTAAAGCCTACCA<br>AAAAAGCAGAAACTTAATCAGCATGTCTAACATGATCATCACTTTATAATAAAGAACAATTATATTCATAT<br>ATTTCA
CrASR2	AATAATGTTATAAATTAAGAAGCCAAATTTTATCTGTAAAACAAATAATAATTTAATAGTCGCATAAAATA<br>CTTTTGGGAAACTACCTTGCTCGTGCGATTAGCCTCCAAGGAAGTCAACACATAAGTATTTCCCTAGTTTA<br>GGTGACTTCCAGTTCCAAACTACACTCGATCGTAACTTTTAATTTCACCATTATCTTAGTATAGAAGAAAC<br>CAATTTTTATAAGAATTCTGTGTCTTTCAAAAATATAAGAAATTACAAACAAACTCCAGCATAGCAATTAC<br>ATAAATAATTATGTTTTTTAAATATACTGATAGGGATTTAAATTTGAATTATATATATATATATAAATTAT<br>ATGAAAATAGTAAAATTTAATTTAATAATTTTTATATTTTAAGAAACTAATCTTTAACTATAGCAAATAAT<br>TGTATGTTGATAAAAAAAATTGAATAAATAAATATGTTAAATGTACAAAAACAAATACCCATCAAATTTTC<br>AGAATGTGTTATTTTATTATTATACTCAAATTTTCTTAATATAAGACAGAGTGATATTTTGCAAAAAGAAA<br>AATATAAAAGCATTTATAGTAAGAAAGTCATTATATATGTAAAGTTTATGAATATTTTTGTTAAAATTACA<br>TTCACATCCTCTTAAATTTATAAAAAAAAAAAAAGTATTCGGTGTTTTTTCTGTTATTTATCCTGCACATA<br>ACACTGTATATATTTTTCCAATTATAAAACTCTGTCAACACTTTCATAATTTCTTATCATATTACATCTAT<br>AAAAAAAATACCATATATTTTTTCAATAAATTTTAGAAGATGTTAACATAATTTATTTTTTTTCTATTTAAA<br>TATTATAGAAACCTAATATTTTTCTCCATTTTAATATTCTTTGAATTTGGGCATTGAATCATTCTTGGGAA<br>TTTCCATGAGACTTTTCCACATACCGGCAAACAATACATTCTTTATCACCTATAAGTAGCTTAATTTCATT

	CACAGA
CrASR3	ATTATTTAATACAATAAAGTGATTAATAAACAAAGAAACAACAAGAAAGAGAAGTTTGTGATAAAATGATG<br>GATAGAATAGAGAGAACTAGAAAAGAAAAATAATGCTATATAAAATATTGTATAAAAGTTATGTAGGAATA<br>ATATAATTTATAATTTTATATTTAAAAATAATTTTTTATTCAAGAGTAGTTTAATCTTGAAAAGTGATCAA<br>AGAACATATTTGGGTTATATTCTAAAATCAAAACTGGTAGCAAAACACTCTTTTGAATATATTATATATTT<br>AACATACTTTTTTATAATAGATTTTTTTGTCTTGATCAATAGTGCTCTTAGGATACTCGTTAAAAAAATTA<br>AAATGAGAAATTTTTATTAAAATTAAATTTGAAATATATTAAAATCATGGCACTTGTAATTTAATAATATT<br>TTTTATTTTTTAAAAAAATCAAAACATTTTATACAAATTAATCATTTTACAAATAAAATATTTCAACTTTTT<br>AAAAAATAAATCAATAATAAAAAATATATTTAAAAGAGTGTCCTTAGTCCTTACATTTCTGGTTCTGAAAT<br>GTAAAAATCTAAACCCACTCGGTACTCCTGTACACATATCATGATCTCTGCTTCCATGTGCTCCATTTGTT<br>CAGTGAAAAGGCTTCTTATACTGTAGCCAGTGGCCAGCCCCATGGAAAAATCGTCATTGATACCCACTGTA<br>ATGGAGATTTTTTCTCATCTCCATGTATCCTTTACTTAATTGGGTAACTAAAACAATCCATTAATTAATTT<br>TCTTAATTTATATTTTATAAACTCCTAAATACTATTACTCCAAAGTTAAAAATTTAATGAATATACAAAAA<br>CATAATGATATTTAGGGGACCCATATTTATAAATCTTTCATAAAATCATTTATCATGGCCCACCAAGAAAG<br>CTGCACTTAACCAACCAATTACTCATGGTTAACATAATCATCAATTTATAAATAGGTTACCTAAGCTTCCC<br>AATTCT

Table S3. The conserved motifs of the CrLEA proteins.

Group	Motif	E-value	Consensus sequence
CrLEA_1	1	3.0e-032	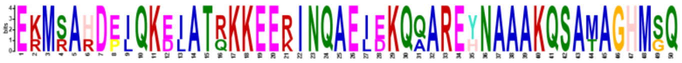<br>EKMSAHDEIQKEIATRKKEERINQAEIEKQQAREYNAAAKQSAMAGHMSQ
	2	1.3e-016	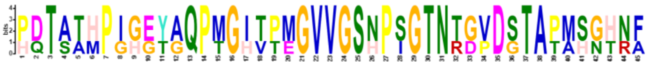<br>PDTATHPIGEYAQPMGITPMGVVGSNPSGTNTGVDSTAPMSGHNF
	3	8.5e-010	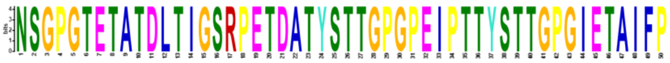<br>NSGPGTETATDLTIGSRPETDATYSTTGPGPEIPTTYSTTGPGIETAIFP
CrLEA_2	1	1.0e-113	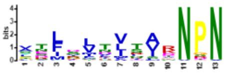<br>VTLDLTVTARNPN
	2	2.2e-167	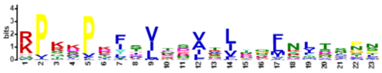<br>RPKKPKFSVTSVTLTQFNLTSPN
	3	7.9e-193	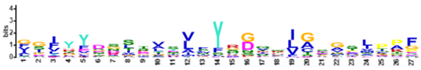<br>GGIYYDSSTVSVFYRGQQIGSGSJPPF
CrLEA_3	1	7.5e-034	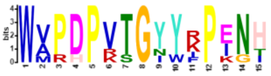<br>WVPDPVTGYYKPENH
	2	9.8e-014	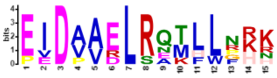<br>EIDAAELRQTLLNKK
	3	4.5e-006	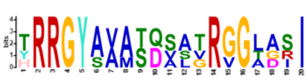<br>TRRGYAVASDSATRGGLASI
CrLEA_4	1	1.9e-004	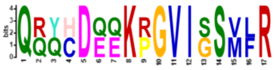<br>QRQCDZZKRGVIGSMFR

	2	4.4e-003	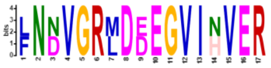 FNDVGRLDEEGVINVER
	3	3.6e-001	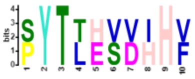 PYTLEVDIHF
CrLEA_5	1	6.7e-007	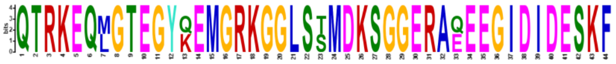 QTRKEQLGTEGYKEMGRKGGLSSMDKSGGERAZEEGIDIDESKF
	2	1.2e-004	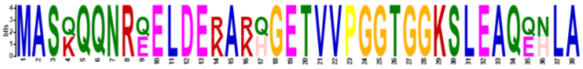 MASKQQNRZELDEKAKQGETVVPGGTGGKSLEAQZNLA
	3	1.2e+001	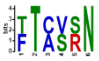 FTCVRN
CrLEA_6	1	6.6e-002	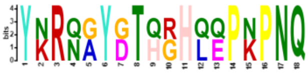 YKRNGYGTQGHQZPKPNQ
	2	1.2e-001	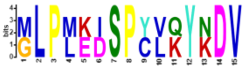 GLPLEDSPCLKYKDV
	3	6.2e+001	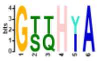 GSQHIA
CrDHN	1	3.6e-017	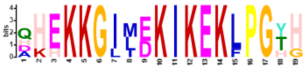 QHEKKGILEKIKEKLPGYH
	2	2.6e-007	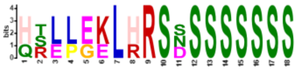 HSLLEKLHRSBSSSSSSS
	3	5.3e-007	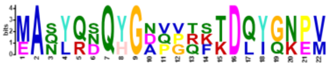 MANYQBQYGDPPRSTDQYGNPV
CrSMP	1	1.1e-026	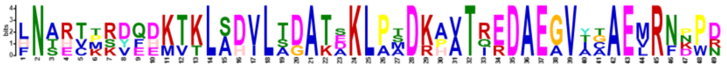 LNARTPRDQDKTKLADVLTDATEKLPSDKAVTREDAEGVIAAEMRNDPD
	2	3.1e-013	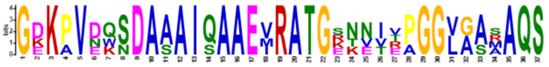 GDKPVDQSDAAAIQAAEVRATGKNNIVPGGLGAAAQS
	3	1.3e-011	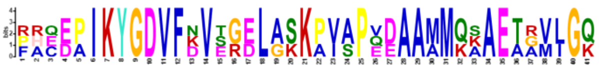 PRQEPIKYGDVFDVSGELASKPVAPZDAAMMQAAETGVLGQ

CrASR	1	2.0e-018	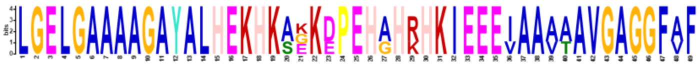<br>LGELGAAAAGAYALHEKHKAGKDPEHAHRHKIEEEIAAAAAVGAGGFAF
	2	5.4e-002	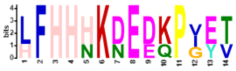<br>LFHHHKDEDKPIET
	3	6.5e-001	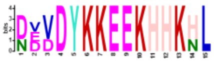<br>DDVDYKKEEKHHKHL

Table S4. The *cis*-regulatory elements identified in promoter regions of *CrLEA*s and *CrASR*s.

	light responsive element	gibberellin-responsive element	MeJA-responsiveness element	auxin-responsive element	salicylic acid responsiveness	ABRE	ERE	MYC	MYB	MBS	TC-rich repeats	LTR	as-1
CrDHN1	6	0	2	1	0	3	0	3	2	0	0	0	1
CrDHN2	7	0	0	2	0	2	0	1	0	0	0	0	0
CrDHN3	10	0	0	1	1	4	4	1	1	1	0	0	0
CrDHN4	10	0	4	1	1	1	1	2	1	0	0	0	2
CrDHN5	28	0	0	1	0	10	1	3	3	1	0	1	0
CrASR1	9	1	0	1	0	0	4	3	2	0	0	0	0
CrASR2	4	0	0	0	0	0	0	0	0	0	0	0	0
CrASR3	10	0	2	0	1	0	0	2	3	0	0	0	1
CrLEA1-1	9	0	2	1	1	8	0	1	2	0	0	1	1
CrLEA1-2	3	0	2	0	0	0	1	3	1	0	0	0	1
CrLEA1-3	6	0	0	0	1	1	5	0	0	1	0	0	0
CrLEA2-1	11	1	0	0	0	1	3	1	2	2	0	0	0
CrLEA2-2	6	0	0	1	0	0	0	0	0	0	0	0	0
CrLEA2-3	4	1	2	0	1	2	0	4	4	0	0	0	1
CrLEA2-4	3	0	2	0	0	0	0	3	3	0	0	1	1
CrLEA2-5	3	2	2	0	0	0	0	3	0	1	0	0	1
CrLEA2-6	6	0	2	0	0	1	0	2	1	0	0	0	1
CrLEA2-7	8	0	2	0	1	1	3	0	2	0	0	1	1
CrLEA2-8	4	1	0	0	0	0	0	0	1	1	2	0	0
CrLEA2-9	14	0	0	0	0	1	3	2	0	0	0	0	0
CrLEA2-10	10	1	0	0	0	0	4	0	0	0	0	0	0
CrLEA2-11	11	0	0	0	0	0	1	0	2	0	1	0	0
CrLEA2-12	6	0	4	1	0	1	1	1	3	0	0	0	2
CrLEA2-13	2	1	0	0	0	0	1	2	3	2	0	1	0
CrLEA2-14	4	1	0	0	0	0	2	2	2	0	0	0	0
CrLEA2-15	9	0	2	0	2	1	3	1	2	0	0	0	1
CrLEA2-16	8	1	0	0	0	3	0	2	2	0	0	0	0
CrLEA2-17	7	0	0	1	1	0	1	1	2	0	0	2	0
CrLEA2-18	11	1	0	0	0	1	1	0	0	1	0	1	0
CrLEA2-19	6	0	0	0	1	0	0	3	5	1	0	1	0
CrLEA2-20	4	0	0	0	0	1	1	1	1	0	0	0	0
CrLEA2-21	9	1	0	1	0	0	7	1	0	0	1	0	0

CrLEA2-22	3	0	0	1	0	0	0	2	1	2	0	1	0
CrLEA2-23	8	0	8	0	0	2	2	3	1	0	1	0	4
CrLEA2-24	10	0	2	0	0	3	0	0	2	1	1	1	1
CrLEA2-25	4	1	4	0	0	0	0	1	1	0	0	1	2
CrLEA2-26	8	1	2	0	0	0	0	0	3	0	0	0	1
CrLEA2-27	12	1	0	0	0	1	7	0	2	0	0	1	0
CrLEA2-28	10	0	2	1	0	0	3	0	2	0	0	0	1
CrLEA2-29	11	1	4	0	0	1	0	0	4	0	0	0	2
CrLEA2-30	8	1	0	0	0	1	0	2	2	0	1	0	0
CrLEA2-31	7	0	2	0	0	0	1	1	0	0	0	0	1
CrLEA2-32	5	1	0	1	1	0	1	1	1	1	0	0	0
CrLEA2-33	6	0	2	0	1	1	2	3	1	0	0	0	1
CrLEA2-34	7	0	0	0	0	0	0	0	0	0	0	0	0
CrLEA2-35	4	0	0	0	0	0	1	1	1	0	0	0	0
CrLEA2-36	12	0	0	0	0	1	2	0	0	0	0	0	0
CrLEA2-37	2	1	0	1	1	0	6	0	0	0	1	0	0
CrLEA2-38	11	0	0	0	0	0	2	1	0	2	0	0	0
CrLEA2-39	13	0	0	0	0	1	2	1	1	0	1	1	0
CrLEA2-40	3	0	1	1	0	1	3	3	2	1	0	0	1
CrLEA2-41	4	0	0	0	0	0	8	1	0	0	1	0	0
CrLEA2-42	6	1	0	0	0	1	0	1	2	0	2	1	0
CrLEA2-43	11	0	0	0	0	0	2	1	2	0	0	0	0
CrLEA2-44	6	0	0	1	0	1	6	2	0	1	0	0	0
CrLEA2-45	11	0	0	0	0	0	0	0	4	0	0	0	0
CrLEA2-46	7	0	0	0	0	1	1	2	3	0	1	0	0
CrLEA2-47	5	1	2	0	1	1	2	3	4	0	1	0	1
CrLEA2-48	8	0	0	0	0	1	0	0	0	0	0	0	0
CrLEA2-49	3	0	0	0	1	1	0	1	2	0	0	0	0
CrLEA2-50	7	1	2	0	0	2	3	9	1	0	0	0	1
CrLEA2-51	5	0	0	0	0	1	2	5	1	0	1	1	0
CrLEA2-52	5	0	0	0	0	1	2	1	1	0	1	1	0
CrLEA2-53	11	0	0	0	0	1	3	3	3	0	0	1	0
CrLEA2-54	4	0	0	0	0	0	0	4	0	0	0	1	0
CrLEA2-55	3	0	4	0	0	2	2	1	2	0	1	0	2
CrLEA2-56	7	1	2	1	0	0	1	1	0	0	1	0	1
CrLEA2-57	9	0	0	0	0	1	0	0	1	0	0	0	0
CrLEA2-58	11	0	4	0	1	0	1	2	1	1	0	0	2
CrLEA2-59	12	0	4	0	1	0	1	2	1	2	0	0	2
CrLEA2-60	4	1	4	1	0	0	1	1	0	0	0	0	2
CrLEA3-1	10	0	4	2	0	2	4	2	1	1	1	0	2
CrLEA3-2	7	0	0	1	0	0	0	2	2	0	0	0	0
CrLEA3-3	8	0	2	0	0	0	1	3	0	0	0	0	1
CrLEA3-4	4	1	2	0	0	0	11	1	4	0	1	0	1
CrLEA3-5	4	0	0	0	0	0	0	4	1	0	0	0	0
CrLEA3-6	2	0	0	0	1	0	3	1	1	0	0	0	0
CrLEA4-1	11	1	0	0	0	5	4	0	2	0	0	0	0

CrLEA4-2	5	0	4	0	0	1	1	3	3	0	1	0	2
CrLEA5-1	18	0	0	1	0	8	0	1	1	0	0	0	0
CrLEA5-2	19	1	0	0	2	4	0	1	2	0	0	0	0
CrLEA6-1	14	0	4	0	0	2	2	1	0	0	0	0	2
CrLEA6-2	11	0	2	0	0	6	0	3	0	1	0	0	1
CrSMP1	5	0	0	1	0	0	1	2	0	0	0	0	0
CrSMP2	10	1	0	1	0	4	2	3	2	0	0	0	0
CrSMP3	5	0	0	0	0	2	0	2	1	0	0	1	0
CrSMP4	13	1	2	0	1	3	4	2	3	0	0	0	1

Table S5. The numbers of subfamilies of *LEA*/*ASR* genes in different legume species.

Species	Total No.	The No. of LEA/ASR members in each subfamily								
		LEA_1	LEA_2	LEA_3	LEA_4	LEA_5	LEA_6	DHN	SMP	ASR
Canavalia rosea	87	3	60	6	2	2	2	5	4	3
Phaseolus vulgaris	82	5	55	8	2	2	1	3	4	2
Glycine max	149	7	105	11	3	5	2	7	6	3
Medicago truncatula	103	6	72	6	2	2	2	4	6	3
Cicer arietinum	72	5	47	4	2	2	2	2	4	4

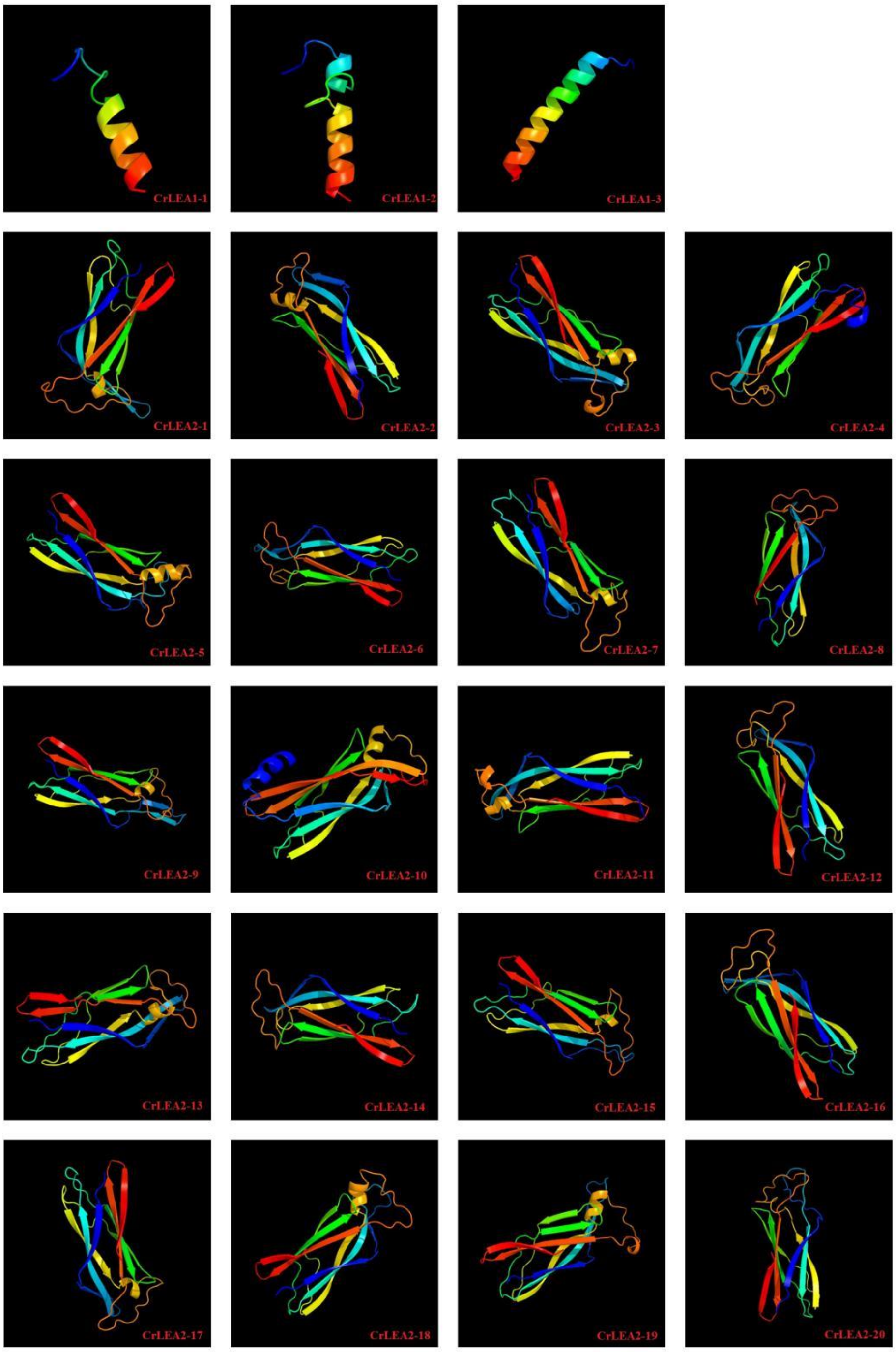
CrLEA1-1
CrLEA1-2
CrLEA1-3
CrLEA2-1
CrLEA2-2
CrLEA2-3
CrLEA2-4
CrLEA2-5
CrLEA2-6
CrLEA2-7
CrLEA2-8
CrLEA2-9
CrLEA2-10
CrLEA2-11
CrLEA2-12
CrLEA2-13
CrLEA2-14
CrLEA2-15
CrLEA2-16
CrLEA2-17
CrLEA2-18
CrLEA2-19
CrLEA2-20

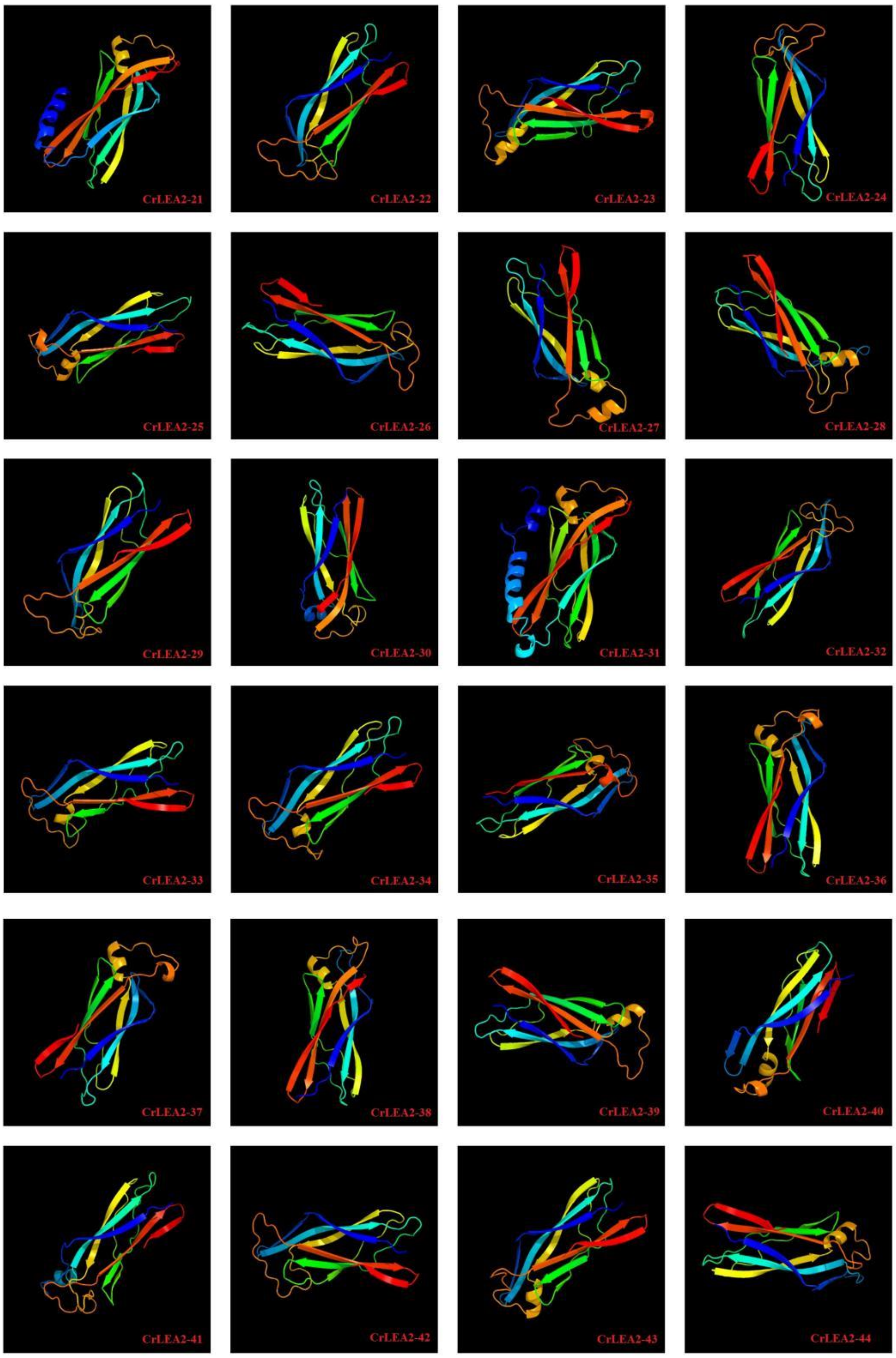
CrLEA2-21
CrLEA2-22
CrLEA2-23
CrLEA2-24
CrLEA2-25
CrLEA2-26
CrLEA2-27
CrLEA2-28
CrLEA2-29
CrLEA2-30
CrLEA2-31
CrLEA2-32
CrLEA2-33
CrLEA2-34
CrLEA2-35
CrLEA2-36
CrLEA2-37
CrLEA2-38
CrLEA2-39
CrLEA2-40
CrLEA2-41
CrLEA2-42
CrLEA2-43
CrLEA2-44

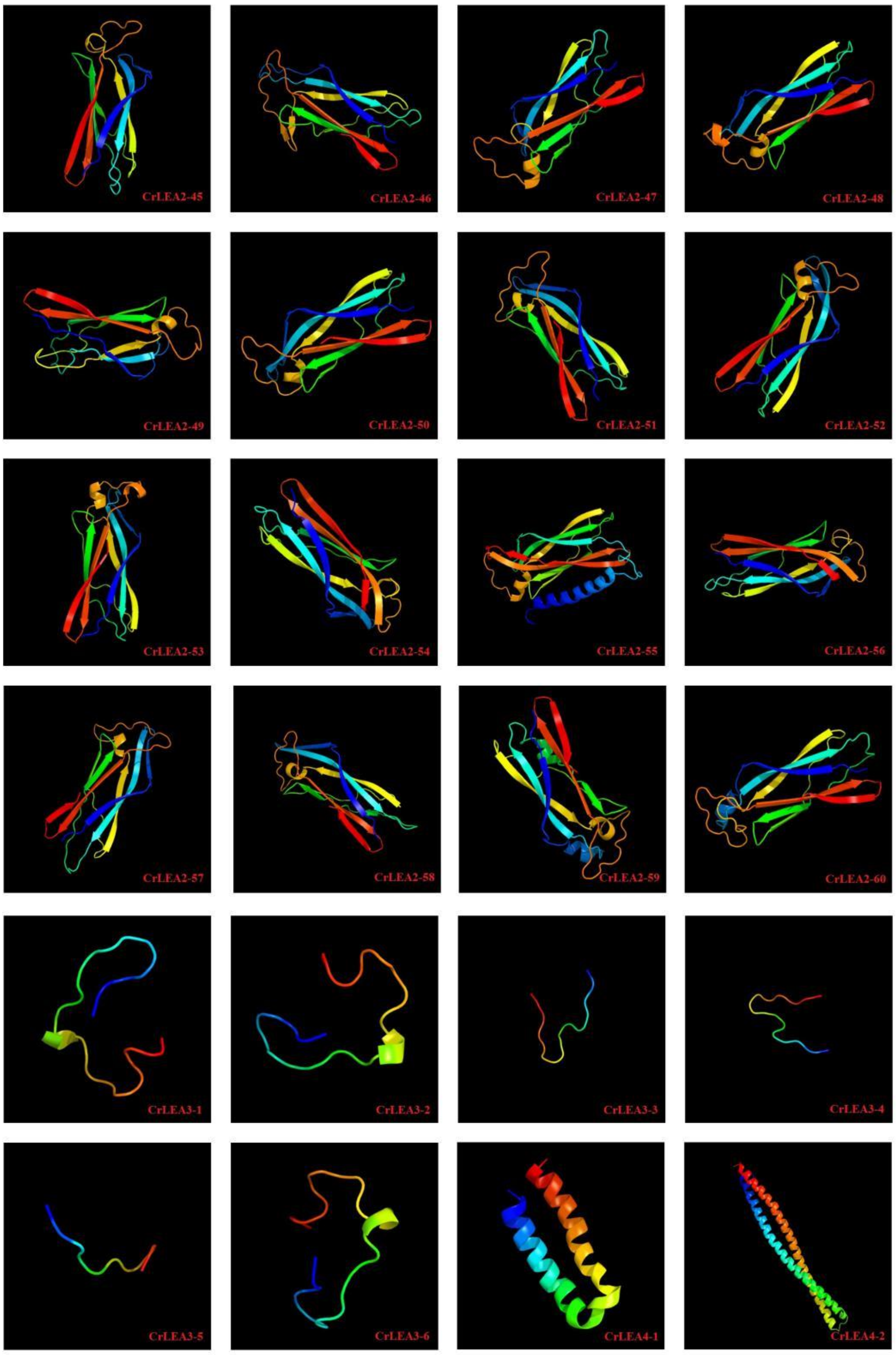
CrLEA2-45
CrLEA2-46
CrLEA2-47
CrLEA2-48
CrLEA2-49
CrLEA2-50
CrLEA2-51
CrLEA2-52
CrLEA2-53
CrLEA2-54
CrLEA2-55
CrLEA2-56
CrLEA2-57
CrLEA2-58
CrLEA2-59
CrLEA2-60
CrLEA3-1
CrLEA3-2
CrLEA3-3
CrLEA3-4
CrLEA3-5
CrLEA3-6
CrLEA4-1
CrLEA4-2

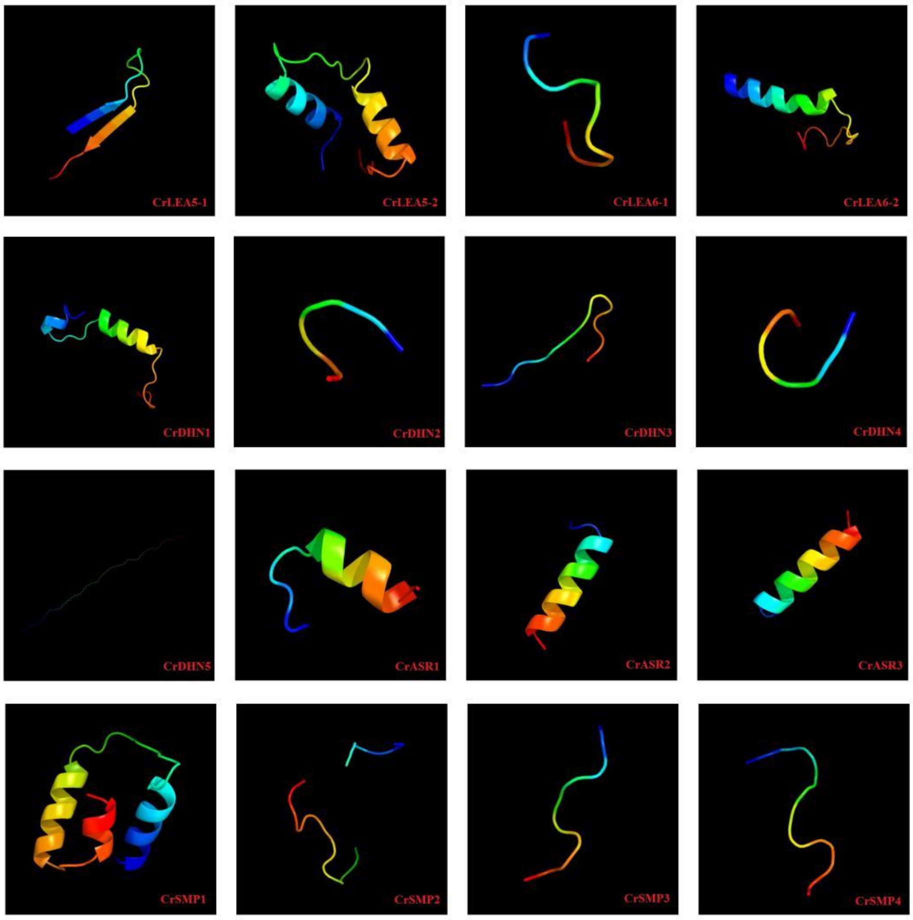

Figure S1. Predicted 3D models of CrLEA and CrASR proteins.

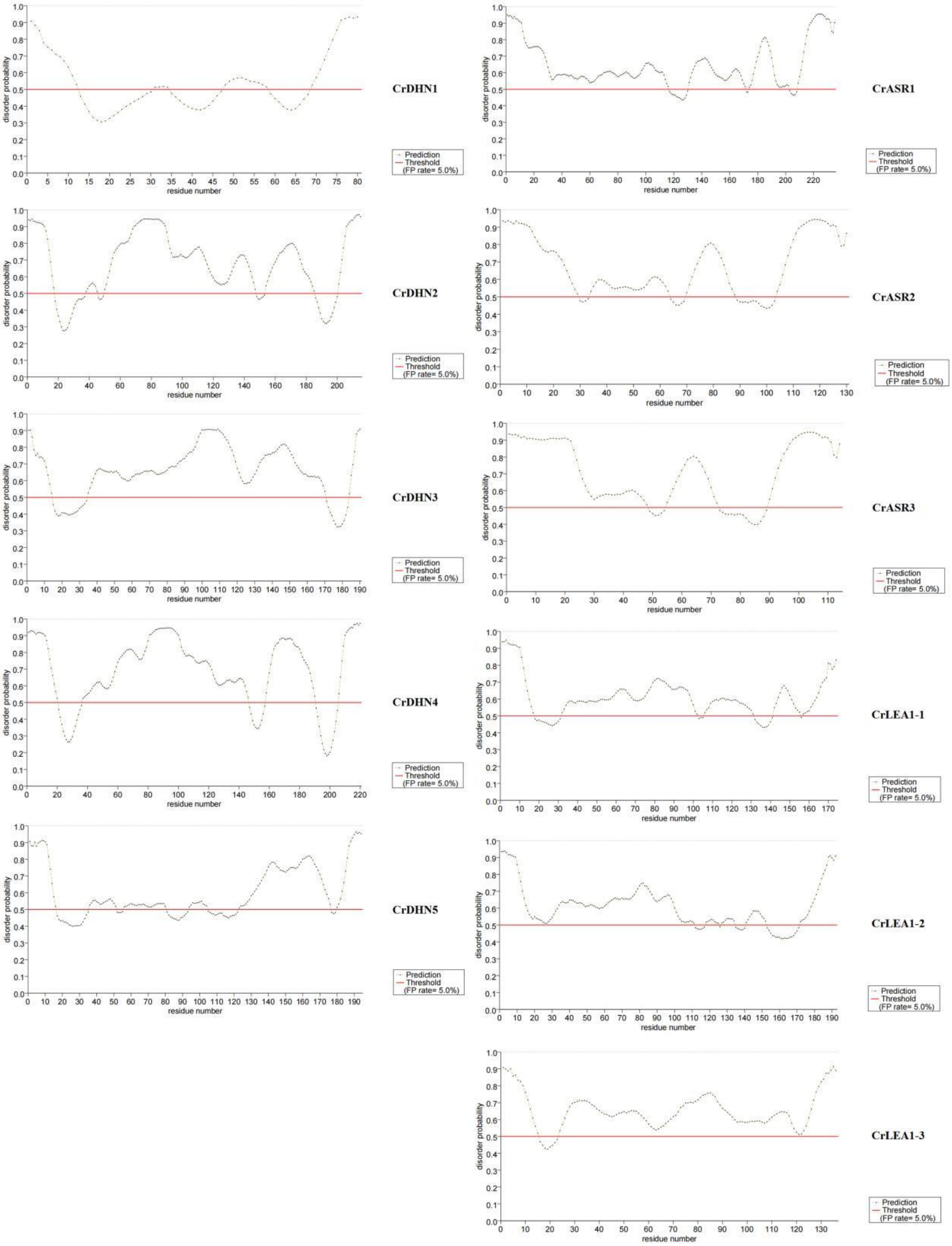
CrDHN1
CrASR1
CrDHN2
CrASR2
CrDHN3
CrASR3
CrDHN4
CrLEA1-1
CrDHN5
CrLEA1-2
CrLEA1-3
disorder probability
residue number
Prediction
Threshold
(FP rate= 5.0%)

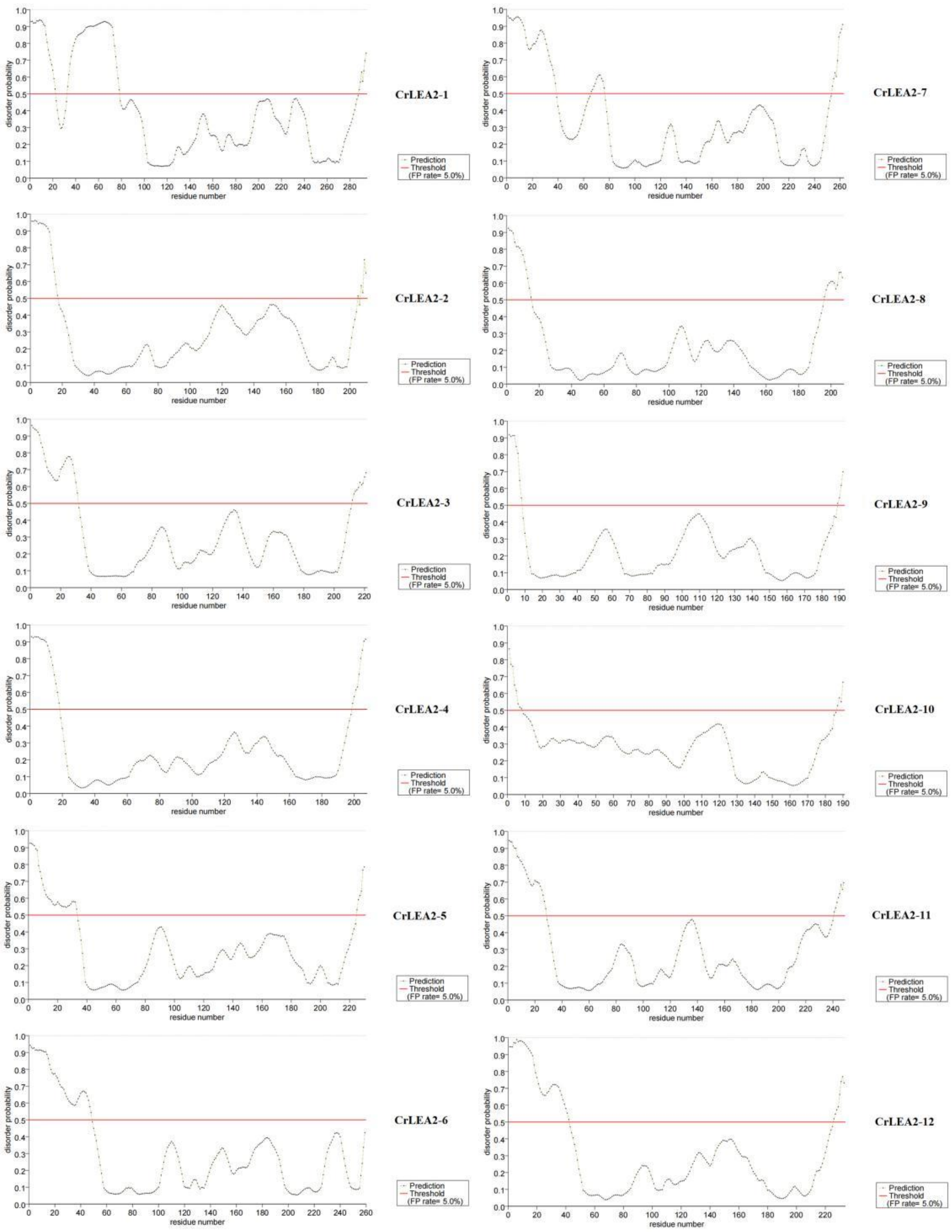
disorder probability
residue number
Prediction
Threshold
(FP rate= 5.0%)
CrLEA2-1
CrLEA2-7
CrLEA2-2
CrLEA2-8
CrLEA2-3
CrLEA2-9
CrLEA2-4
CrLEA2-10
CrLEA2-5
CrLEA2-11
CrLEA2-6
CrLEA2-12

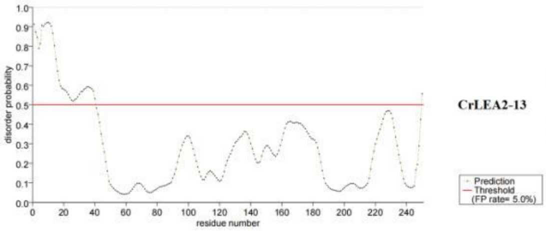

CrLEA2-13

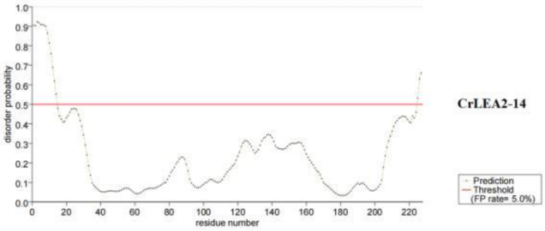

CrLEA2-14

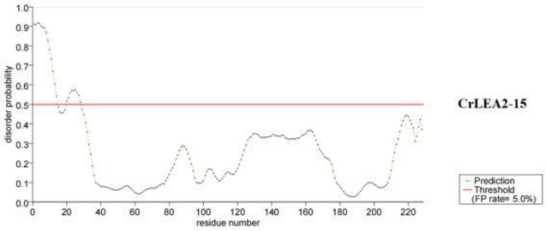

CrLEA2-15

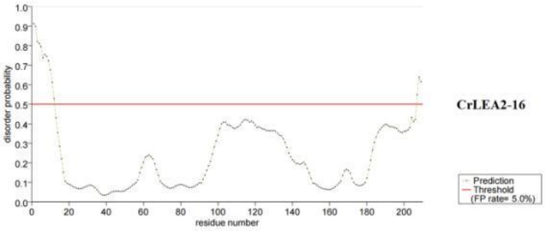

CrLEA2-16

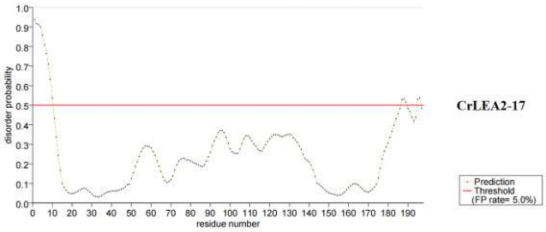

CrLEA2-17

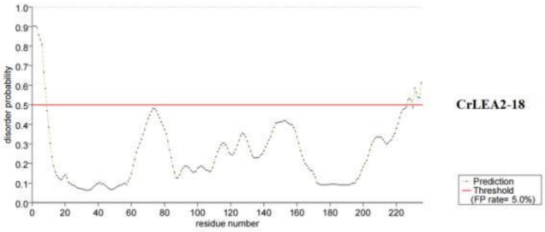

CrLEA2-18

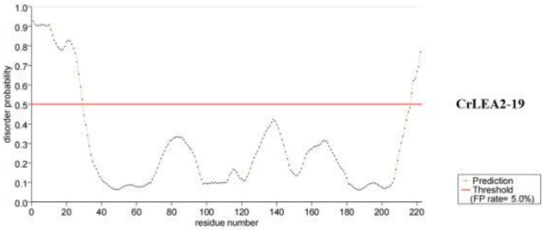

CrLEA2-19

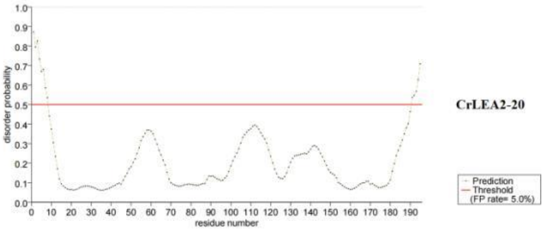

CrLEA2-20

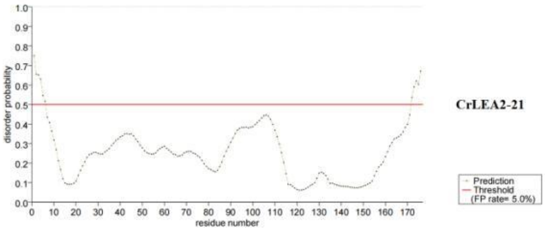

CrLEA2-21

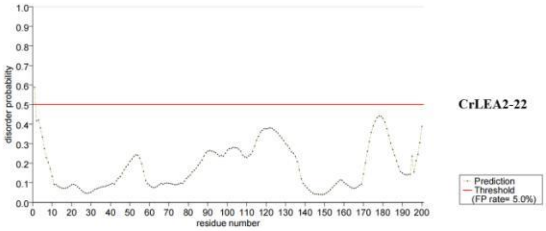

CrLEA2-22

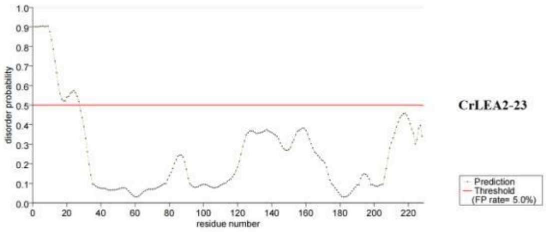

CrLEA2-23

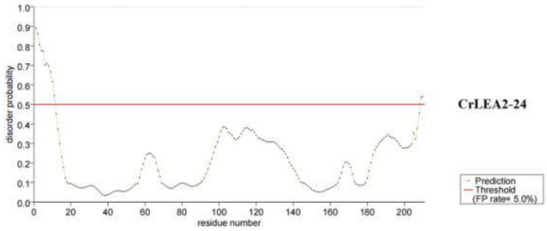

CrLEA2-24

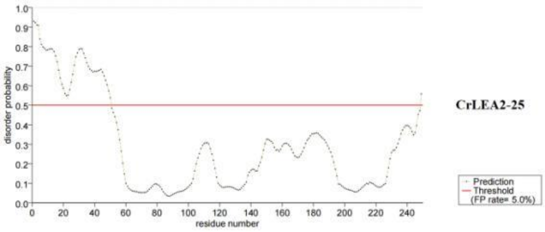

CrLEA2-25

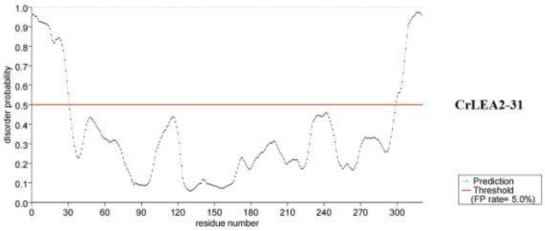

CrLEA2-31

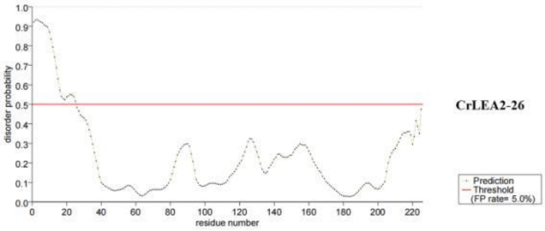

CrLEA2-26

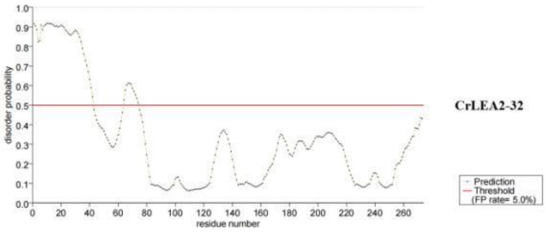

CrLEA2-32

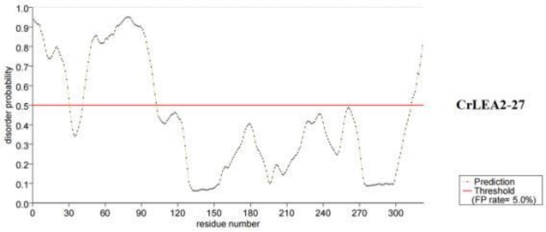

CrLEA2-27

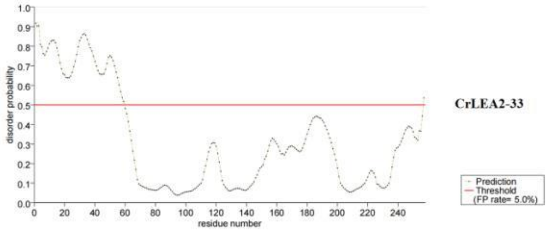

CrLEA2-33

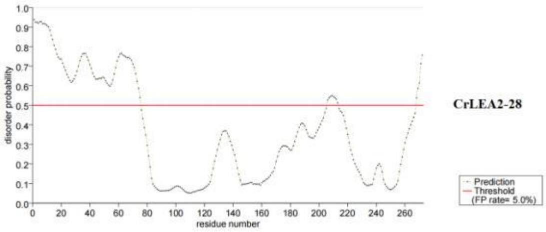

CrLEA2-28

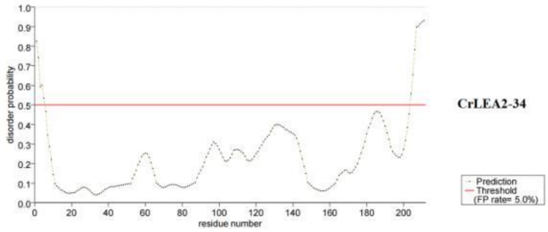

CrLEA2-34

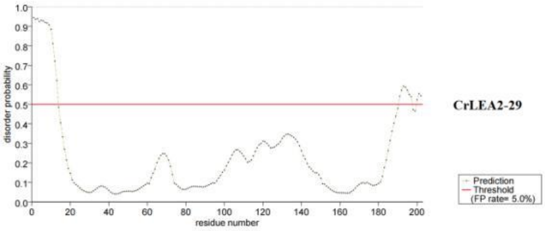

CrLEA2-29

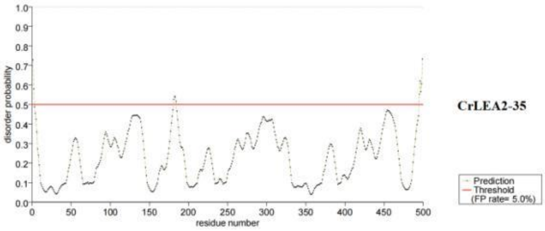

CrLEA2-35

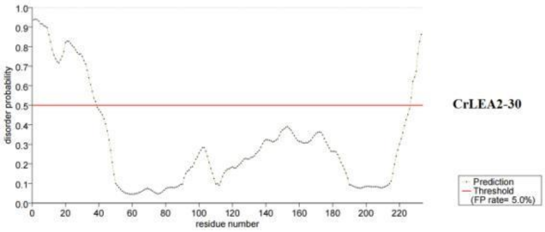

CrLEA2-30

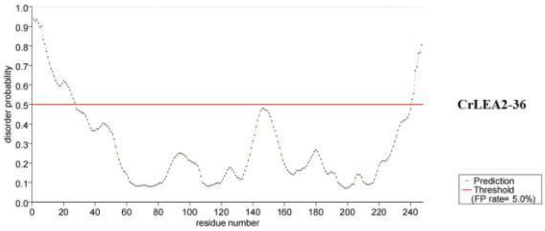

CrLEA2-36

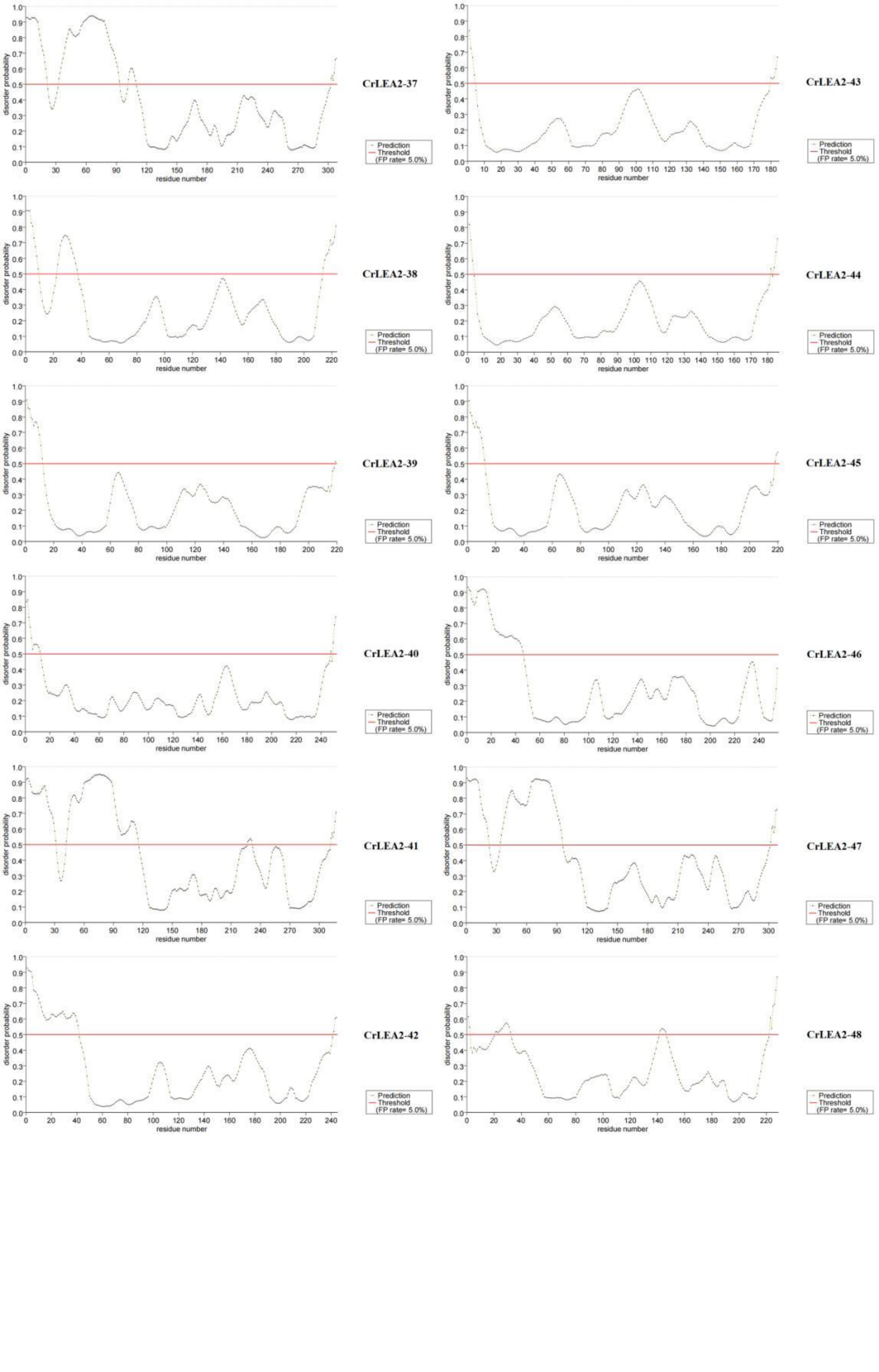
CrLEA2-37
CrLEA2-43
CrLEA2-38
CrLEA2-44
CrLEA2-39
CrLEA2-45
CrLEA2-40
CrLEA2-46
CrLEA2-41
CrLEA2-47
CrLEA2-42
CrLEA2-48
disorder probability
residue number
Prediction
Threshold
(FP rate= 5.0%)

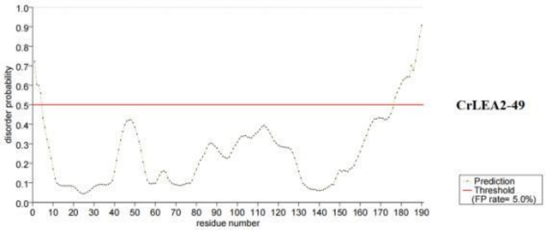

CrLEA2-49

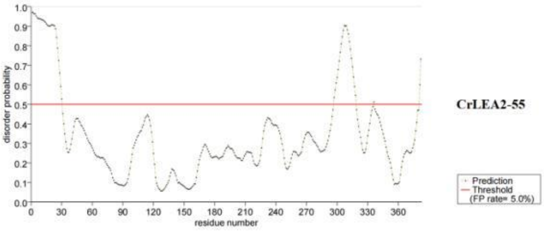

CrLEA2-55

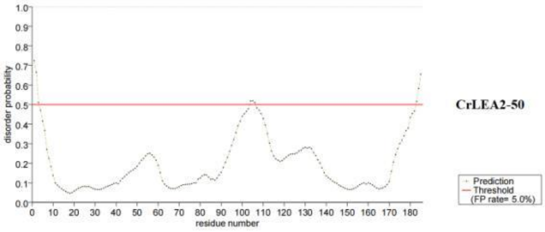

CrLEA2-50

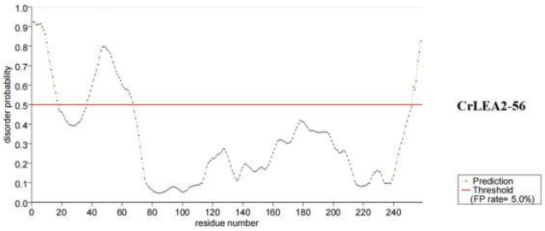

CrLEA2-56

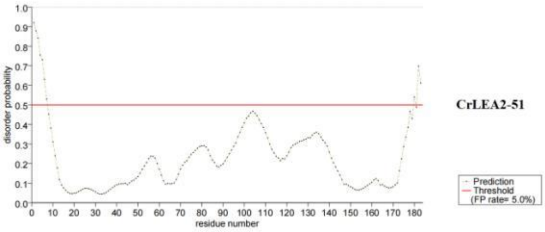

CrLEA2-51

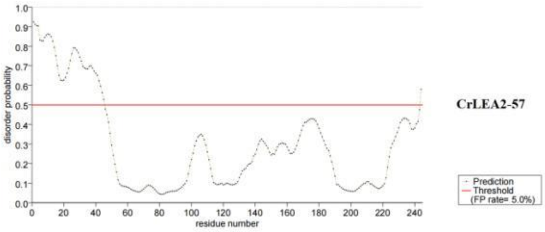

CrLEA2-57

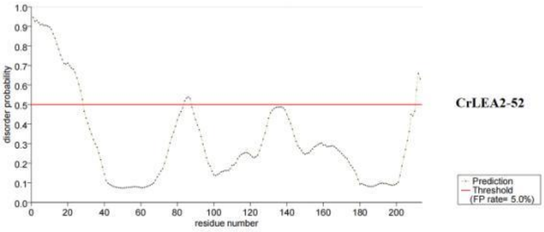

CrLEA2-52

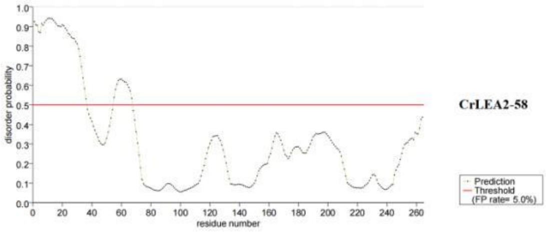

CrLEA2-58

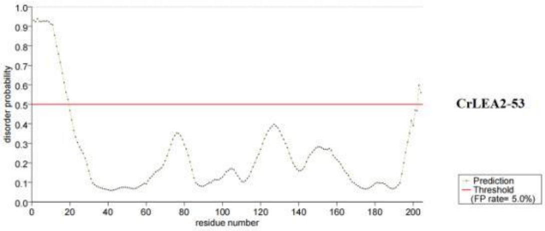

CrLEA2-53

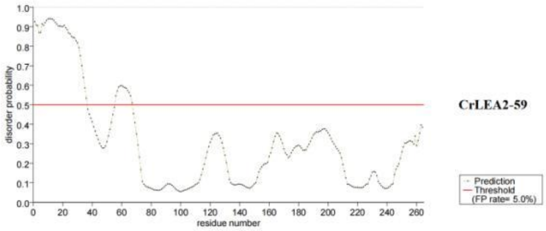

CrLEA2-59

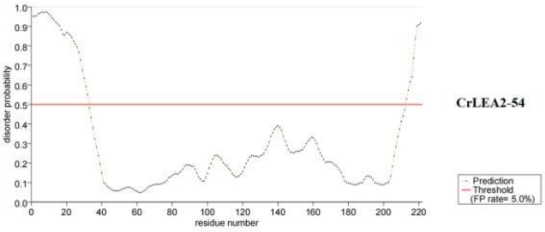

CrLEA2-54

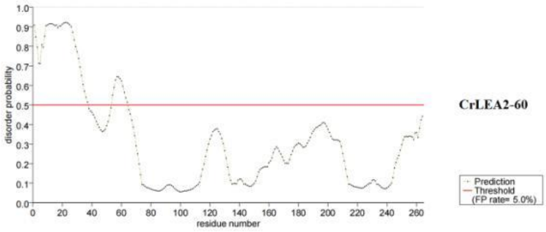

CrLEA2-60

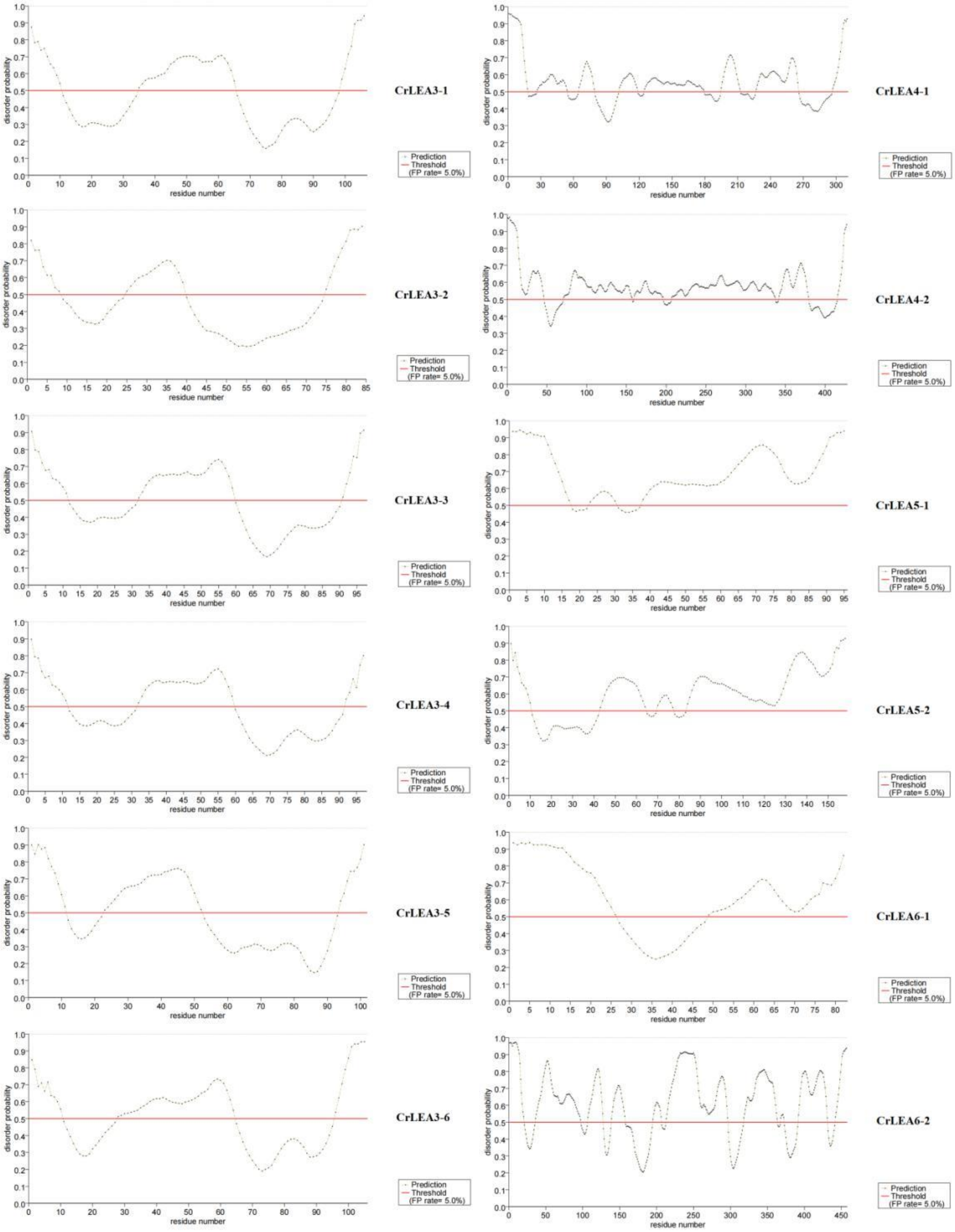
CrLEA3-1
CrLEA4-1
CrLEA3-2
CrLEA4-2
CrLEA3-3
CrLEA5-1
CrLEA3-4
CrLEA5-2
CrLEA3-5
CrLEA6-1
CrLEA3-6
CrLEA6-2
disorder probability
residue number
Prediction
Threshold
(FP rate= 5.0%)

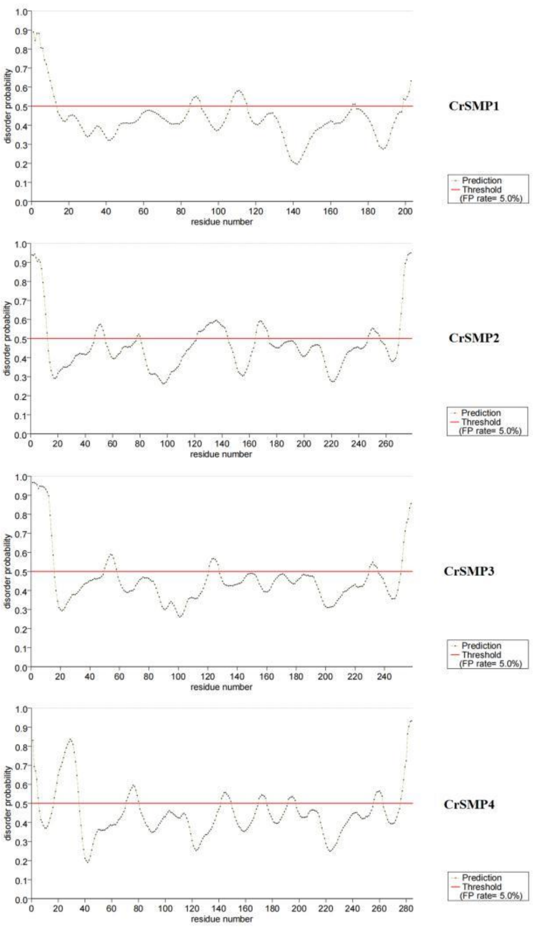

Figure S2. Predicted disorder profile plots of CrLEA and CrASR proteins.

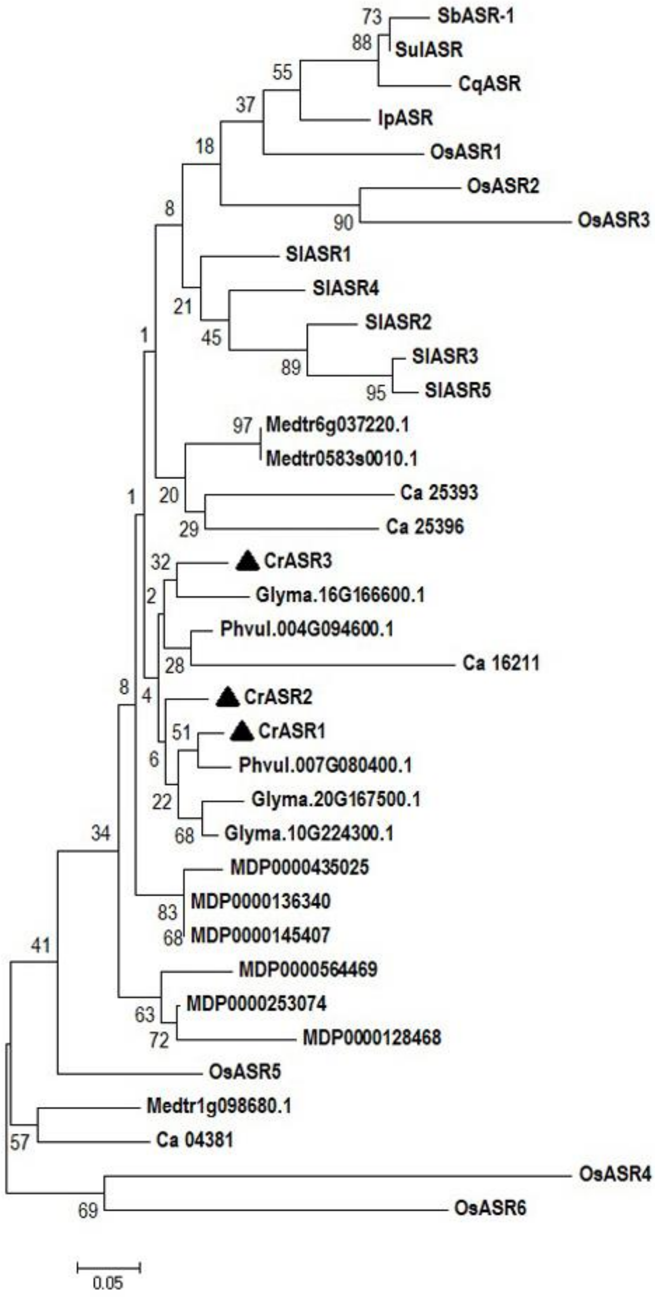

Figure S3. Phylogenetic tree of the plant ASR members.
